# Biased Agonism at Nociceptin/Orphanin FQ Receptors: A Structure Activity Study on N/OFQ(1–13)-NH_2_

**DOI:** 10.1021/acs.jmedchem.9b02057

**Published:** 2020-09-09

**Authors:** Salvatore Pacifico, Federica Ferrari, Valentina Albanese, Erika Marzola, Joaquim Azevedo Neto, Chiara Ruzza, Girolamo Calò, Delia Preti, Remo Guerrini

**Affiliations:** † Department of Chemical and Pharmaceutical Sciences, University of Ferrara, Via Luigi Borsari 46, 44121 Ferrara, Italy; ‡ Department of Medical Sciences, Section of Pharmacology, University of Ferrara, Via Fossato di Mortara 17/19, 44121 Ferrara, Italy; § LTTA Laboratory for Advanced Therapies, Technopole of Ferrara, Via Fossato di Mortara 70, 44121 Ferrara, Italy

## Abstract

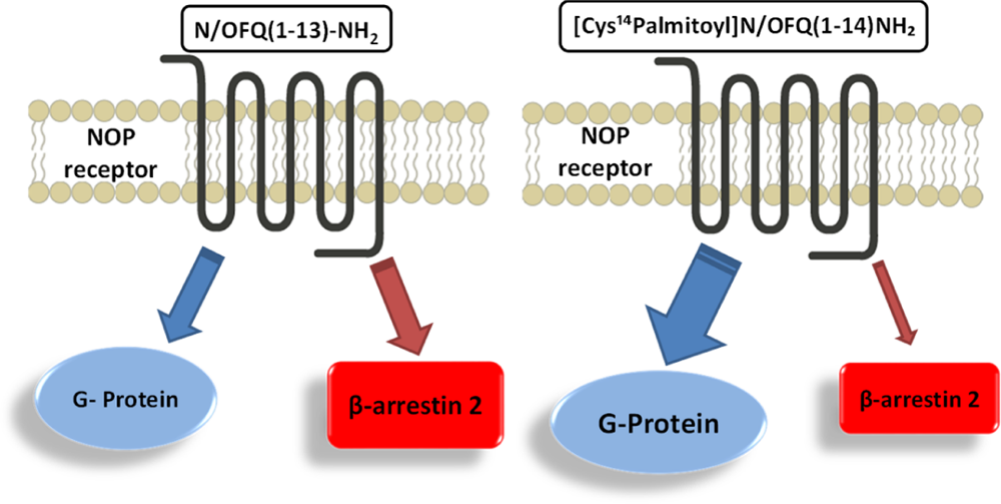

Nociceptin/orphanin FQ (N/OFQ) controls different biological functions *via* selective stimulation of the N/OFQ peptide (NOP) receptor. The pleiotropic actions of N/OFQ may limit the development of NOP ligands as innovative drugs in different therapeutic areas. The pharmacological concept of functional selectivity (aka biased agonism) might be useful for amplifying beneficial actions and/or counteracting side effects. Thus, molecules with large bias factors toward G protein or β arrestin are required for investigating the translational value of NOP biased modulation. Herein, the biased behavior of a heterogeneous library of NOP-targeting peptide derivatives was evaluated *in vitro* with the aim to provide possible insights into the structural determinants that govern the selective activation of G protein *versus* β-arrestin. Our results demonstrate that lipidation of N/OFQ(1–13)-NH_2_ is a useful strategy for obtaining G protein biased agonists for the NOP receptor.

## Introduction

The peptide nociceptin/orphanin FQ (Phe-Gly-Gly-Phe-Thr-Gly-Ala-Arg-Lys-Ser-Ala-Arg-Lys-Leu-Ala-Asn-Gln, N/OFQ) is the endogenous ligand of the Gi coupled N/OFQ peptide (NOP) receptor.^1,2^ Previous structure–activity relationship studies, that have been recently reviewed,^3^ suggested that the N/OFQ N-terminal message domain (Phe-Gly-Gly-Phe) binds the NOP binding pocket while the address domain of the peptide (Thr-Gly-Ala-Arg-Lys-Ser-Ala-Arg-Lys-Leu-Ala-Asn-Gln) interacts with the second extracellular loop of the receptor. This proposal has been confirmed by the available NOP crystal structure studies.^4,5^ The N/OFQ-NOP receptor system regulates several biological functions, including locomotor activity, memory, emotional states, food intake, drug abuse, pain transmission, micturition, cough reflexes, and cardiovascular, respiratory, and immune functions.^1,2^ Thus, the NOP receptor is an attractive target for the development of innovative drugs, and there is now clinical evidence for possible indications of NOP ligands, including systolic hypertension for the partial agonist SER100,^6^ urinary incontinence due to overactive bladder for the full agonists N/OFQ and Rec 0438,^7^ pain for the mixed NOP/opioid receptor agonist cebranopadol,^8^ and depression for the antagonist BTRX-246040.^9^

However, the pleiotropic effects exerted by N/OFQ and NOP agonists may be viewed as an obstacle in terms of drug development. To overcome this, the novel concept of functional selectivity or biased agonism might be useful.^10,11^ In fact, biased agonists are ligands able to stabilize different active conformations of the same receptor (*i.e.*, by promoting the interaction of the receptor with G protein better than arrestin or *vice versa*) thus eliciting distinct signaling patterns. In the opioid field, the observation that morphine displays a larger therapeutic index in β-arrestin 2 knockout mice^12,13^ suggested that mu receptor agonists biased toward G protein may maintain analgesic efficacy while showing reduced side effects compared to classical opioid drugs; this proposal has been confirmed with different molecules as previously reviewed.^14^ However, recent genetic and pharmacological studies do not support this concept.^15−17^ Regarding the NOP receptor, few studies investigated the ability of ligands to discriminate between G proteins and arrestins.^18,19^ Very recently, a comparison of structurally distinct NOP receptor agonists demonstrated a clear dissociation between G protein-dependent signaling and receptor phosphorylation.^20^ However, the *in vivo* consequences of these differences are largely unknown; the only information available suggests that NOP ligands, producing similar effects on NOP/G protein interactions but showing different effects on β-arrestin 2 recruitment, elicited different actions on anxiety and mood.^21^

To investigate the potential of biased agonism in the NOP receptor field, molecules displaying a large bias factor toward G protein and toward arrestin are needed. Thus, the aim of this study was to investigate the effector-specific (G protein *vs* arrestin) structure–activity relationship of the NOP peptide ligand N/OFQ(1–13)-NH_2_ (the smaller N/OFQ sequence acting as potent NOP full agonist^3,22^) in order to identify NOP receptor biased agonists. The ability of known (compounds **1–38**) and novel (compounds **39–56**) N/OFQ(1–13)-NH_2_ derivatives to promote NOP-G protein and NOP-arrestin interactions was investigated using a bioluminescence resonance energy transfer (BRET) assay. This assay originally set up for classical opioid receptors^23^ has been then extended to the NOP receptor and validated using a large panel of standard NOP ligands.^19^ Furthermore, novel NOP ligands have been more recently characterized using this NOP BRET assay, including the mixed NOP/opioid receptor agonists cebranopadol,^24^ DeNo,^25^ and PWT2-[Dmt^1^]N/OFQ(1–13),^26^ and the NOP selective agonists AT-403^27^ and AT-090.^21^ To quantify biased signaling, the Black/Leff operational model that provides a quantifiable and scalable method to characterize ligand bias was used.^28^

## Results and Discussion

### Chemistry

All peptide derivatives listed in Tables 1–5 (**1–38**) have been obtained through standard solid phase peptide synthesis (SPPS) in analogy to previously reported procedures and as described in the Experimental Section.^29−38^ The chemical structures of unusual amino acids have been reported in Table S1 of the Supporting Information.

**Table 1:** Effects of N/OFQ(1–13)-NH_2_ and Its Ala Scan Derivatives in NOP/G Protein and NOP/β-Arrestin 2 Experiments

		NOP/G protein			NOP/β-arrestin 2			
		pEC_50_ (CL_95%_)	CR	α ± sem	pEC_50_ (CL_95%_)	CR	α ± sem	bias factor (CL_95%_)
	N/OFQ(1–13)-NH_2_	8.80 (8.34–9.26)	1	1.00	8.26 (8.11–8.41)	1	1.00	0.00
**1**	[Ala^1^]N/OFQ(1–13)-NH_2_	Crc incomplete	∼700		inactive			
**2**	[Ala^2^]N/OFQ(1–13)-NH_2_	6.36 (5.89–6.83)	275	0.54 ± 0.07^a^	inactive			
**3**	[Ala^3^]N/OFQ(1–13)-NH_2_	7.88 (6.79–8.96)	8	0.87 ± 0.02	7.54 (7.22–7.86)	5	0.65 ± 0.02^a^	–0.21 (−0.96–0.55)
**4**	[Ala^4^]N/OFQ(1–13)-NH_2_	Crc incomplete	∼1000		inactive			
**5**	[Ala^5^]N/OFQ(1–13)-NH_2_	7.12 (6.61–7.64)	48	0.86 ± 0.02	6.13 (5.55–6.72)	135	0.72 ± 0.10^a^	0.40 (−0.24–1.05)
**6**	[Ala^6^]N/OFQ(1–13)-NH_2_	7.66 (6.59–8.72)	14	0.93 ± 0.04	7.10 (6.64–7.56)	14	0.97 ± 0.04	0.04 (−0.60–0.67)
**7**	[Ala^8^]N/OFQ(1–13)-NH_2_	Crc incomplete	∼1000		inactive			
**8**	[Ala^9^]N/OFQ(1–13)-NH_2_	8.14 (6.88–9.41)	5	0.97 ± 0.04	7.77 (7.43–8.11)	3	1.03 ± 0.07	–0.15 (−0.79–0.49)
**9**	[Ala^10^]N/OFQ(1–13)-NH_2_	9.25 (7.82–10.68)	0.35	0.94 ± 0.04	8.43 (7.91–8.95)	0.68	1.00 ± 0.04	–0.08 (−0.67–0.50)
**10**	[Ala^12^]N/OFQ(1–13)-NH_2_	7.31 (6.41–8.22)	31	0.91 ± 0.04	6.85 (6.57–7.14)	26	0.99 ± 0.05	–0.15 (−0.78–0.49)
**11**	[Ala^13^]N/OFQ(1–13)-NH_2_	7.91 (7.24–8.59)	8	0.96 ± 0.06	7.88 (7.75–8.01)	2	1.03 ± 0.05	–0.48 (−1.14–0.18)

a *p* < 0.05 vs N/OFQ(1–13)NH_2_ one-way ANOVA followed the Dunnett post hoc test. Data are expressed as the mean ± sem of five independent experiments made in duplicate; CR, concentration ratio; crc, concentration–response curve; CL_95%_, 95% confidence limits; sem, standard error of the mean.

**Table 2:** Effects of N/OFQ(1–13)-NH_2_ and Its D Scan Derivatives in NOP/G Protein and NOP/β-Arrestin 2 Experiments

		NOP/G protein			NOP/β-arrestin 2			
		pEC_50_ (CL_95%_)	CR	α ± sem	pEC_50_ (CL_95%_)	CR	α ± sem	bias factor (CL_95%_)
	N/OFQ(1–13)-NH_2_	8.37 (8.30–8.45)	1	1.00	8.02 (7.79–8.24)	1	1.00	0.00
**12**	[DPhe^1^]N/OFQ(1–13)-NH_2_	6.02 (5.36–6.69)	224	0.70 ± 0.03^a^	crc incomplete			
**13**	[DPhe^4^]N/OFQ(1–13)-NH_2_	5.99 (5.39–6.58)	240	0.79 ± 0.03^a^	crc incomplete			
**14**	[DThr^5^]N/OFQ(1–13)-NH_2_	crc incomplete			inactive			
**15**	[DAla^7^]N/OFQ(1–13)-NH_2_	7.26 (7.01–7.52)	13	0.95 ± 0.03	6.76 (6.56–6.96)	18	0.84 ± 0.03^a^	0.21 (−0.28–0.70)
**16**	[DArg^8^]N/OFQ(1–13)-NH_2_	7.03 (6.64–7.42)	22	0.95 ± 0.02	6.55 (6.31–6.79)	30	0.79 ± 0.02^a^	0.26 (−0.25–0.77)
**17**	[DLys^9^]N/OFQ(1–13)-NH_2_	7.01 (6.85–7.17)	23	0.94 ± 0.02	6.31 (6.00–6.62)	51	0.85 ± 0.06^a^	0.24 (−0.25–0.73)
**18**	[DSer^10^]N/OFQ(1–13)-NH_2_	7.80 (7.51–8.09)	4	1.08 ± 0.03	7.65 (7.49–7.80)	2	1.01 ± 0.03	–0.07 (−0.52–0.39)
**19**	[DAla^11^]N/OFQ(1–13)-NH_2_	7.40 (7.03–7.78)	9	1.07 ± 0.02	7.14 (7.01–7.27)	8	0.97 ± 0.04	0.14 (−0.31–0.60)
**20**	[DArg^12^]N/OFQ(1–13)-NH_2_	7.63 (7.38–7.88)	5	1.06 ± 0.01	7.39 (7.21–7.57)	4	0.94 ± 0.04	0.26 (−0.19–0.71)
**21**	[DLys^13^]N/OFQ(1–13)-NH_2_	8.34 (8.12–8.55)	1	1.07 ± 0.02	8.23 (8.12–8.34)	0.62	1.01 ± 0.04	0.03 (−0.42–0.48)

a *p* < 0.05 vs N/OFQ(1–13)NH_2_ one-way ANOVA followed by the Dunnett post hoc test. Data are expressed as the mean ± sem of five independent experiments made in duplicate.

**Table 3:** Effects of N/OFQ(1–13)-NH_2_ and Its Derivatives (Compounds **22**–**33**) in NOP/G Protein and NOP/β-Arrestin 2 Experiments

		NOP/G protein			NOP/β-arrestin 2			
		pEC_50_ (CL_95%_)	CR	α ± sem	pEC_50_ (CL_95%_)	CR	α ± sem	bias factor (CL_95%_)
	N/OFQ(1–13)-NH_2_	8.70 (8.34–9.06)	1	1.00	8.28 (8.03–8.53)	1	1.00	0.00
**22**	[Cha^1^]N/OFQ(1–13)-NH_2_	8.93 (8.57–9.29)	0.6	1.08 ± 0.04	8.28 (7.88–8.67)	1	1.03 ± 0.05	0.20 (−0.36–0.76)
**23**	[Leu^1^]N/OFQ(1–13)-NH_2_	8.19 (7.88–8.51)	3	1.06 ± 0.02	7.87 (7.69–8.04)	3	1.04 ± 0.06	–0.08 (−0.53–0.37)
**24**	[Trp^4^]N/OFQ(1–13)-NH^2^	7.63 (7.15–8.11)	12	1.01 ± 0.01	7.17 (7.00–7.33)	13	0.77 ± 0.03^a^	0.11 (−0.43–0.65)
**25**	[D-Phe^3^]N/OFQ(1–13)-NH_2_	inactive			inactive			
**26**	[D-Ala^2^]N/OFQ(1–13)-NH_2_	7.57 (7.28–7.86)	13	1.03 ± 0.03	7.16 (6.94–7.34)	13	0.95 ± 0.05	0.20 (−0.26–0.66)
**27**	[Nphe^1^]N/OFQ(1–13)-NH_2_	inactive			inactive			
**28**	[(*S*)βMeNphe^1^]N/OFQ(1–13)-NH_2_	inactive			inactive			
**29**	[(pF)Phe^4^]N/OFQ(1–13)-NH_2_	9.34 (9.14–9.50)	0.2	1.08 ± 0.04	8.40 (8.11–8.69)	0.8	0.93 ± 0.04	0.59^b^ (0.13–1.05)
**30**	[Phe^1^ψ(CH_2_–NH)Gly^2^]N/OFQ(1–13)-NH_2_	8.05 (7.66–8.44)	4	0.59 ± 0.03^a^	Inactive			
**31**	[Phe^1^ψ(CH_2_–S)Gly^2^]N/OFQ(1–13)-NH_2_	inactive			inactive			
**32**	[Asn^5^]N/OFQ(1–13)-NH_2_	8.03 (7.45–8.60)	5	1.01 ± 0.03	7.56 (7.11–8.00)	5	1.03 ± 0.07	–0.12 (−0.59–0.35)
**33**	[Val^5^]N/OFQ(1–13)-NH_2_	7.94 (7.47–8.42)	6	0.94 ± 0.05	7.38 (7.03–7.74)	8	0.93 ± 0.06	0.09 (−0.38–0.56)

a *p* < 0.05 vs N/OFQ(1–13)NH_2_ one-way ANOVA followed by the Dunnett post hoc test.

b Statistically different from 0. Data are expressed as the mean ± sem of five independent experiments made in duplicate.

**Table 4:** Antagonist Potency of N/OFQ(1–13)-NH_2_ Derivatives in NOP/G Protein and NOP/β-arrestin 2 Experiments^a^

		p*A*_2_ (CL_95%_)	
		NOP/G protein	NOP/β-arrestin 2
**27**	[Nphe^1^]N/OFQ(1–13)-NH_2_	7.51 (6.83–8.19)	7.13 (6.85–7.41)
**28**	[(*S*)βMeNPhe^1^]N/OFQ(1–13)-NH_2_	7.86 (7.28–8.44)	7.67 (6.91–8.43)
**30**	[Phe^1^ψ(CH_2_–NH)Gly^2^]N/OFQ(1–13)-NH_2_	8.23 (7.24–9.22)	7.83 (7.64–8.02)
**31**	[Phe^1^ψ(CH_2_–S)Gly^2^]N/OFQ(1–13)-NH_2_	6.88 (6.00–7.76)	6.99 (6.86–7.12)

a Data are expressed as the mean ± sem of five independent experiments made in duplicate.

**Table 5:** Effects of N/OFQ-NH_2_ and Its Derivatives (Compounds **34–38**) in NOP/G Protein and NOP/β-Arrestin 2 Experiments^a^

		NOP/G protein			NOP/β-arrestin 2			
		pEC_50_ (CL_95%_)	CR	α ± sem	pEC_50_ (CL_95%_)	CR	α ± sem	bias factor (CL_95%_)
	N/OFQ(1–13)-NH_2_	8.08 (7.70–8.45)	1	1.00	8.01 (7.80–8.22)	1	1.00	0.00
**34**	[Aib^7^]N/OFQ-NH_2_	8.92 (8.68–9.17)	0.2	1.08 ± 0.04	8.40 (8.25–8.55)	0.4	0.98 ± 0.05	0.46 (−0.08–1.01)
**35**	[Aib^11^]N/OFQ-NH_2_	8.35 (7.84–8.86)	0.5	1.07 ± 0.03	8.21 (8.13–8.29)	0.6	0.95 ± 0.03	0.29 (−0.20–0.79)
**36**	[AC_3_C^7^]N/OFQ-NH_2_	7.67 (7.11–8.23)	3	1.13 ± 0.04	7.56 (7.40–7.73)	3	1.01 ± 0.04	0.40 (−0.11–0.90)
**37**	[AC_5_C^7^]N/OFQ-NH_2_	8.10 (7.59–8.61)	1	1.11 ± 0.02	8.10 (7.86–8.34)	0.8	1.02 ± 0.03	0.08 (−0.41–0.58)
**38**	[AC_5_C^11^]N/OFQ-NH_2_	8.61 (8.20–9.03)	0.3	1.09 ± 0.04	8.22 (8.08–8.35)	0.6	1.05 ± 0.03	0.31 (−0.19–0.81)

a Data are expressed as the mean ± sem of five independent experiments made in duplicate.

**Scheme 1:**
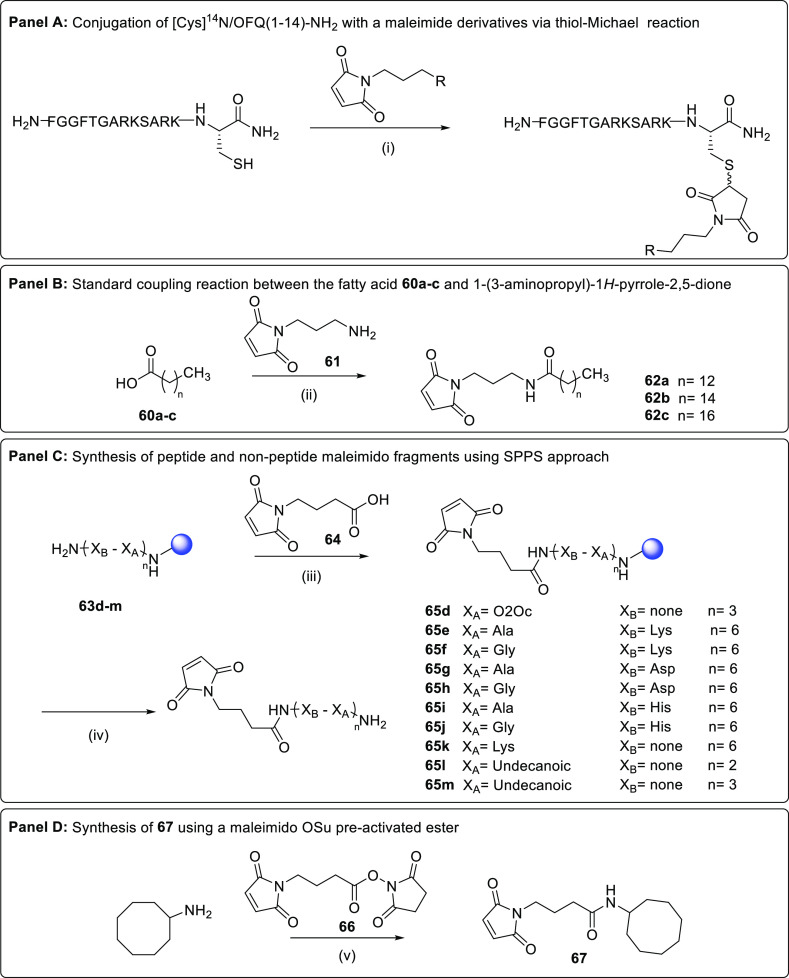
Reagents and conditions: (i) NaHCO_3_, CH_3_CN/H_2_O, 5 min; (ii) HATU, DIPEA, DMF, 0 °C to rt, 12 h; (iii) WSC, HOBt, DMF, rt, 3 h; (iv) TFA/H_2_O/triethylsilane, rt, 3 h; and (v) Et_3_N, CH_3_CN, rt, 0.5 h.

In order to obtain the conjugated peptide derivatives reported in Tables 6 and 7, the sequence of N/OFQ(1–13)-NH_2_ was initially elongated at the C-terminal position (for the obtainment of compounds **39–52**) or replaced at the 10–13 positions (for compounds **53–56**) with a cysteine residue. As described in Scheme 1 (panel A), [Cys^14^]N/OFQ(1–14)-NH_2_, previously synthesized in SPPS, was efficiently reacted with various maleimide containing derivatives through a thiol-Michael reaction carried out in liquid phase conditions. The same approach has been used for the synthesis of peptides substituted with Cys in positions 10, 11, 12, and 13. Compounds **39–41** and **53–56** were obtained through the reaction of the cysteine-modified peptide precursors with a maleimide-functionalized fatty acid (*i.e.*, myristic, palmitic, and stearic acid). The latter compounds **62a–c** (Scheme 1, panel B) were prepared by a standard coupling reaction between the proper fatty acid and the *N*-(4-aminobutyl)maleimide derivative **61**.

**Table 6:**
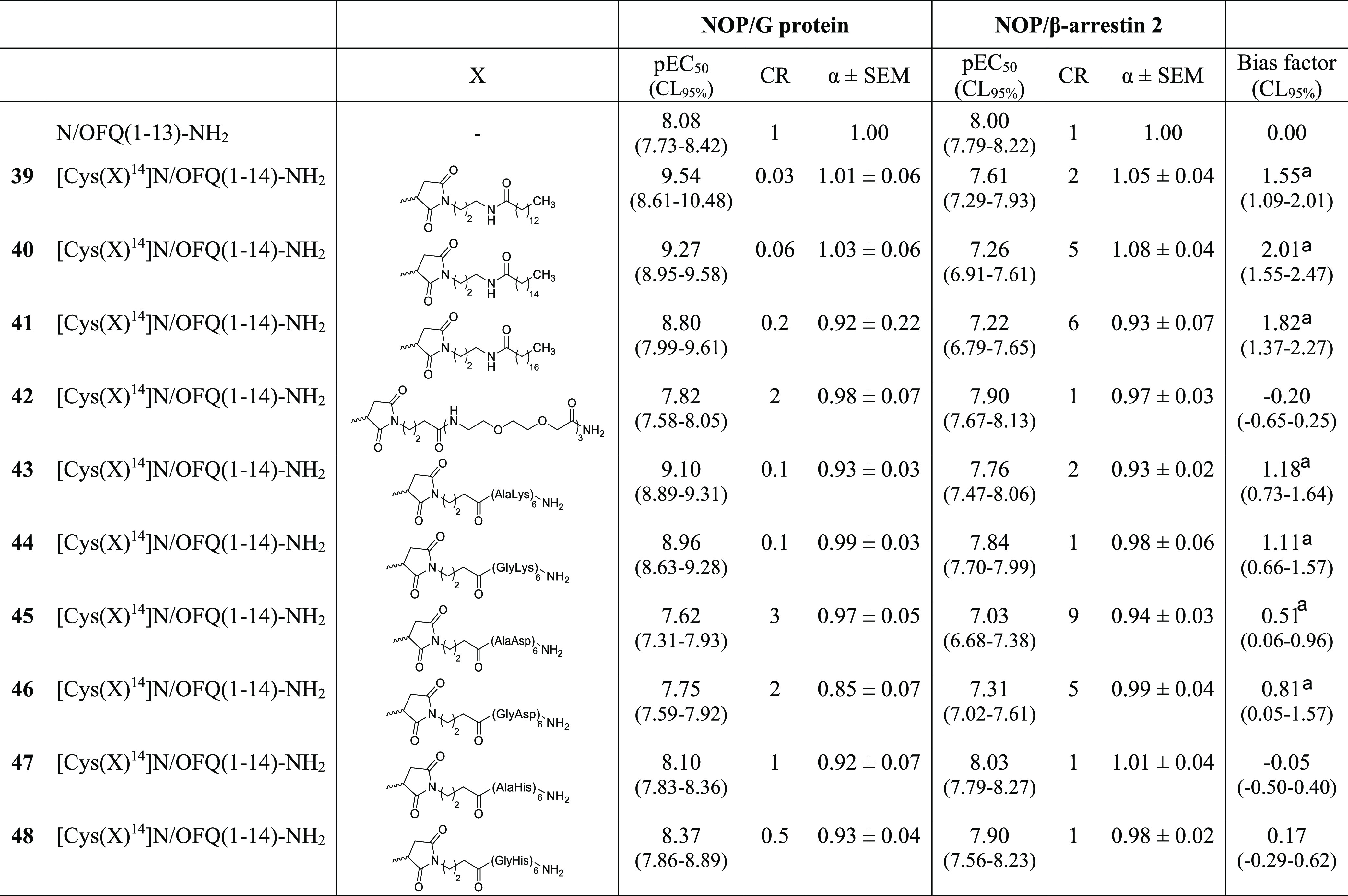
Effects of N/OFQ(1–13)NH_2_ and Its Derivatives from **39** to **48** in NOP/G Protein and NOP/β-Arrestin 2

a Statistically different from 0. Data are expressed as the mean ± sem of five independent experiments made in duplicate.

**Table 7:**
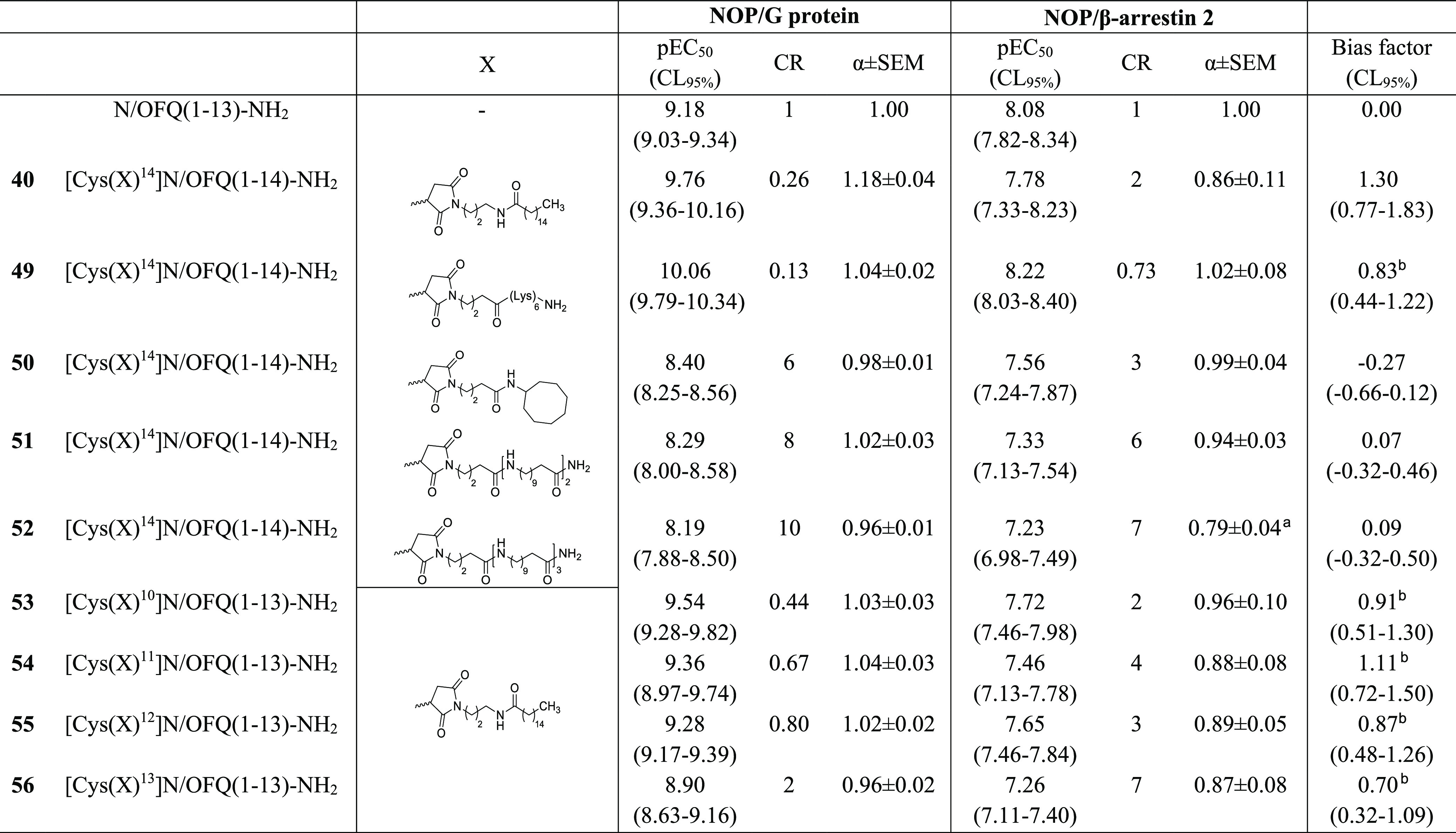
Effects of N/OFQ(1–13)NH_2_ and Compounds **40**, **49**–**56** in NOP/G Protein and NOP/β-Arrestin 2

a *p* < 0.05 vs N/OFQ one-way ANOVA followed by the Dunnett post hoc test.

b Statistically different from 0. Data are expressed as the mean ± sem of five independent experiments made in duplicate.

Compounds **42–49**, **51**, and **52** were synthesized following a convergent scheme in which [Cys^14^]N/OFQ(1–14)-NH_2_ was grafted *via* a thiol-Michael reaction to different pre-assembled peptide and non-peptide fragments (**65d–m**) prepared up front by solid phase synthesis and bearing at the N-terminal position a 4-(2,5-dioxo-2,5-dihydro-1*H*-pyrrol-1-yl)butanoic moiety (Scheme 1, panel C).

Moreover, compound **50** was obtained through the addition reaction of [Cys^14^]N/OFQ(1–14)-NH_2_ to the cyclooctyl amine derivative **67** which was previously prepared using the active succinic ester-activated derivative of the butanoic maleimide and the cyclooctyl amine, as described in Scheme 1 (panel D).

Finally, the palmitoylated opioid peptides **57–59** in Tables 8 and 9 were prepared from the corresponding [Cys^8^]dermorphin-NH_2_, [Cys^8^]deltorphin A-NH_2_, and [Cys^6^]Leu-enkephalin-NH_2_ peptide derivatives, obtained in SPPS and coupled *via* thiol-Michael to the maleimide derivative of palmitic acid (**62b**).

**Table 8:**
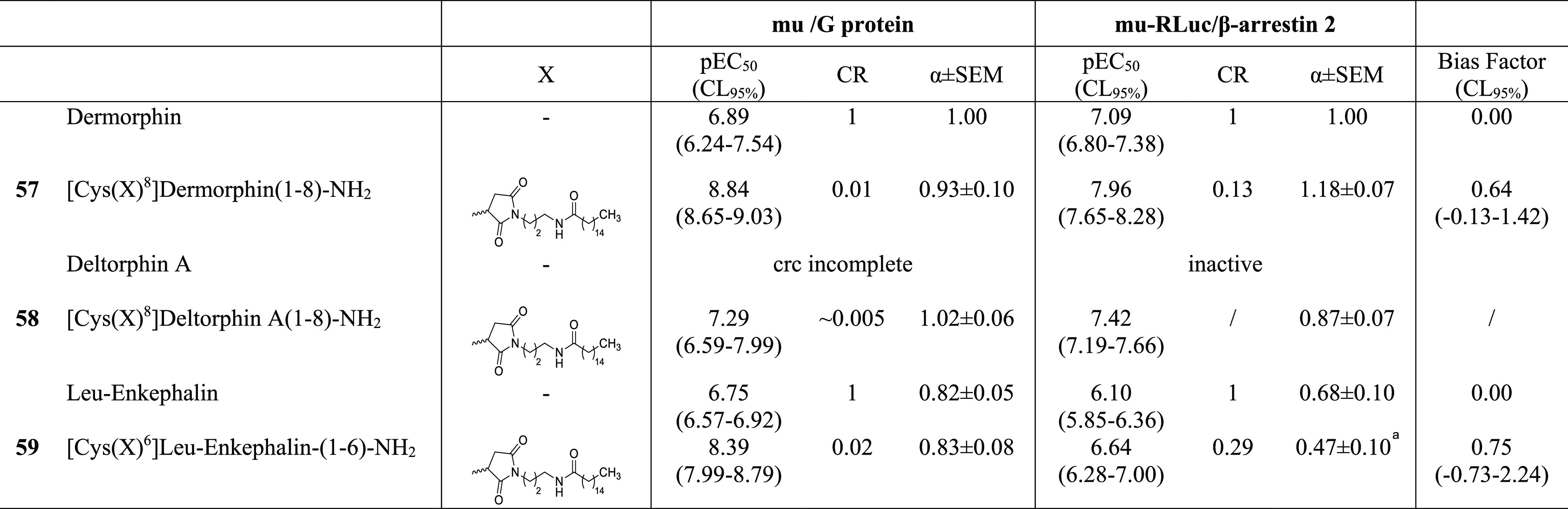
Effects of Dermorphin, Deltorphin A, Leu-Enkephalin, and Their C-Terminal Palmitoylate Analogues **57–59** in mu/G Protein and mu/β-Arrestin 2 Experiments

a *p* < 0.05 vs dermorphin, one-way ANOVA followed by the Dunnett post hoc test. Data are expressed as the mean ± sem of five independent experiments made in duplicate. RLuc, renilla luciferase.

**Table 9:**
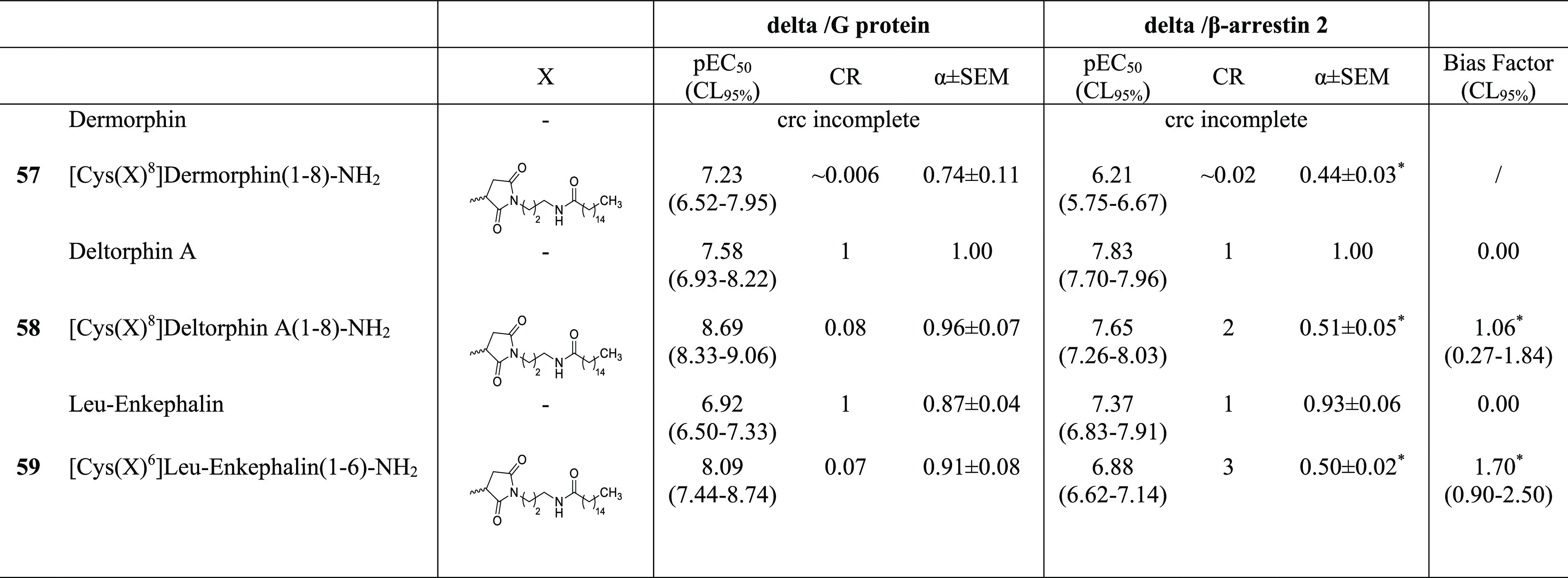
Effects of Dermorphin, Deltorphin A, Leu-Enkephalin, and Their C-Terminal Palmitoylate Analogues **57–59** in Delta/G Protein and Delta/β-Arrestin 2 Experiments

a *: *p* < 0.05 vs deltorphin A , one-way ANOVA followed by the Dunnett post hoc test. *: statistically different from 0. Data are expressed as the mean ± sem of five independent experiments made in duplicate.

As described above, the strategy for the synthesis of the new peptide conjugates **39–59** consisted of a convergent approach combining SPPS and a thiol-Michael conjugation reaction performed in liquid phase conditions. The overall process required the preparation of the target peptide sequence with an additional cysteine residue whose thiol function was exploited as anchoring point for the final derivatization. The fragments to be linked to the peptide sequence were independently synthesized (through SPPS or in liquid phase) and conveniently functionalized with a maleimide reactive group. The final thiol-Michael reaction was generally carried out in very mild conditions using only a small excess of the thiol reactant. As previously observed,^39^ this conjugation step confirmed its effectiveness, chemoselectivity, and utility because a complete conversion of the maleimide acceptor was detected within only 5 min of reaction, thus allowing an easy final purification of the desired products in quantitative yields. The methodology was particularly useful for the preparation of long peptide sequences or lipidated peptides that are problematic to be fully synthesized through SPPS because of solubility concerns. Moreover, this would require the use of an excess of lipidated Fmoc amino acids that are generally characterized by high costs.

Of note, the developed modular synthesis is particularly versatile and allows extending all modifications herein considered to be virtually any bioactive peptide sequence in which a cysteine residue can be added without compromising its pharmacological activity.

### Pharmacology

In the first series of studies, we assessed in parallel experiments the ability to promote NOP/G protein and NOP/β-arrestin 2 interactions of N/OFQ(1–13)-NH_2_, of its Ala- (**1–11**; Table 1) and D-scan (**12–21**; Table 2) derivatives as well as of analogues modified either in the amino acid side chain or in the peptide bond (**22–33**; Table 3). N/OFQ(1–13)-NH_2_ promoted NOP/G protein interactions in a concentration-dependent manner with high potency (pEC_50_ 8.80). All Ala-scan derivatives showed similar maximal effects as N/OFQ(1–13)-NH_2_ with the exception of [Ala^2^]N/OFQ(1–13)-NH_2_ that behaved as a low potency partial agonist. Moreover, compounds replaced in positions 1, 4 and 8 displayed very low potency, being able to promote the NOP/G protein interaction only at micromolar concentrations. N/OFQ(1–13)-NH_2_ stimulated the interaction of the NOP receptor with β-arrestin 2 in a concentration-dependent manner with high potency (pEC_50_ 8.26). Ala-scan derivatives of N/OFQ(1–13)-NH_2_ compounds **1–11** stimulated the interaction of the NOP receptor with β-arrestin 2, showing the same rank of potency as displayed in NOP/G protein experiments (Table 1).

Regarding NOP/G protein interactions, D-scan derivatives **12–21** displayed variable results depending on the position of the amino acid investigated. The inversion of the configuration of the chiral amino acids of the message domain produced drastic (>100-fold) reduction of potency. When the same modification was applied from the position 7 to 9, a moderate (>10-fold) reduction in potency was observed while modifications of the C-terminal of the peptide produced minor effects on peptide potency (<10-fold). Regarding NOP/β-arrestin 2 interactions, D-scan derivatives of N/OFQ(1–13)-NH_2_ showed the same rank of potency as displayed in NOP/G protein experiments (Table 2).

As far as N/OFQ(1–13)-NH_2_ analogues modified in the amino acid side chain of the message domain are concerned, all derivatives displayed full agonist activity at NOP/G protein with the exception of [Nphe^1^]N/OFQ(1–13)-NH_2_ (**27**) that was devoid of efficacy. Moreover, the potency of these compounds was similar to that of N/OFQ(1–13)-NH_2_ with the exception of [(pF)Phe^4^]N/OFQ(1–13)-NH_2_ (**29**) that was 5-fold more potent and [Trp^4^]N/OFQ(1–13)-NH_2_ (**24**) and [d-Ala^2^]N/OFQ(1–13)-NH_2_ (**26**) that were approximately 10-fold less potent. Finally, the replacement of the first peptide bond with Ψ(CH_2_–NH) (**30**) or Ψ(CH_2_–S) (**31**) caused reduction or elimination of peptide efficacy, respectively. Regarding NOP/β-arrestin 2 interactions, N/OFQ(1–13)-NH_2_ analogues showed the same rank of potency as displayed in NOP/G protein experiments (Table 3). The behavior of [(pF)Phe^4^]N/OFQ(1–13)-NH_2_ (**29**) was however slightly different: in fact, as mentioned above, it displayed increased potency in NOP/G protein compared to N/OFQ(1–13)-NH_2_ while similar potency in NOP/β-arrestin 2 experiments. This caused a small (3-fold) but statistically significant bias toward G protein for this NOP ligand.

Compounds inactive as agonists were tested as antagonists against N/OFQ (Table 4). 10 μM [Nphe^1^]N/OFQ(1–13)-NH_2_ (**27**) antagonized N/OFQ stimulatory effects, showing similar p*A*_2_ values in NOP/G protein and NOP/β-arrestin 2 experiments. The addition of a methyl group on Nphe as in the [(*S*)βMeNphe^1^]N/OFQ(1–13)-NH_2_ (**28**) produced a slight increase in potency with no changes in antagonist activity. [Phe^1^Ψ(CH_2_–NH)Gly^2^]N/OFQ(1–13)-NH_2_ (**30**) behaved as partial agonist in NOP/G protein and as pure antagonist in NOP/β-arrestin 2 studies; its agonist potency for promoting NOP/G protein interactions and its antagonist potency for blocking NOP/β-arrestin 2 interactions were in the range 7.83–8.23. Finally, the substitution of the CH_2_–NH bond between Phe^1^ and Gly^2^ with CH_2_–S (compound **31**) caused a complete elimination of efficacy in NOP/G protein experiments that was however associated with an approximately 10-fold reduction in antagonist potency.

As far as N/OFQ analogues modified in the address domain (Table 5), all derivatives displayed full agonist activity at NOP/G protein with minor modifications of potency: the most potent agonist was [Aib^7^]N/OFQ-NH_2_ (**34**) and the least potent was [AC_3_C^7^]N/OFQ-NH_2_ (**36**). Regarding NOP/β-arrestin 2 interactions, N/OFQ analogues showed the same rank of potency as displayed in NOP/G protein experiments.

The above mentioned experiments were aimed at investigating the possible biased agonist activity of known N/OFQ analogues, including Ala- and D-scan derivatives^29,30^ as well as peptides replaced in different amino acid positions or containing peptide bond modifications.^31−38^ The results obtained with these peptides in the NOP/G protein BRET assay perfectly confirmed previous findings regarding the crucial role of the side chain of residues in positions 1, 2, 4, 8 and of the chirality of residues in positions 1, 4 and 5.^29,30^ Moreover, as previously demonstrated, the first peptide bond and the benzyl moiety at the position 1 affect ligand efficacy,^32,33^ while alpha helix inducing amino acids at the 7 and 11 positions^36,37^ and the introduction of a fluorine atom in the para position of Phe^4^ promote an increase of agonist potency.^34^

In addition, these experiments allowed appreciating features of some NOP peptide ligands not detected in previous studies. For instance, [(*S*)βMeNphe^1^]N/OFQ(1–13)-NH_2_ (**28**) was slightly more potent than [Nphe^1^]N/OFQ(1–13)-NH_2_ (**27**) both in NOP/G protein and NOP/β-arrestin 2 experiments while it was reported as equipotent in previous studies.^35^ [Phe^1^Ψ(CH_2_–S)Gly^2^]N/OFQ(1–13)-NH_2_ (**31**) contrary to [Phe^1^Ψ(CH_2_–NH)Gly^2^]N/OFQ(1–13)-NH_2_ (**30**) behaved as pure antagonist in NOP/G protein experiments; this has not been appreciated in previous bioassay experiments performed in the electrically stimulated mouse vas deferens possibly because of off-target agonist effects.^38^

Results obtained with these peptides in the NOP/β-arrestin 2 assay were virtually superimposable to those of the NOP/G protein assay with the exceptions of compounds **29** and **30**. In fact, compound **29** displayed a small (3-fold) but statistically significant bias toward G protein. On the other hand, compound **30** ([Phe^1^Ψ(CH_2_–NH)Gly^2^]N/OFQ(1–13)-NH_2_) in line with the previous findings^19^ behaved as a partial agonist in NOP/G protein and as pure antagonist in NOP/β-arrestin 2 studies. This is an interesting feature, and compound **30** has been used as a pharmacological tool for performing initial studies on NOP functional selectivity that demonstrated that the action of a NOP ligand on emotional states is better predicted based on its β-arrestin 2 rather than G protein efficacy.^21^ Apart from these exceptions, all modified peptides behaved as unbiased NOP receptor agonists. This result is somewhat unexpected because similar subtle chemical modifications were sufficient for generating biased agonists when applied to other peptide sequences, including angiotensin, apelin, glucagon-like peptide, and parathormone.^40−43^ It could be speculated that for the NOP receptor, the chemical requirements of peptide agonists for promoting the interaction of the receptor with G protein are very similar to those required for promoting receptor/arrestin interactions. However, we cannot exclude that future studies may eventually identify small chemical modifications of the primary sequence of N/OFQ which were able to produce strongly biased agonists for the NOP receptor.

Collectively, the results obtained with the known N/OFQ derivatives demonstrated that their pharmacological activity was virtually identical in NOP/G protein and NOP/β-arrestin 2 experiments. In other words, all these compounds behaved, similarly to the natural peptide, as unbiased NOP agonists. Interestingly, a certain degree of biased agonism toward G protein has been detected in previous studies by investigating the pharmacological activity of tetrameric N/OFQ derivatives, including PWT2-N/OFQ^19^ and PWT2-[Dmt^1^]N/OFQ(1–13).^26^ These multimeric ligands can be considered as N/OFQ-related peptides modified with a rather hindered chemical group at the C-terminal.

On these bases, we designed novel N/OFQ derivatives modified at the C terminus with various chemical moieties, including lipophilic (compounds **39–41**), hydrophilic (compound **42**), positively charged (compounds **43**, **44**), negatively charged (compounds **45**, **46**), and aromatic (compounds **47**, **48**) moieties. The results obtained with these novel compounds are summarized in Table 6. Compounds **39–41** displayed, compared to N/OFQ(1–13)-NH_2_, higher potency at NOP/G protein and lower potency at NOP/β-arrestin 2, thus behaving as agonists biased toward G protein. Similar results were obtained with positively charged peptides (compounds **43**, **44**) whose bias factor was however lower than that of compounds functionalized with fatty acid chains. A statistically significant bias toward G protein was also displayed by negatively charged peptides (compounds **45**, **46**), but their bias factor was low and associated with reduced agonist potency. Finally, C-terminal modification of N/OFQ(1–13)-NH_2_ with hydrophilic neutral (compound **42**) or aromatic (compounds **47**, **48**) moieties did not produce significant changes in the pharmacological activity of the peptide.

In order to further investigate the contribution toward G protein bias of positive charges and lipophilicity of C-terminal-modified N/OFQ(1–13)-NH_2_ analogues, a second series of peptides was synthesized and tested, and the relative results are summarized in Table 7. Compound **49** characterized by a (Lys)_6_ moiety displayed a larger increase in potency at NOP/G protein than NOP/β-arrestin 2 interactions, showing a statistically significant bias factor of approximately 10-fold. The use of a cyclic aliphatic moiety (compound **50**) or of chains generated using two (compound **51**) or three (compound **52**) amino undecanoic acids caused a similar reduction of NOP/G protein and NOP/β-arrestin 2 potency, thus producing unbiased NOP agonists. The shift of the position of the palmitoyl moiety produced different results depending on the substituted amino acid. In particular, similar results were obtained when the palmitoyl moiety has been located in position 14 (compound **40**) and 11 (compound **54**), while, when the same moiety was introduced in position 10 (compound **53**), 12 (compound **55**), and 13 (compound **56**), it produced a variable decrease in potency and a consistent decrease in the bias factor.

The addition of neutral hydrophilic (**42**) or aromatic (**47**, **48**) moieties did not change the unbiased profile of the reference peptide. On the contrary, the addition of charged moieties particularly in the case of positively charged peptide sequences elicited a shift toward G protein biased agonism (**4344**, and **49**). This effect was mainly due to an increase in agonist potency for receptor/G protein interactions associated with no changes for receptor/arrestin interactions. The higher bias was detected with compound **43** characterized by the dipeptide sequence Ala–Lys repeating for six times. However, similar results were obtained with compound **44** and **49** in which the charges were organized in different ways. Thus, for promoting G protein bias, the presence of C-terminal positive charges seems to be more important than their spatial distribution and orientation. Interestingly enough, the potent and selective NOP agonist UFP-112^44^ that has an extra couple of positively charged residues (Arg^14^–Lys^15^) displayed a small but statistically significant G protein bias (0.71).^19^ Charged residues may promote receptor interactions *via* ionic bonds with acidic residues of which the second extracellular loop of the NOP receptor is rich.^45−48^ This proposal has been corroborated by the results obtained in molecular modeling studies based on the crystal structure of the NOP receptor (see for details Figure 4e in a study by Thompson *et al.* 2012^4^). As a speculative hypothesis, we might suggest that this mechanism is more effective for NOP conformations interacting with G proteins than for those interacting with arrestins.

The most interesting results have been achieved by introducing lipophilic linear aliphatic moieties at the C-terminus of N/OFQ(1–13)-NH_2_. Small differences were obtained with chains of different lengths, that is, 14, 16, and 18 carbon atoms (compounds **39–41**). On the contrary, the linear structure of the chain seems to be important because compound **50**, with a cyclooctane ring, displayed reduced potency and behaved as an unbiased agonist. Moreover, the linear moieties must be fully aliphatic because their substitution with amphipathic sequences (compounds **51** and **52**) reduced their potencies and totally eliminated the biased profile. The importance of the palmitoylation site has been investigated with compounds **53–56**. The shift of the palmitoyl group from position 14 to positions 11, 10, 12, and 13 caused a progressive reduction of the G protein bias. For the last two compounds, it should be noted that the Cys residue needed for palmitoylation substituted positively charged amino acids (Arg^12^ or Lys^13^) that are important for NOP binding, in agreement with previous results.^29−31^ Altogether these findings suggest that a linear aliphatic chain, particularly the palmitoyl group at the C-terminal of N/OFQ(1–13)-NH_2_, promoted a large (10–100 fold) G protein biased agonism because of increased NOP/G protein potency associated with a slight reduction of NOP/β-arrestin 2 potency. The mechanism by which C-terminal modifications of N/OFQ(1–13)-NH_2_ promote biased agonism toward G protein is at present unknown. To the best of our knowledge, there is a single example of naturally occurring palmitoylated ligands for G protein-coupled receptors (GPCRs). Wnts are a family of proteins that must be palmitoylated to exert their biological effects through the activation of Frizzled receptors. Recent crystallographic studies demonstrated that Wnts use the fatty acid as a hotspot residue to engage their receptor.^49^ However, it is unlikely that such a mechanism might be relevant for the interaction of compound **40** with the NOP receptor because NOP and Frizzled receptors are phylogenetically very far from each other.^50^ Moreover, it is noteworthy that compound **40** is structurally similar to pepducins, lipidated peptides of 10–20 amino acid residues with sequences derived from the receptor intracellular loops or C-terminus.^51^ Pepducins, most probably acting intracellularly,^52^ are able to modulate GPCR signaling, sometimes acting as biased agonists either toward G protein^53−55^ or arrestin.^56^ However, it is unlikely that compound **40** acts as a pepducin. In fact, this peptide is palmitoylated at the C-terminus while pepducins are lipidated at their N-terminus. More importantly, there is no homology between the peptide sequence of compound 40 and those of the intracellular loops or the C-terminus of the NOP receptor.^57^ The lipophilicity of the palmitoyl moiety may favor the insertion of the peptide into the plasma membrane or it may directly interact with the NOP transmembrane domains. In both cases, this mechanism may favor G protein- rather than arrestin-preferring NOP active conformations.

To gain insights into the mechanism of action of compounds **40** and **43**, their effects on NOP/G protein interactions were challenged with the NOP selective antagonist SB-612111^58,59^ and compared to those obtained with the standard agonist N/OFQ(-1–13)-NH_2_. The antagonist did not produce any effect per se but elicited a rightward parallel shift of the concentration–response curve to N/OFQ(-1–13)-NH_2_ without modifying the agonist maximal effect; a p*A*_2_ value of 8.05 (CL_95%_ 7.74–8.37) was derived from these experiments. Similar results were obtained using compound **40** and **43** as NOP agonists; the p*A*_2_ values of SB-612111 calculated from these experiments were 7.95 (CL_95%_ 7.43–8.47) and 7.77 (CL_95%_ 7.32–8.21), respectively (see Figure S1 of the Supporting Information). Thus, SB-612111 competitively antagonized the effects of N/OFQ,^19,60^ N/OFQ(1–13)-NH_2_, and compounds **40** and **43**, showing similar p*A*_2_ values. This demonstrated that similar to N/OFQ and N/OFQ(1–13)-NH_2_, compounds **40** and **43** activate the NOP receptor by interacting with the orthosteric binding pocket that has been described at the atomic level in previous NOP/C-24^4^ and NOP/SB-612111^5^ crystal structure studies.

Finally, in order to investigate whether the effects of palmitoylation of the peptide C-terminus are specific for N/OFQ and the NOP receptor or might influence the pharmacology of other opioid systems, this chemical modification has been applied to dermorphin, deltorphin A, and Leu-enkephalin,^61,62^ and the peptides evaluated at the mu (Table 8) and delta (Table 9) opioid receptors. Dermorphin promoted mu receptor interactions with G protein and arrestin with similar potency while producing an incomplete concentration–response curve at the delta receptor. Opposite results were obtained with deltorphin A that promoted delta receptor interactions with G protein and arrestin with similar potency while eliciting stimulatory effects at the mu receptor only at micromolar concentrations. Leu-enkephalin produced similar stimulatory effects at mu and delta receptors, being slightly more potent on the latter. Palmitoylation of the C terminus of dermorphin (compound **57**) increased peptide potency both in mu/G protein and mu/β-arrestin 2 experiments. In addition, **57** was able to stimulate delta receptor interactions with both G protein and β-arrestin 2 although with lower potency compared to the mu receptor. Palmitoylation of the C terminus of deltorphin A (**58**) increased peptide potency in delta/G protein but not in delta/β-arrestin 2 studies, thus displaying a bias factor of 1.06. In addition, compound **58** was able to stimulate mu receptor interactions with both G protein and β-arrestin 2 although with lower potency compared to the delta/G protein experiments. Finally, when C-terminal palmitoylation was applied to Leu-enkephalin (compound **59**), it caused a large increase in receptor/G protein potency both at the mu and delta receptors. However, this was associated with a slight increase in mu/β-arrestin 2 potency and with a slight decrease in delta/β-arrestin 2 potency. Thus, compound **59** behaved as a G protein biased delta agonist with a bias factor of 1.70.

Results obtained with reference opioid peptides confirmed previous findings:^23^ dermorphin and deltorphin A behaved as selective agonists for the mu and delta receptors, respectively, while Leu-enkephalin displayed similar potency at both receptors. C-terminus palmitoylated peptides (compounds **57–59**) consistently displayed higher potency than their parent peptides in receptor/G protein experiments both at the mu and delta receptors. For the mu receptor, a similar increase in potency was also measured in receptor/β-arrestin 2 experiments; as a result, palmitoylated opioid peptides behaved as potent unbiased mu receptor agonists. On the contrary, for the delta receptor, the increase of receptor/G protein potency of palmitoylated peptides was associated with a slight decrease in receptor/β-arrestin 2 potency; as a consequence, palmitoylated deltorphin A (compound **58**) and Leu-enkephalin (compound **59**) behaved as potent G protein biased delta receptor agonists (bias factors 1.06 and 1.70, respectively).

Collectively the results obtained by the insertion of a palmitoyl moiety at the C-terminal of N/OFQ or opioid peptides consistently produced a rather large (10–100-fold) increase of agonist potency in receptor/G protein experiments. On the contrary, the effect of this chemical modification in receptor/β-arrestin 2 experiments was variable depending on the receptor under evaluation: increase in potency for the mu opioid receptor and no changes or little decrease in potency for the delta and NOP receptors. These combined actions make palmitoylated peptides, at least for these specific examples, G protein biased agonists for NOP and delta receptor and unbiased agonists for the mu receptors.

## Conclusions

The present study was aimed at the identification of NOP receptor peptide biased agonists. Subtle chemical modifications in the N/OFQ(1–13)-NH_2_ sequence even if able to produce large changes in ligand potency and/or efficacy did not provide useful information for the design of NOP biased agonists. Biased agonism toward G protein can be obtained by N/OFQ(1–13)-NH_2_ C-terminal modifications with positively charged peptide sequences or linear aliphatic chains. The best results in terms of bias factors were obtained with the palmitoyl moiety. This chemical modification was also applied to mu and delta receptor peptide ligands; palmitoylated peptides consistently behaved as highly potent agonists for receptor/G protein interactions acting as G protein biased agonists for NOP and delta receptors and as unbiased agonists for the mu receptor. Further studies are needed to understand the mechanism by which C-terminal palmitoylation modulates the pharmacological profile of peptide agonists. Nevertheless, palmitoylation of biologically active peptides can be proposed as a chemical probe for generating highly potent agonists and in some cases G protein biased agonists. The availability of biased ligands together with solid knowledge of cell types and relative signaling pathways responsible for pathologies^63^ are equally important to reduce translational gaps and eventually make functional selectivity a successful strategy in drug discovery.^28^

## Experimental Section

### Materials and Methods

All chemicals, the resins for peptide synthesis, and Fmoc-protected amino acids were purchased from Bachem and Sigma-Aldrich, they were enantiopure and used as received. Peptides were synthesized according to published methods^64^ using Fmoc/*t*-butyl chemistry with a Syro XP multiple peptide synthesizer (MultiSynTech GmbH, Witten Germany). Peptides were synthesized at a 0.11 mM scale on a Rink amide MBHA resin [4-(2′,4′-dimethoxyphenyl-Fmoc-aminomethyl)-phenoxyacetamido-norleucyl-MBHA resin; loading 0.55 mmol/g] as a solid support. Fmoc-amino acids (4-fold excess) were sequentially coupled to the growing peptide chain using DIPCDI/HOBt (*N*,*N*′-diisopropylcarbodiimide/1-hydroxybenzotriazole) (4-fold excess) as an activating mixture for 1 h at room temperature. Cycles of deprotection of Fmoc (40% piperidine/*N*,*N*-dimethylformamide) and coupling with the subsequent amino acids were repeated until the desired peptide-bound resin was obtained. Peptides were cleaved from the resin using the standard cleavage cocktail (95% TFA, 2.5% H_2_O, 2.5% triethylsilane) at room temperature for 3 h. After this time, peptides were treated with ice-cold diethyl ether and the precipitate was isolated by centrifugation. Analytical high-performance liquid chromatography (HPLC) analyses were performed on a Beckman 116 liquid chromatograph equipped with a Beckman 166 diode array detector. Analytical purity of the peptides was assessed using a XBridge C18 column (4.6 × 150 mm, 5 μm particle size) at a flow rate of 0.7 mL/min with a linear gradient from 100% of solvent A (H_2_O + 0.1% TFA) to 100% of solvent B (CH_3_CN + 0.1% TFA) over 25 min. All final compounds showed ≥95% purity when monitored at 220 nm, and their molecular weights were confirmed by a mass spectrometer ESI Micromass ZQ. High-resolution mass spectrometry (HRMS) analysis of final compounds (see the Supporting Information) was performed with an ESI-Q-TOF Nano HPLC-CHIP Cube Agilent 6520 instrument or with a Q Exactive Hybrid Quadrupole-Orbitrap (Thermo Fisher Scientific) spectrometer equipped with HESI-II (ESI). For the HRMS spectra, the samples were analyzed by dissolving them in a mixture H_2_O/CH_3_OH 3:7 with 0.1% of formic acid (see the Supporting Information). NMR spectroscopy was performed using a Varian 400 MHz instrument (s: singlet, d: doublet, dd: double doublet, t: triplet, and m: multiplet), and all experiments were performed in CDCl_3_. Crude peptides were purified on a reverse-phase Waters Prep 600 HPLC system equipped with a Jupiter column C18 (250 × 30 mm, 300 Å, 15 μm spherical particle size) eluted with solvent A (H_2_O + 0.1% TFA) and B (40% H_2_O in CH_3_CN + 0.1% TFA) at a flow rate of 20 mL/min. Gradient was programmed time by time taking into account the analytical HPLC profile of the crude peptide. For the elution of lipidated compounds, solvent B has been replaced with CH_3_CN + 0.1% TFA. Stock solutions of the tested compounds were made in bidistilled water (1 mM) and stored at −20 °C. All cell culture media and supplements were purchased from Invitrogen (Paisley, UK) or EuroClone (Milano, Italy). Native coelenterazine (CLZN, 5 mM, EtOH) was purchased from Synchem UG & Co. KG (Altenburg, Germany). SB-612111 was purchased from Tocris Bioscience (Brisol, UK). Stock solution of SB-612111 was made in dimethyl sulfoxide (10 mM) and stored at −20 °C.

### Chemistry

#### Synthesis of Compounds **62a–c**

To a stirring solution of **60a–c** (1.0 mmol) in dimethylformamide (DMF) (2 mL) at 0 °C, hexafluorophosphate azabenzotriazole tetramethyl uronium (HATU) (1.2 equiv) and *N*,*N*-diisopropylethylamine (DIPEA) (2.0 equiv) were added. After 10 min, compound **61** (2.0 equiv), previously dissolved in DMF (2 mL) and DIPEA (2.0 equiv), was added dropwise. The reaction was stirred at room temperature for 16 h. After the solvent was evaporated under vacuum, the residue was dissolved in EtOAc (30 mL) and the organic layer was washed with 1 M HCl (30 mL), 5% NaHCO_3_ (25 mL), and brine (10 mL). The organic phase was dried over anhydrous Na_2_SO_4_ and concentrated to dryness. The crude residue was purified *via* column chromatography using a mixture of EtOAc and petroleum ether as the eluent to give compounds **62a–c**.

##### *N*-(3-(2,5-Dioxo-2,5-dihydro-1*H*-pyrrol-1-yl)propyl)tetradecanamide (**62a**)

White amorphous solid (yield 15%). ^1^H NMR (400 MHz, CDCl_3_): δ 6.72 (s, 2H), 6.06 (m, 1H), 3.61 (t, *J* = 6.4 Hz, 2H), 3.21 (t, *J* = 7.4 Hz, 2H), 2.23 (m, 2H), 1.80 (m, 2H), 1.66 (m, 2H), 1.27 (m, 20H), 0.89 (t, *J* = 6.3 Hz, 3H). ^13^C NMR (CDCl_3_): δ 173.41, 171.19, 134.33, 94.74, 36.99, 35.99, 34.88, 32.01, 29.74, 29.59, 29.49, 28.40, 25.87, 22.78, 14.23. MS (ESI) *m*/*z*: calcd for C_21_H_36_N_2_O_3_ [M + H]^+^, 365.53; found, 365.32.

##### *N*-(3-(2,5-Dioxo-2,5-dihydro-1*H*-pyrrol-1-yl)propyl)palmitamide (**62b**)

White amorphous solid (yield 37%). ^1^H NMR (400 MHz, CDCl_3_): δ 6.74 (s, 2H), 6.00 (m, 1H), 3.60 (t, *J* = 6.7 Hz, 2H), 3.21 (m, 2H), 2.23 (t, *J* = 7.5 Hz, 2H), 1.78 (m, 4H), 1.66 (m, 2H), 1.29 (m, 22H), 0.89 (t, *J* = 6.8 Hz, 3H). ^13^C NMR (CDCl_3_): δ 173.41, 171.17, 134.30, 110.26, 37.01, 35.90, 34.87, 32.01, 29.61, 28.40, 25.85, 22.78, 14.23. MS (ESI) *m*/*z*: calcd for C_23_H_40_N_2_O_3_ [M + H]^+^, 393.58; found, 393.41.

##### *N*-(3-(2,5-Dioxo-2,5-dihydro-1*H*-pyrrol-1-yl)propyl)stearamide (**62c**)

White amorphous solid (yield 11%). ^1^H NMR (400 MHz, CDCl_3_): δ 6.75 (s, 2H), 6.10 (m, 1H), 3.60 (t, *J* = 6.3 Hz, 2H), 3.22 (m, 2H), 2.24 (t, *J* = 7.8 Hz, 2H), 1.80 (m, 4H), 1.64 (m, 2H), 1.29 (m, 26H), 0.89 (t, *J* = 6.4 Hz, 3H). ^13^C NMR (CDCl_3_): δ 173.54, 171.19, 134.32, 36.97, 35.95, 34.88, 32.01, 29.62, 28.40, 25.86, 22.79, 14.23. MS (ESI) *m*/*z*: calcd for C_25_H_44_N_2_O_3_ [M + H]^+^, 421.64; found, 421.50.

### General Procedure for the Synthesis of **65d–m**

A solution of 4-(2,5-dioxo-2,5-dihydro-1*H*-pyrrol-1-yl)butanoic acid (**64**, 4.0 equiv),^65^ EDAC (4.0 equiv), and HOBt (4.0 equiv) in DMF (3 mL) was added to the peptide-resin **63d–m** (1.0 equiv). The mixture was left stirring for 3 h, and the completion of the reaction was monitored *via* electrospray ionization–mass spectrometry (ESI-MS) after micro-cleavage of the product from the resin using the standard cleavage cocktail. Compounds were cleaved from the resin using the standard cleavage cocktail (95% TFA, 2.5% H_2_O, 2.5% triethylsilane) at room temperature for 3 h. After this time, the mixtures were treated with ice-cold diethyl ether, and the precipitate was isolated through centrifugation. The crude mixture was purified by preparative HPLC to give the desired derivatives **65d–m** (15–48% yields). All products were characterized by ESI-MS (see the Supporting Information).

### Synthesis of *N*-Cyclooctyl-4-(2,5-dioxo-2,5-dihydro-1*H*-pyrrol-1-yl)butanamide (**67**)

To a solution of the maleimidocarboxylic NHS-ester **66** (1.0 mmol)^66^ in CH_3_CN, cyclooctylamine (1.0 equiv) and Et_3_N (1.0 equiv) were added at room temperature and the reaction was left stirring for 30 min. After the removal of the solvent, the crude residue was dissolved in EtOAc (50 mL) and the organic phase was washed with 1 M HCl (20 mL) and brine (20 mL). The organic layer was dried over anhydrous Na_2_SO_4_, filtered, and concentrated under vacuum. **67** was obtained after purification *via* column chromatography with a 2:1 mixture of EtOAc and petroleum ether. White amorphous solid (67% yield). ^1^H NMR (400 MHz, CDCl_3_): δ 6.72 (s, 2H), 5.85 (m, 1H), 4.00 (m, 1H), 3.58 (t, *J* = 7.6 Hz, 2H), 2.12 (t, *J* = 7.0 Hz, 2H), 1.94 (m, 2H), 1.84 (m, 2H), 1.59 (m, 12H). ^13^C NMR (CDCl_3_): δ 170.43, 169.74, 133.55, 48.83, 36.50, 33.27, 31.52, 26.64, 24.77, 24.46, 23.01. MS (ESI) *m*/*z*: calcd for C_16_H_24_N_2_O_3_ [M + H]^+^, 293.38; found, 293.49. *t*_R_ = 19.00.

### General Approach for the Synthesis of the N/OFQ Peptide Derivatives **39–59***via* Thiol-Michael Coupling

To a solution of the maleimido compound (**62a–c**, **65d–m** and **67**, 10 μmol) and the appropriate peptide sequence functionalized with a cysteine residue (1.05 equiv) in CH_3_CN/H_2_O (2:1, 300 μL), 10 μL of a 5% aqueous solution of NaHCO_3_ were added. After 5 min, the ESI-MS spectrum showed the completion of the reaction and the crude mixture was purified by preparative HPLC to give the desired derivatives **39–59**. All products were characterized by ESI-MS, and their purity degree (>95% at 220 nm) was evaluated by analytical HPLC (see the Supporting Information).

### Pharmacology

#### BRET Assay

For this study, human embryonic kidney (HEK293) cells, permanently co-expressing the different pairs of fusion proteins NOP-RLuc/Gβ1-RGFP and NOP-RLuc/β-arrestin 2-RGFP, and SH-SY5Y cells stably, co-expressing the different pairs of fusion proteins mu-RLuc/Gβ1-RGFP and mu-RLuc/β-arrestin 2-RGFP or delta-RLuc/Gβ1-RGFP and delta-RLuc/β-arrestin 2-RGFP, were used. Cells were prepared and cultured, as described previously.^19,23^ Cells were grown in Dulbecco’s modified Eagle’s medium (DMEM)/HAMS F12 (1:1) supplemented with 10% fetal bovine serum, penicillin G (100 units/mL), streptomycin (100 μg mL^–1^), l-glutamine (2 mM), fungizone (1 μg mL^–1^), geneticin (G418; 200 μg mL^–1^), and hygromycin B (100 μg mL^–1^) in a humidified atmosphere of 5% CO_2_ at 37 °C. For G protein experiments, enriched plasma membrane aliquots from transfected cells were prepared by differential centrifugation; cells were detached with phosphate buffered saline (PBS)/EDTA solution (1 mM, pH 7.4 NaOH), then, after 5 min 500*g* centrifugation, Dounce-homogenized (30 strokes) in cold homogenization buffer (tris(hydroxymethyl)aminomethane 5 mM, EGTA 1 mM, DTT 1 mM, pH 7.4 HCl) in the presence of sucrose (0.32 M). Three following centrifugations were performed at 10 min 1000*g* (4 °C), and the supernatants were kept. Two 20 min 24,000*g* (4 °C) subsequent centrifugations (the second in the absence of sucrose) were performed for separating enriched membranes that, after discarding the supernatant, were kept in ultrapure water at −80 °C.^67^ The protein concentration in membrane preparations was determined using the QPRO-BCA kit (Cyanagen Srl, Bologna, IT) and the Ensight (PerkinElmer, Waltham, USA) in the spectrophotometer mode.

Luminescence in membranes was recorded in 96-well untreated white opaque microplates, while in whole cells, it was recorded in 96-well sterile poly-d-lysine-coated white opaque microplates (PerkinElmer, Waltham, MA, USA) using the luminometer Victor 2030 (PerkinElmer, Waltham, MA, USA). For the determination of receptor/G protein interactions, membranes (3 μg of protein) prepared from cells co-expressing NOP (or mu or delta)/RLuc and Gβ1/RGFP were added to wells in DPBS. For the determination of receptor/β-arrestin 2 interactions, cells co-expressing NOP (or mu or delta)/RLuc and β-arrestin 2/RGFP were plated 24 h before the experiment in poly-d-lysine-treated plates (100,000 cells/well). The cells were prepared for the experiment substituting the medium with PBS with MgCl_2_ (0.5 mM) and CaCl_2_ (0.9 mM). Coelenterazine at a final concentration of 5 μM was injected 15 min prior to reading the cell plate. Different concentrations of ligands in 20 μL of PBS–BSA 0.01% were added and incubated 5 min before reading luminescence. In antagonism experiments, antagonists (**27**, 10 μM; **28**, 10 μM; **30**, 1 μM; **31**, 1 μM; SB-612111, 10 nM) were incubated 15 min before adding N/OFQ(1–13)-NH_2_. The BRET ratio was measured 15 min after agonist injection. All experiments were performed at room temperature.

#### Data Analysis and Terminology

The pharmacological terminology adopted in this paper is consistent with IUPHAR recommendations.^68^ All data are expressed as the mean ± sem of five independent experiments made in duplicate. For potency values, 95% confidence limits (CL_95%_) were indicated. Ligand efficacy was expressed as intrinsic activity (α), calculated as the ratio between the *E*_max_ of the ligand and that of the standard agonist. Count per second (CPS) measured for the RGFP and RLuc light emitted using 510(10) and 460(25) filters (PerkinElmer, Waltham, MA), respectively, was used to calculate the BRET ratio. All data are expressed as the stimulated BRET ratio, that is, the ratio between CPS from RGFP and RLuc in the presence of ligands minus the BRET ratio in vehicle-treated wells. Maximal agonist effects were expressed as a fraction of the standard [N/OFQ(1–13)-NH_2_ for NOP, deltorphin A for delta, dermorphin for mu] maximal effects which were determined in every assay plate. Agonist potencies are given as pEC_50_, that is, the negative logarithm to base 10 of the molar concentration of an agonist that produces 50% of the maximal effect of that agonist. Concentration–response curves to agonists were fitted to the classical four-parameter logistic nonlinear regression model
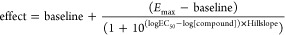


EC_50_ is the concentration of agonist producing a 50% maximal response. Curves fitting were performed using PRISM 6.0 (GraphPad Software In., San Diego, USA). As listed in Tables 1–3 and 5–9, ligand potency is expressed as a concentration ratio (CR), calculated as the ratio between the EC_50_ of the ligand and that of the standard agonist.

Antagonist potencies were derived from the Gaddum–Schild equation
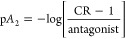
assuming a slope value equal to unity, where CR indicates the ratio between agonist potency in the presence and absence of antagonist.^69^

Bias factors were calculated using N/OFQ(1–13)-NH_2_ for NOP, dermorphin for mu, and deltorphin A for delta as standard unbiased ligand.

The concentration–response curves of each compound were fitted to the Black–Leff operational model, described by Nagi and Pineyro^70^
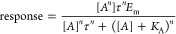
where [*A*] is the agonist concentration, the maximal response of the system is given by *E*_m_, *n* is a fitting parameter for the slope, the affinity of the agonist is represented by the equilibrium dissociation constant of the agonist–receptor complex (*K*_A_), and the efficacy of the agonist is defined by τ. τ and *K*_A_ are descriptive parameters of intrinsic efficacy and binding affinity and may be directly obtained by fitting the experimental data to the operational equation and can be expressed as “transduction coefficients” log(τ/*K*_A_). The relative efficiency of agonists producing activation of any pathways can thus be quantified with a “normalized” transduction coefficient, namely Δlog(τ/*K*_A_). Finally, the bias factors were calculated as difference between Δlog(τ/*K*_A_) values for a given agonist between the pathways (G protein and β-arrestin 2)
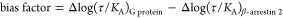


Bias factors are expressed as the mean of at least five independent experiments, and CL_95%_ are indicated. Biased factor is considered to be statistically different from 0 when 0 is not included in CL_95%_. α values have been statistically analyzed with one-way analysis of variance (ANOVA) followed by the Dunnett post hoc test for multiple comparisons; *P* values less than 0.05 were considered to be statistically significant.

## Supporting Information Available

ESI mass spectra of compounds **62a–c**, **65d–m**, **67**, and **39–59**; HPLC chromatograms of compounds **67** and **39–59**; HRMS profiles of final compounds; concentration–response curves to N/OFQ(1–13)-NH_2_, compounds **40** and **43** in the absence and presence of the NOP antagonist SB-612111; and chemical structures of unusual amino acids (PDF)
Molecular formula strings (CSV)

## Abbreviations

BRET: bioluminescence resonance energy transfer
BSA: bovine serum albumin
DIPCDI: *N*,*N*′-diisopropylcarbodiimide
DIPEA: *N*,*N*-diisopropylethylamine
CL_95%_: 95% confidence limits
CLZN: coelenterazine
CPS: count per second
CR: concentration ratio
CRC: concentration–response curve
DMEM: Dulbecco’s modified Eagle’s medium
DPBS: phosphate buffered saline
DTT: dithiothreitol
EDAC: 1-ethyl-3-(3-dimethylaminopropyl)carbodiimide
EGTA: ethylene glycol-bis(β-aminoethyl ether)-*N*,*N*,*N*′,*N*′-tetra-acetic acid
GPCR: G protein-coupled receptor
HATU: hexafluorophosphate azabenzotriazole tetramethyl uronium
HOBt: 1-hydroxybenzotriazole
MBHA resin: 4-methylbenzhydrylamine resin
NHS: *N*-hydroxy succinimide
N/OFQ: nociceptin/orphanin FQ
NOP: N/OFQ peptide
PBS: phosphate buffered saline
PWT: peptide welding technology
RGFP: recombinant green fluorescent protein
RLuc: Renilla luciferase
sem: standard error of the mean
SPPS: solid phase peptide synthesis
TFA: trifluoroacetic acid
