# Supplementary material for: Biased Agonism at Nociceptin/Orphanin FQ Receptors: A Structure Activity Study on N/OFQ(1–13)-NH2
Source: J Med Chem. 2020 Sep 9;63(19):10782–95. doi: 10.1021/acs.jmedchem.9b02057 (PMC8011926; doi:10.1021/acs.jmedchem.9b02057)

# Biased agonism at nociceptin/orphanin FQ receptors: a structure activity study on N/OFQ(1-13)-$NH_2$

Salvatore Pacifico[1], Federica Ferrari[2], Valentina Albanese [1], Erika Marzola[1], Joaquim Azevedo Neto[2], Chiara Ruzza[2,3], Girolamo Calò[2], Delia Preti[1*] and Remo Guerrini[1,3]

*[1]Department of Chemical and Pharmaceutical Sciences, University of Ferrara, Via Luigi Borsari 46, 44121 Ferrara, Italy. [2]Department of Medical Sciences, Section of Pharmacology, University of Ferrara, Via Fossato di Mortara 17/19, 44121 Ferrara, Italy.[3] Technopole of Ferrara, LTTA Laboratory for Advanced Therapies, via Fossato di Mortara 70, 44121 Ferrara, Italy.*

## CONTENTS


| | Pag. |
|---|---|
| Figure S1: concentration-response curves to N/OFQ(1-13)-$NH_2$, compounds **40** and **43**, in absence and presence of the NOP antagonist SB-612111 | S2 |
| Table S1: Structures of unusual amino acids or of dipeptides modified at the peptide bond. | S3 |
| ESI mass spectra of compounds **62a-c** | S4-S5 |
| ESI mass spectra of compounds **65d-m** | S6-S10 |
| HPLC chromatogram and ESI mass spectrum of compound **67** | S11 |
| HPLC chromatograms, ESI mass spectra and HRMS profiles of compounds **39-59** | S12-S66 |

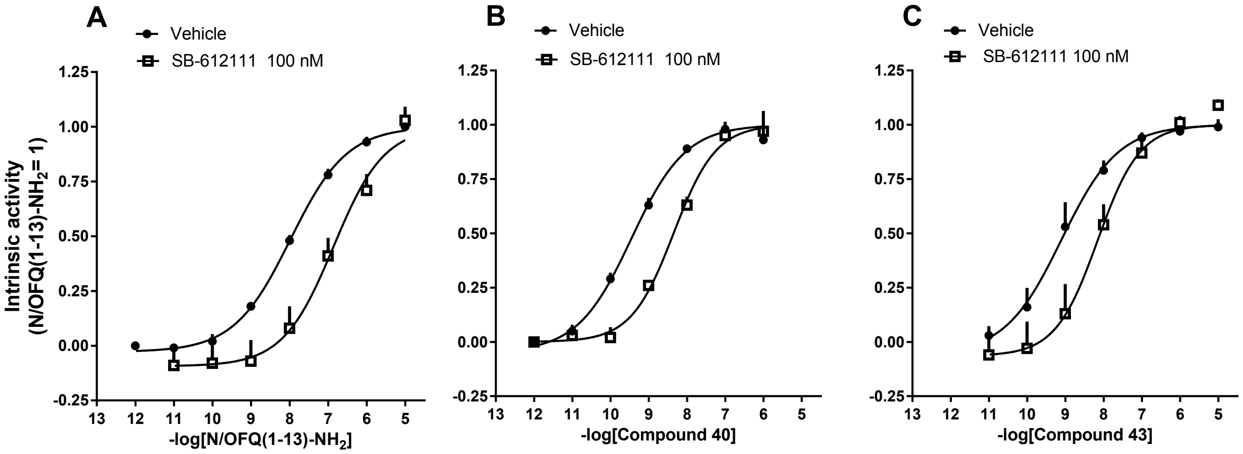


**Figure S1.** NOP/G-protein interaction in BRET experiments. Concentration-response curves to N/OFQ(1-13)-$NH_2$ (panel A), compound 40 (panel B) and compound 43 (panel C), in absence (vehicle) and presence of the NOP antagonist SB-612111 at 100 nM. Data are the mean ± S.E.M. of 4 separate experiments made in duplicate.

| Abbreviation | Chemical Structure |
|---|---|
| Cha | 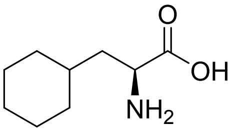 |
| Nphe | 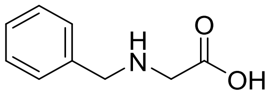 |
| (S)βMeNphe | 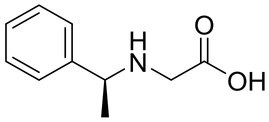 |
| (pF)Phe | 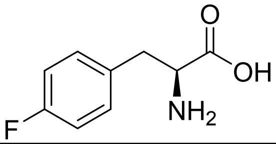 |
| Pheψ($CH_2$-NH)Gly | 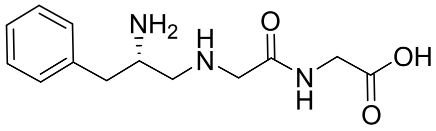 |
| Pheψ($CH_2$-S)Gly | 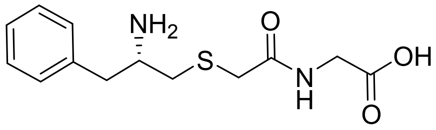 |
| Aib | 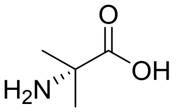 |
| $AC_3C$ | 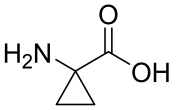 |
| $AC_5C$ | 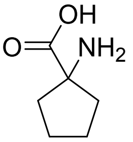 |

**Table S1.** Structures of unusual amino acids or of dipeptides modified at the peptide bond.

**N-(3-(2,5-dioxo-2,5-dihydro-1H-pyrrol-1-yl)propyl)tetradecanamide (62a)**

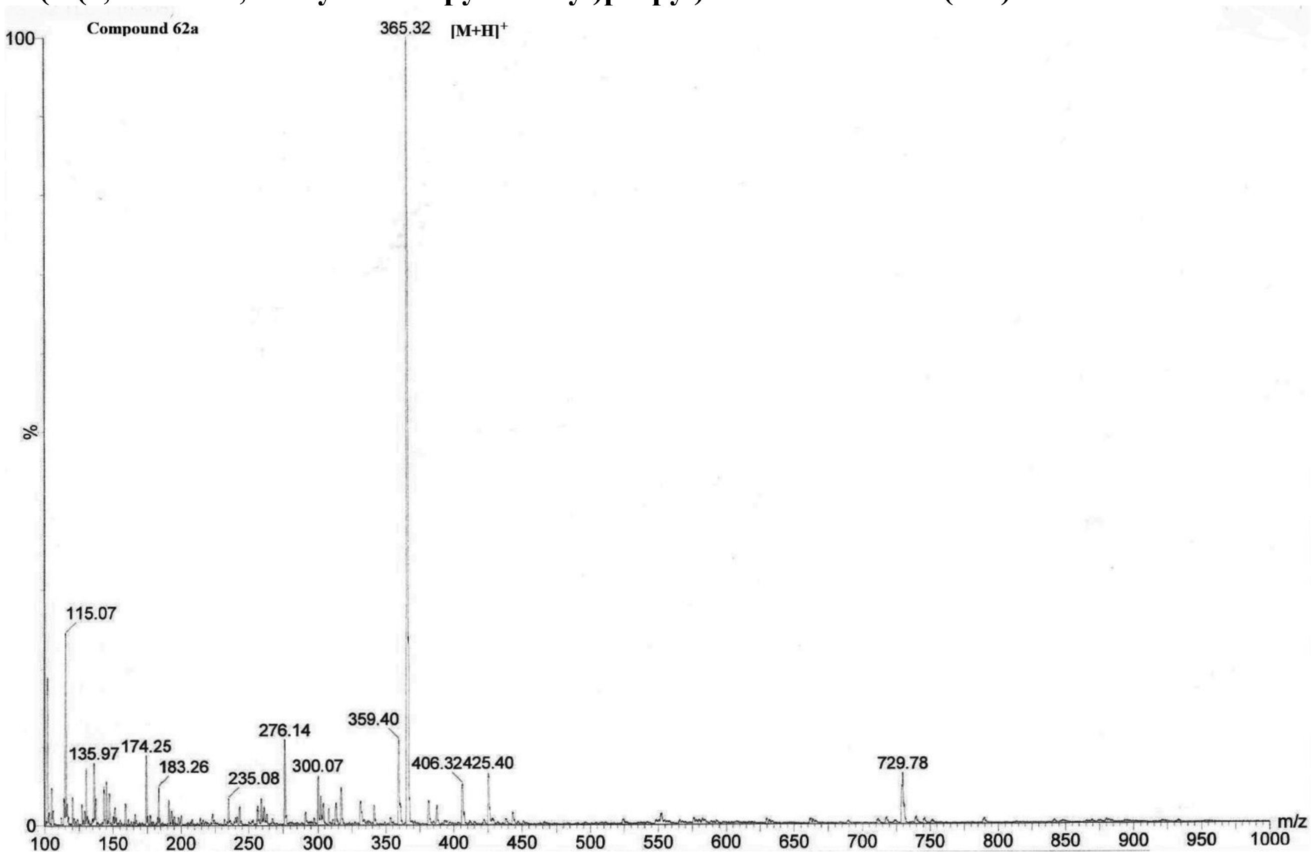


**N-(3-(2,5-dioxo-2,5-dihydro-1H-pyrrol-1-yl)propyl)palmitamide (62b)**

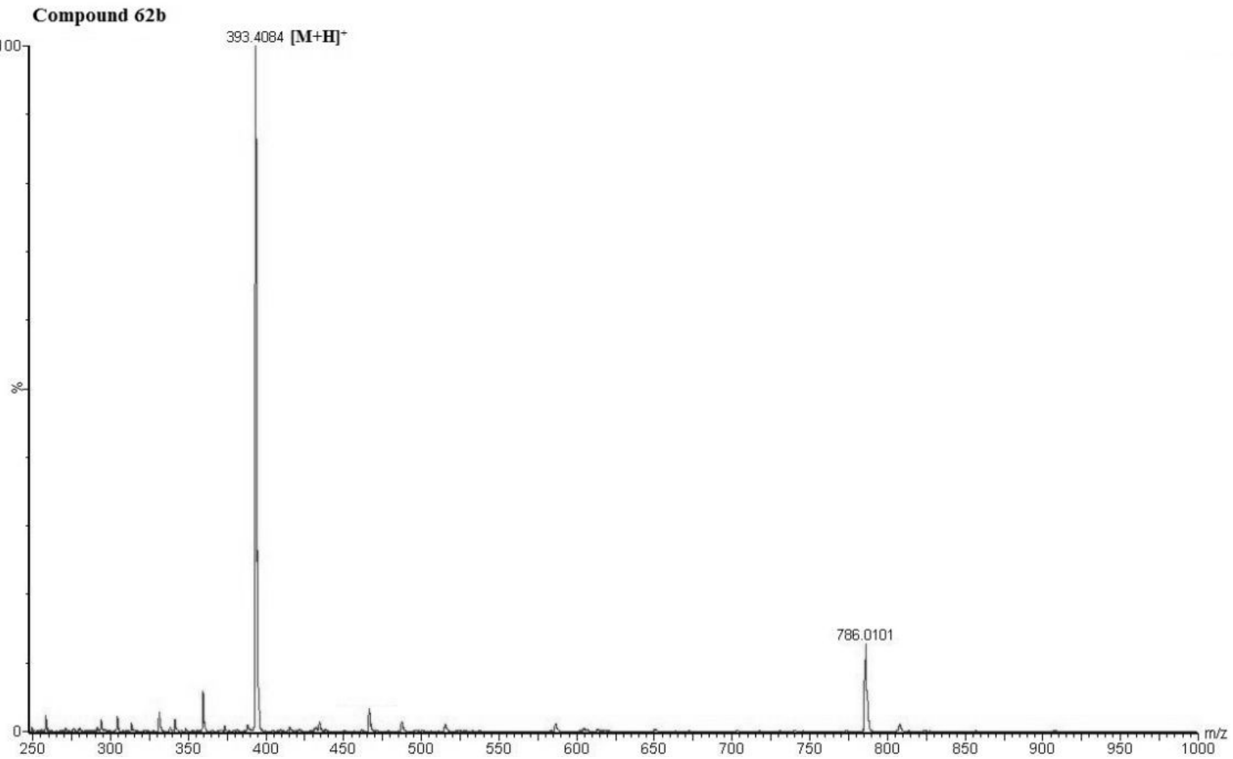

**N-(3-(2,5-dioxo-2,5-dihydro-1H-pyrrol-1-yl)propyl)stearamide (62c)**

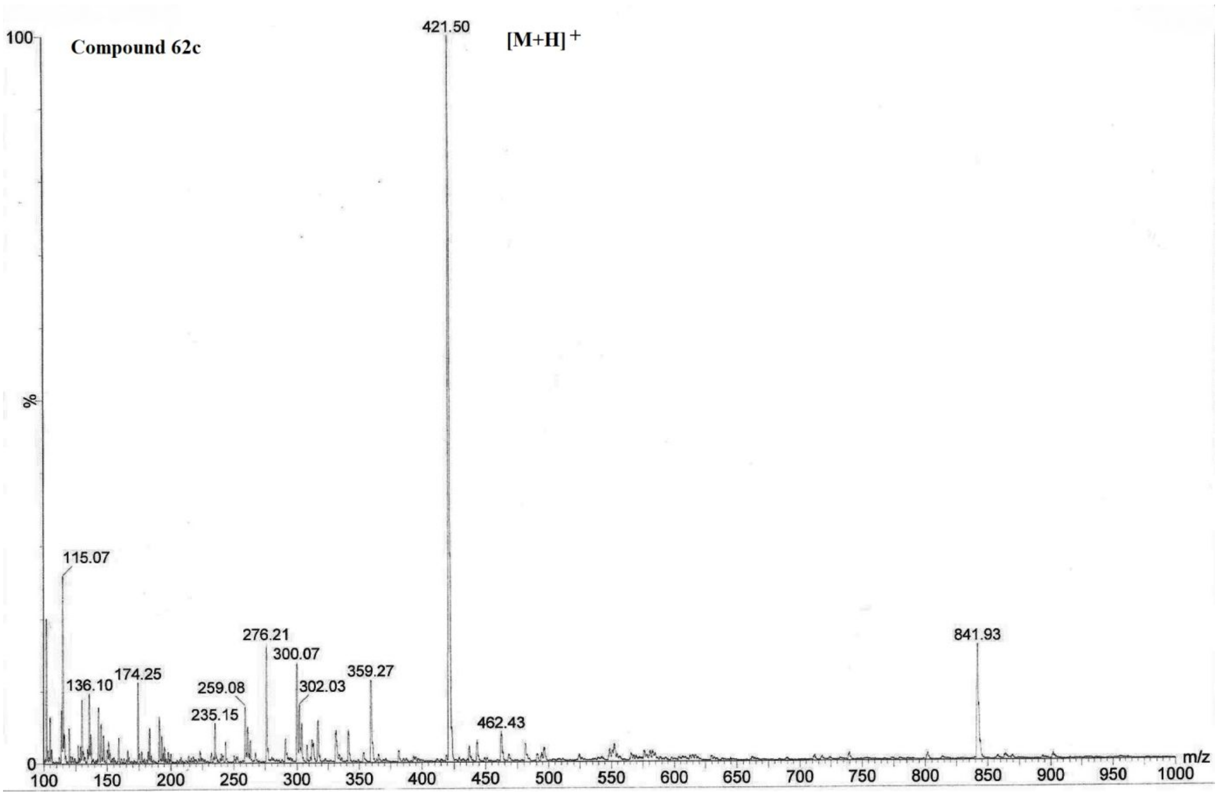

**Compound 65d.** MS (ESI): m/z calcd for $C_{26}H_{43}N_5O_{12}$ $[M+H]^+$ 618.65, found 618.74.

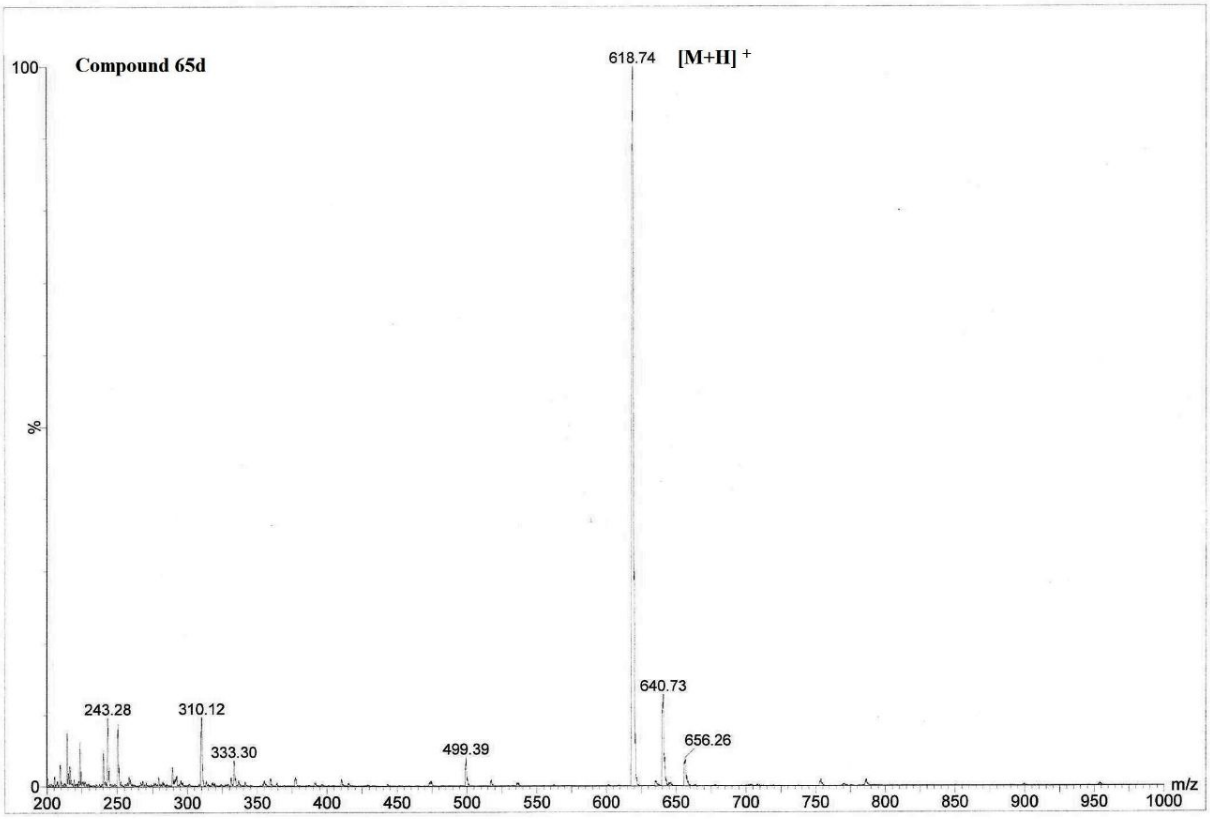


**Compound 65e.** MS (ESI): m/z calcd for $C_{62}H_{112}N_{20}O_{15}$ $[M+3H]^{3+}$ 460.23, found 460.00.

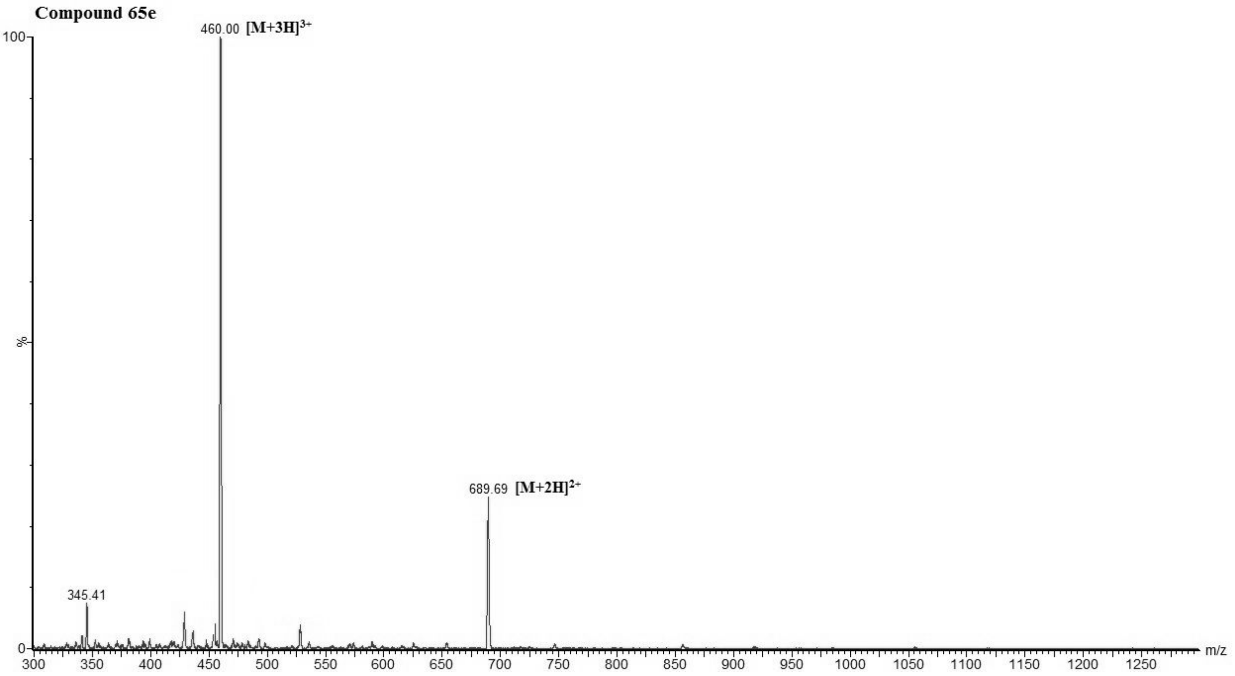

**Compound 65f.** MS (ESI): m/z calcd for $C_{56}H_{100}N_{20}O_{15}$ $[M+3H]^{3+}$ 432.18, found 432.15.

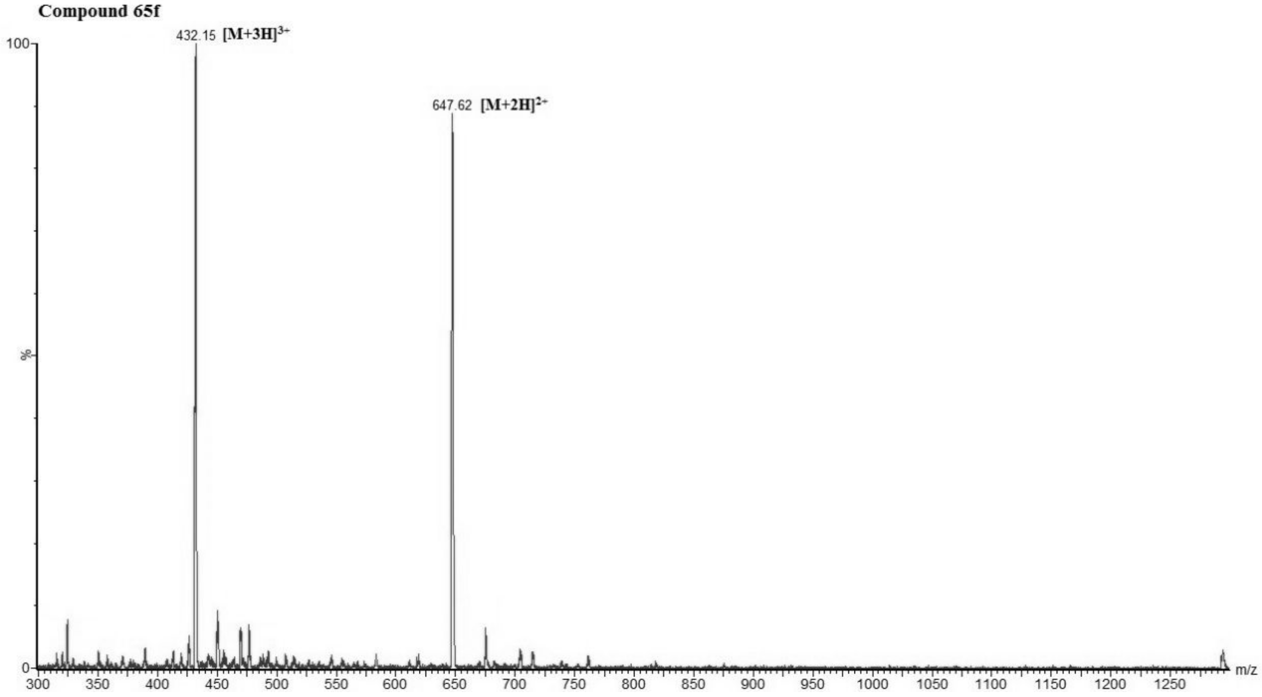


**Compound 65g.** MS (ESI): m/z calcd for $C_{50}H_{70}N_{14}O_{27}$ $[M+H]^{+}$ 1300.18, found 1299.84.

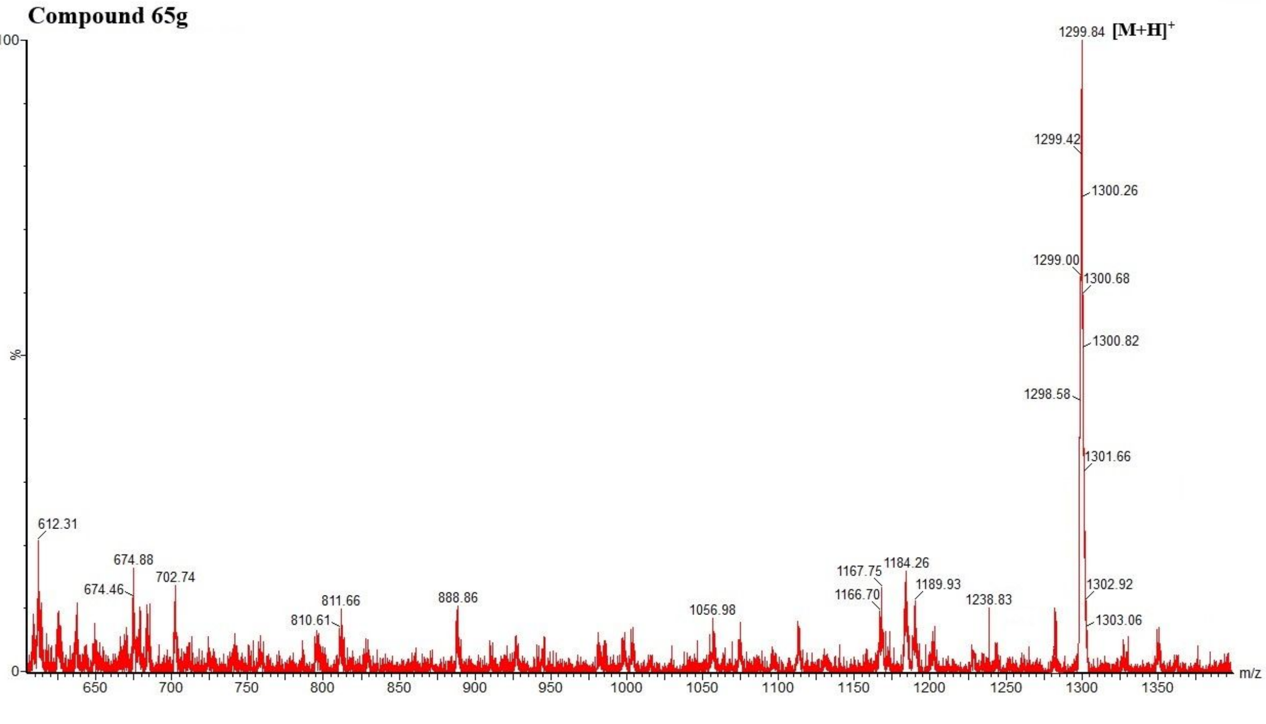

**Compound 65h.** MS (ESI): m/z calcd for $C_{44}H_{58}N_{14}O_{27}$ $[M+H]^{+}$ 1216.02, found 1215.86.

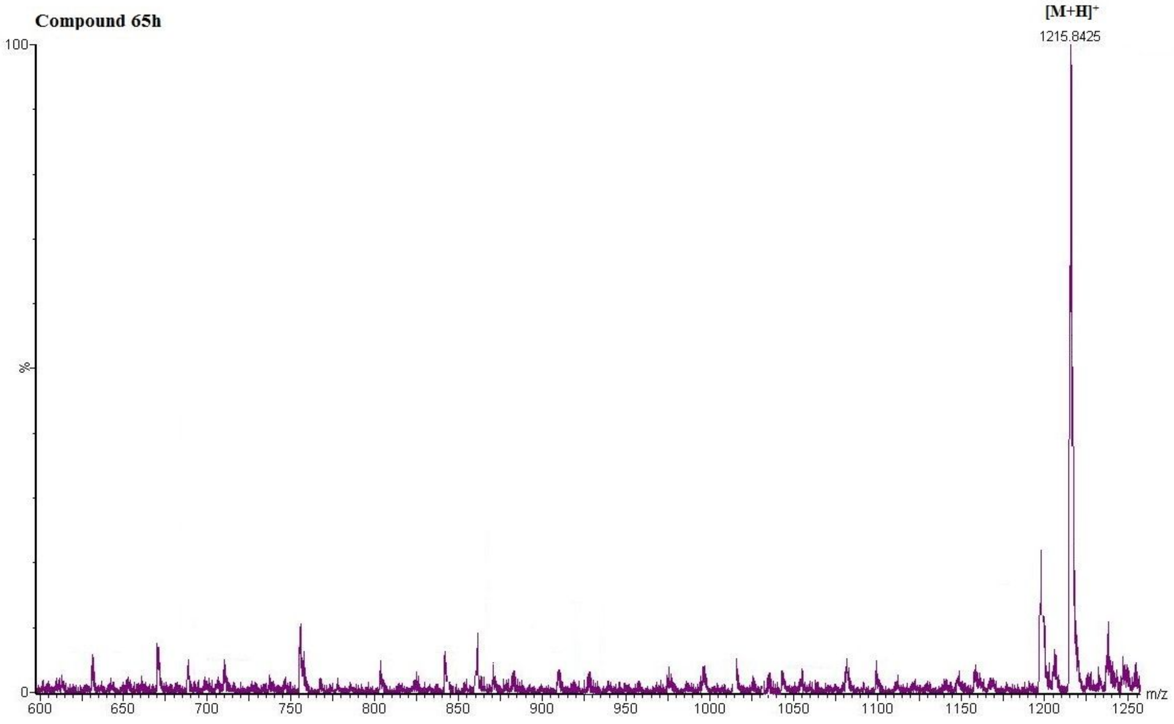


**Compound 65i.** MS (ESI): m/z calcd for $C_{62}H_{82}N_{26}O_{15}$ $[M+3H]^{3+}$ 478.17, found 478.50.

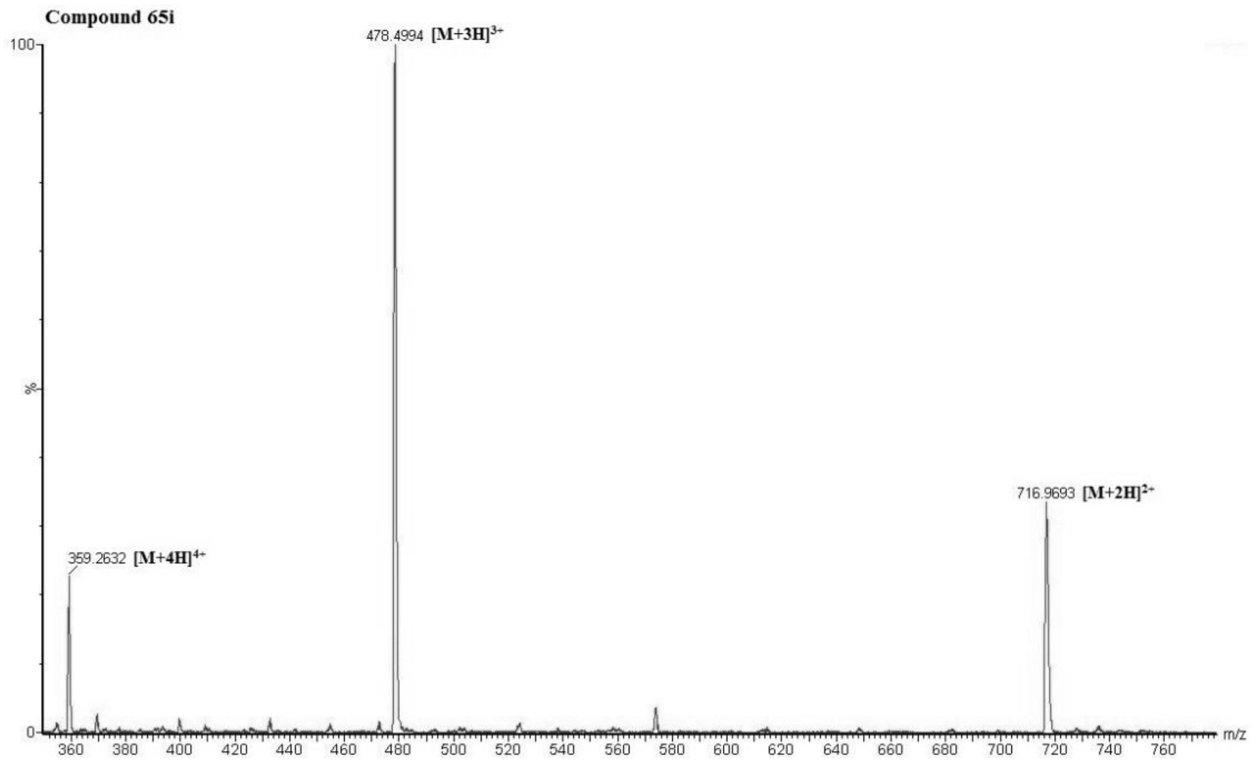

**Compound 65j.** MS (ESI): m/z calcd for $C_{56}H_{70}N_{26}O_{15}$ $[M+2H]^{2+}$ 450.11, found 450.46.

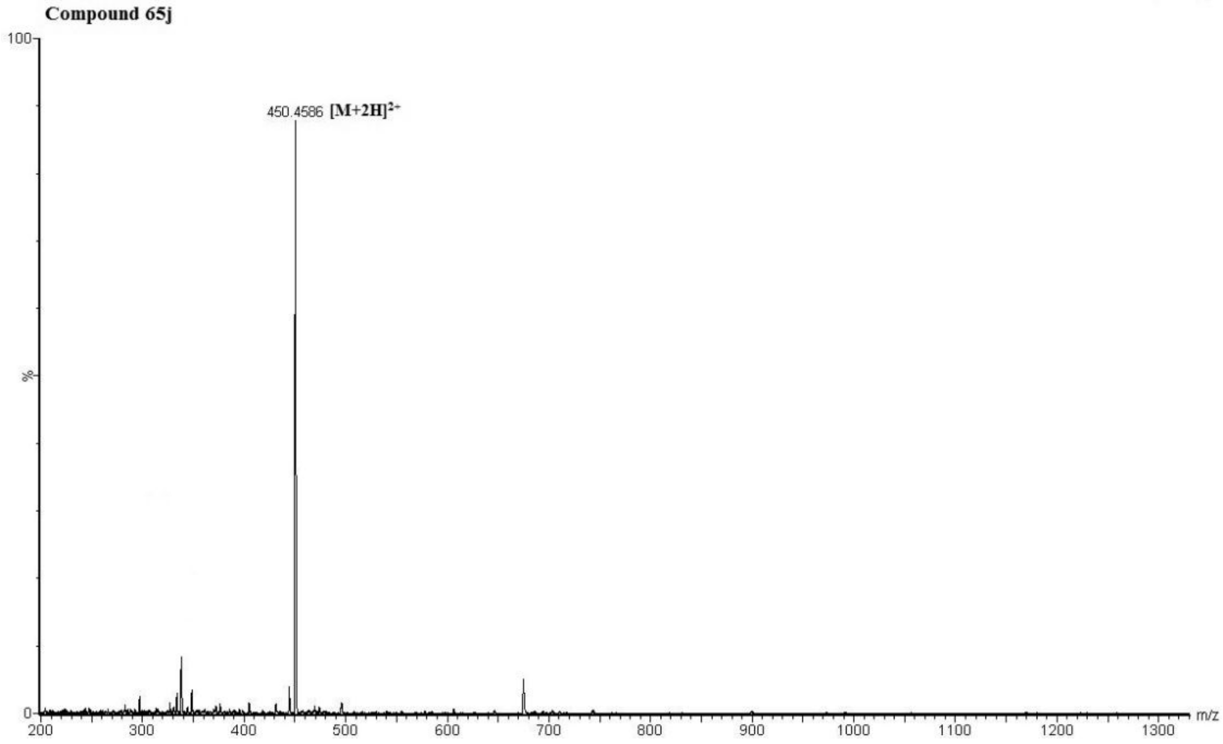


**Compound 65k.** MS (ESI): m/z calcd for $C_{44}H_{82}N_{14}O_9$ $[M+2H]^{2+}$ 476.62, found 476.67.

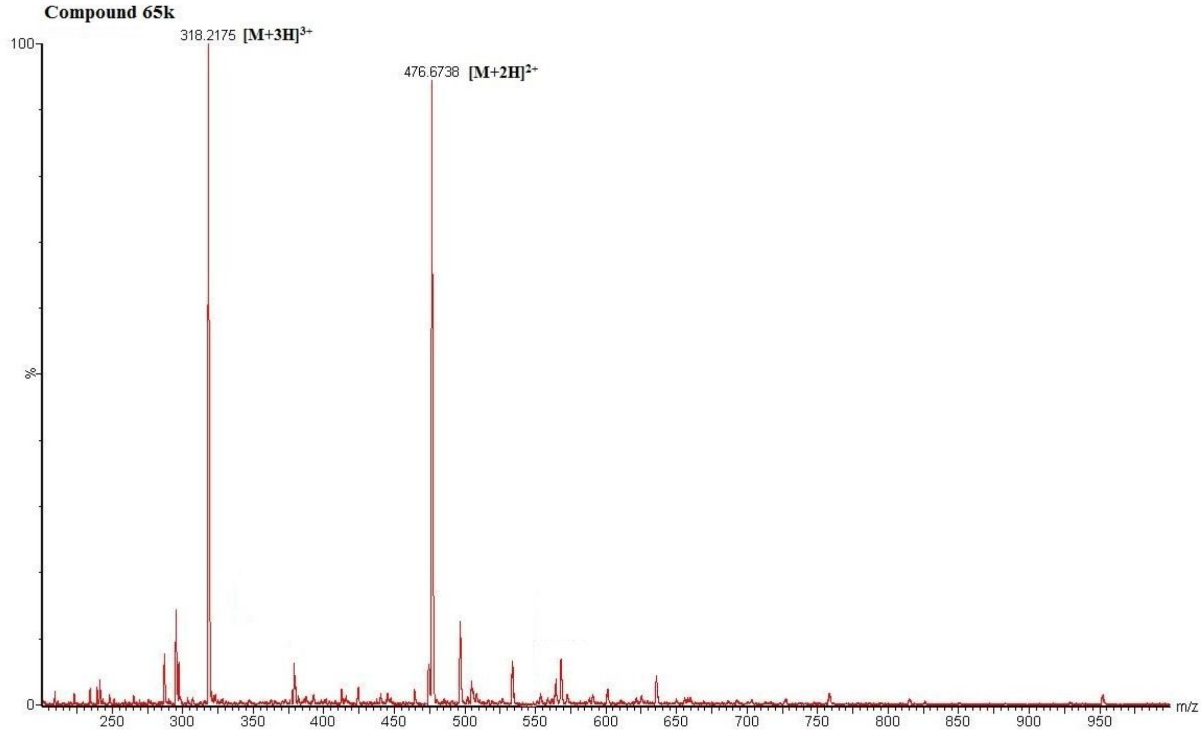

**Compound 65l.** MS (ESI): m/z calcd for $C_{28}H_{48}N_4O_5$ $[M+H]^+$ 549.76, found 549.60.

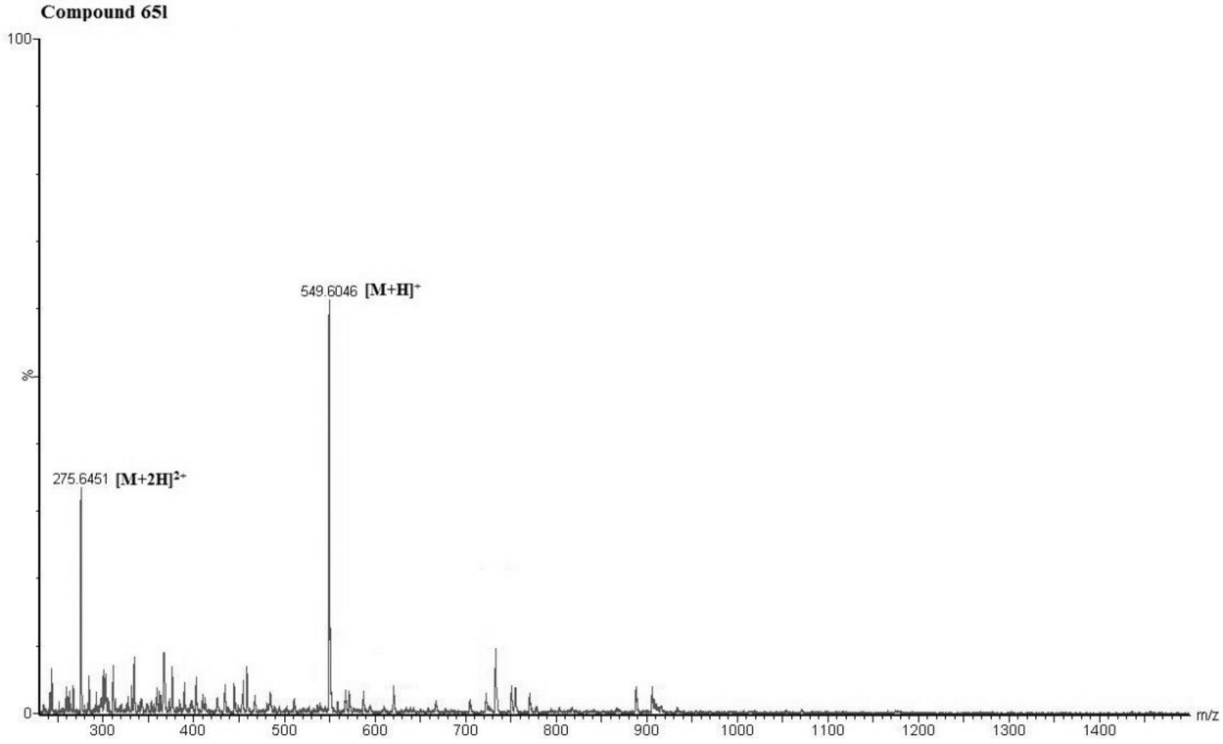


**Compound 65m.** MS (ESI): m/z calcd for $C_{38}H_{67}N_5O_6$ $[M+H]^+$ 733.06, found 733.00.

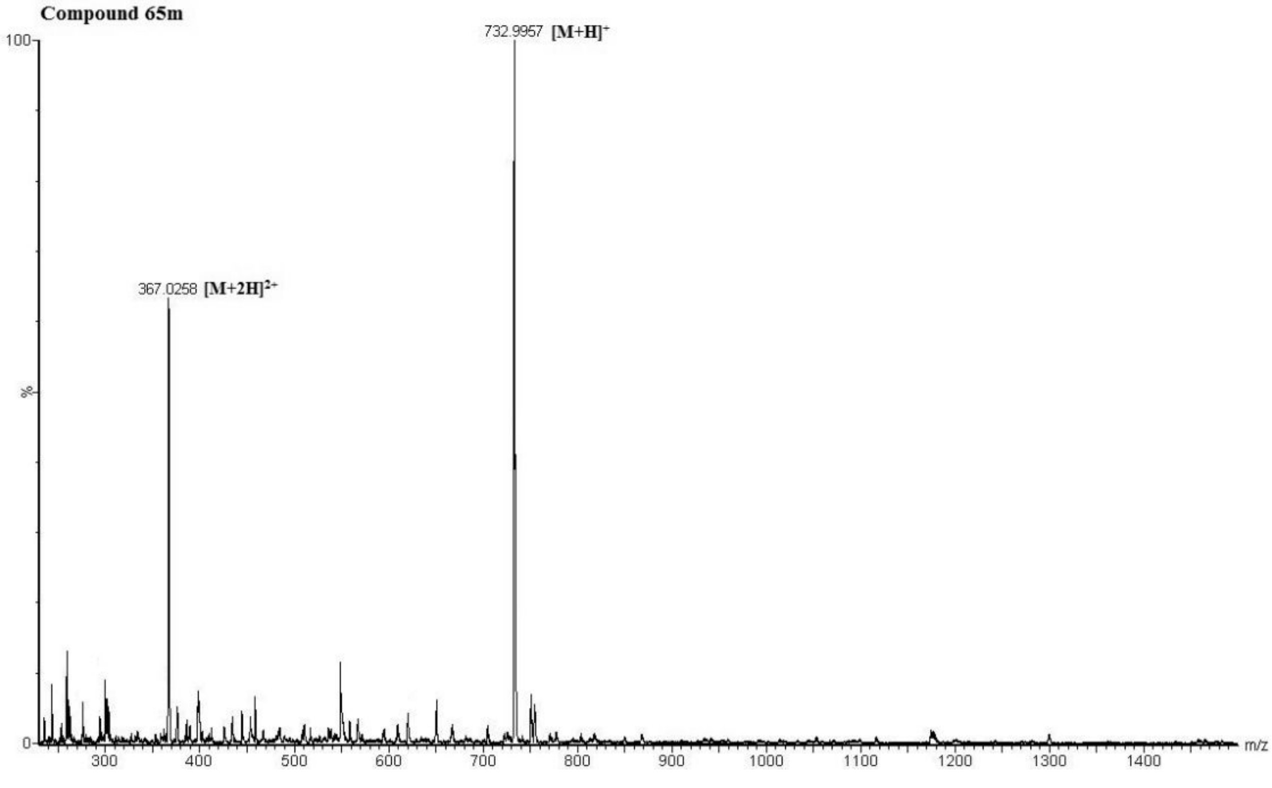

**N-cyclooctyl-4-(2,5-dioxo-2,5-dihydro-1H-pyrrol-1-yl)butanamide (67)**

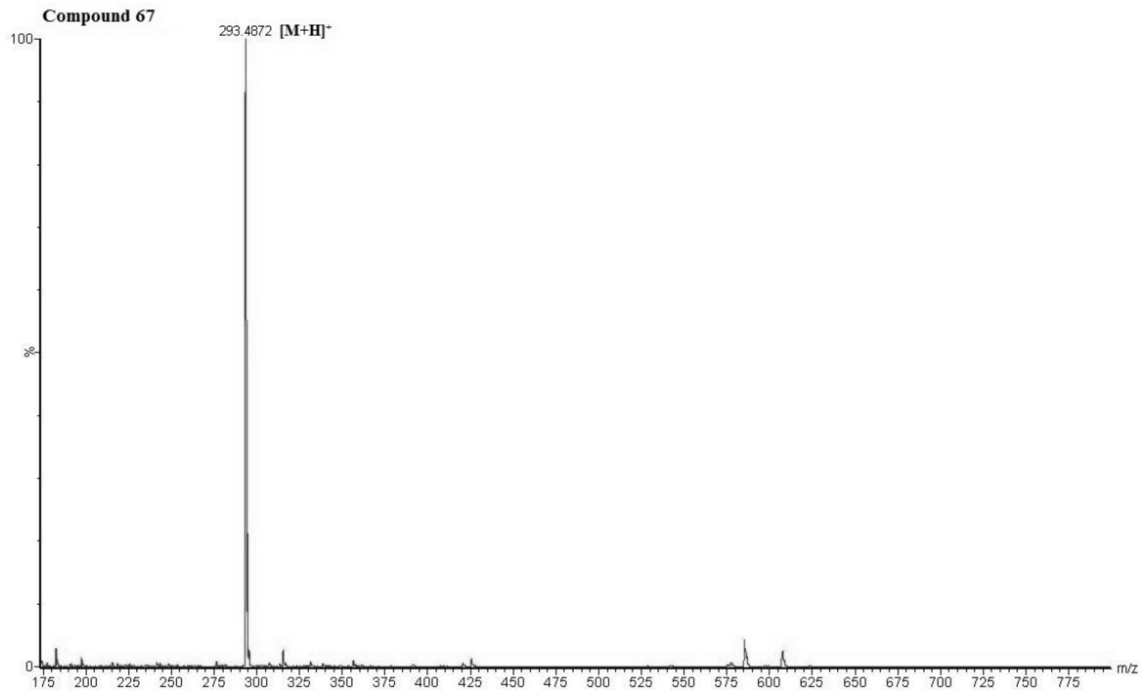


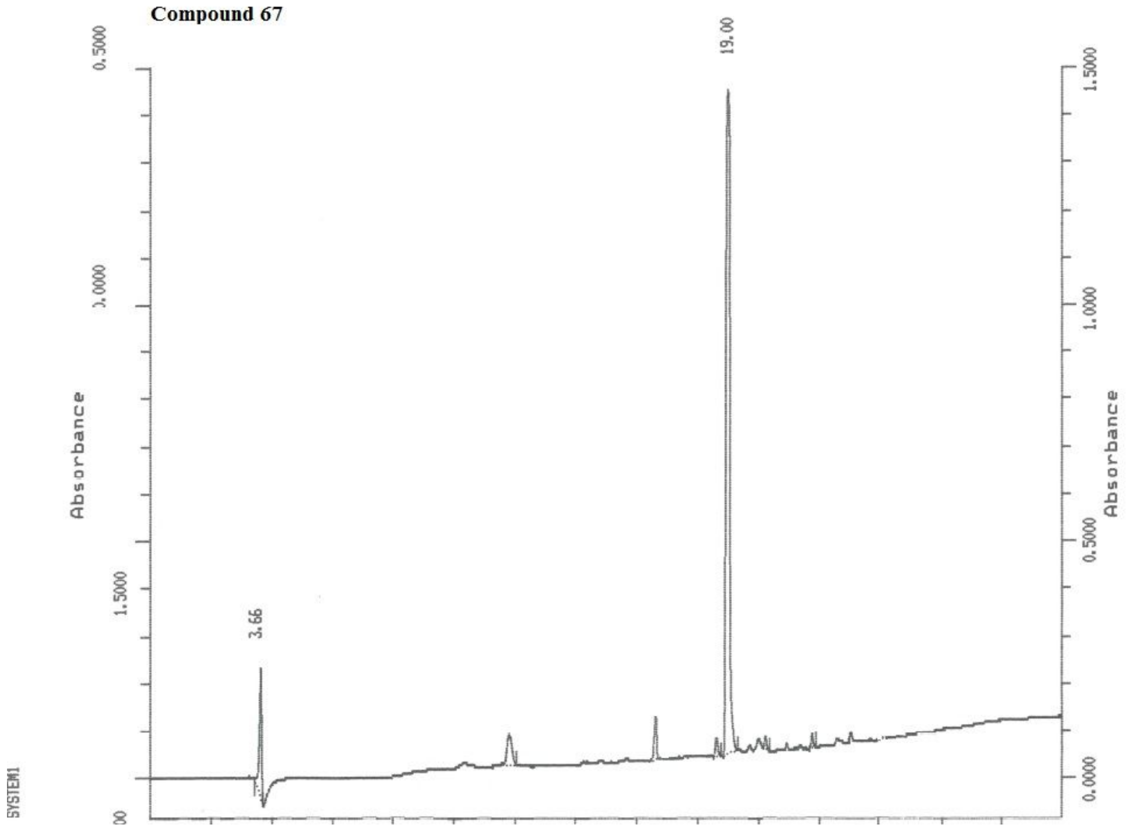

**Compound 39**

MS (ESI): m/z calcd for $C_{85}H_{141}N_{25}O_{19}S$ $[M+3H]^{3+}$ 617.42 found 617.70. $t_R$ = 14.95

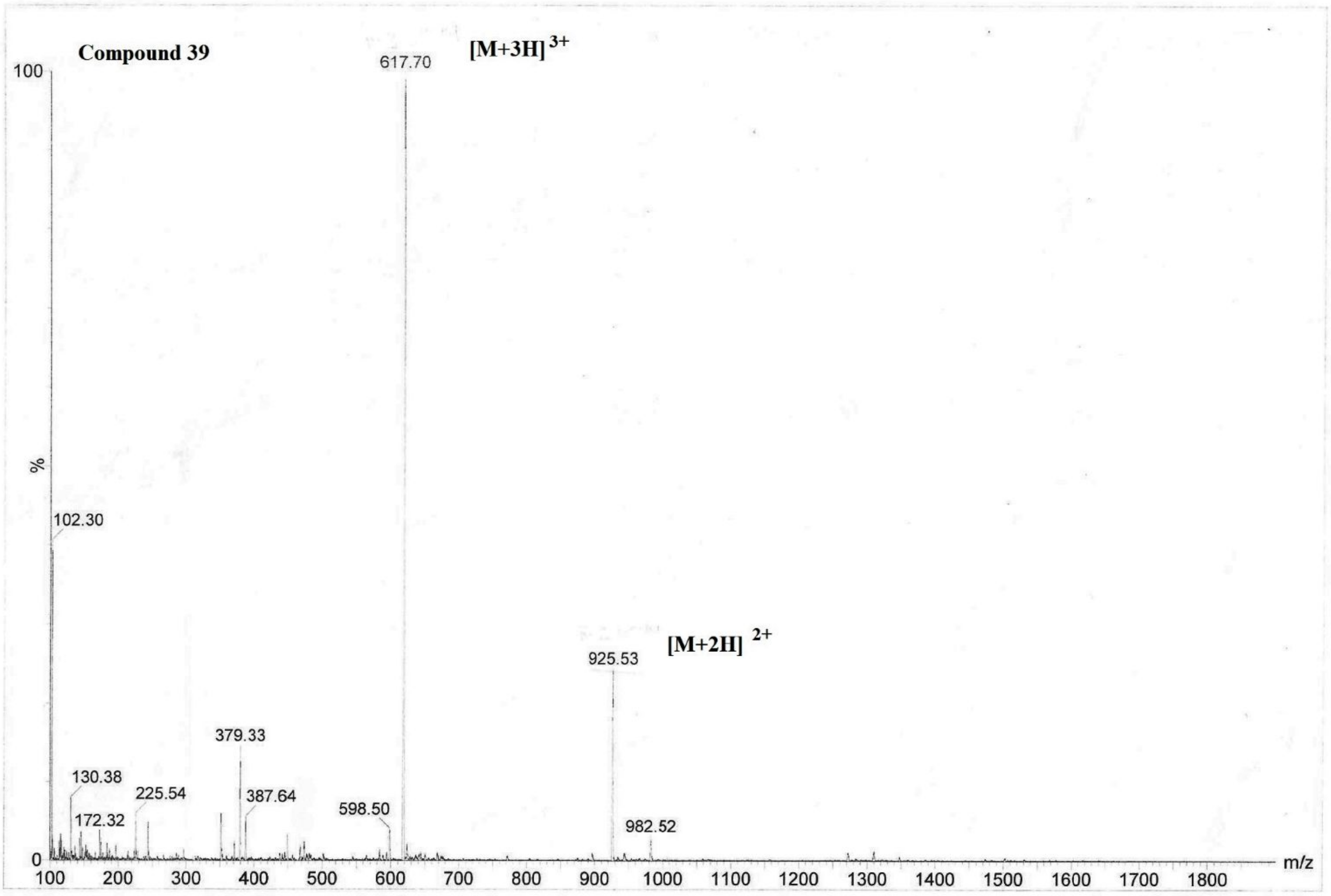


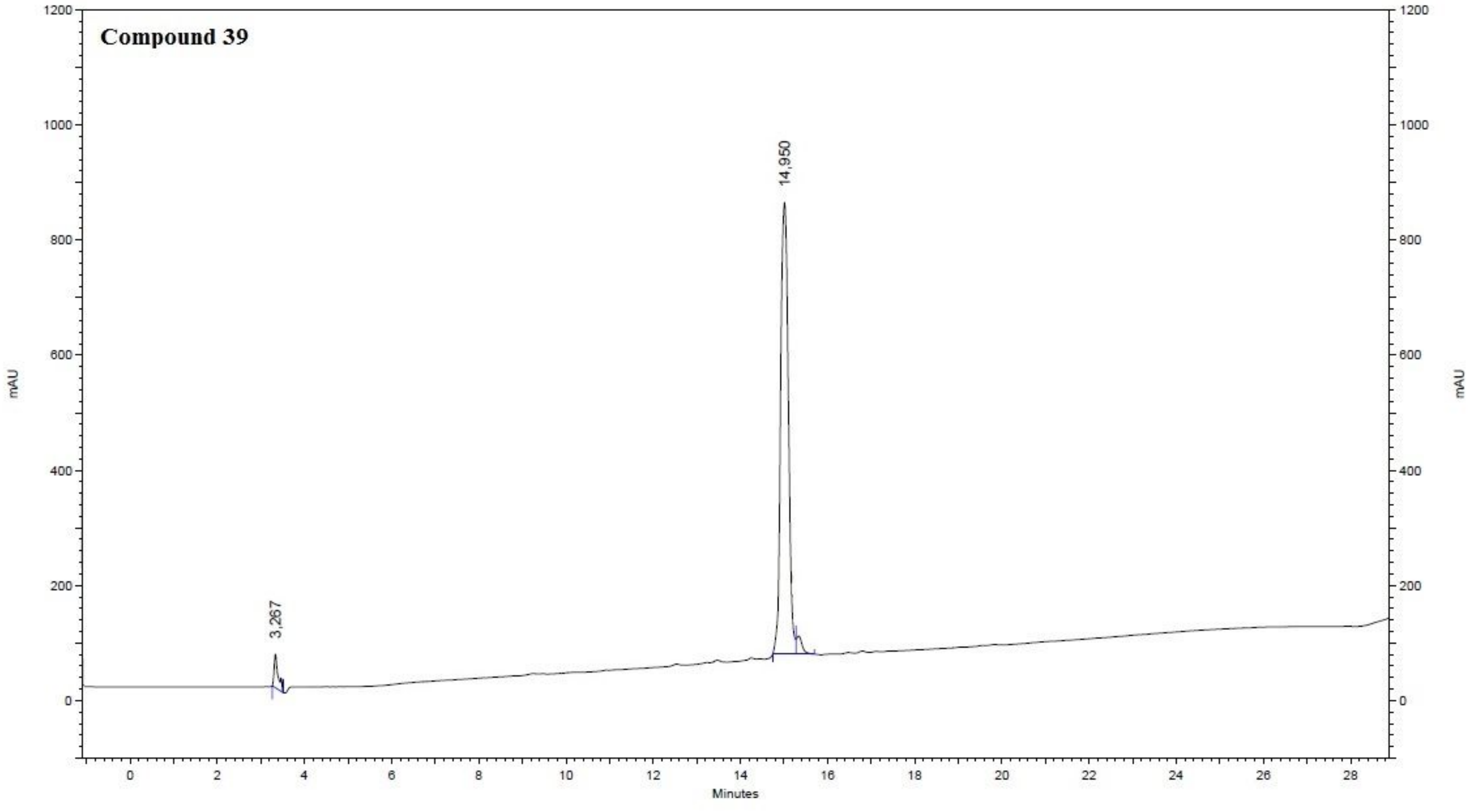

## Compound 39 (HRMS analysis)

## HRMS (teo) for $[M+4H]^{4+}$

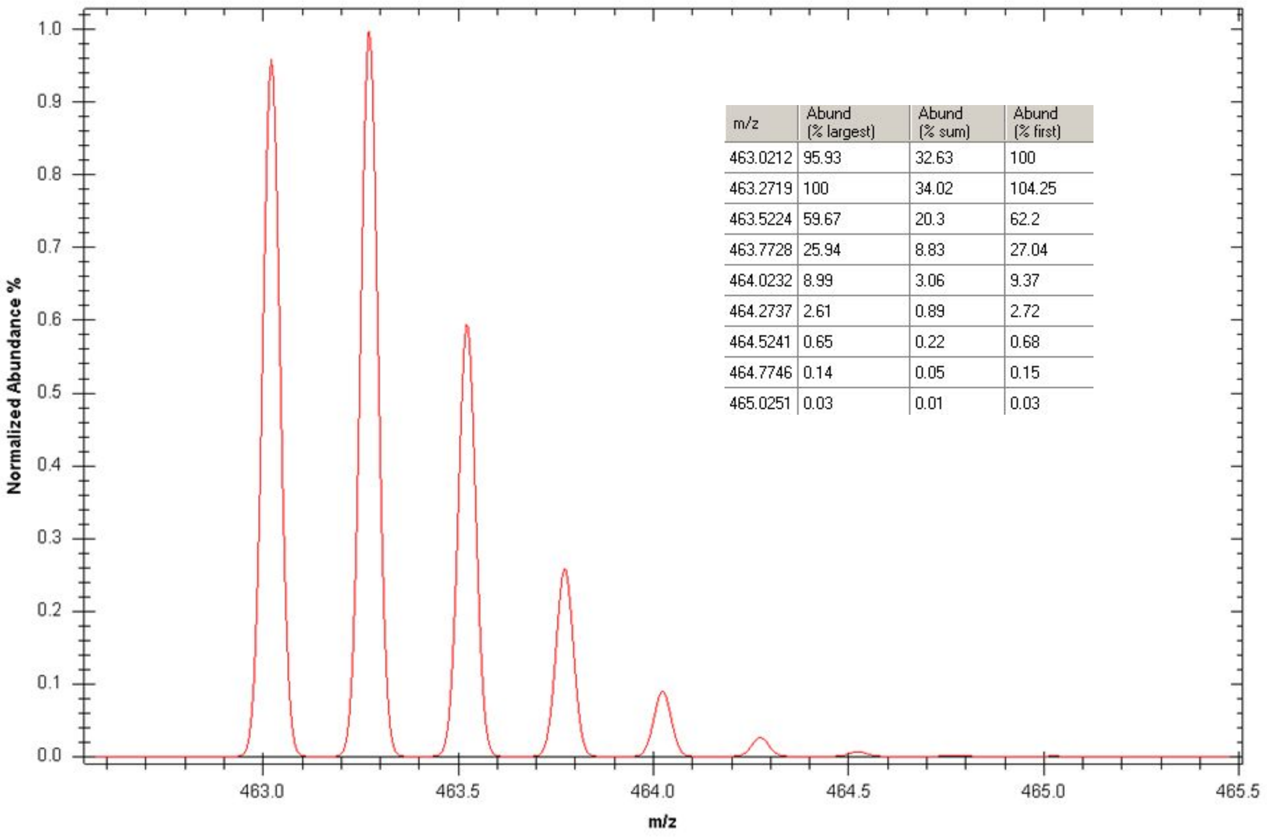


| m/z | Abund (% largest) | Abund (% sum) | Abund (% first) |
|---|---|---|---|
| 463.0212 | 95.93 | 32.63 | 100 |
| 463.2719 | 100 | 34.02 | 104.25 |
| 463.5224 | 59.67 | 20.3 | 62.2 |
| 463.7728 | 25.94 | 8.83 | 27.04 |
| 464.0232 | 8.99 | 3.06 | 9.37 |
| 464.2737 | 2.61 | 0.89 | 2.72 |
| 464.5241 | 0.65 | 0.22 | 0.68 |
| 464.7746 | 0.14 | 0.05 | 0.15 |
| 465.0251 | 0.03 | 0.01 | 0.03 |

## HRMS (exp) for $[M+4H]^{4+}$

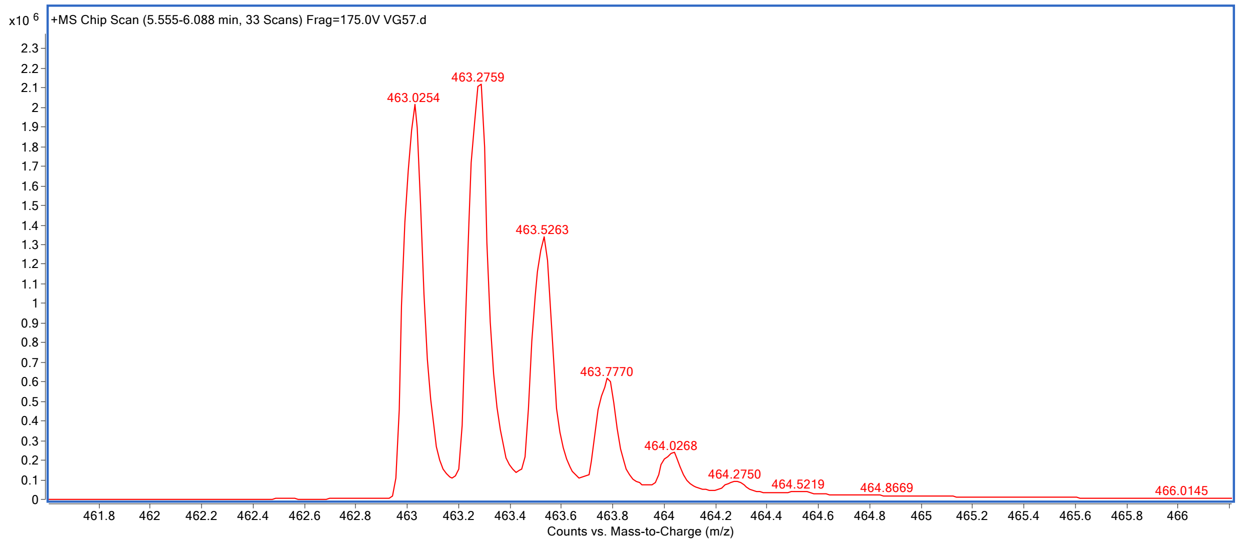

## Compound 40

MS (ESI): m/z calcd for $C_{87}H_{145}N_{25}O_{19}S$ $[M+3H]^{3+}$ 626.78 found 626.84. $t_R$ = 16.15

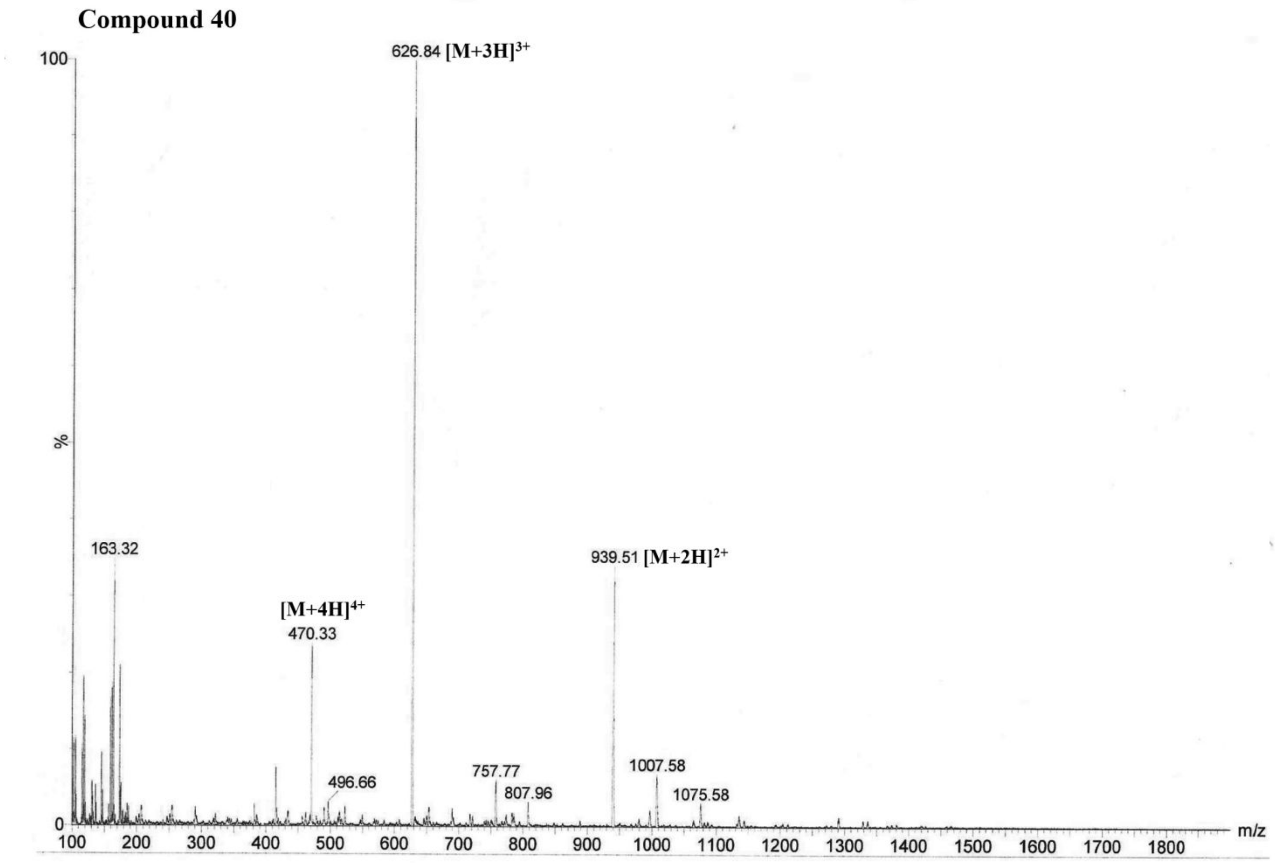


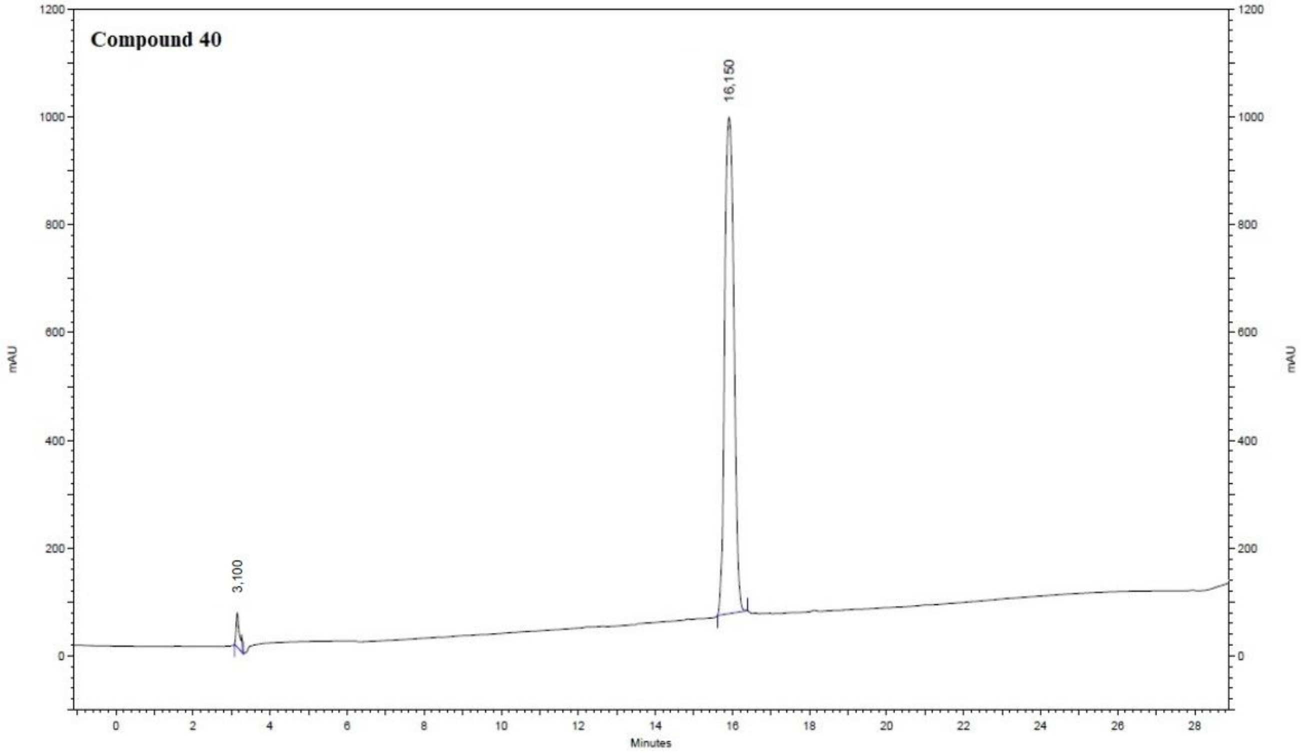

## Compound 40 (HRMS analysis)

## HRMS (teo) for $[M+3H]^{3+}$

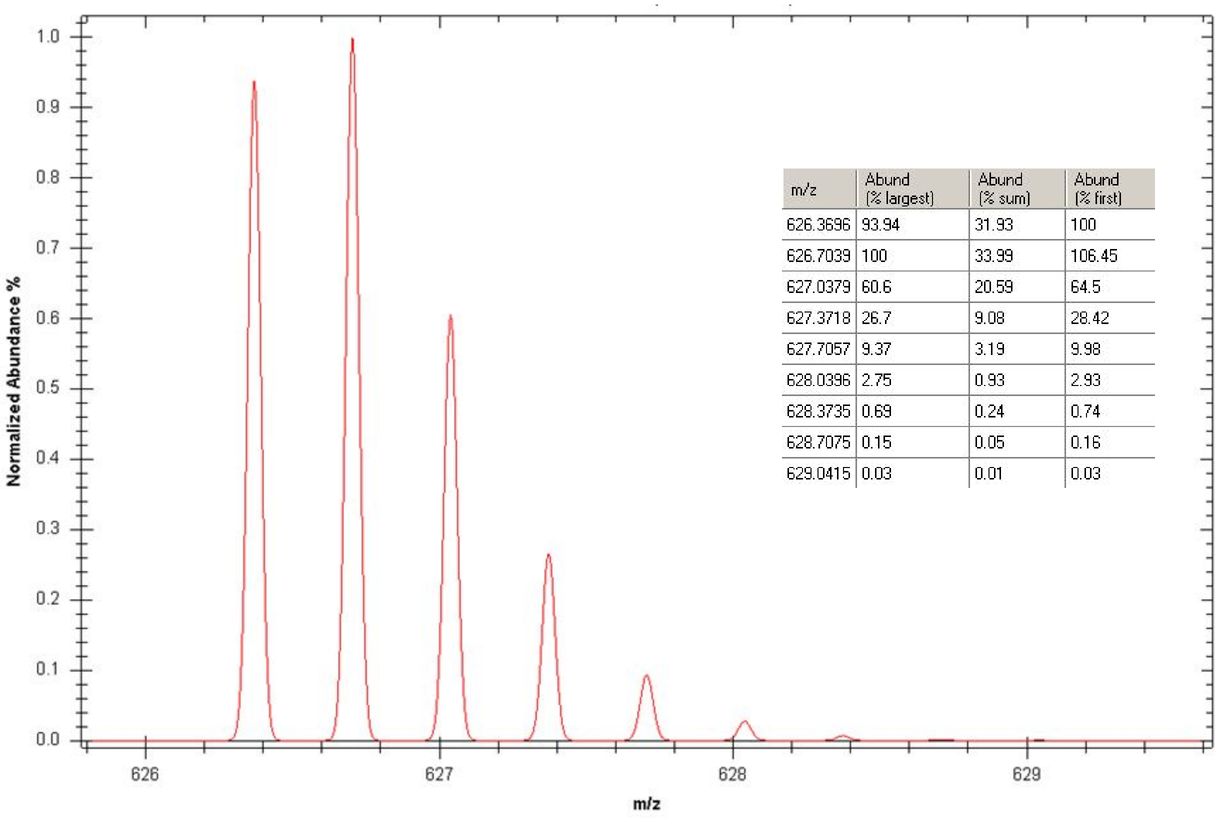


| m/z | Abund (% largest) | Abund (% sum) | Abund (% first) |
|---|---|---|---|
| 626.3696 | 93.94 | 31.93 | 100 |
| 626.7039 | 100 | 33.99 | 106.45 |
| 627.0379 | 60.6 | 20.59 | 64.5 |
| 627.3718 | 26.7 | 9.08 | 28.42 |
| 627.7057 | 9.37 | 3.19 | 9.98 |
| 628.0396 | 2.75 | 0.93 | 2.93 |
| 628.3735 | 0.69 | 0.24 | 0.74 |
| 628.7075 | 0.15 | 0.05 | 0.16 |
| 629.0415 | 0.03 | 0.01 | 0.03 |

## HRMS (exp) for $[M+3H]^{3+}$

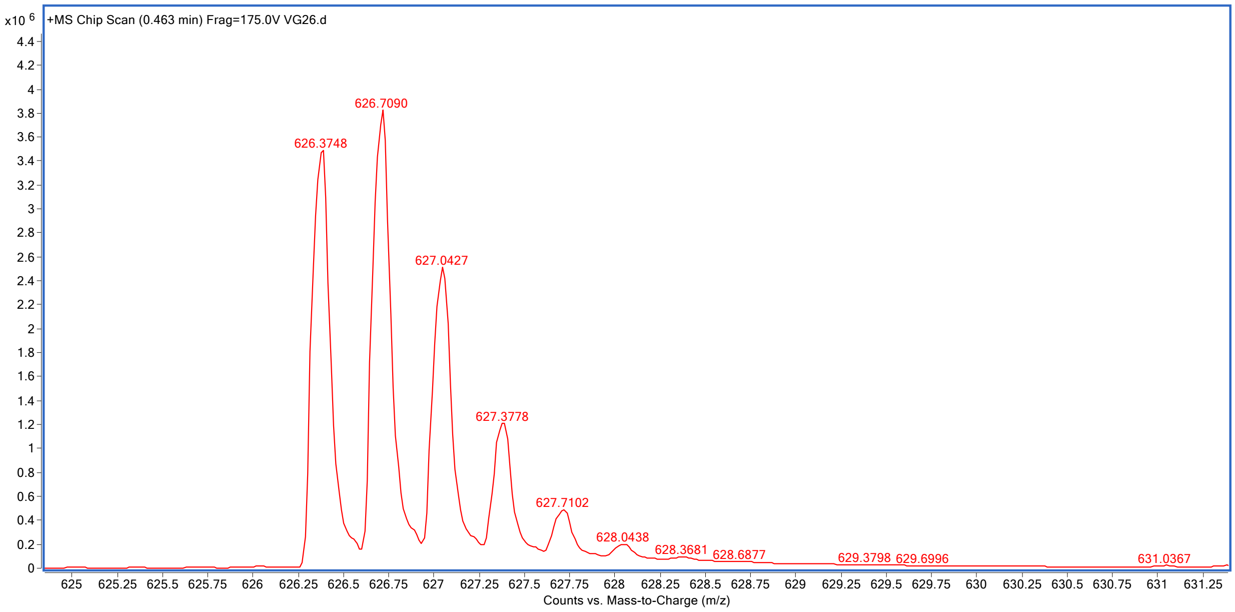

## Compound 40 (HRMS analysis)

## HRMS (teo) for $[M+2H]^{2+}$

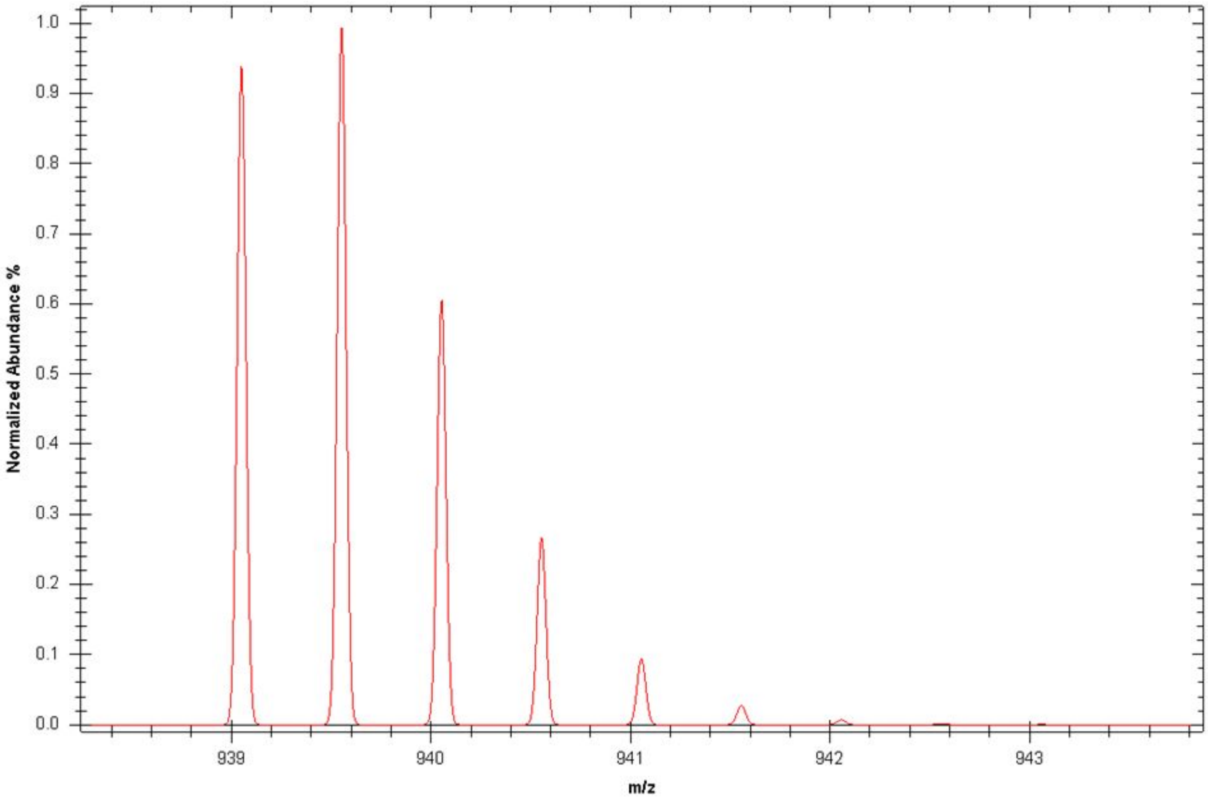


## HRMS (exp) for $[M+2H]^{2+}$

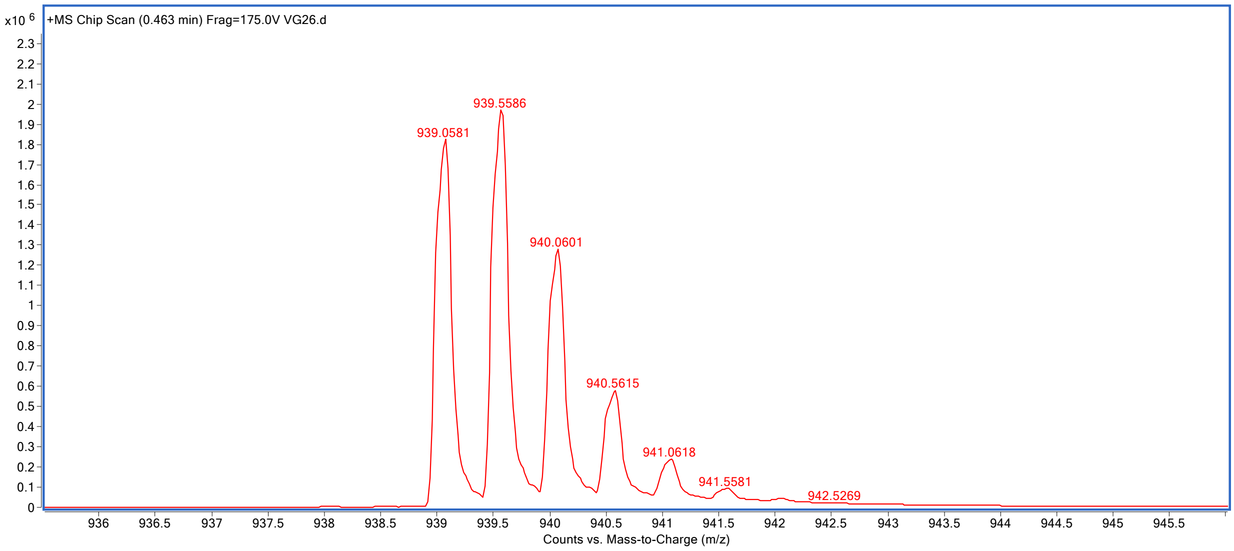

**Compound 41**

MS (ESI): m/z calcd for $C_{89}H_{149}N_{25}O_{19}S$ $[M+3H]^{3+}$ 636.13 found 636.17. $t_R$ = 17.42

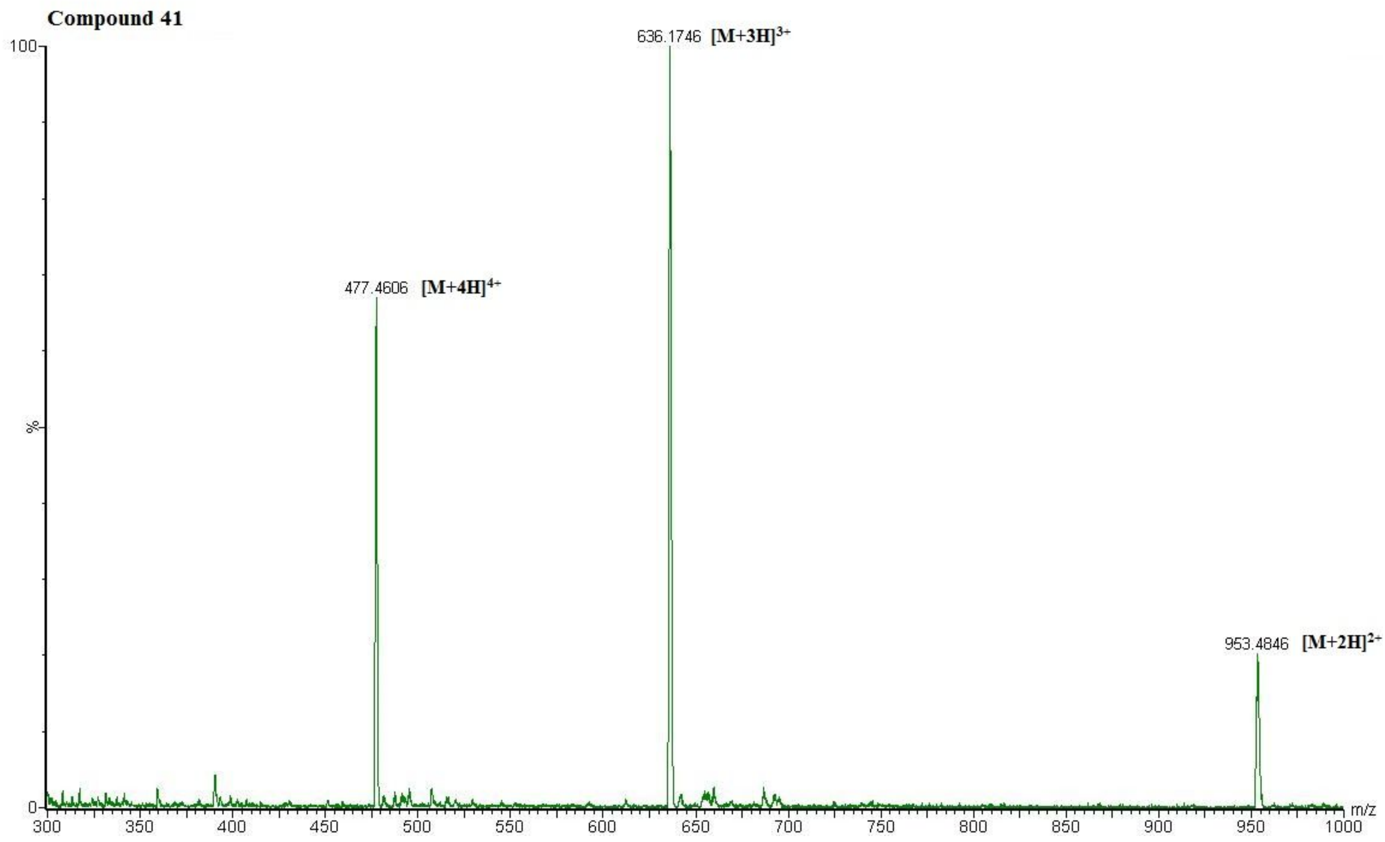


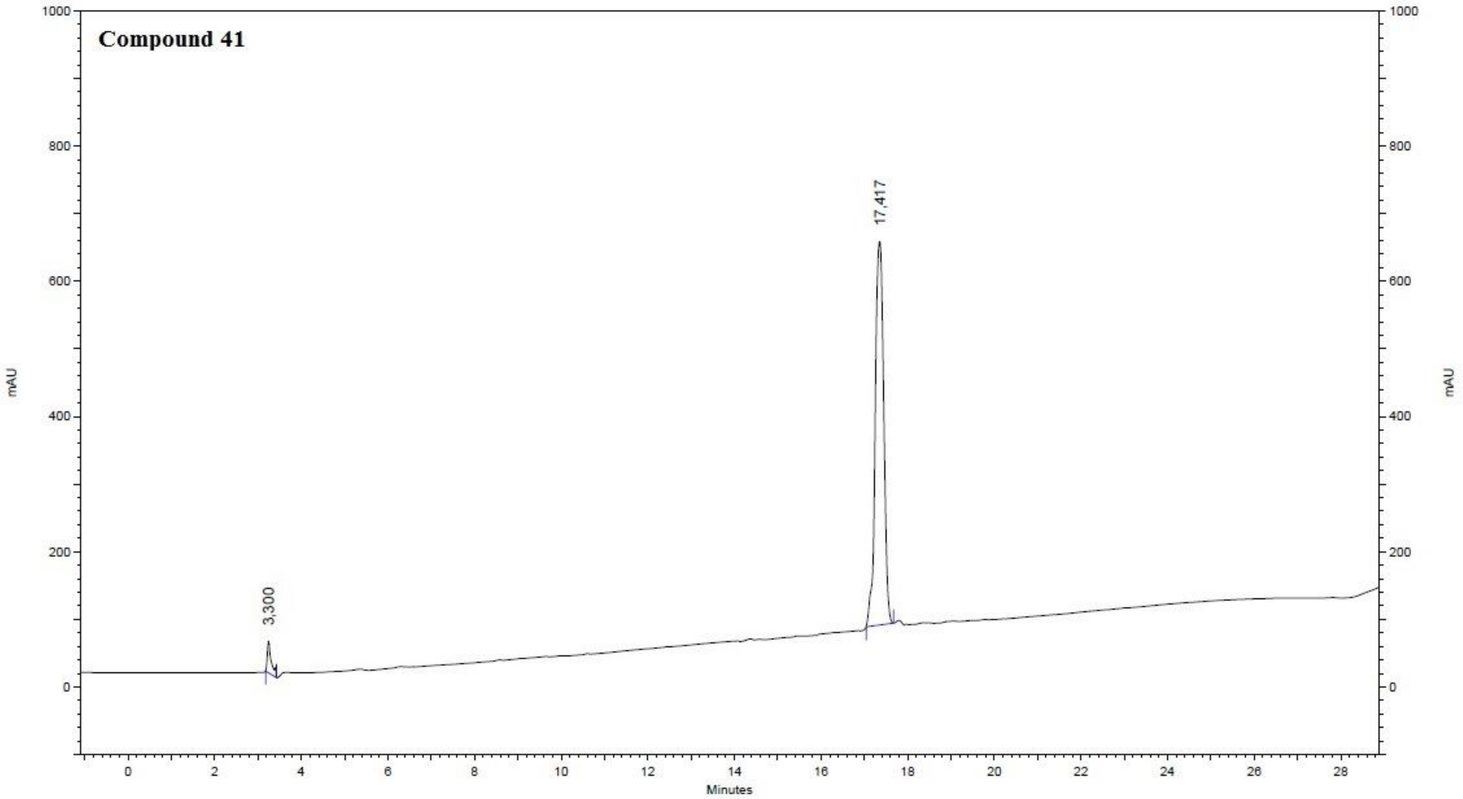

## Compound 41 (HRMS analysis)

| molecular formula | exact mass | | |
|---|---|---|---|
| $C_{89}H_{149}N_{25}O_{19}S$ | 1904,11823 | | |
| molecular ion | m/z (teo) | m/z (exp) | dm/z (ppm) |
| $[M+H]^{+}$ | 1905,12551 | - | - |
| $[M+2H]^{2+}$ | 953,06639 | 953,0641 | 2,4 |
| $[M+3H]^{3+}$ | 635,71335 | 635,7129 | 0,7 |
| $[M+4H]^{4+}$ | 477,03683 | 477,0368 | 0,1 |

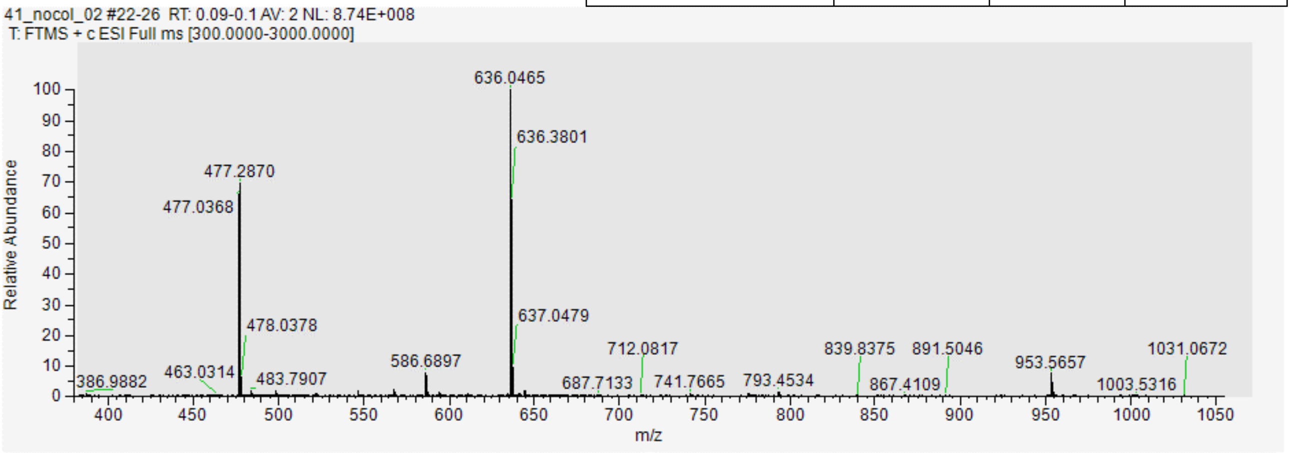


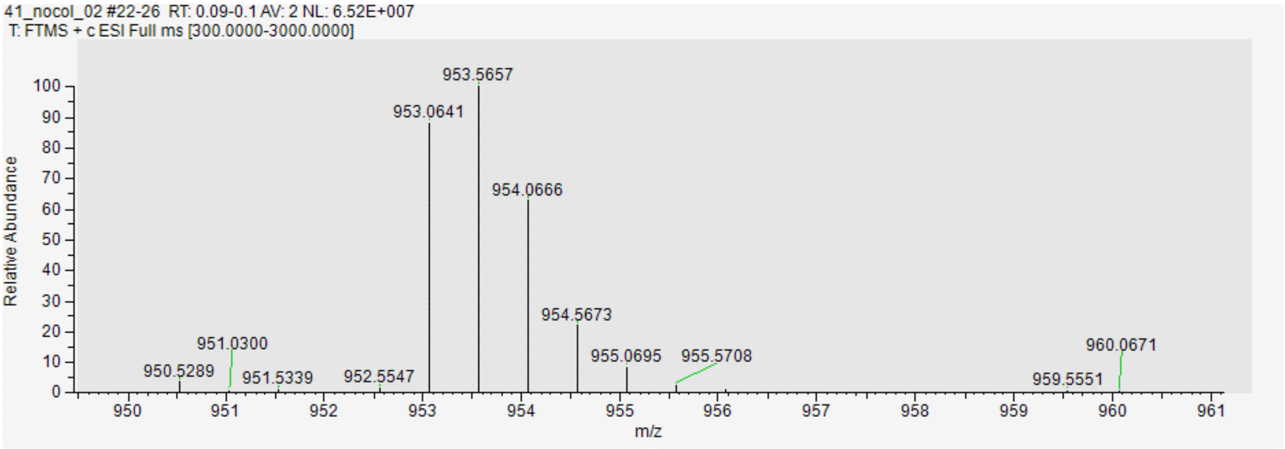


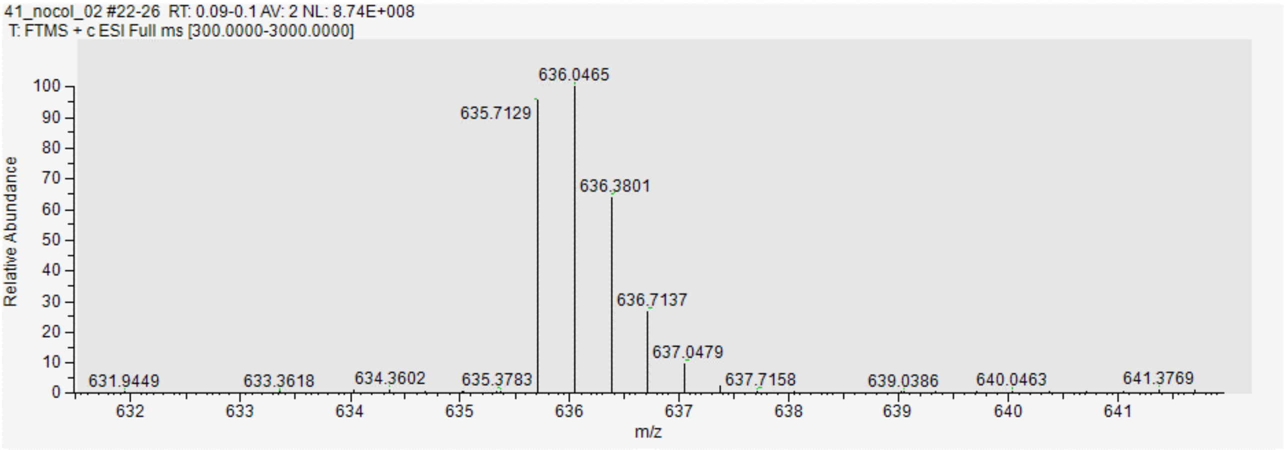

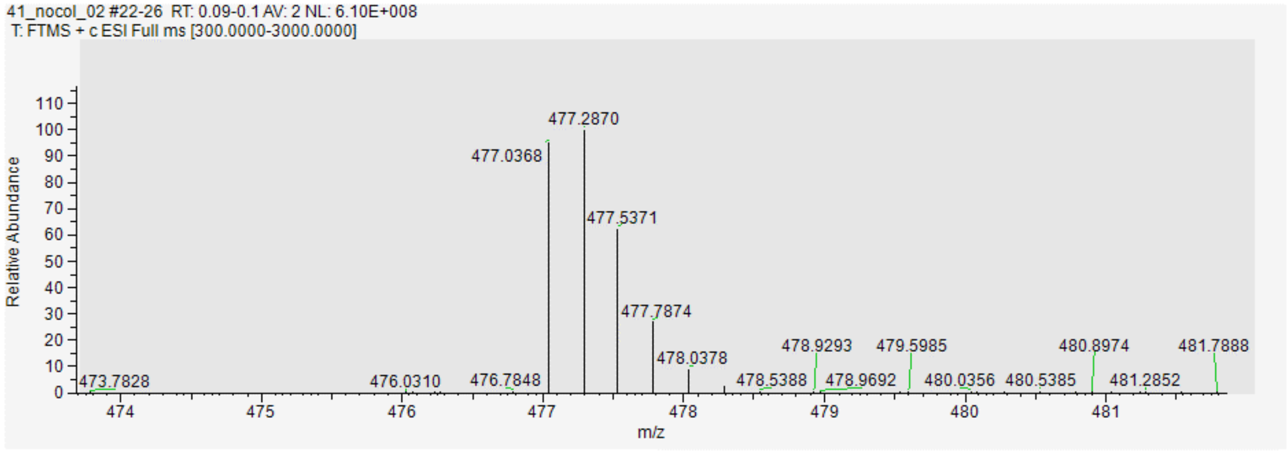
41_nocol_02 #22-26 RT: 0.09-0.1 AV: 2 NL: 6.10E+008
T: FTMS + c ESI Full ms [300.0000-3000.0000]
Relative Abundance
477.2870
477.0368
477.5371
477.7874
478.0378
478.9293
479.5985
480.8974
481.7888
473.7828
476.0310
476.7848
478.5388
478.9692
480.0356
480.5385
481.2852
m/z

**Compound 42**

MS (ESI): m/z calcd for $C_{90}H_{148}N_{28}O_{28}S$ $[M+3H]^{3+}$ 701.80 found 701.87. $t_R$ = 11.42

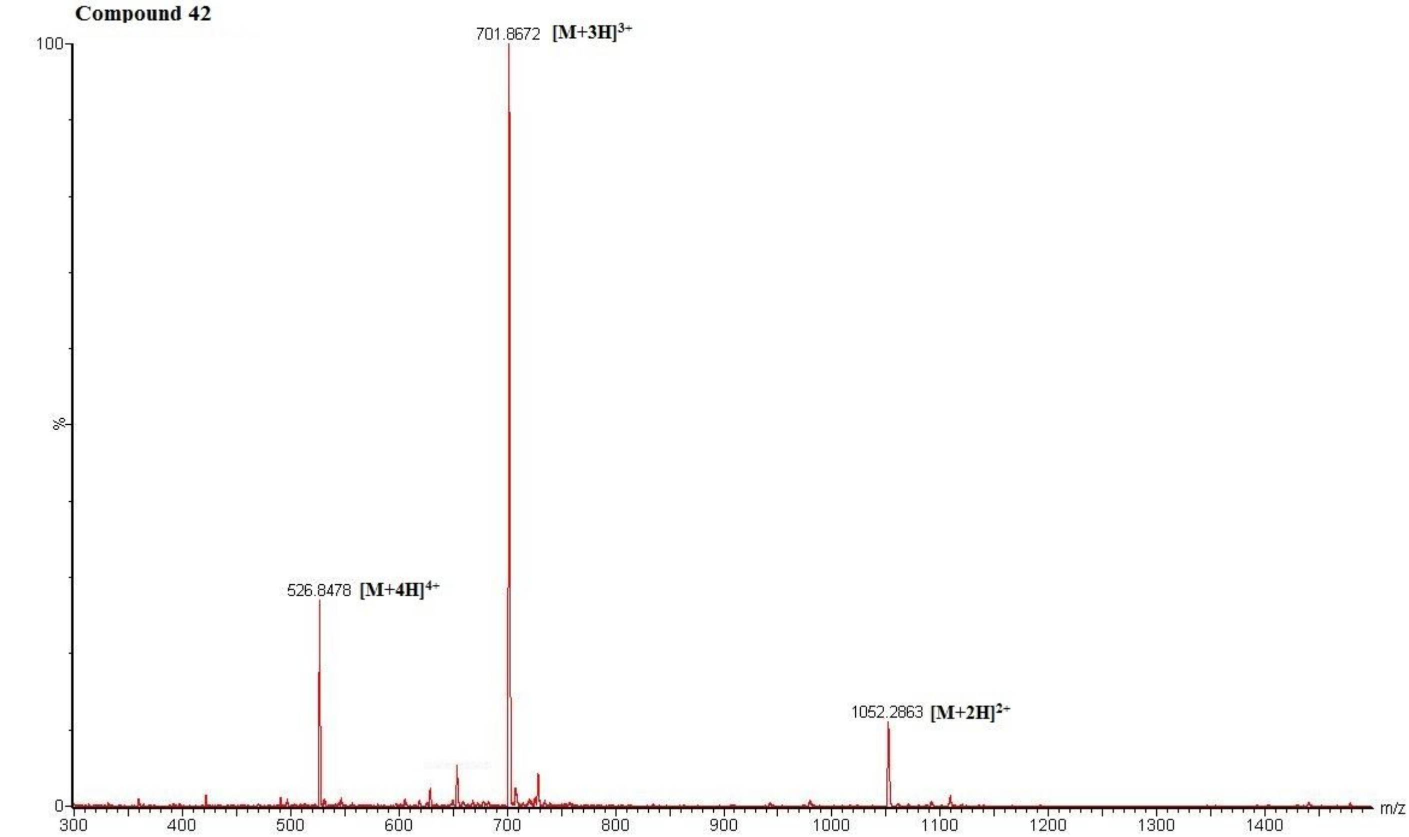


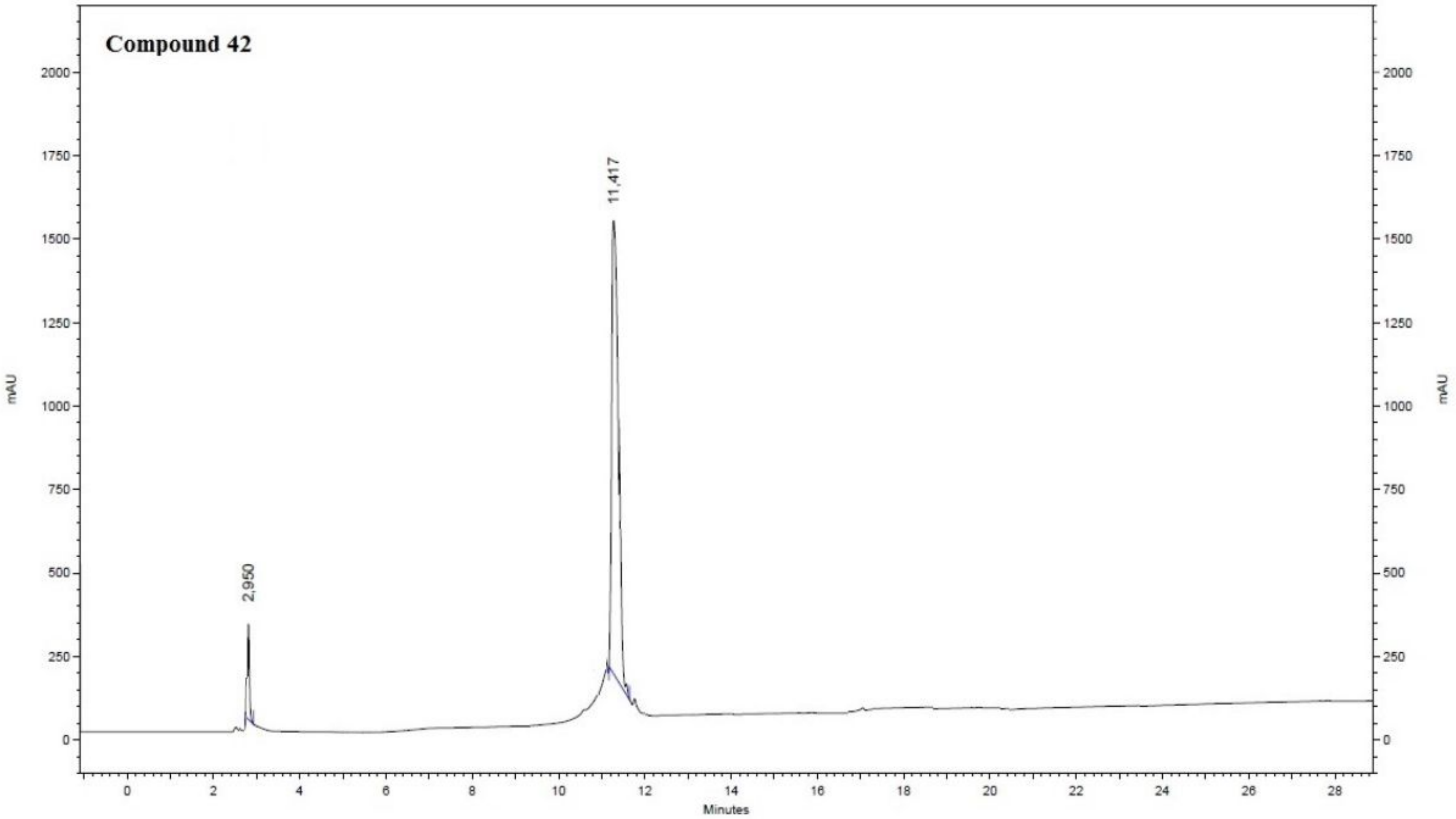

**Compound 42 (HRMS analysis)**

**HRMS (teo) for $[M+4H]^{4+}$**

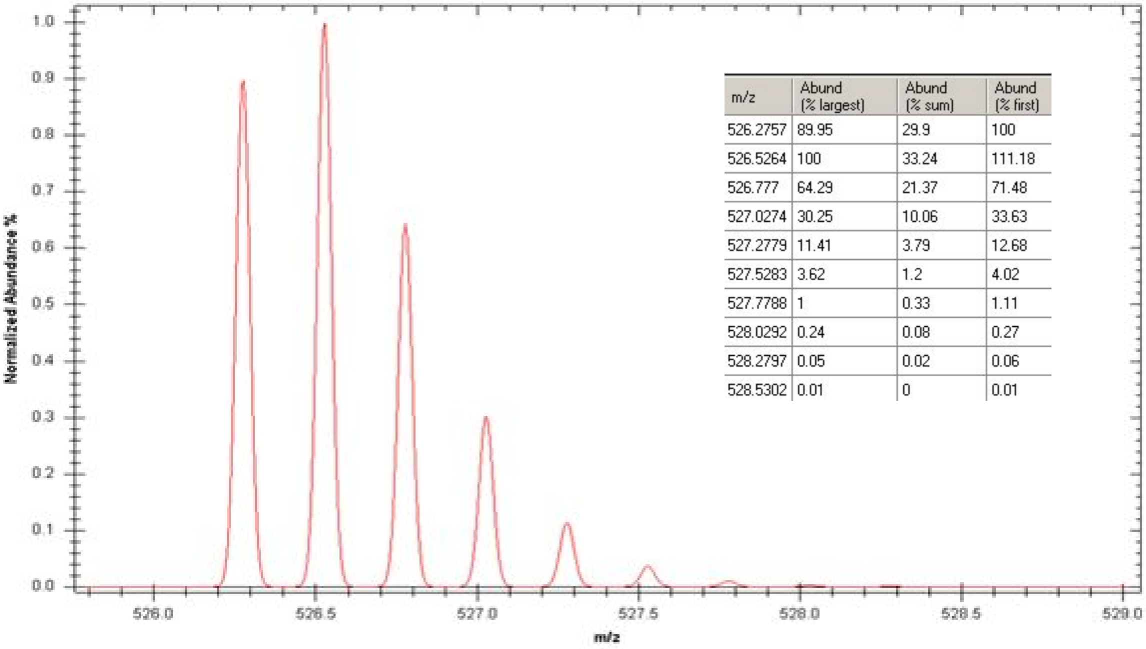


| m/z | Abund (% largest) | Abund (% sum) | Abund (% first) |
|---|---|---|---|
| 526.2757 | 89.95 | 29.9 | 100 |
| 526.5264 | 100 | 33.24 | 111.18 |
| 526.777 | 64.29 | 21.37 | 71.48 |
| 527.0274 | 30.25 | 10.06 | 33.63 |
| 527.2779 | 11.41 | 3.79 | 12.68 |
| 527.5283 | 3.62 | 1.2 | 4.02 |
| 527.7788 | 1 | 0.33 | 1.11 |
| 528.0292 | 0.24 | 0.08 | 0.27 |
| 528.2797 | 0.05 | 0.02 | 0.06 |
| 528.5302 | 0.01 | 0 | 0.01 |

**HRMS (exp) for $[M+4H]^{4+}$**

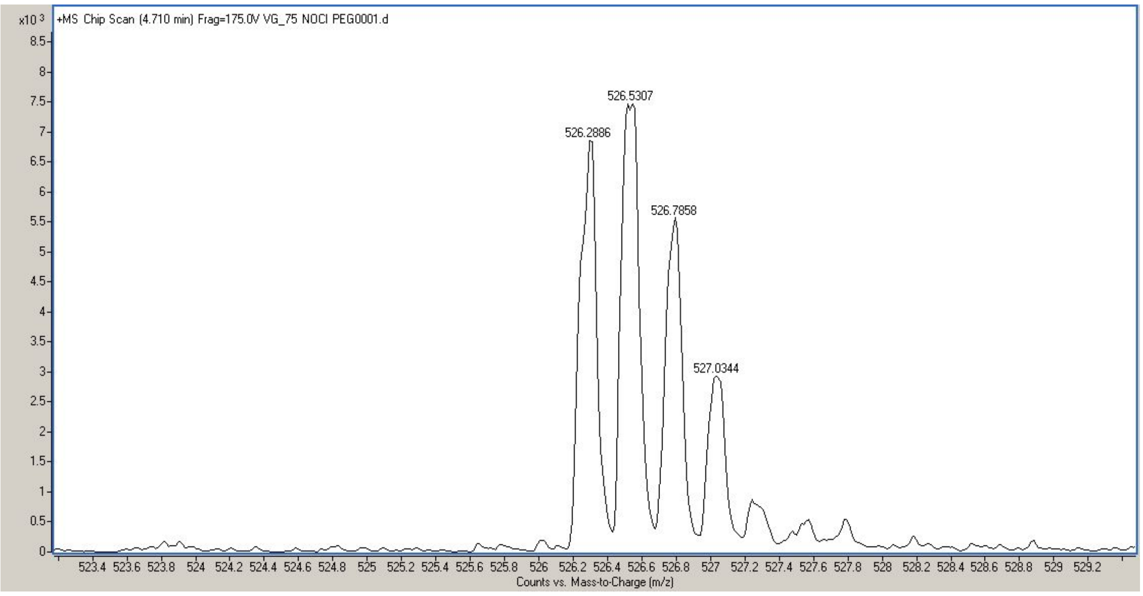

**Compound 43**

MS (ESI): m/z calcd for $C_{126}H_{217}N_{43}O_{31}S$ $[M+4H]^{4+}$ 716.61 found 716.72. $t_R$ = 15.73

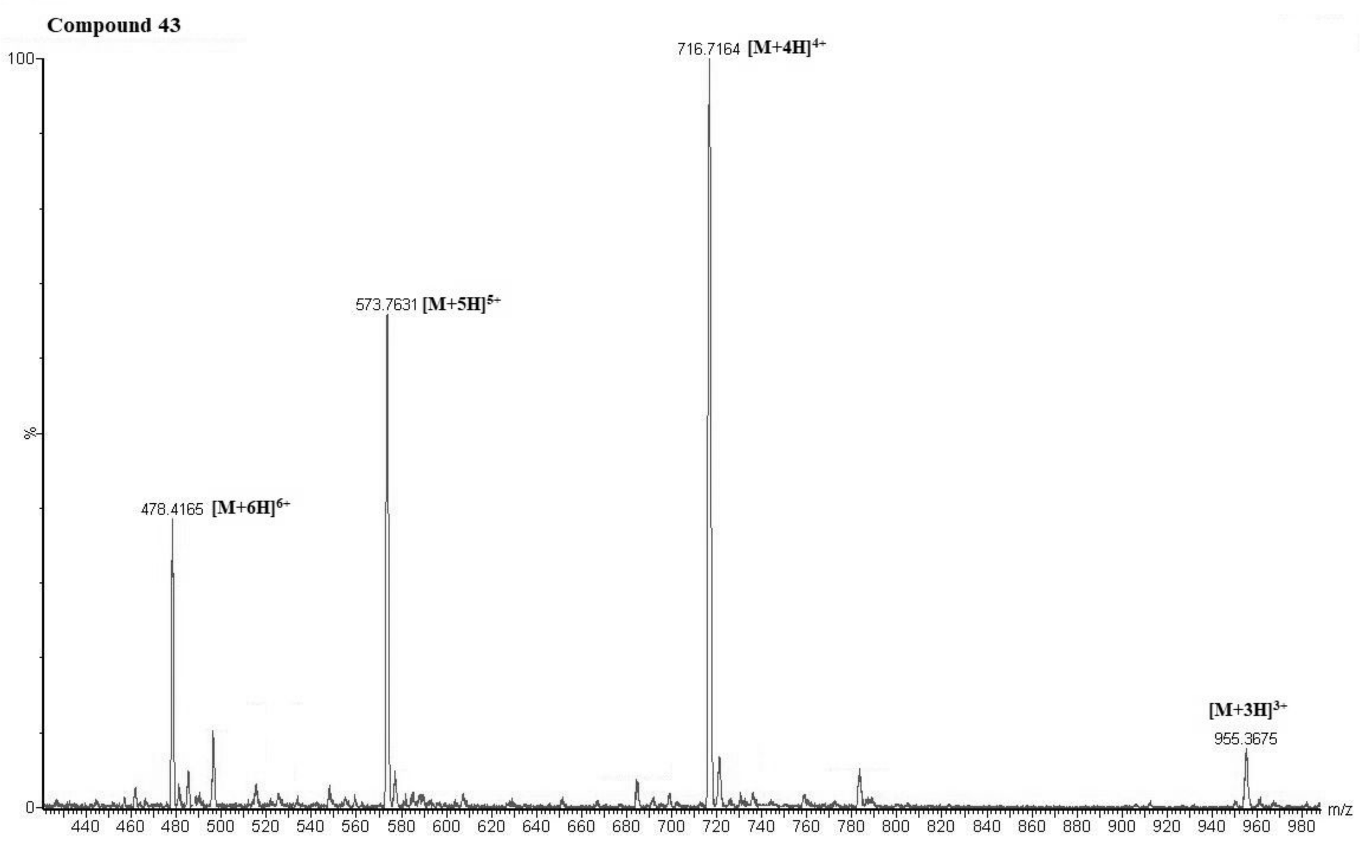


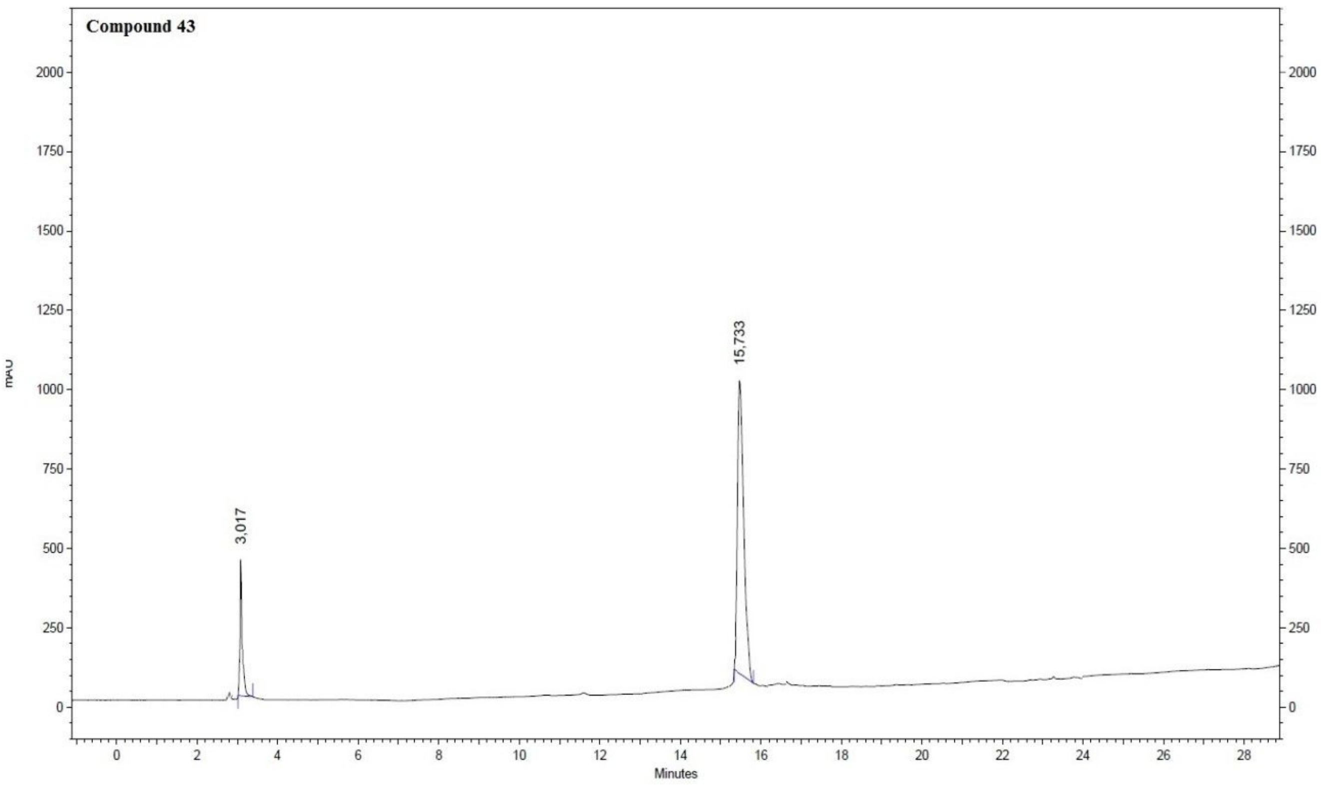

**Compound 43 (HRMS analysis)**

**HRMS (teo) for $[M+7H]^{7+}$**

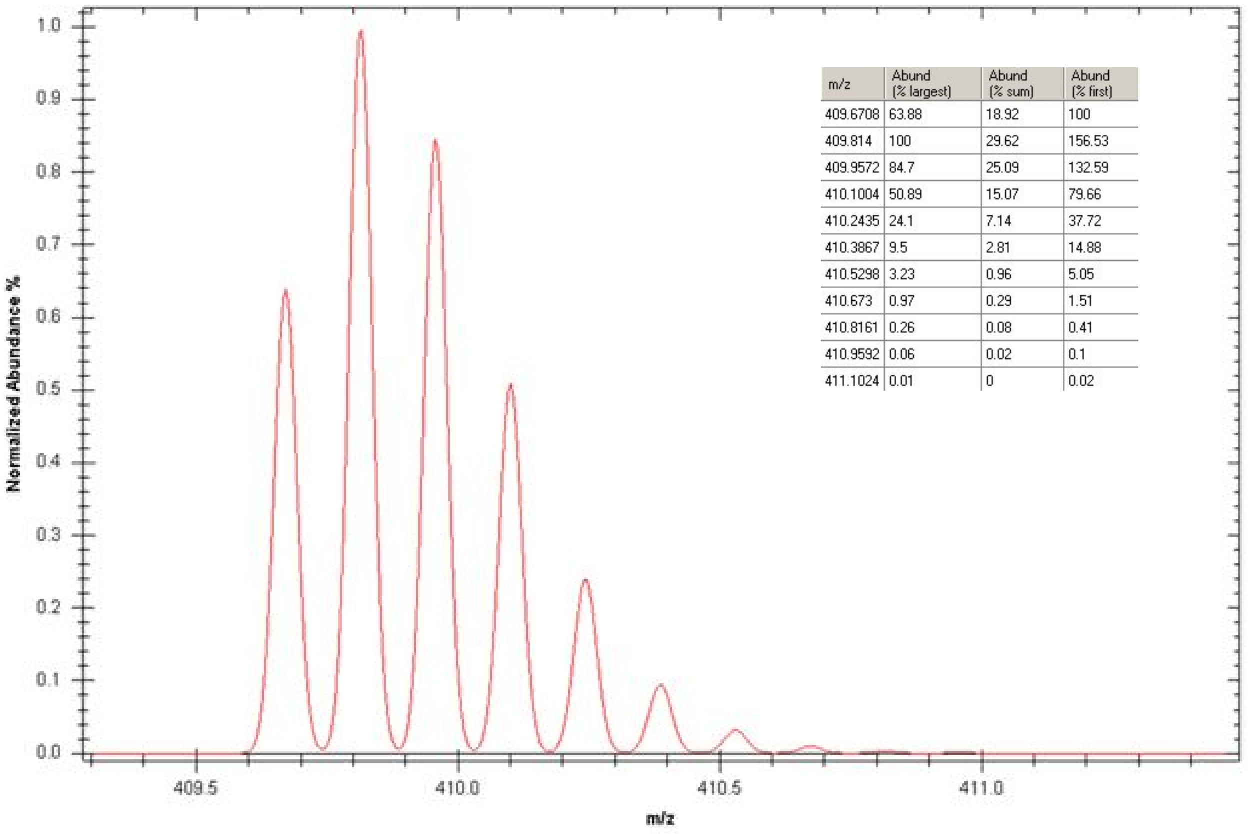


| m/z | Abund (% largest) | Abund (% sum) | Abund (% first) |
|---|---|---|---|
| 409.6708 | 63.88 | 18.92 | 100 |
| 409.814 | 100 | 29.62 | 156.53 |
| 409.9572 | 84.7 | 25.09 | 132.59 |
| 410.1004 | 50.89 | 15.07 | 79.66 |
| 410.2435 | 24.1 | 7.14 | 37.72 |
| 410.3867 | 9.5 | 2.81 | 14.88 |
| 410.5298 | 3.23 | 0.96 | 5.05 |
| 410.673 | 0.97 | 0.29 | 1.51 |
| 410.8161 | 0.26 | 0.08 | 0.41 |
| 410.9592 | 0.06 | 0.02 | 0.1 |
| 411.1024 | 0.01 | 0 | 0.02 |

**HRMS (exp) for $[M+7H]^{7+}$**

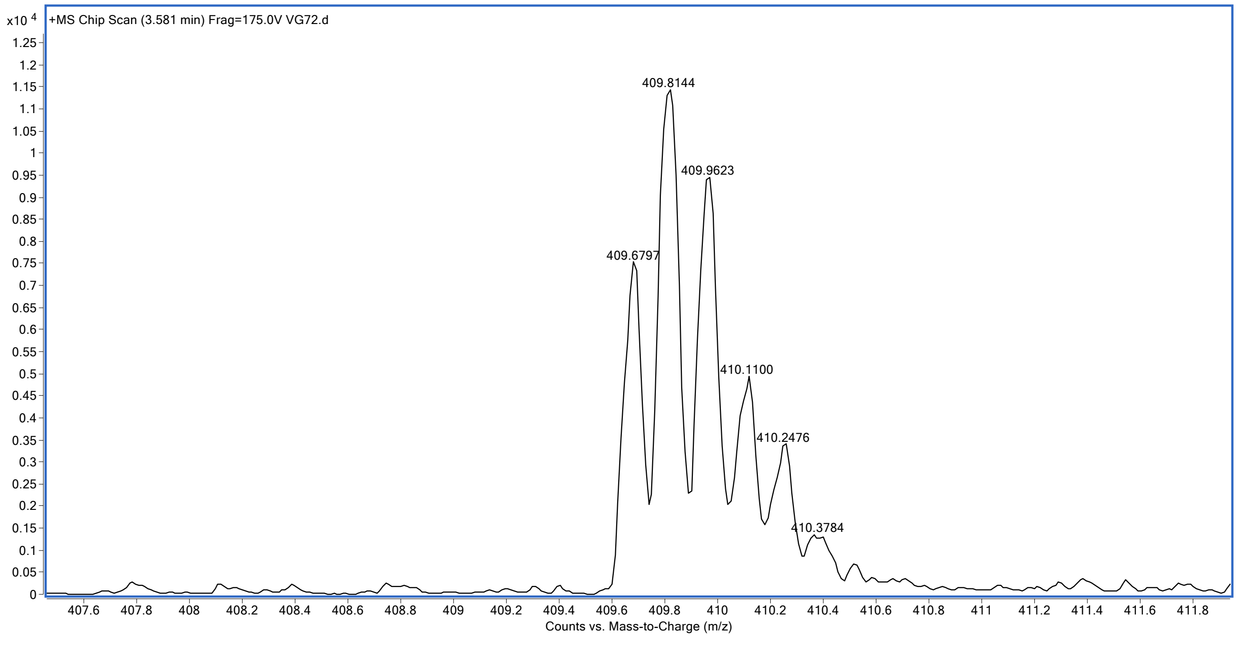

**Compound 44**

MS (ESI): m/z calcd for $C_{120}H_{205}N_{43}O_{31}S$ $[M+4H]^{4+}$ 695.57 found 695.80. $t_R$ = 11.30

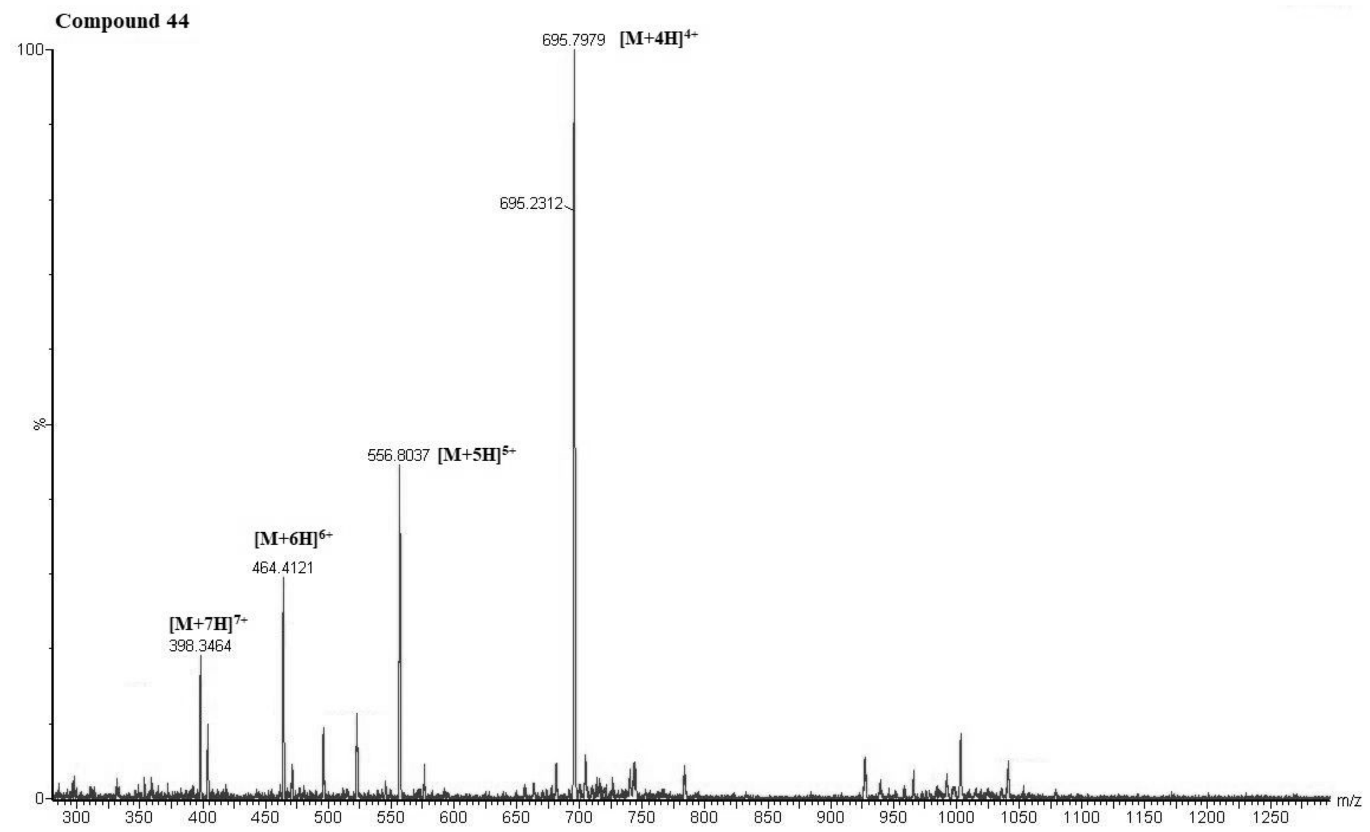


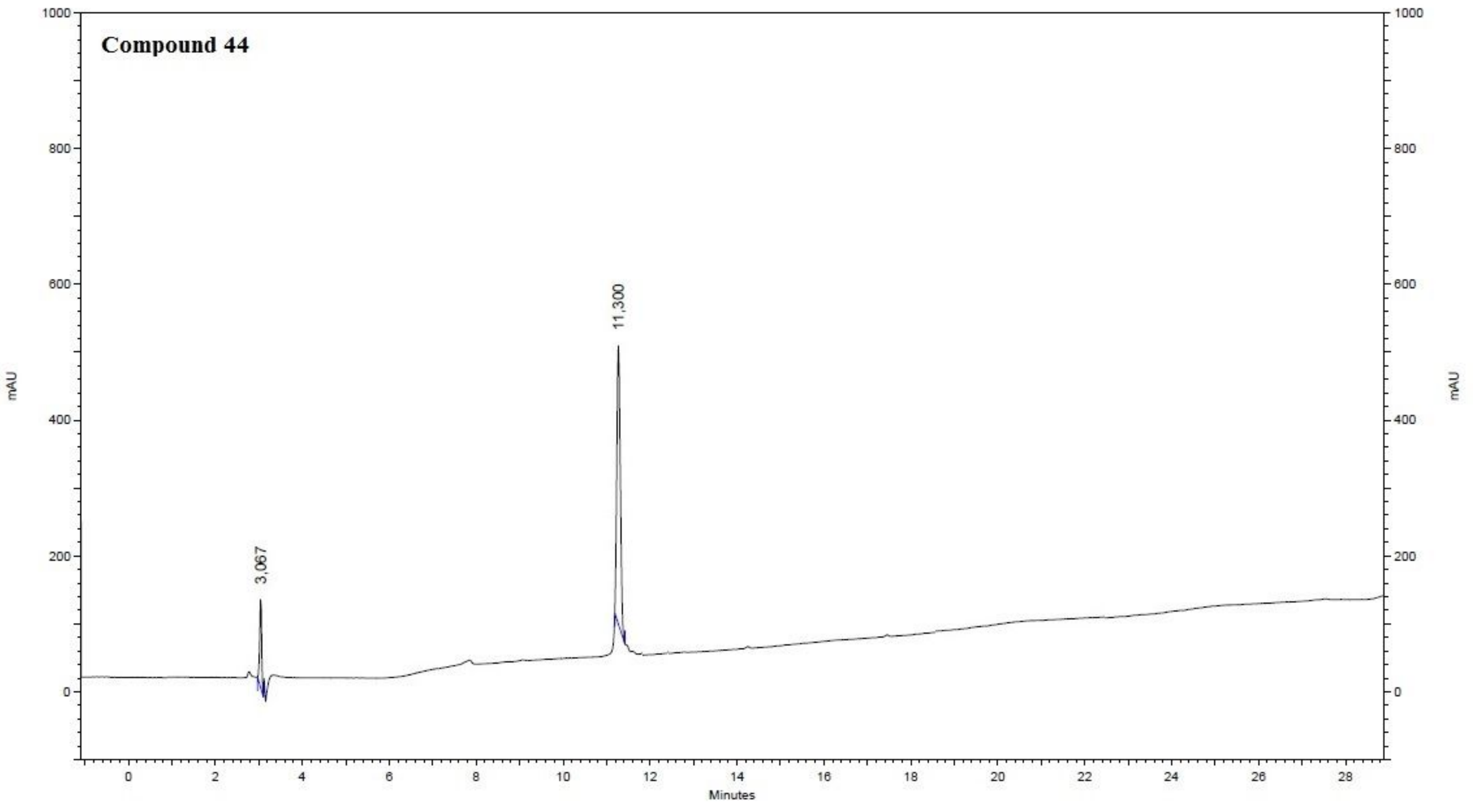

## Compound 44 (HRMS analysis)

## HRMS (teo) for $[M+6H]^{6+}$

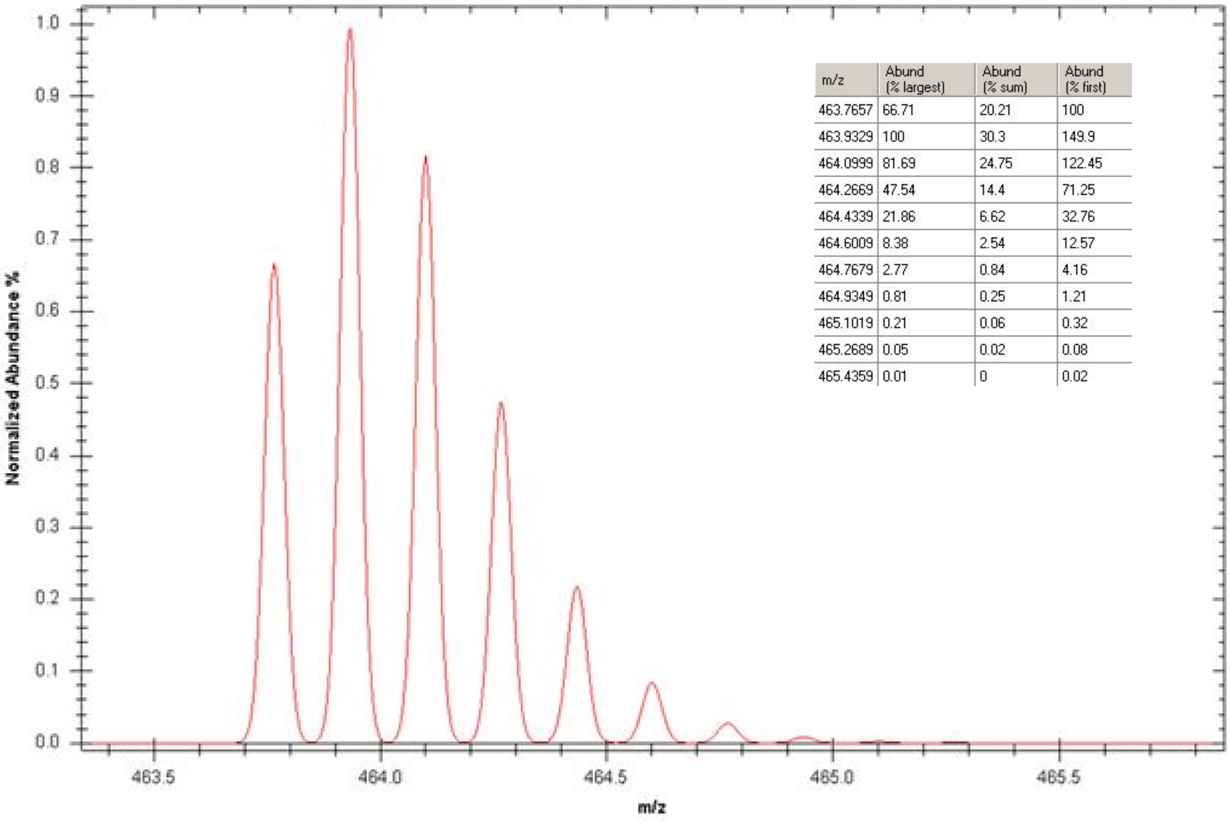


| m/z | Abund (% largest) | Abund (% sum) | Abund (% first) |
|---|---|---|---|
| 463.7657 | 66.71 | 20.21 | 100 |
| 463.9329 | 100 | 30.3 | 149.9 |
| 464.0999 | 81.69 | 24.75 | 122.45 |
| 464.2669 | 47.54 | 14.4 | 71.25 |
| 464.4339 | 21.86 | 6.62 | 32.76 |
| 464.6009 | 8.38 | 2.54 | 12.57 |
| 464.7679 | 2.77 | 0.84 | 4.16 |
| 464.9349 | 0.81 | 0.25 | 1.21 |
| 465.1019 | 0.21 | 0.06 | 0.32 |
| 465.2689 | 0.05 | 0.02 | 0.08 |
| 465.4359 | 0.01 | 0 | 0.02 |

## HRMS (exp) for $[M+6H]^{6+}$

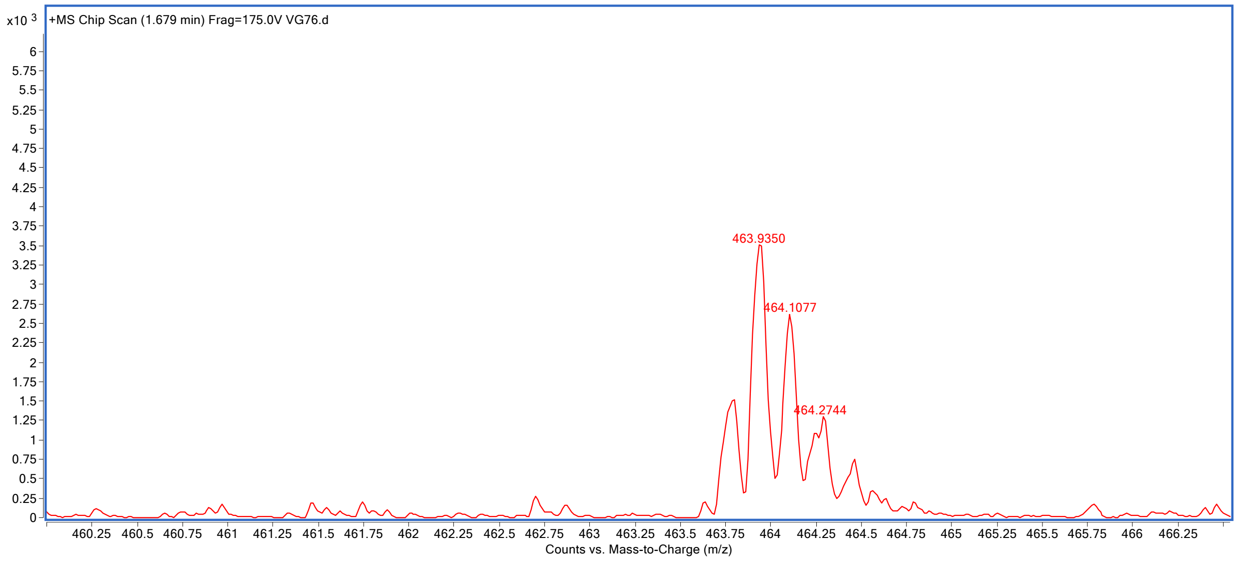

## Compound 45

MS (ESI): m/z calcd for $C_{114}H_{175}N_{37}O_{43}S$ $[M+3H]^{3+}$ 928.97 found 928.87. $t_R$ = 11.22

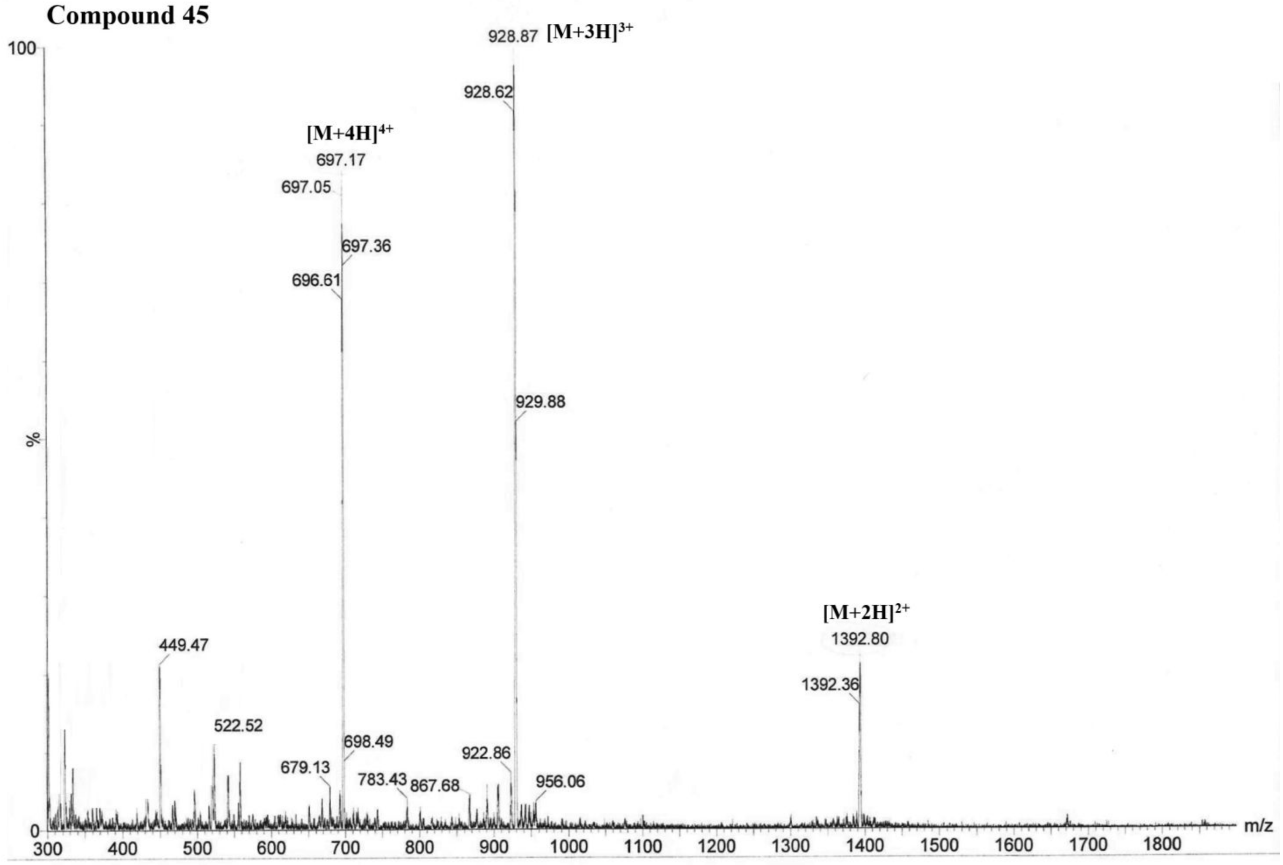


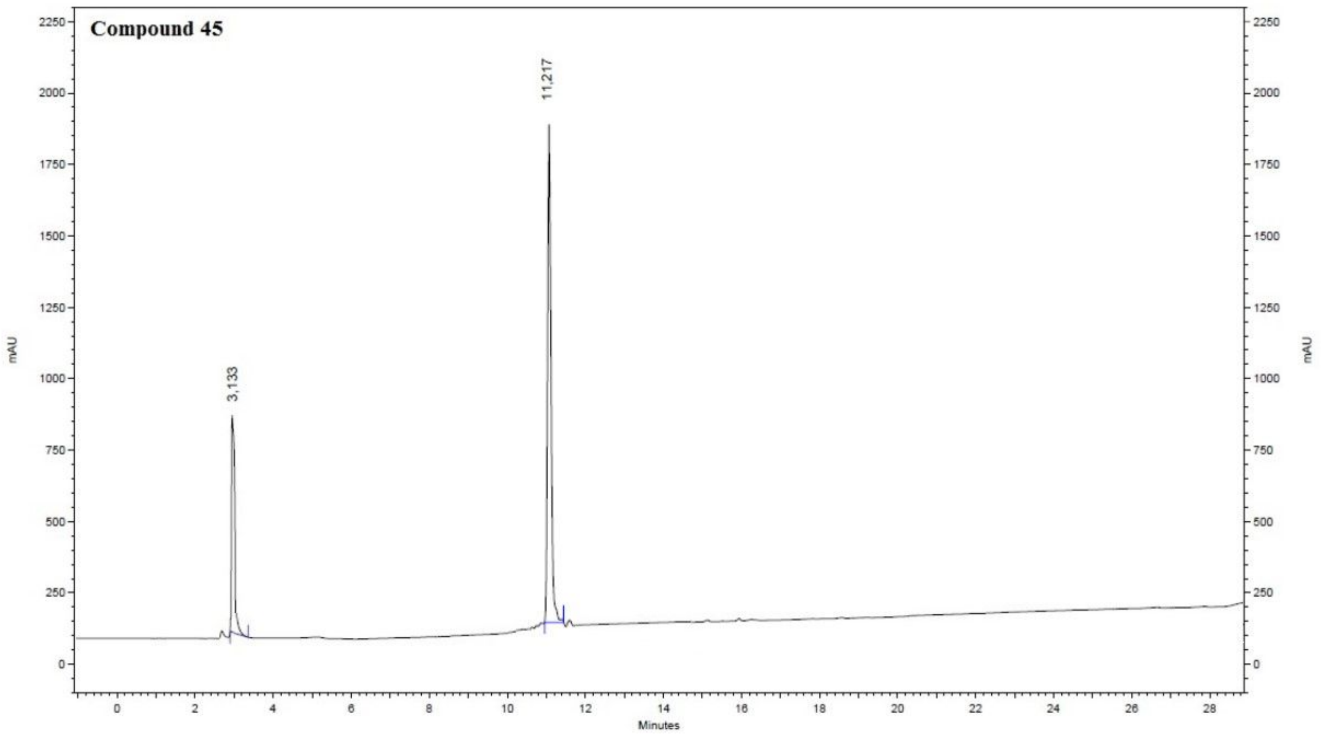

## Compound 45 (HRMS analysis)

## HRMS (teo) for $[M+5H]^{5+}$

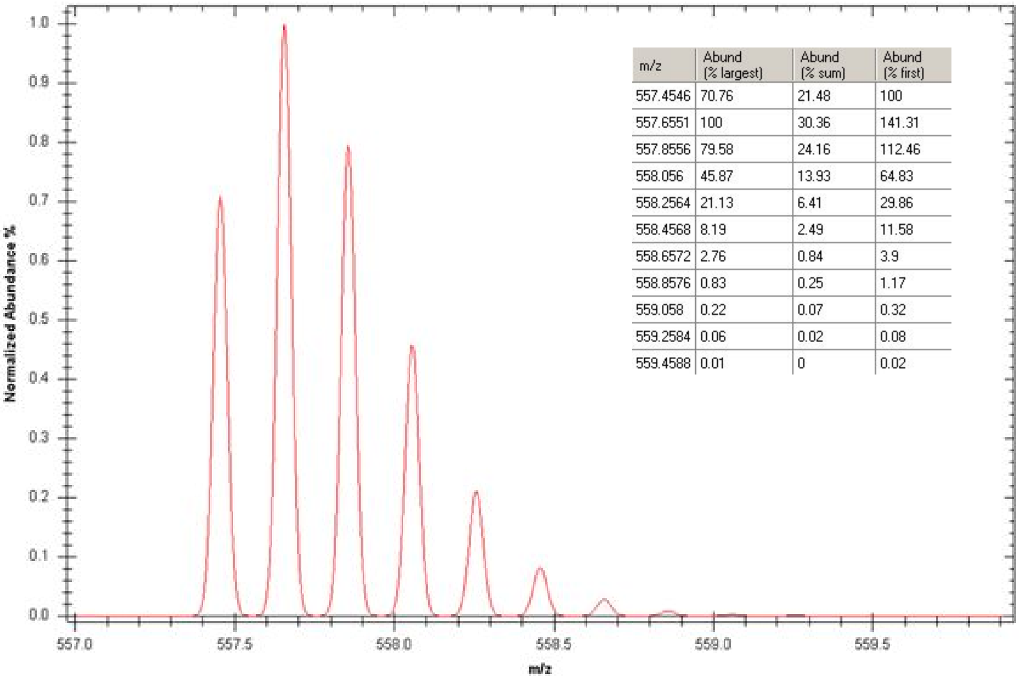


| m/z | Abund (% largest) | Abund (% sum) | Abund (% first) |
|---|---|---|---|
| 557.4546 | 70.76 | 21.48 | 100 |
| 557.6551 | 100 | 30.36 | 141.31 |
| 557.8556 | 79.58 | 24.16 | 112.46 |
| 558.056 | 45.87 | 13.93 | 64.83 |
| 558.2564 | 21.13 | 6.41 | 29.86 |
| 558.4568 | 8.19 | 2.49 | 11.58 |
| 558.6572 | 2.76 | 0.84 | 3.9 |
| 558.8576 | 0.83 | 0.25 | 1.17 |
| 559.058 | 0.22 | 0.07 | 0.32 |
| 559.2584 | 0.06 | 0.02 | 0.08 |
| 559.4588 | 0.01 | 0 | 0.02 |

## HRMS (exp) for $[M+5H]^{5+}$

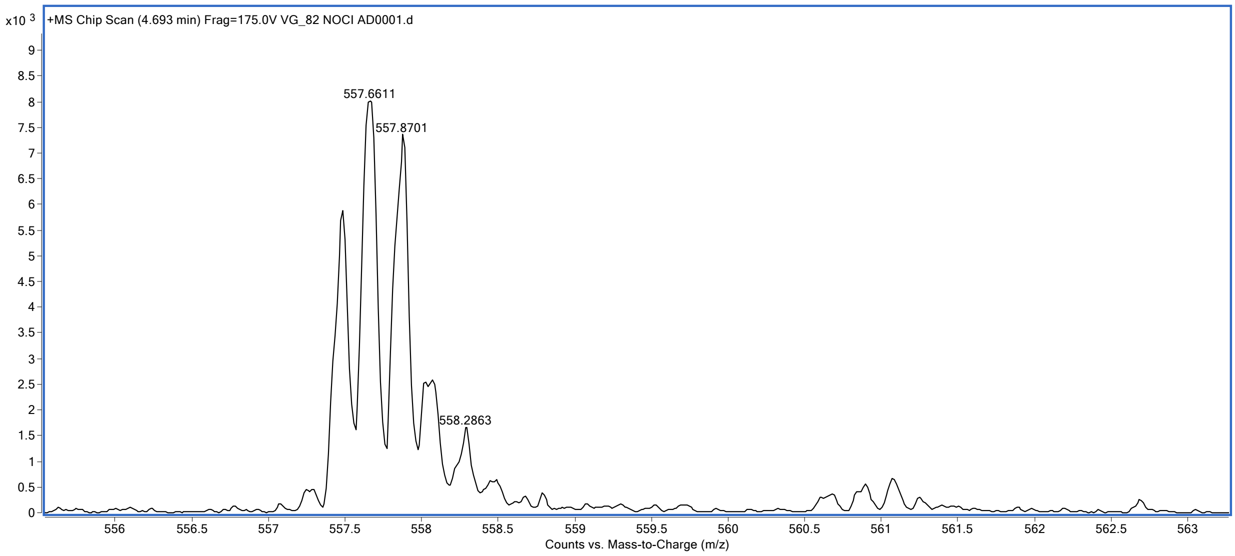

## Compound 45 (HRMS analysis)

## HRMS (teo) for $[M+4H]^{4+}$

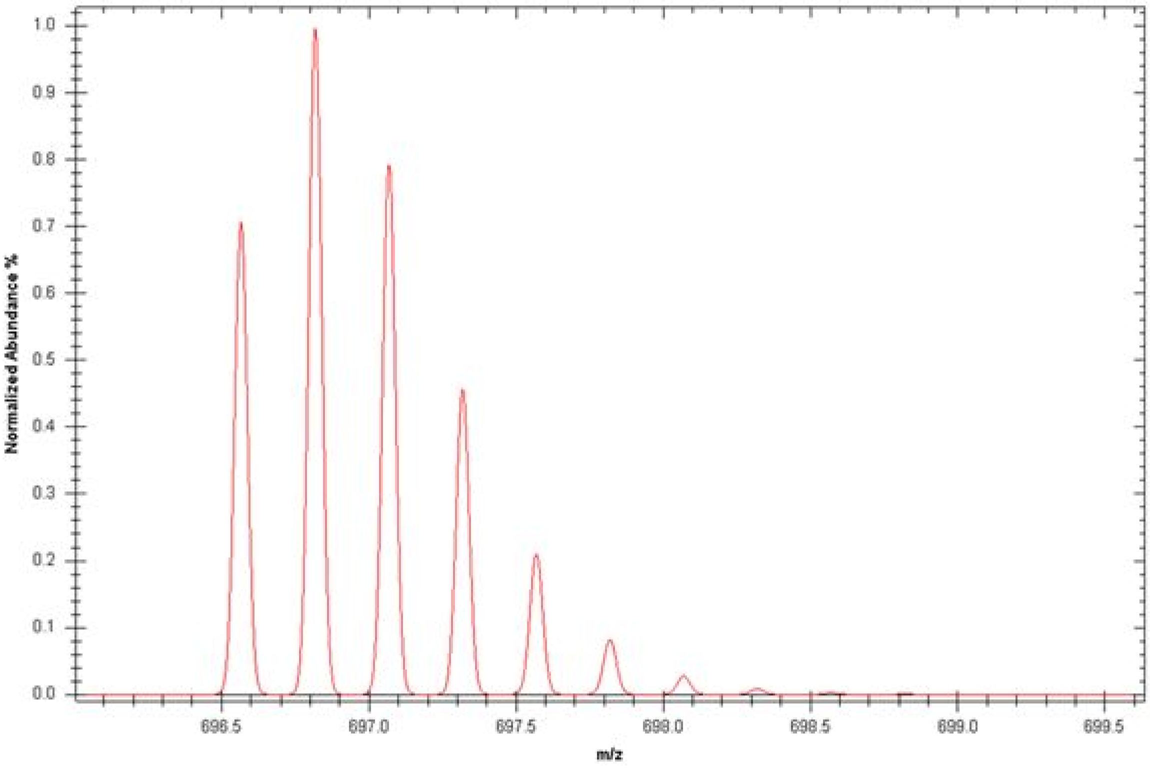


## HRMS (exp) for $[M+4H]^{4+}$

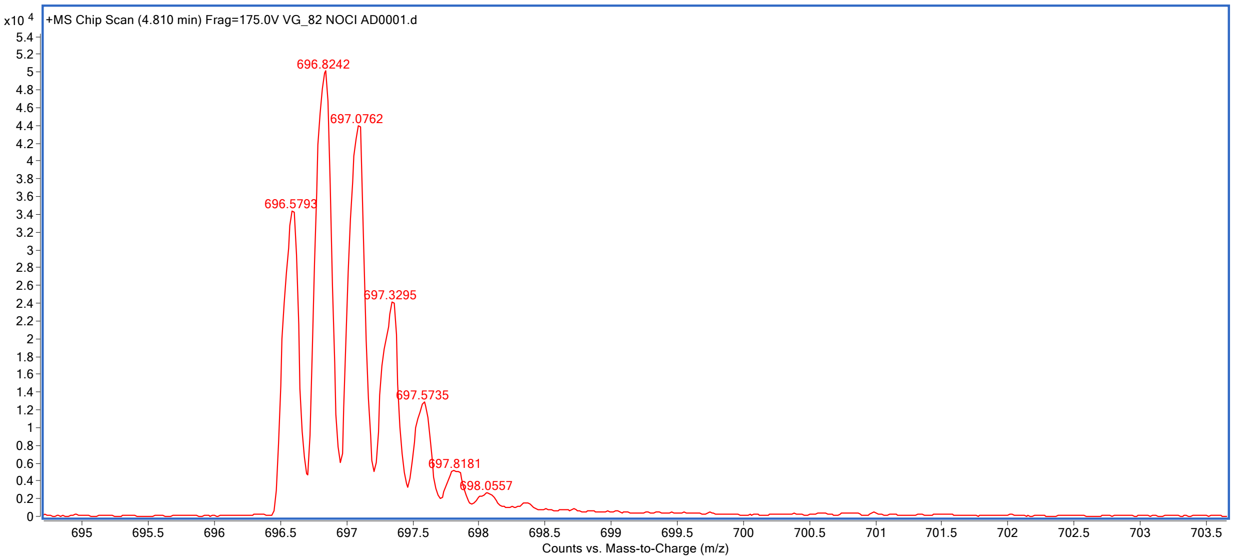

**Compound 46**

MS (ESI): m/z calcd for $C_{108}H_{163}N_{37}O_{43}S$ $[M+3H]^{3+}$ 900.92 found 901.06. $t_R$ = 12.25

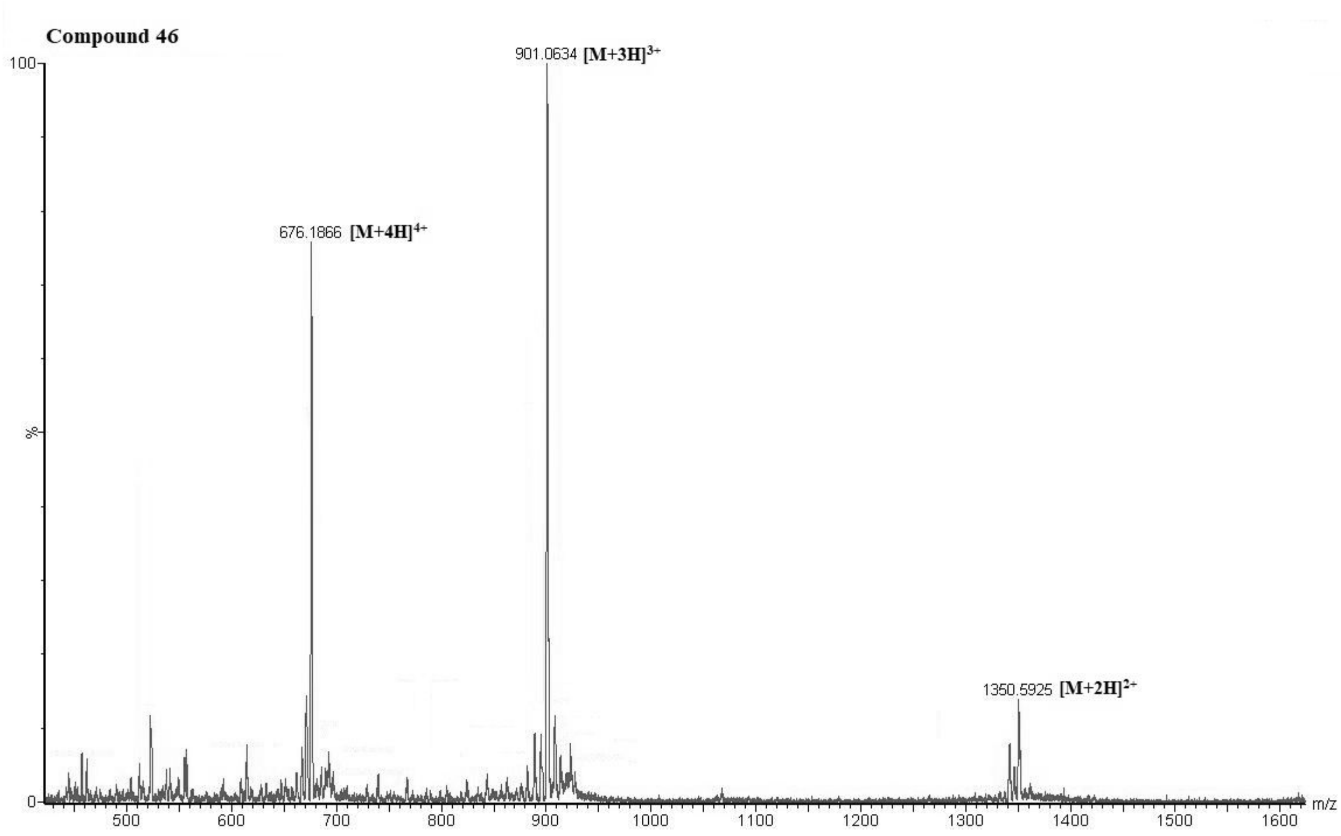


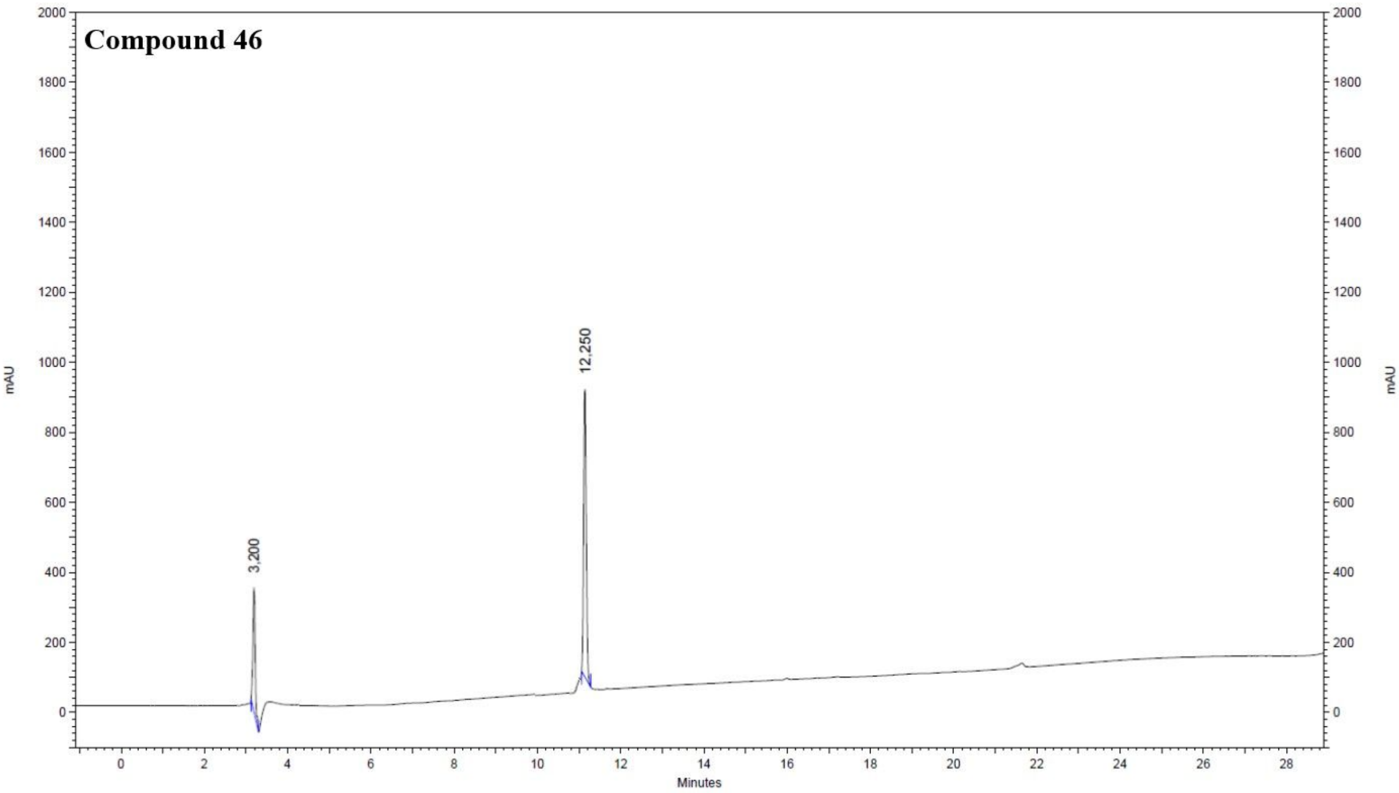

## Compound 46 (HRMS analysis)

## HRMS (teo) for $[M+5H]^{5+}$

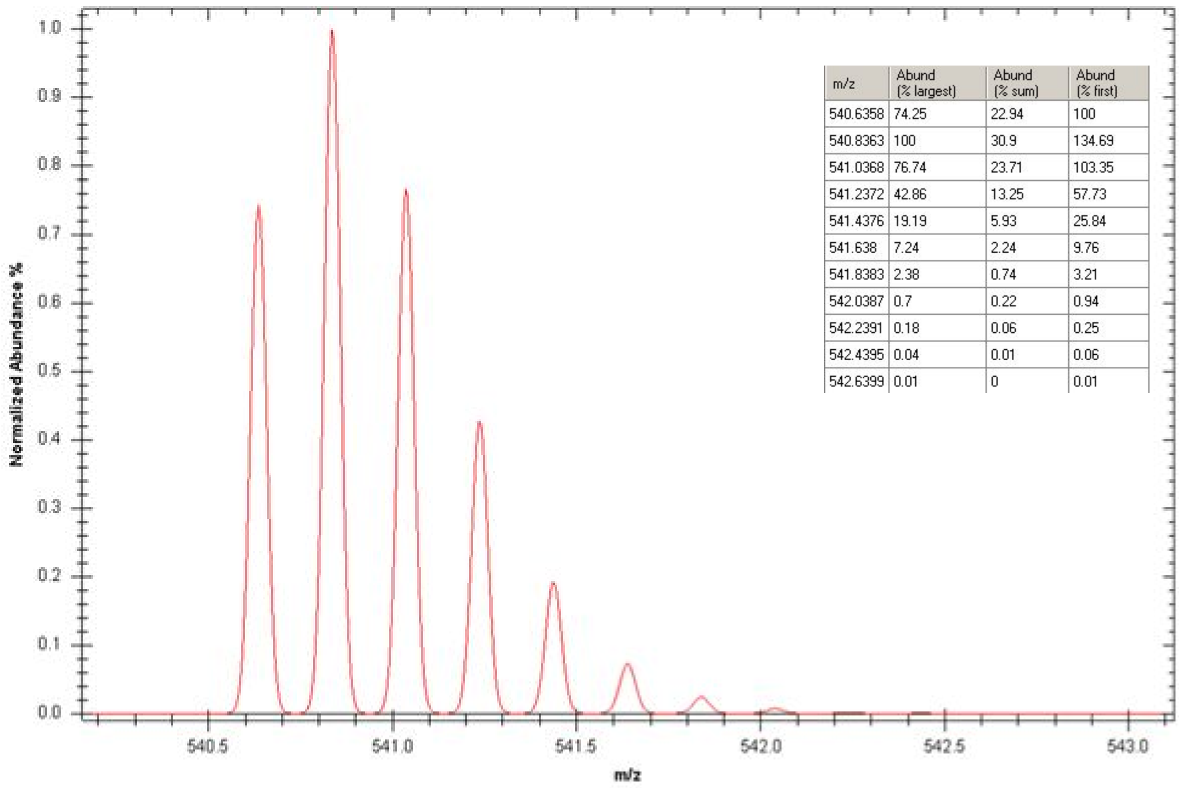


| m/z | Abund (% largest) | Abund (% sum) | Abund (% first) |
|---|---|---|---|
| 540.6358 | 74.25 | 22.94 | 100 |
| 540.8363 | 100 | 30.9 | 134.69 |
| 541.0368 | 76.74 | 23.71 | 103.35 |
| 541.2372 | 42.86 | 13.25 | 57.73 |
| 541.4376 | 19.19 | 5.93 | 25.84 |
| 541.638 | 7.24 | 2.24 | 9.76 |
| 541.8383 | 2.38 | 0.74 | 3.21 |
| 542.0387 | 0.7 | 0.22 | 0.94 |
| 542.2391 | 0.18 | 0.06 | 0.25 |
| 542.4395 | 0.04 | 0.01 | 0.06 |
| 542.6399 | 0.01 | 0 | 0.01 |

## HRMS (exp) for $[M+5H]^{5+}$

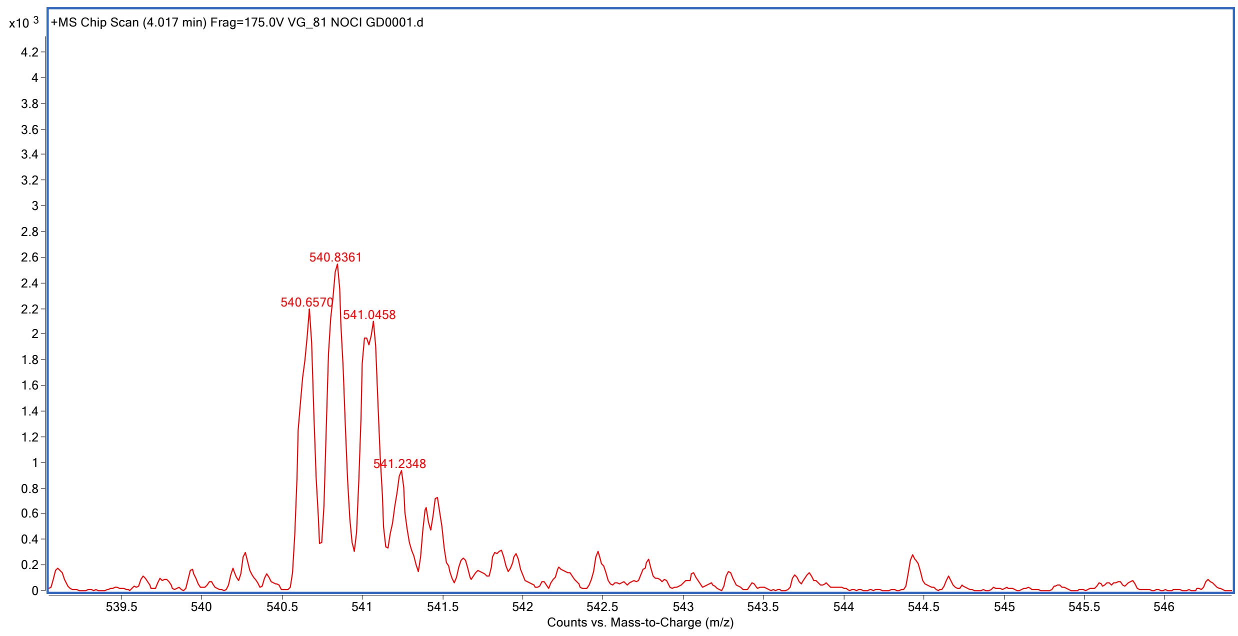

**Compound 47**

MS (ESI): m/z calcd for $C_{126}H_{187}N_{49}O_{31}S$ $[M+4H]^{4+}$ 730.06 found 730.18. $t_R$ = 11.57

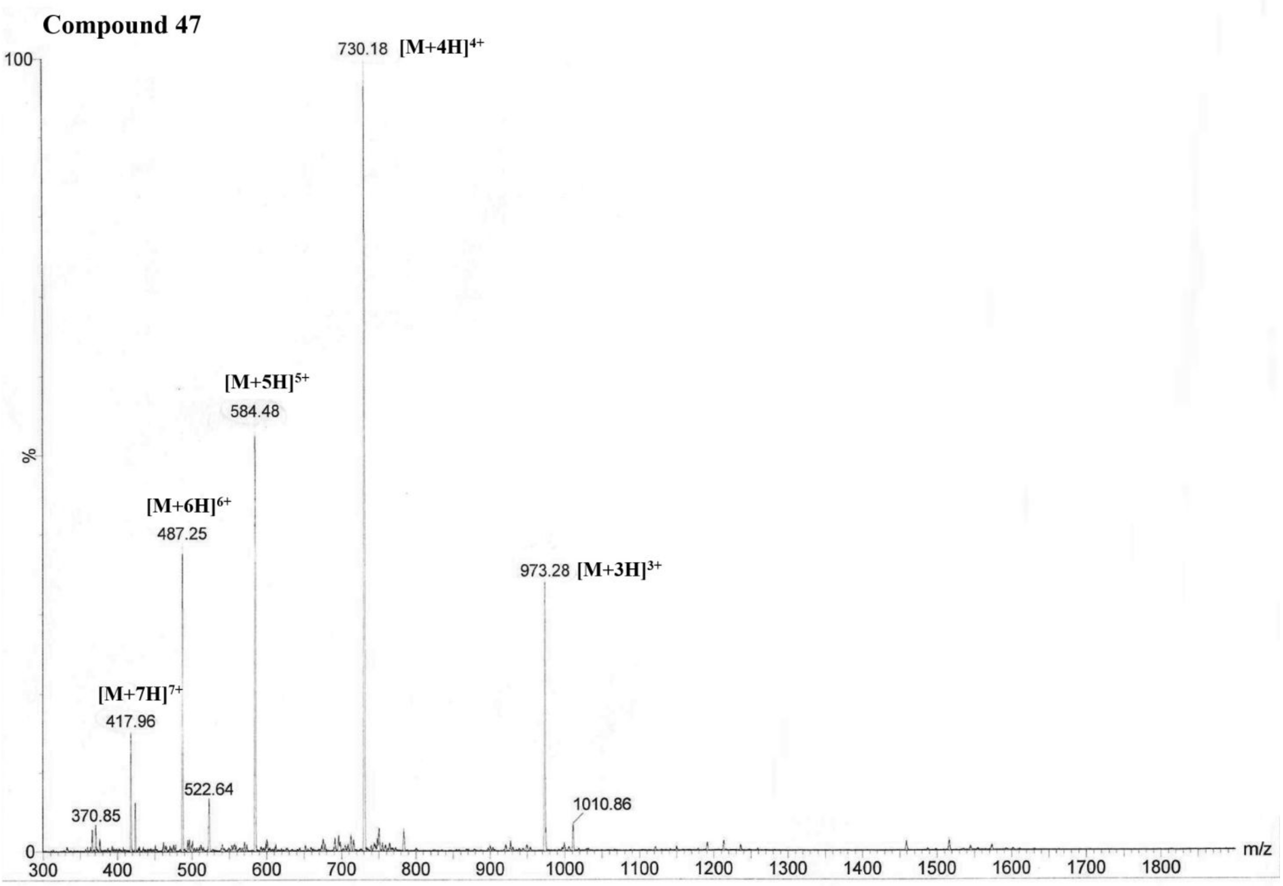


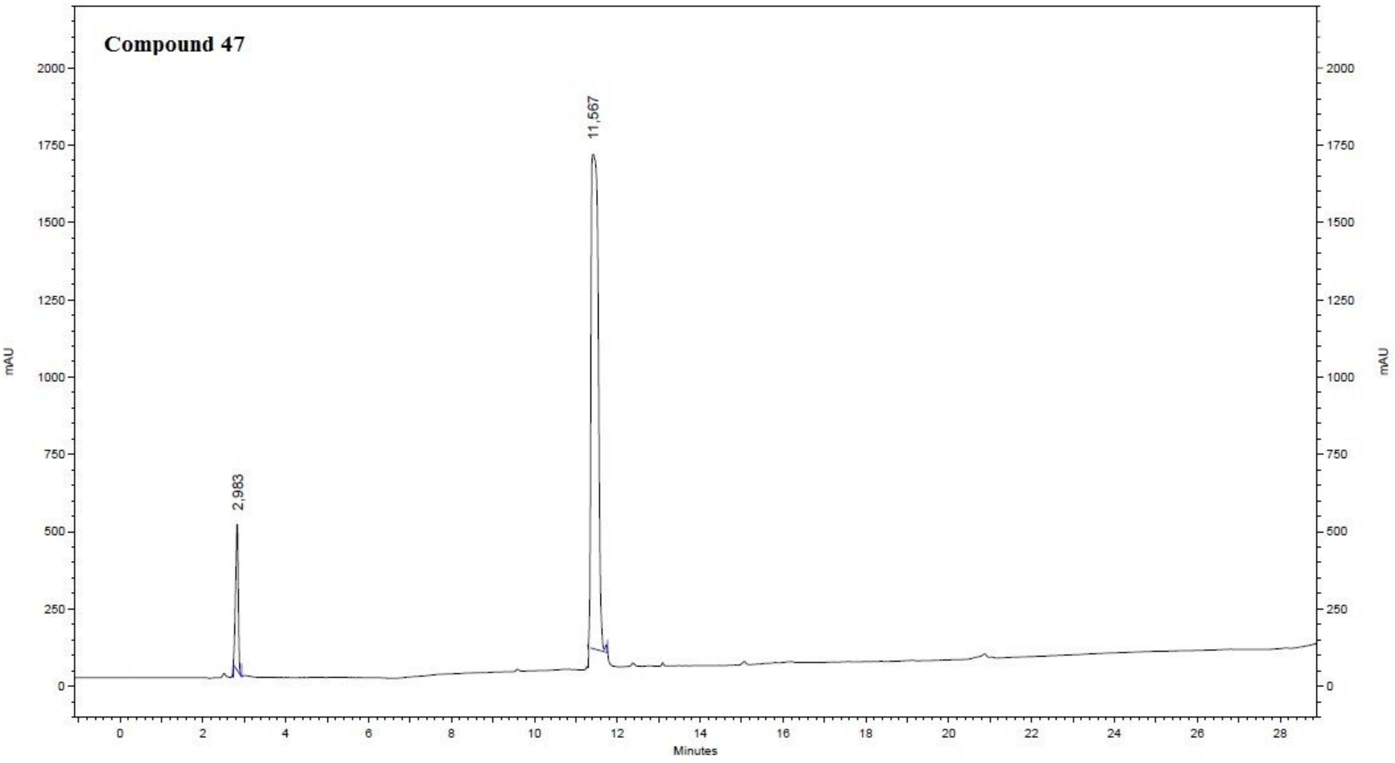

## Compound 47 (HRMS analysis)

## HRMS (teo) for $[M+6H]^{6+}$

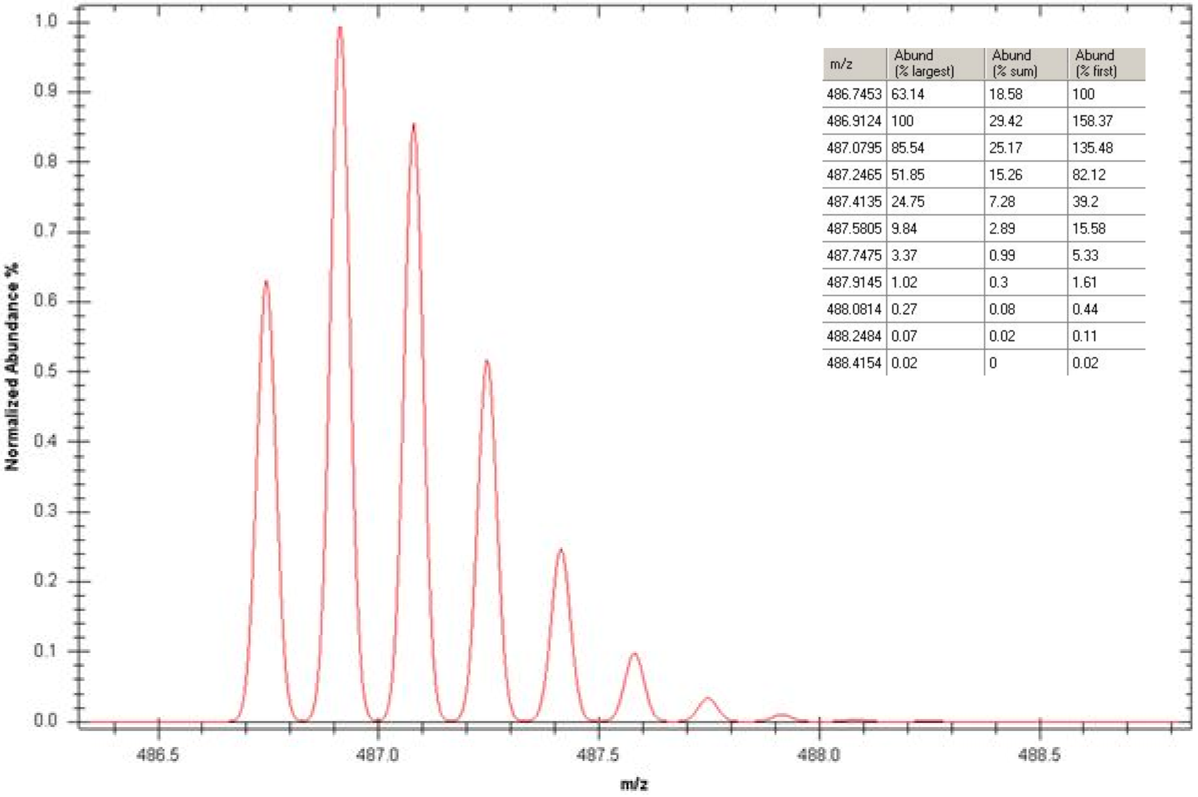


| m/z | Abund (% largest) | Abund (% sum) | Abund (% first) |
|---|---|---|---|
| 486.7453 | 63.14 | 18.58 | 100 |
| 486.9124 | 100 | 29.42 | 158.37 |
| 487.0795 | 85.54 | 25.17 | 135.48 |
| 487.2465 | 51.85 | 15.26 | 82.12 |
| 487.4135 | 24.75 | 7.28 | 39.2 |
| 487.5805 | 9.84 | 2.89 | 15.58 |
| 487.7475 | 3.37 | 0.99 | 5.33 |
| 487.9145 | 1.02 | 0.3 | 1.61 |
| 488.0814 | 0.27 | 0.08 | 0.44 |
| 488.2484 | 0.07 | 0.02 | 0.11 |
| 488.4154 | 0.02 | 0 | 0.02 |

## HRMS (exp) for $[M+6H]^{6+}$

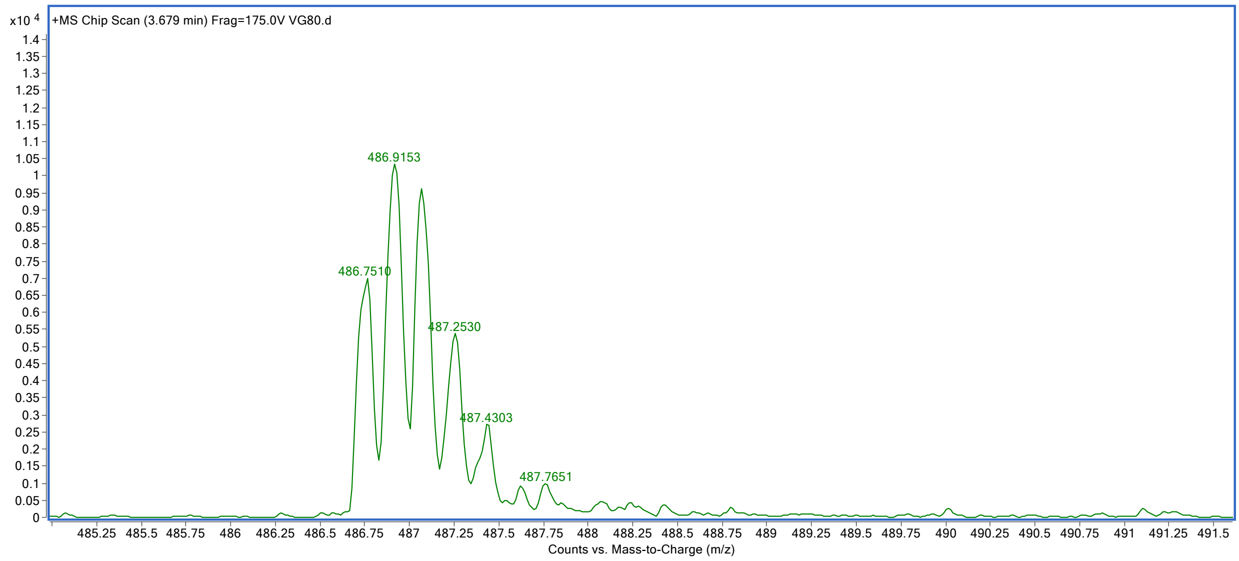

**Compound 48**

MS (ESI): m/z calcd for $C_{120}H_{175}N_{49}O_{31}S$ $[M+5H]^{5+}$ 567.42 found 567.56. $t_R$ = 11.38

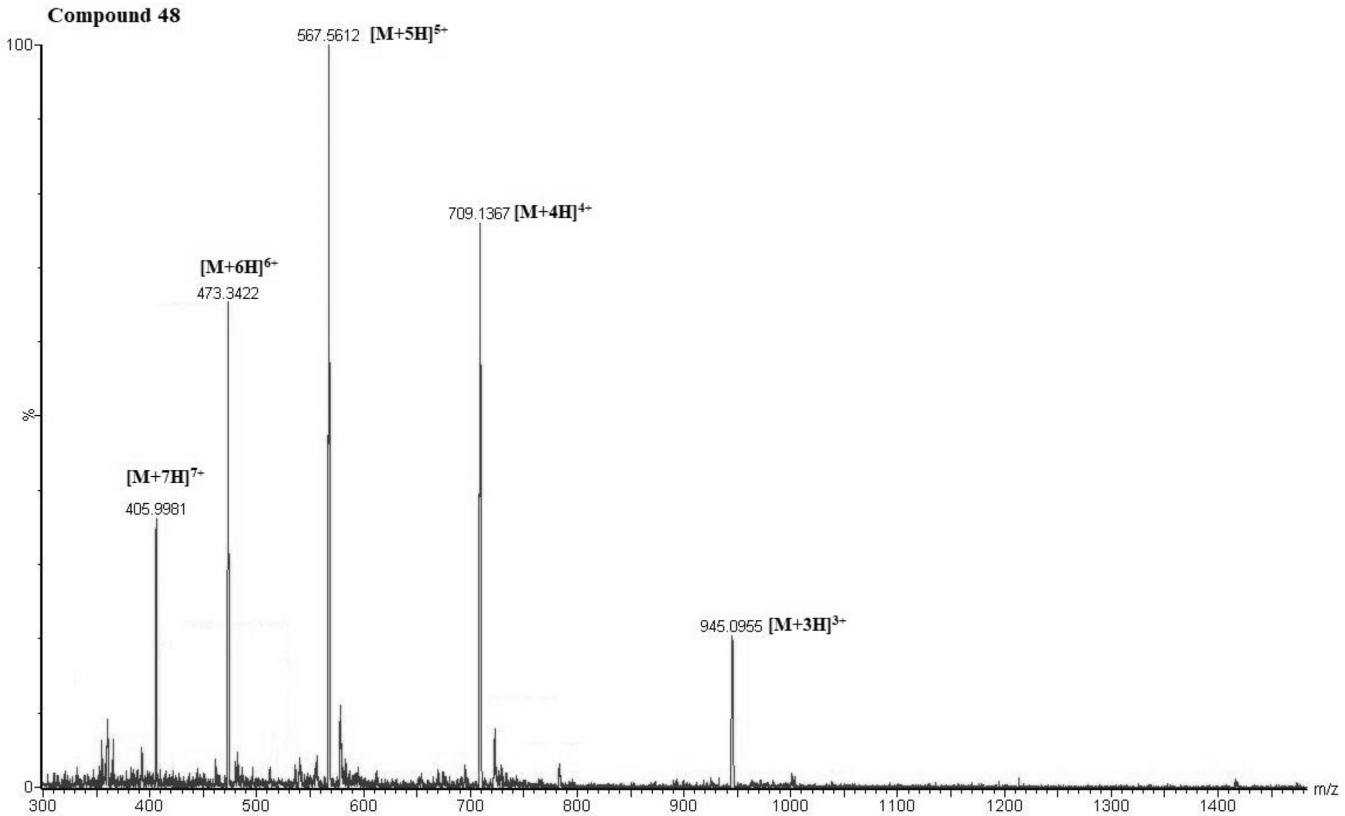


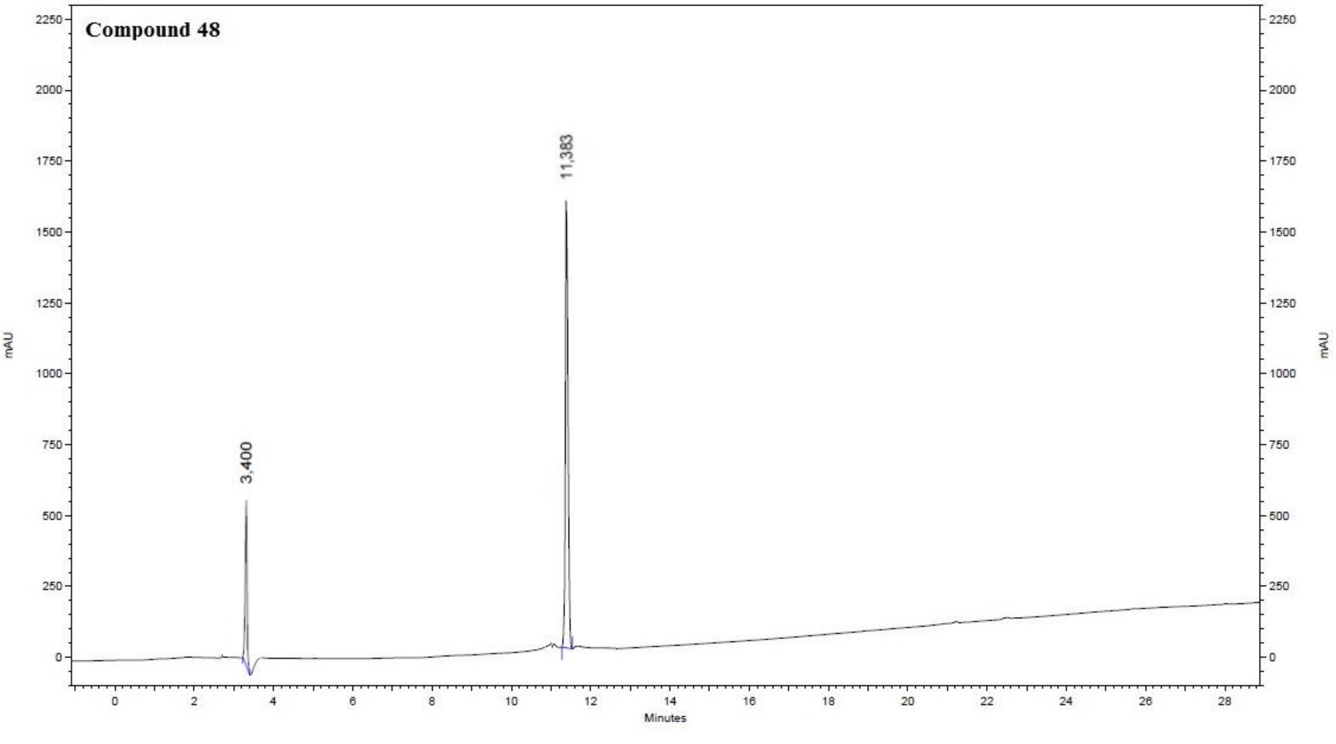

**Compound 48 (HRMS analysis)**

| molecular formula | exact mass | | |
|---|---|---|---|
| $C_{89}H_{149}N_{25}O_{19}S$ | 2830,33443 | | |
| molecular ion | m/z (teo) | m/z (exp) | dm/z (ppm) |
| $[M+H]^{+}$ | 2831,34171 | - | - |
| $[M+2H]^{2+}$ | 1416,17449 | 1416,1739 | 0,4 |
| $[M+3H]^{3+}$ | 944,45209 | 944,4510 | 1,2 |
| $[M+4H]^{4+}$ | 708,59088 | 708,5902 | 1,0 |
| $[M+5H]^{5+}$ | 567,07416 | 567,0741 | 0,1 |
| $[M+6H]^{6+}$ | 472,72968 | 472,7306 | -1,9 |
| $[M+7H]^{7+}$ | 405,34077 | 405,3408 | -0,1 |

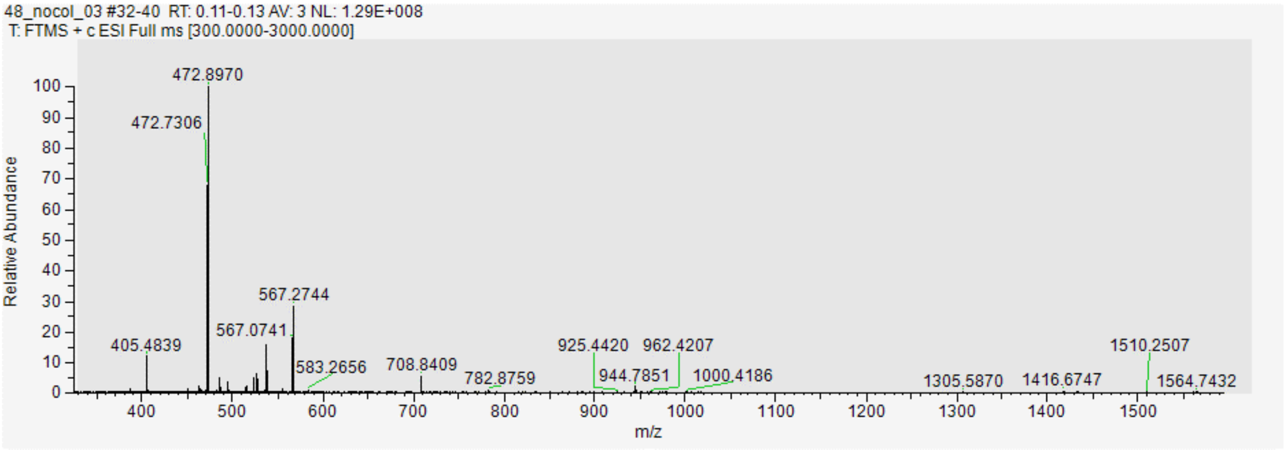


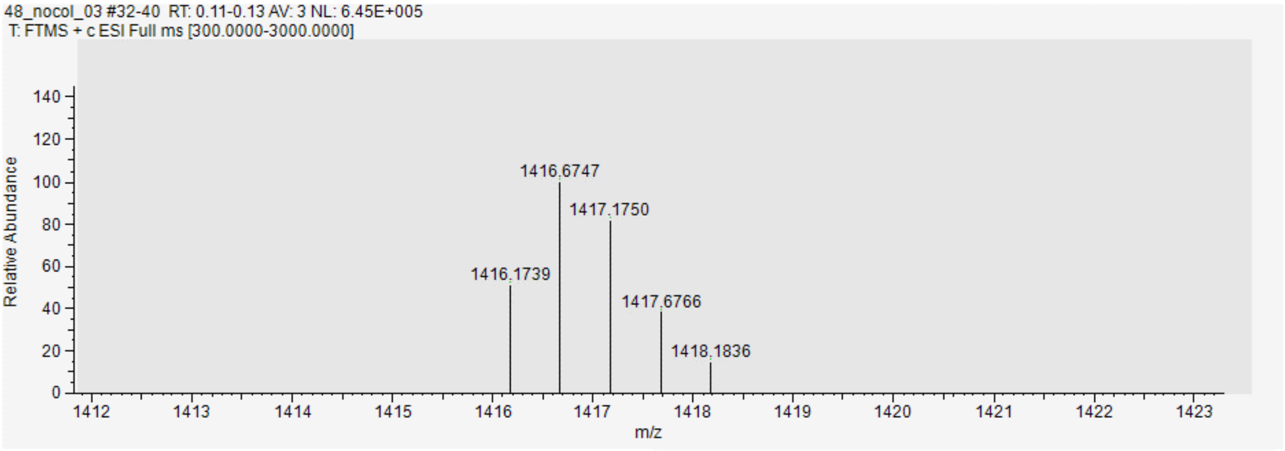

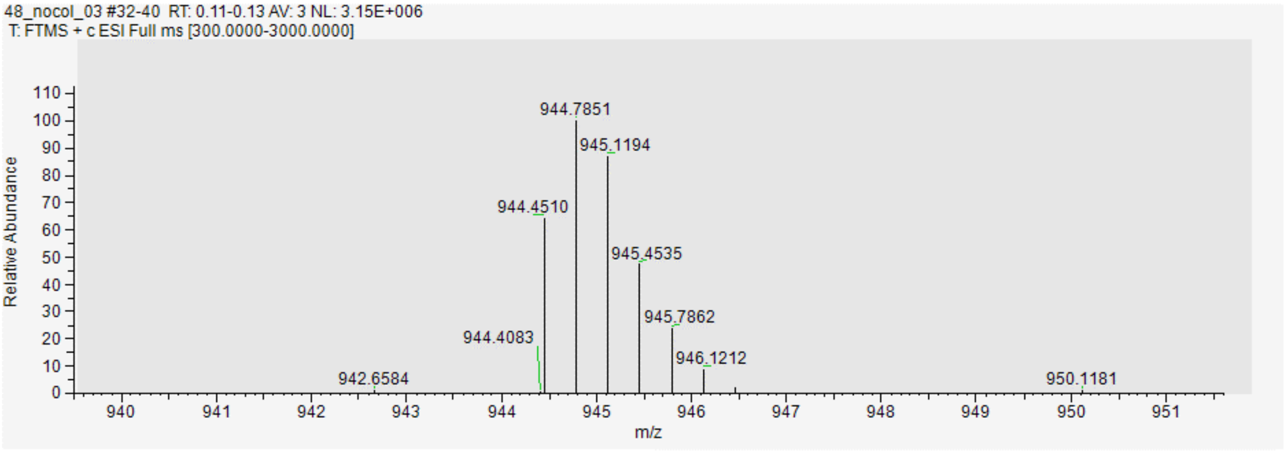
48_nocol_03 #32-40 RT: 0.11-0.13 AV: 3 NL: 3.15E+006
T: FTMS + c ESI Full ms [300.0000-3000.0000]
Relative Abundance
m/z
944.7851
945.1194
944.4510
945.4535
945.7862
944.4083
946.1212
942.6584
950.1181

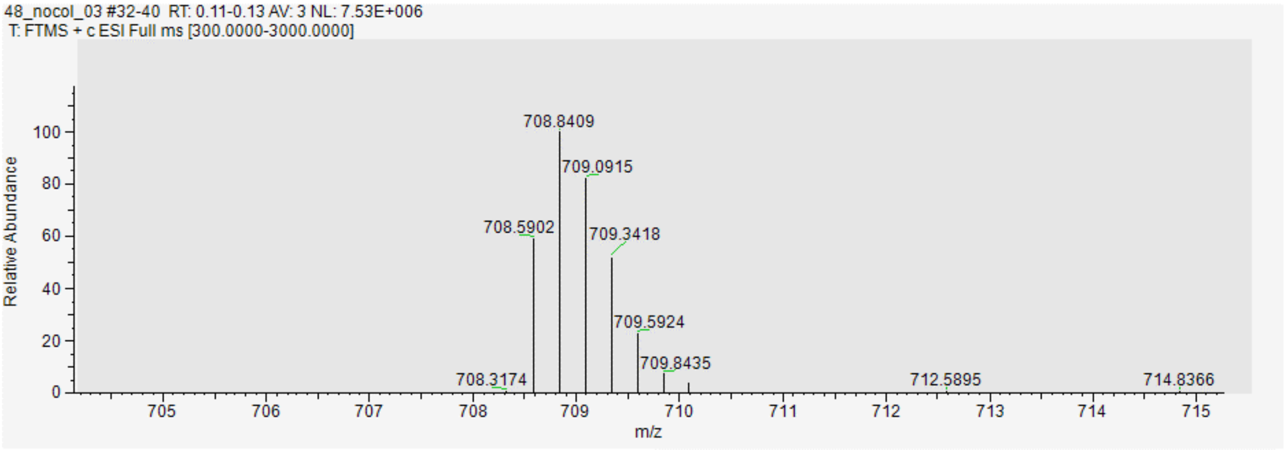
48_nocol_03 #32-40 RT: 0.11-0.13 AV: 3 NL: 7.53E+006
T: FTMS + c ESI Full ms [300.0000-3000.0000]
Relative Abundance
m/z
708.8409
709.0915
708.5902
709.3418
709.5924
709.8435
708.3174
712.5895
714.8366

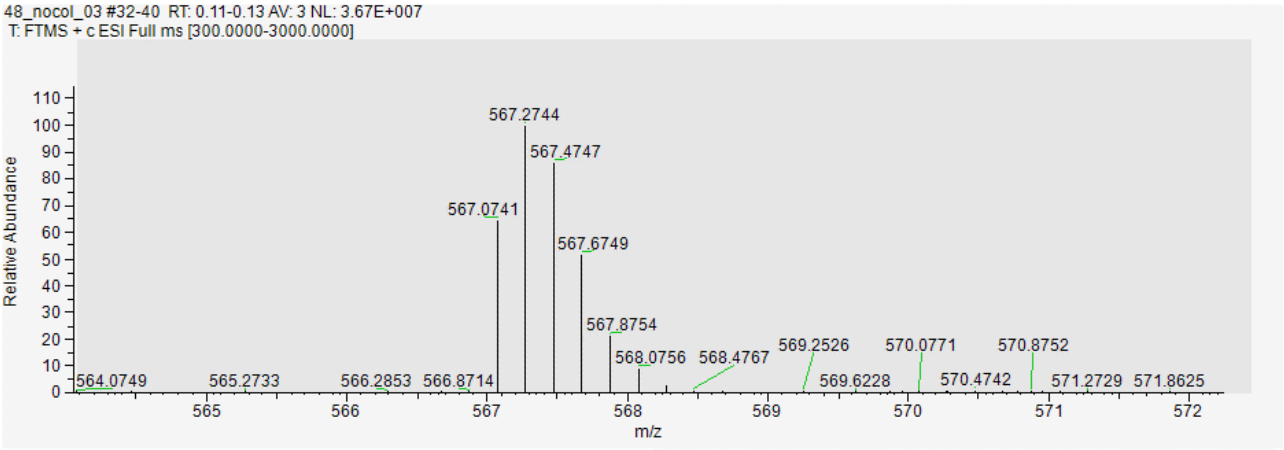
48_nocol_03 #32-40 RT: 0.11-0.13 AV: 3 NL: 3.67E+007
T: FTMS + c ESI Full ms [300.0000-3000.0000]
Relative Abundance
m/z
567.2744
567.4747
567.0741
567.6749
567.8754
568.0756
568.4767
569.2526
570.0771
570.8752
564.0749
565.2733
566.2853
566.8714
569.6228
570.4742
571.2729
571.8625

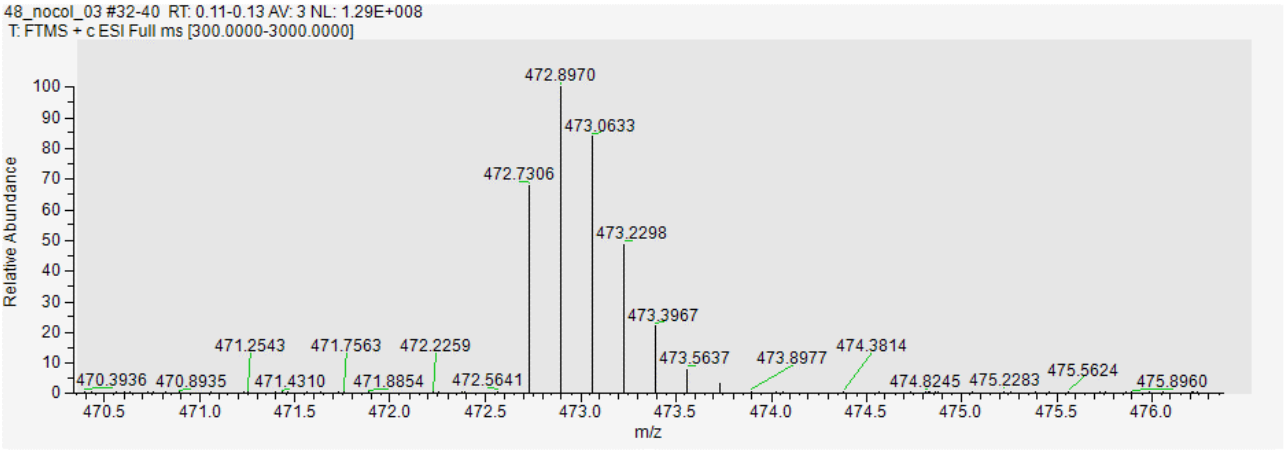
48_nocol_03 #32-40 RT: 0.11-0.13 AV: 3 NL: 1.29E+008
T: FTMS + c ESI Full ms [300.0000-3000.0000]
Relative Abundance
m/z
472.8970
473.0633
472.7306
473.2298
473.3967
471.2543
471.7563
472.2259
474.3814
473.5637
473.8977
470.3936
470.8935
471.4310
471.8854
472.5641
474.8245
475.2283
475.5624
475.8960

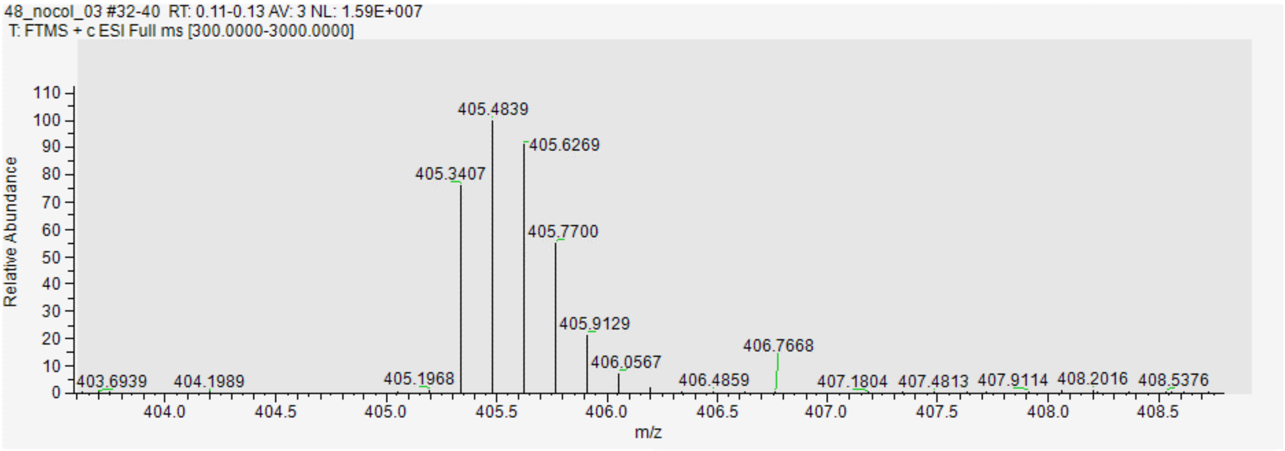

48_nocol_03 #32-40 RT: 0.11-0.13 AV: 3 NL: 1.59E+007
T: FTMS + c ESI Full ms [300.0000-3000.0000]
Relative Abundance
m/z
405.4839
405.6269
405.3407
405.7700
405.9129
406.0567
406.7668
403.6939
404.1989
405.1968
406.4859
407.1804
407.4813
407.9114
408.2016
408.5376

**Compound 49**

MS (ESI): m/z calcd for $C_{108}H_{187}N_{37}O_{25}S$ $[M+4H]^{4+}$ 609.99 found 610.23. $t_R$ = 13.28

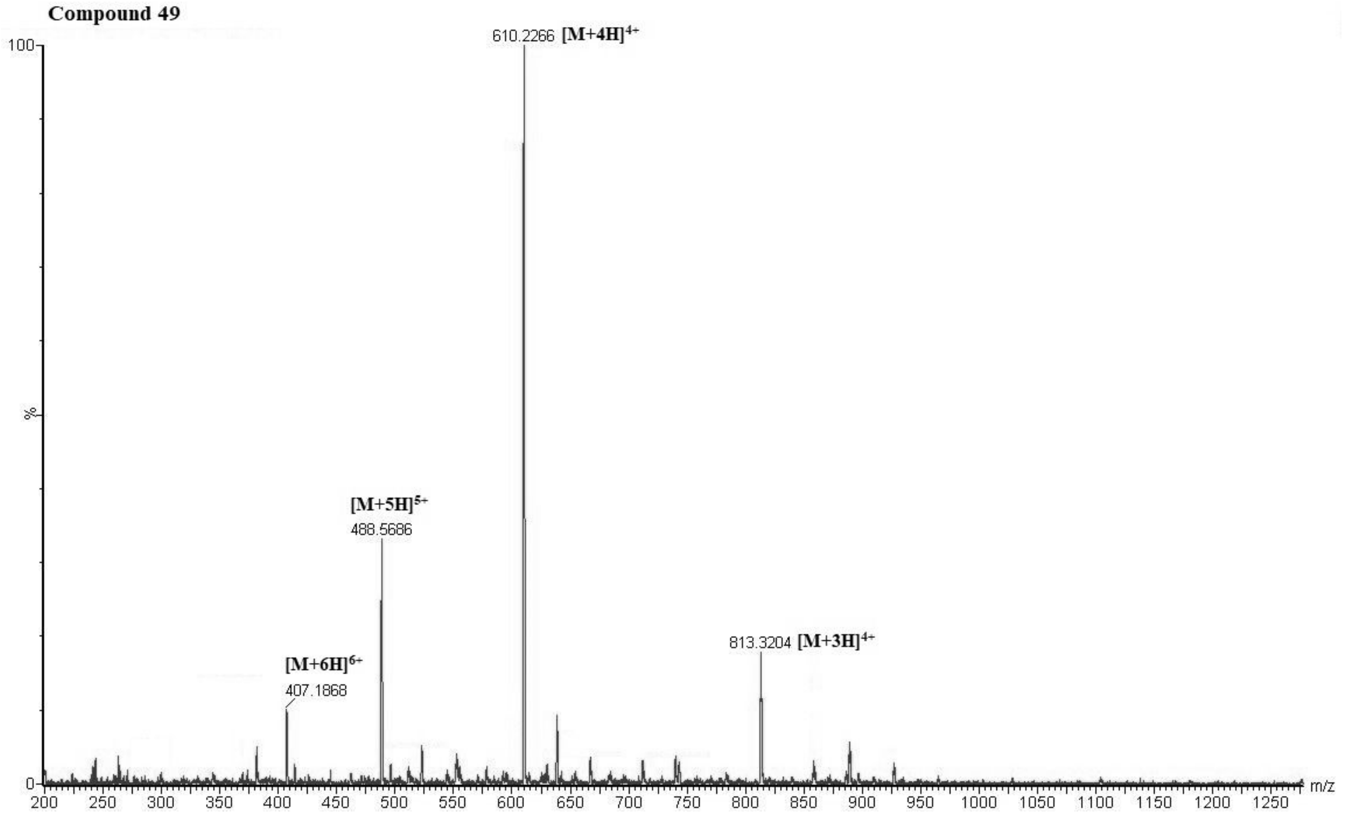


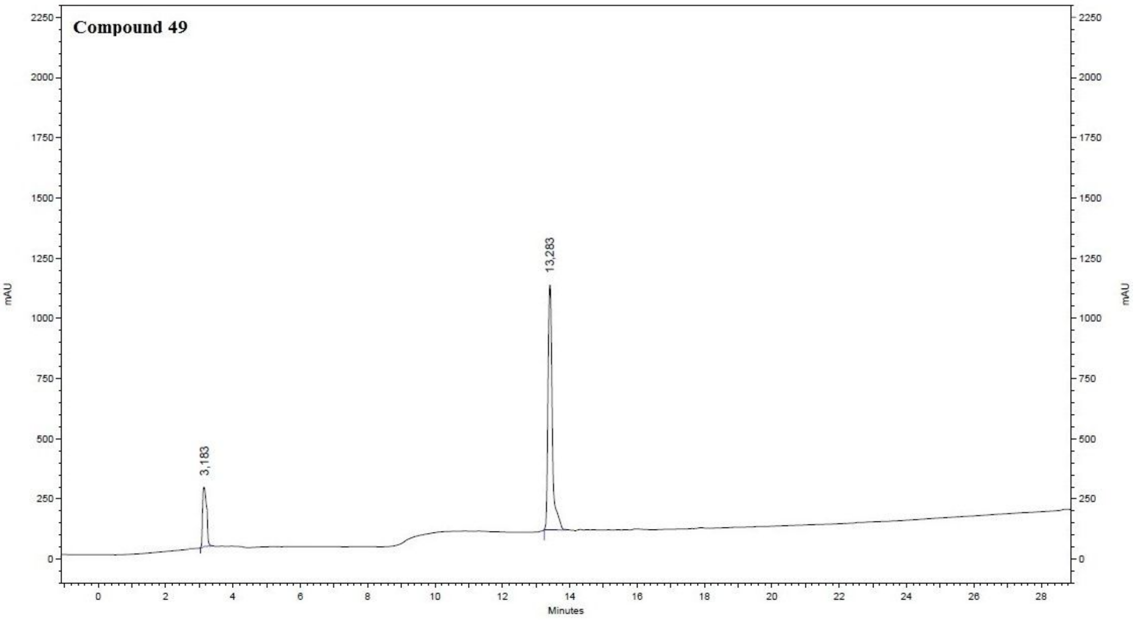

## Compound 49 (HRMS analysis)

| molecular formula | exact mass | | |
|---|---|---|---|
| $C_{108}H_{127}N_{37}O_{25}S$ | 2434,42195 | | |
| molecular ion | m/z (teo) | m/z (exp) | dm/z (ppm) |
| $[M+H]^{+}$ | 2435,42923 | - | - |
| $[M+2H]^{2+}$ | 1218,21825 | 1218,2174 | 0,7 |
| $[M+3H]^{3+}$ | 812,48126 | 812,4800 | 1,6 |
| $[M+4H]^{4+}$ | 609,61276 | 609,6122 | 0,9 |
| $[M+5H]^{5+}$ | 487,89167 | 487,8925 | -1,7 |
| $[M+6H]^{6+}$ | 406,74427 | 406,7442 | 0,2 |

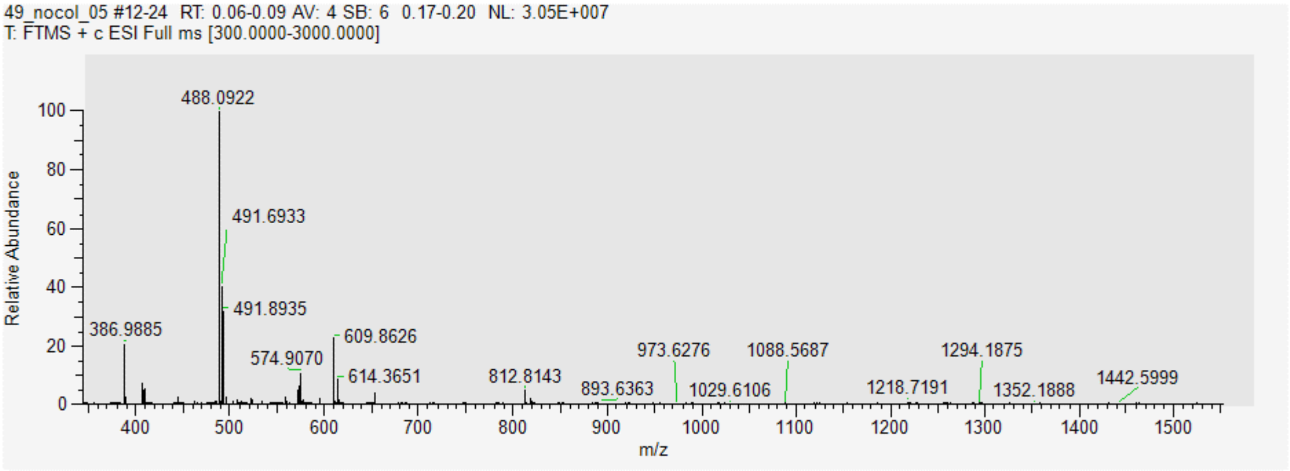


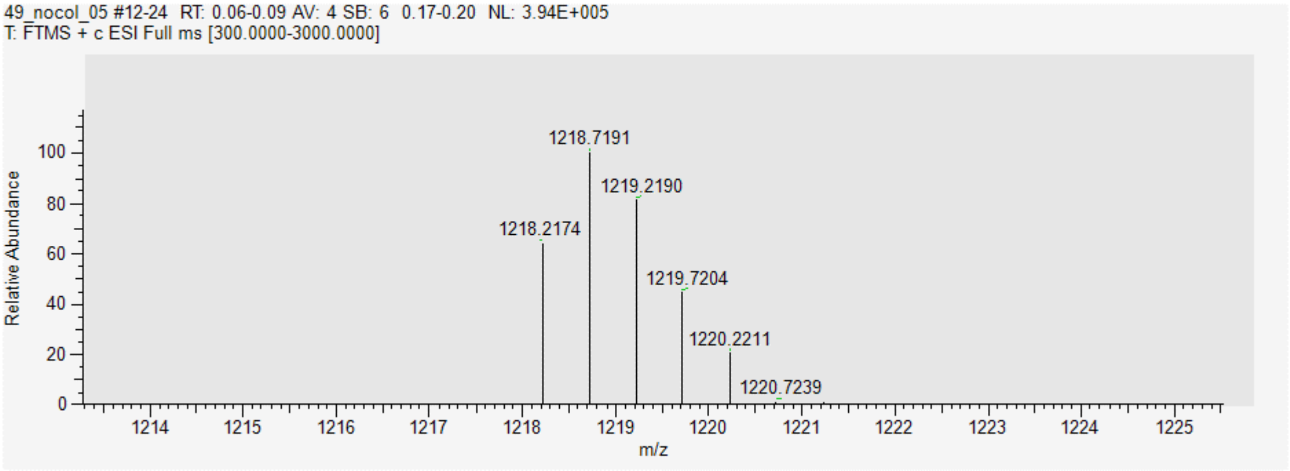

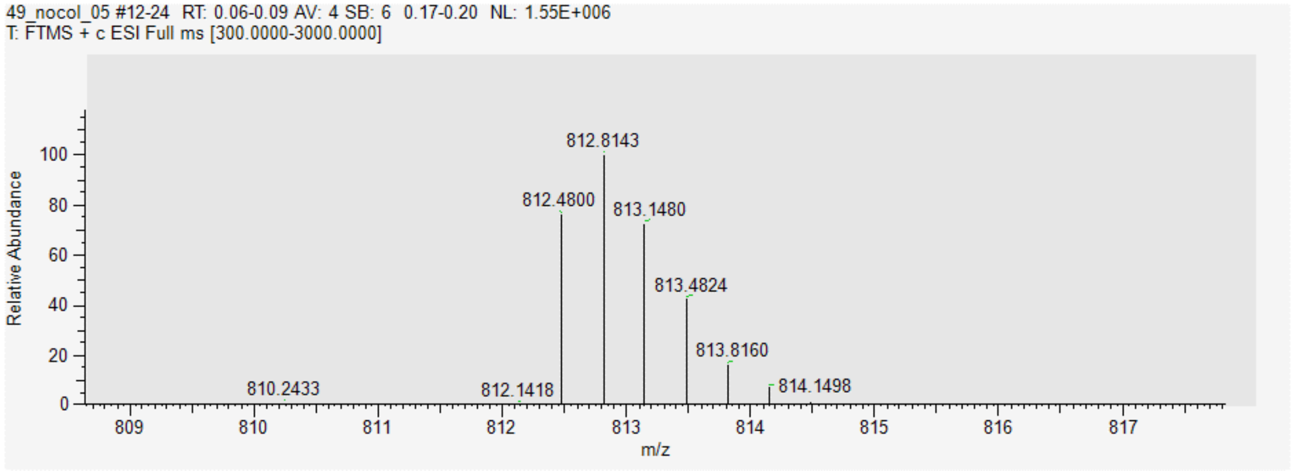
49_nocol_05 #12-24 RT: 0.06-0.09 AV: 4 SB: 6 0.17-0.20 NL: 1.55E+006
T: FTMS + c ESI Full ms [300.0000-3000.0000]
Relative Abundance
m/z
812.8143
812.4800
813.1480
813.4824
813.8160
814.1498
810.2433
812.1418

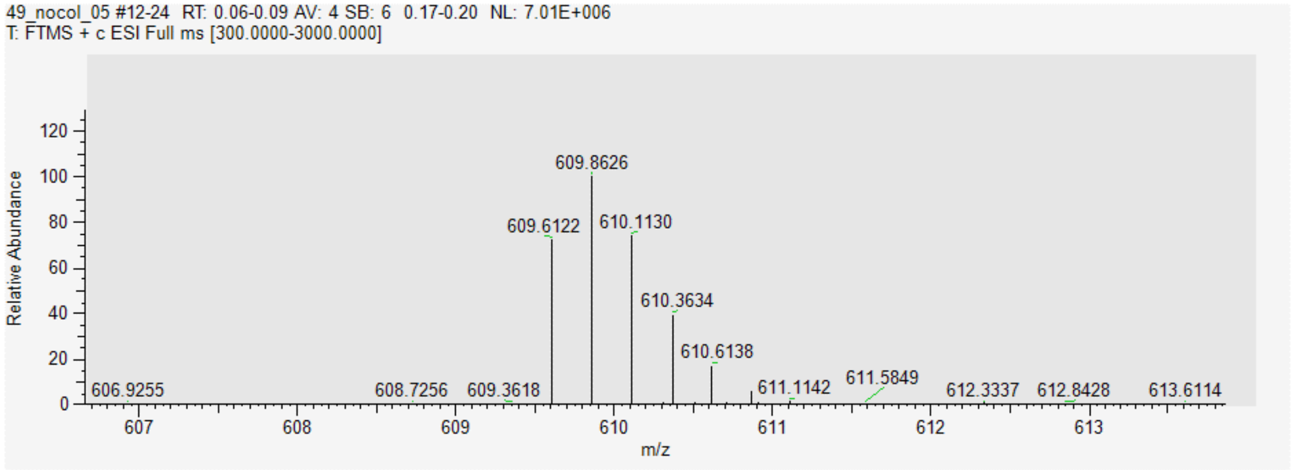
49_nocol_05 #12-24 RT: 0.06-0.09 AV: 4 SB: 6 0.17-0.20 NL: 7.01E+006
T: FTMS + c ESI Full ms [300.0000-3000.0000]
Relative Abundance
m/z
609.8626
609.6122
610.1130
610.3634
610.6138
606.9255
608.7256
609.3618
611.1142
611.5849
612.3337
612.8428
613.6114

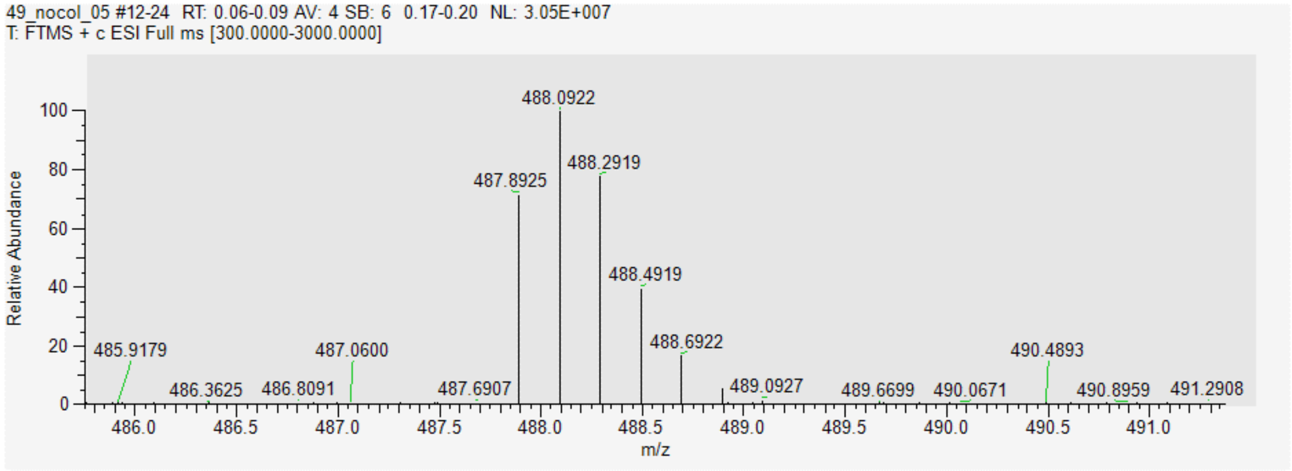
49_nocol_05 #12-24 RT: 0.06-0.09 AV: 4 SB: 6 0.17-0.20 NL: 3.05E+007
T: FTMS + c ESI Full ms [300.0000-3000.0000]
Relative Abundance
m/z
488.0922
488.2919
487.8925
488.4919
488.6922
485.9179
487.0600
490.4893
486.3625
486.8091
487.6907
489.0927
489.6699
490.0671
490.8959
491.2908

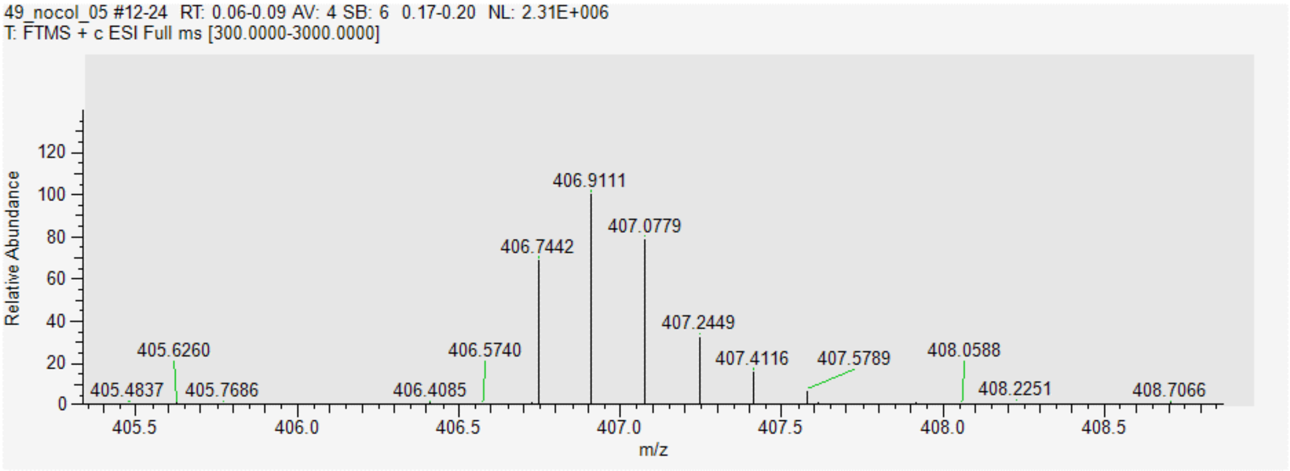
49_nocol_05 #12-24 RT: 0.06-0.09 AV: 4 SB: 6 0.17-0.20 NL: 2.31E+006
T: FTMS + c ESI Full ms [300.0000-3000.0000]
Relative Abundance
m/z
406.9111
407.0779
406.7442
407.2449
407.4116
407.5789
405.6260
406.5740
408.0588
405.4837
405.7686
406.4085
408.2251
408.7066

**Compound 50**

MS (ESI): m/z calcd for $C_{80}H_{129}N_{25}O_{19}S$ $[M+3H]^{3+}$ 593.37 found 593.59. $t_R$ = 13.75

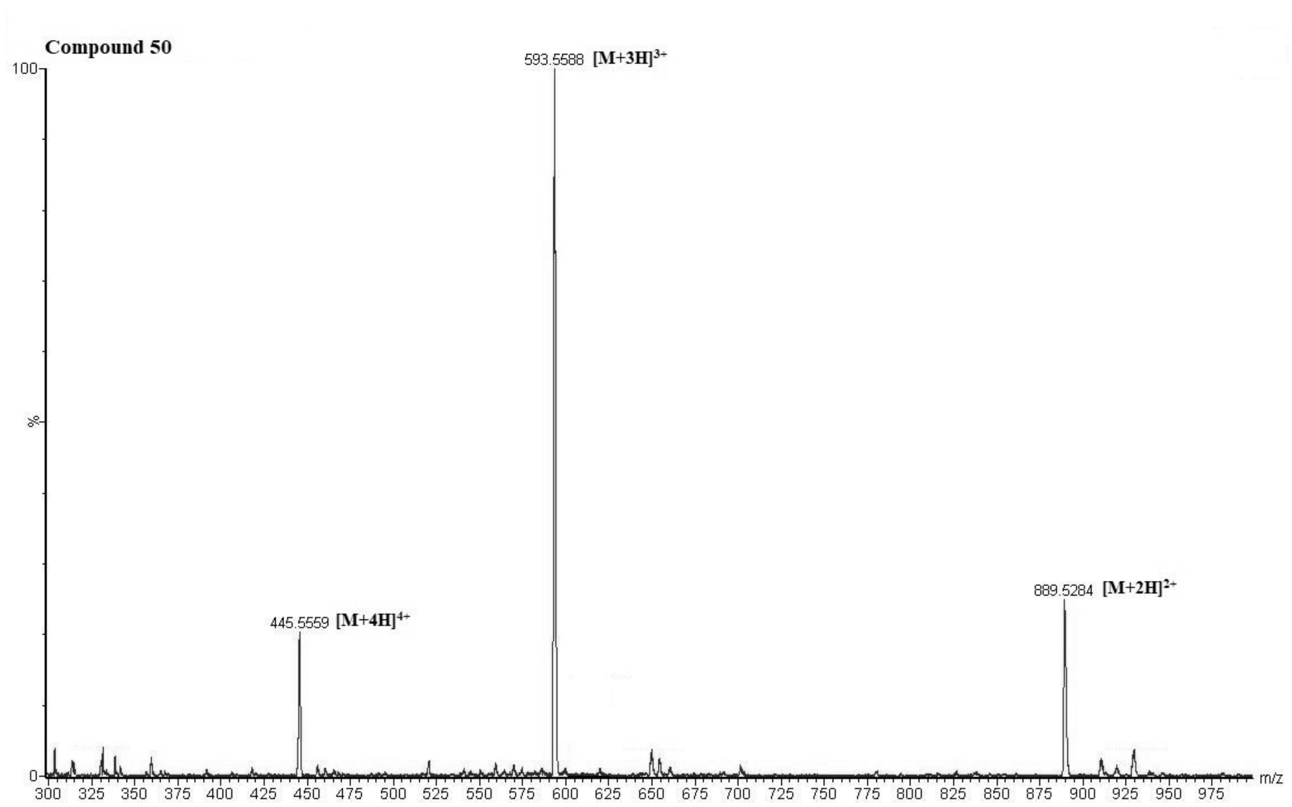


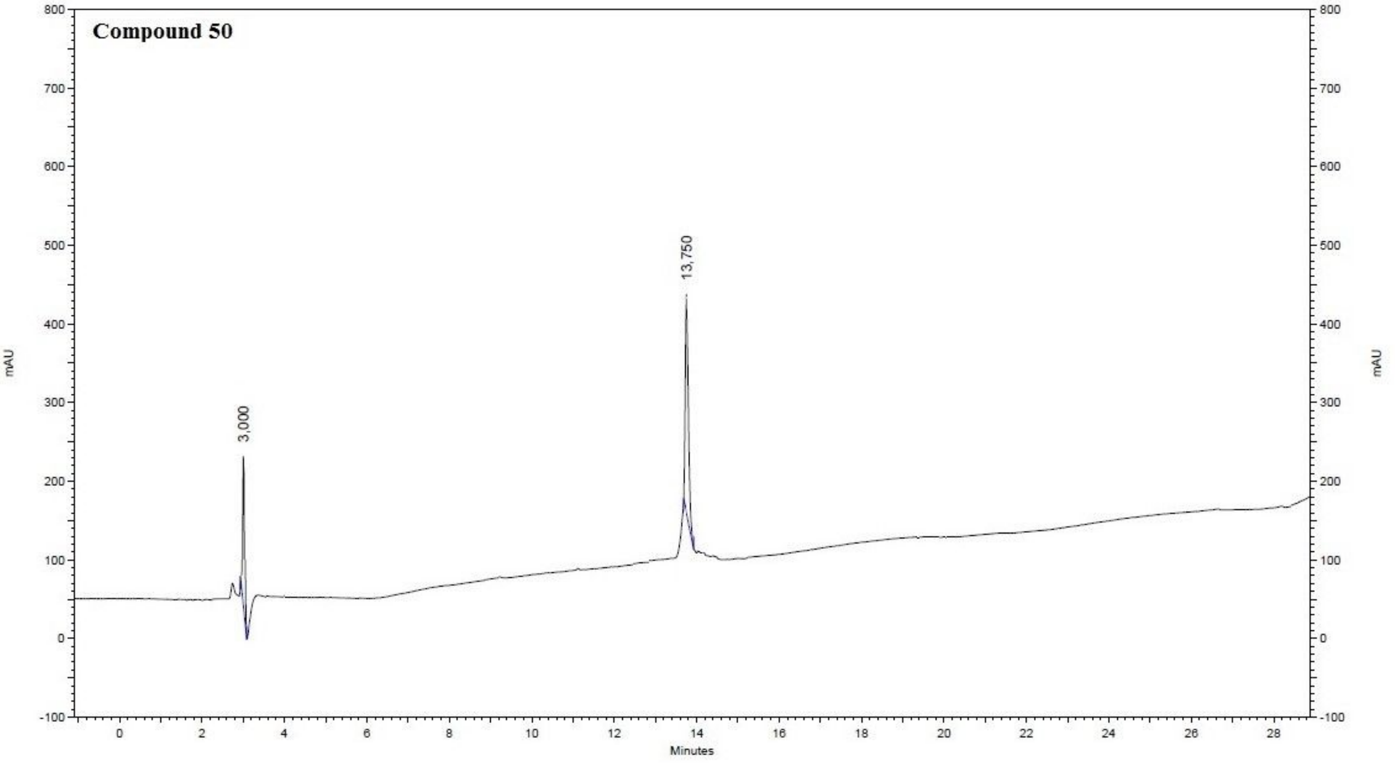

## Compound 50 (HRMS analysis)

| molecular formula | exact mass | | |
|---|---|---|---|
| $C_{80}H_{129}N_{25}O_{19}S$ | 1775,96173 | | |
| molecular ion | m/z (teo) | m/z (exp) | dm/z (ppm) |
| $[M+H]^{+}$ | 1776,96901 | - | - |
| $[M+2H]^{2+}$ | 888,98814 | 888,9871 | 1,2 |
| $[M+3H]^{3+}$ | 592,99452 | 592,9945 | 0,0 |
| $[M+4H]^{4+}$ | 444,99771 | 444,9982 | -1,1 |

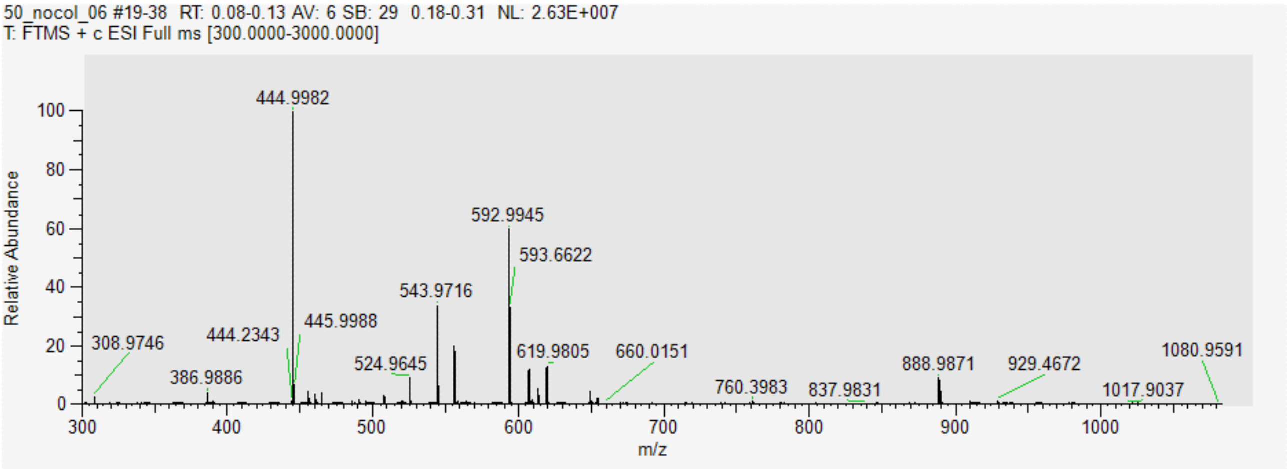


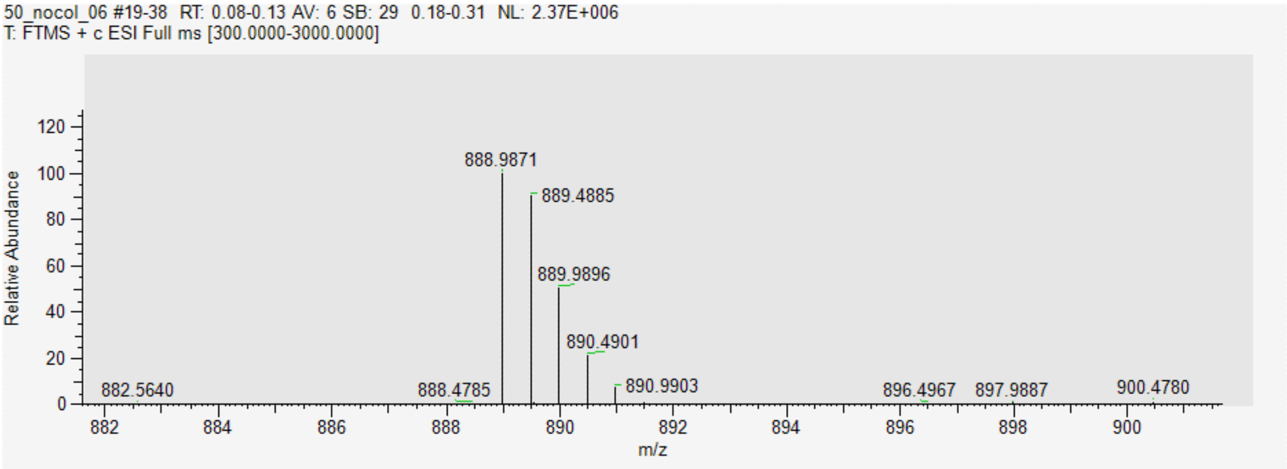


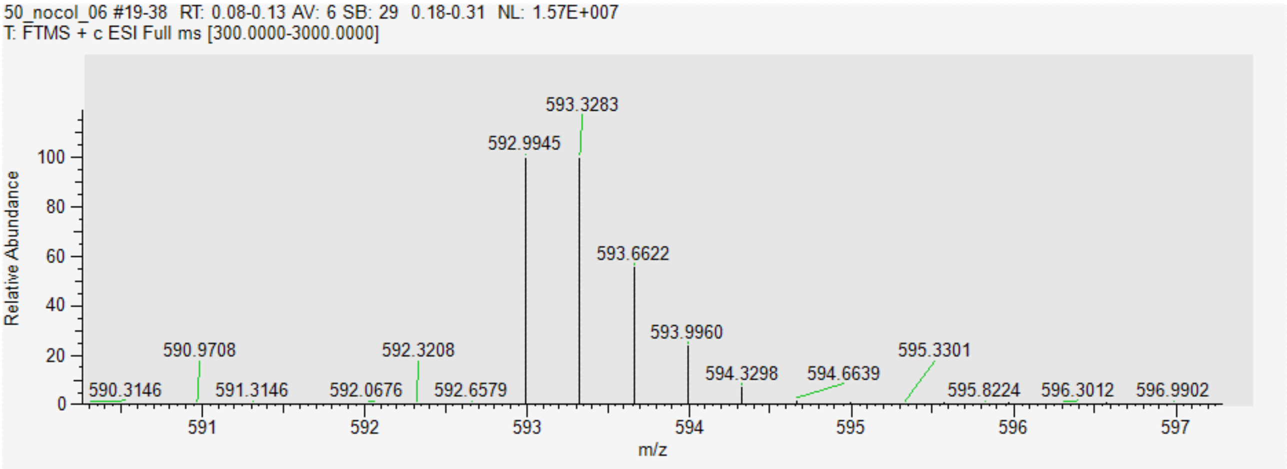

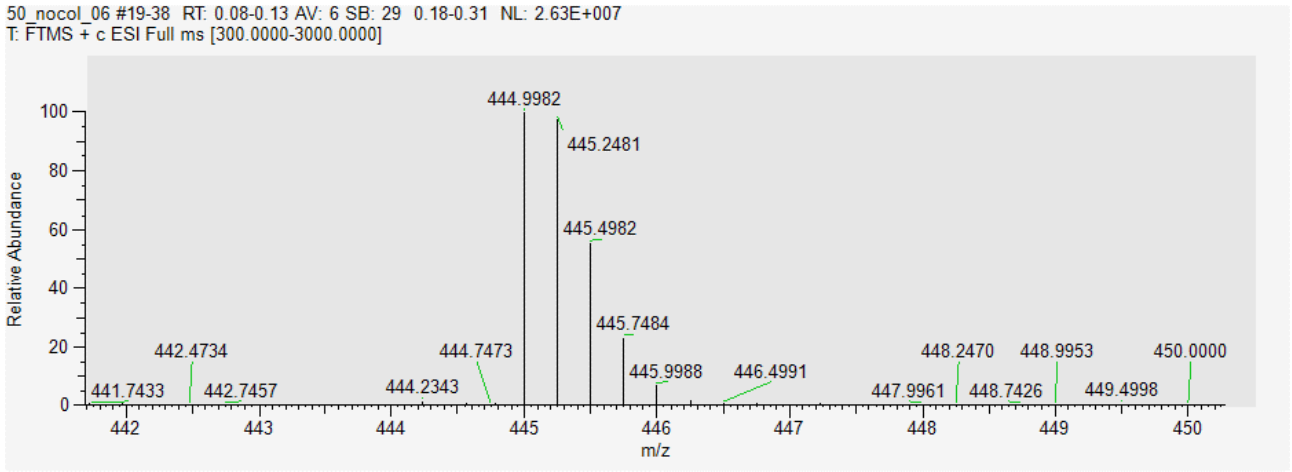
50_nocol_06 #19-38 RT: 0.08-0.13 AV: 6 SB: 29 0.18-0.31 NL: 2.63E+007
T: FTMS + c ESI Full ms [300.0000-3000.0000]
Relative Abundance
m/z
444.9982
445.2481
445.4982
445.7484
445.9988
446.4991
442.4734
441.7433
442.7457
444.2343
444.7473
447.9961
448.2470
448.7426
448.9953
449.4998
450.0000

## Compound 51

MS (ESI): m/z calcd for $C_{94}H_{157}N_{27}O_{21}S$ $[M+3H]^{3+}$ 678.84 found 679.43. $t_R$ = 15.25

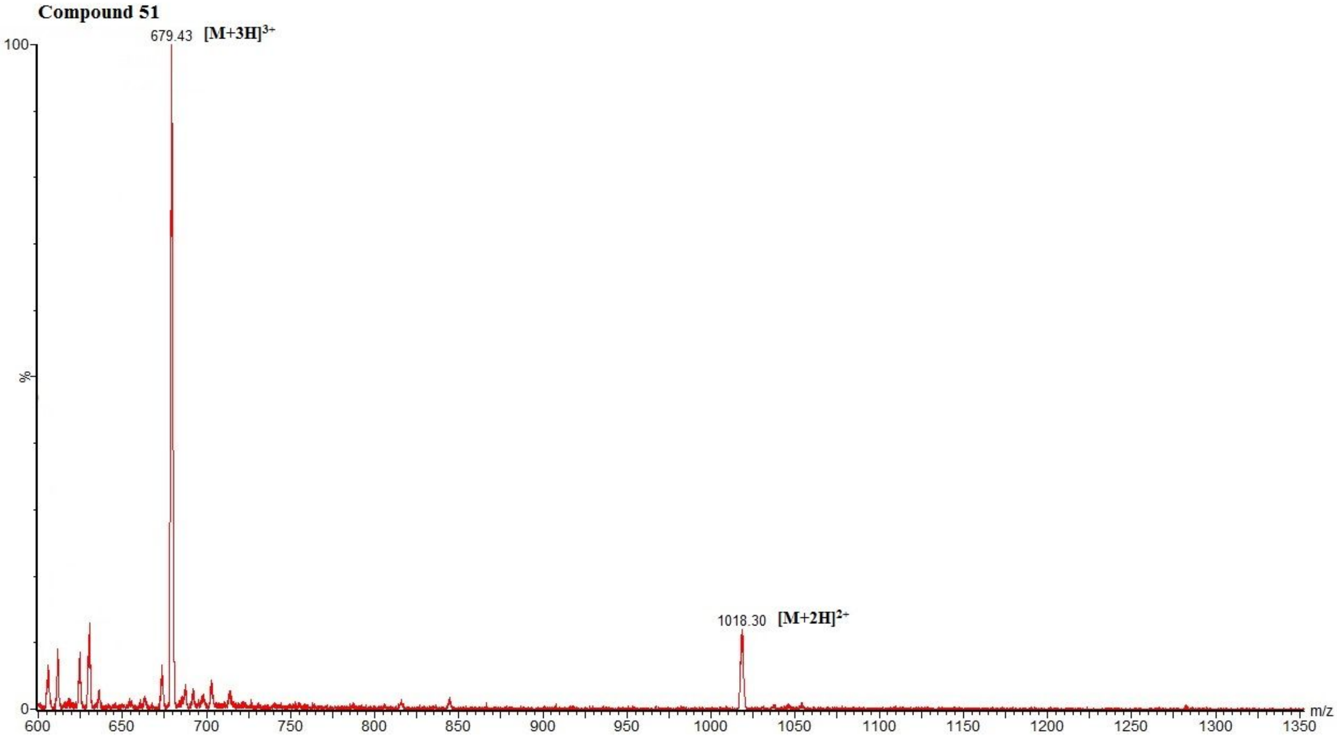


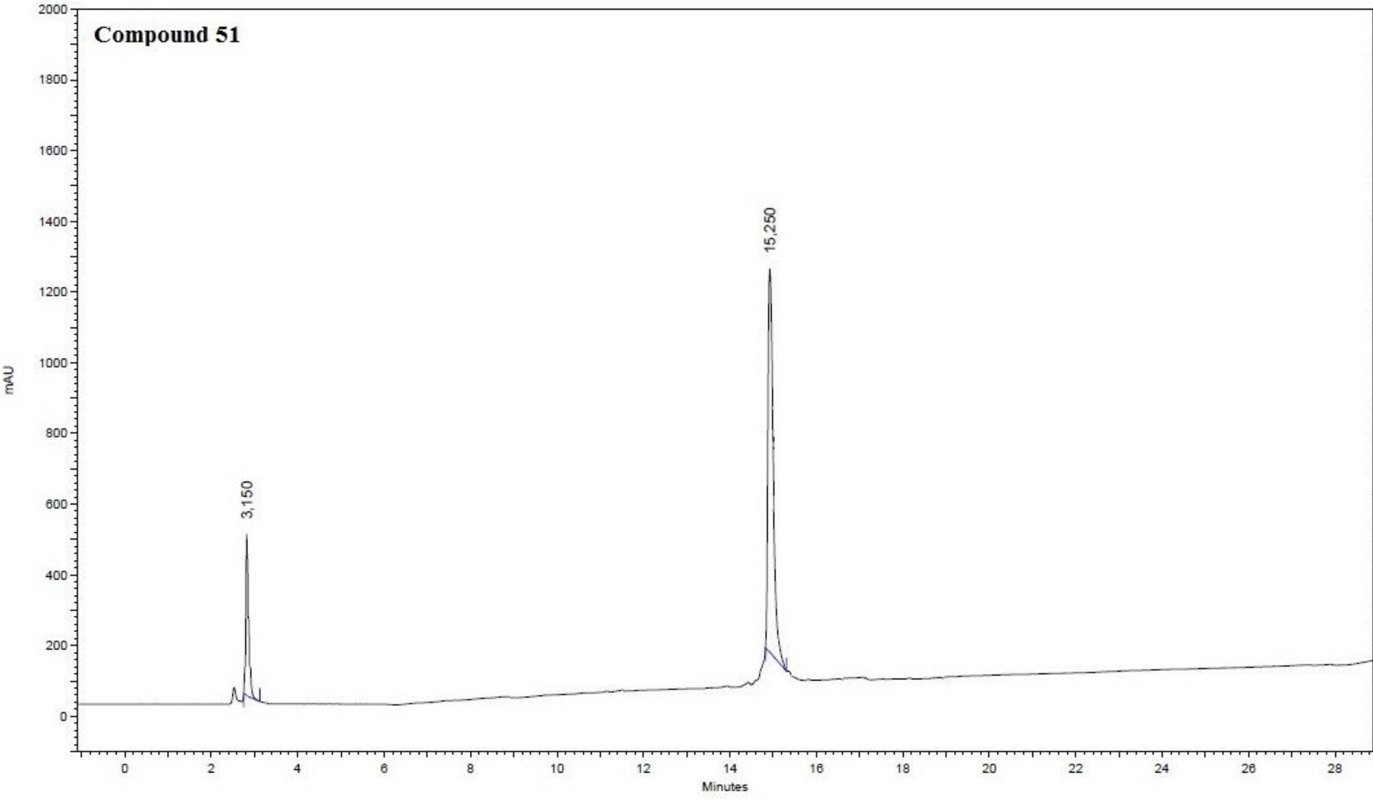

## Compound 51 (HRMS analysis)

| molecular formula | exact mass | | |
|---|---|---|---|
| $C_{94}H_{157}N_{27}O_{21}S$ | 2032,17681 | | |
| molecular ion | m/z (teo) | m/z (exp) | dm/z (ppm) |
| $[M+H]^+$ | 2033,18409 | - | - |
| $[M+2H]^{2+}$ | 1017,09568 | 1017,0960 | -0,3 |
| $[M+3H]^{3+}$ | 678,39955 | 678,4012 | -2,4 |
| $[M+4H]^{4+}$ | 509,05148 | 509,0533 | -3,6 |

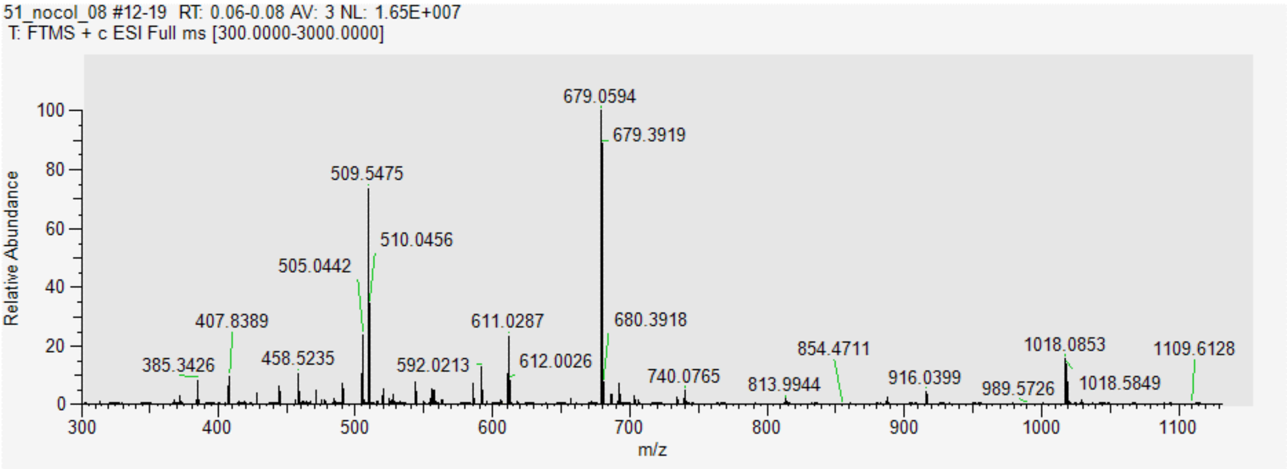


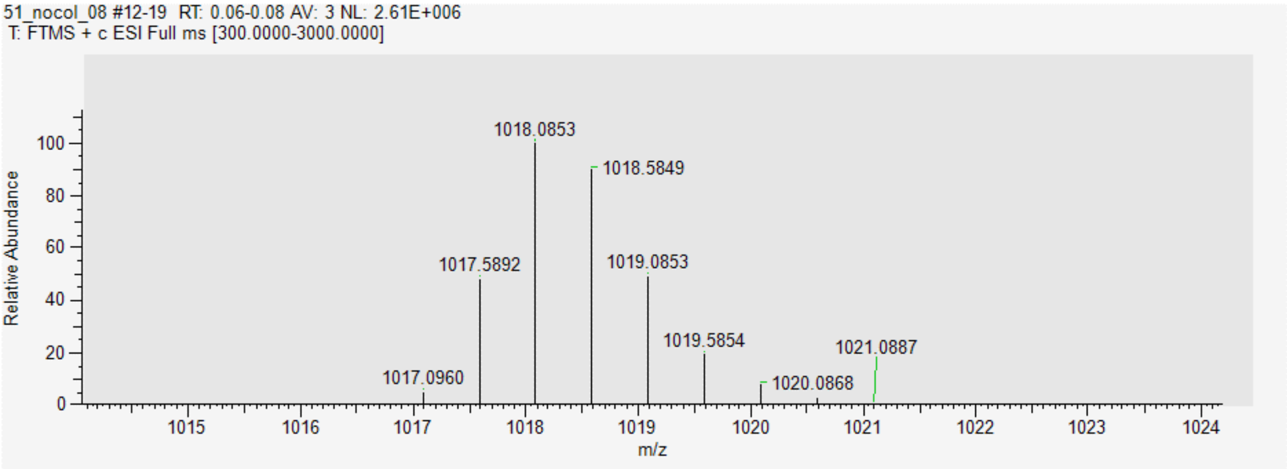


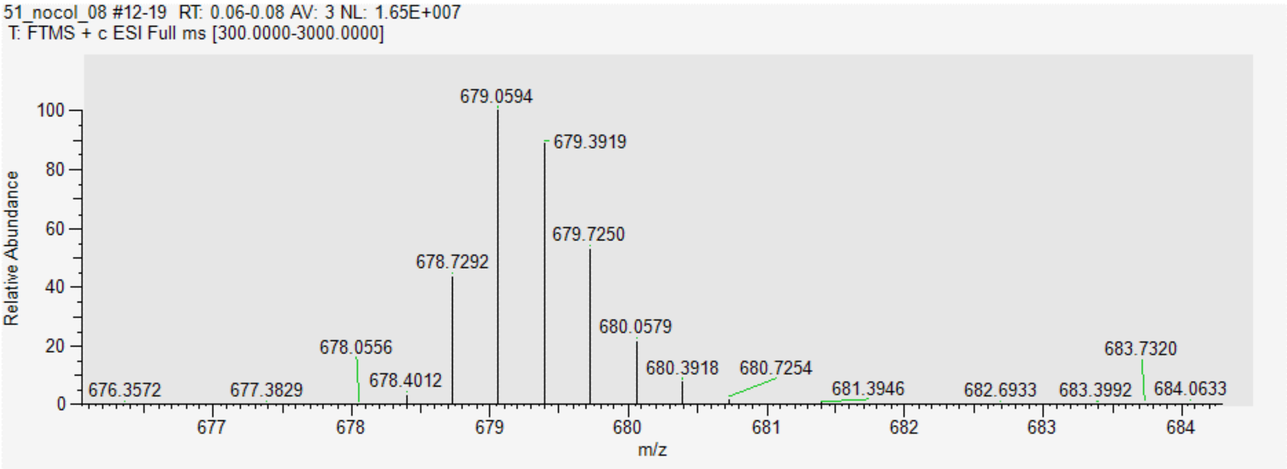

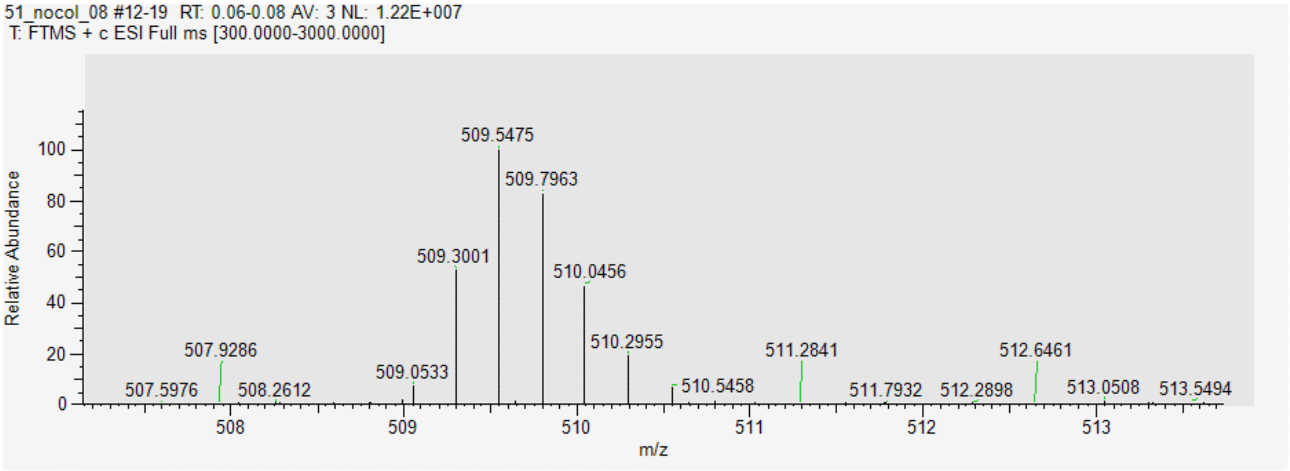

51_nocol_08 #12-19 RT: 0.06-0.08 AV: 3 NL: 1.22E+007
T: FTMS + c ESI Full ms [300.0000-3000.0000]
Relative Abundance
m/z
509.5475
509.7963
509.3001
510.0456
510.2955
507.9286
511.2841
512.6461
509.0533
510.5458
507.5976
508.2612
511.7932
512.2898
513.0508
513.5494

**Compound 52**

MS (ESI): m/z calcd for $C_{105}H_{178}N_{28}O_{22}S$ $[M+3H]^{3+}$ 739.93 found 739.67. $t_R$ = 16.62

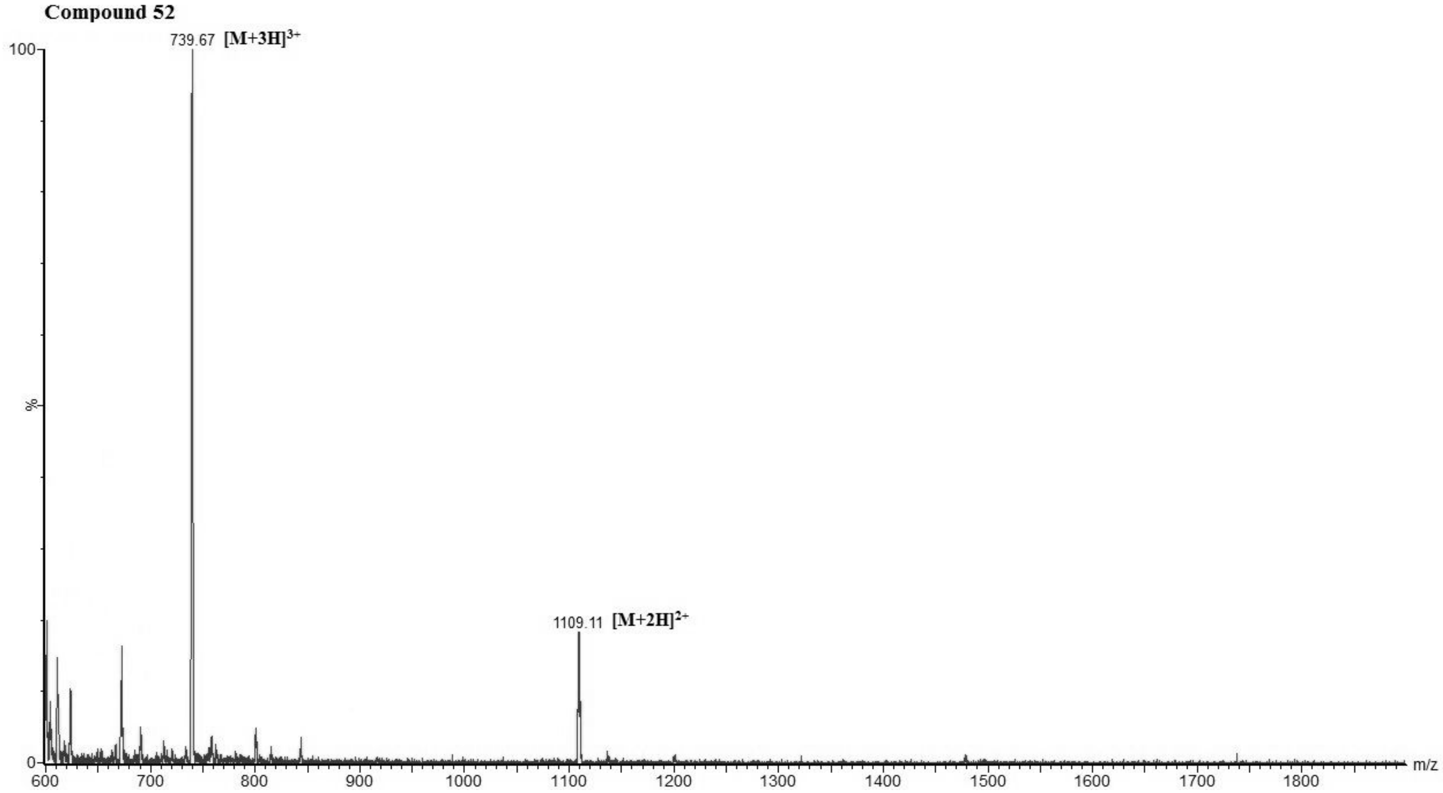


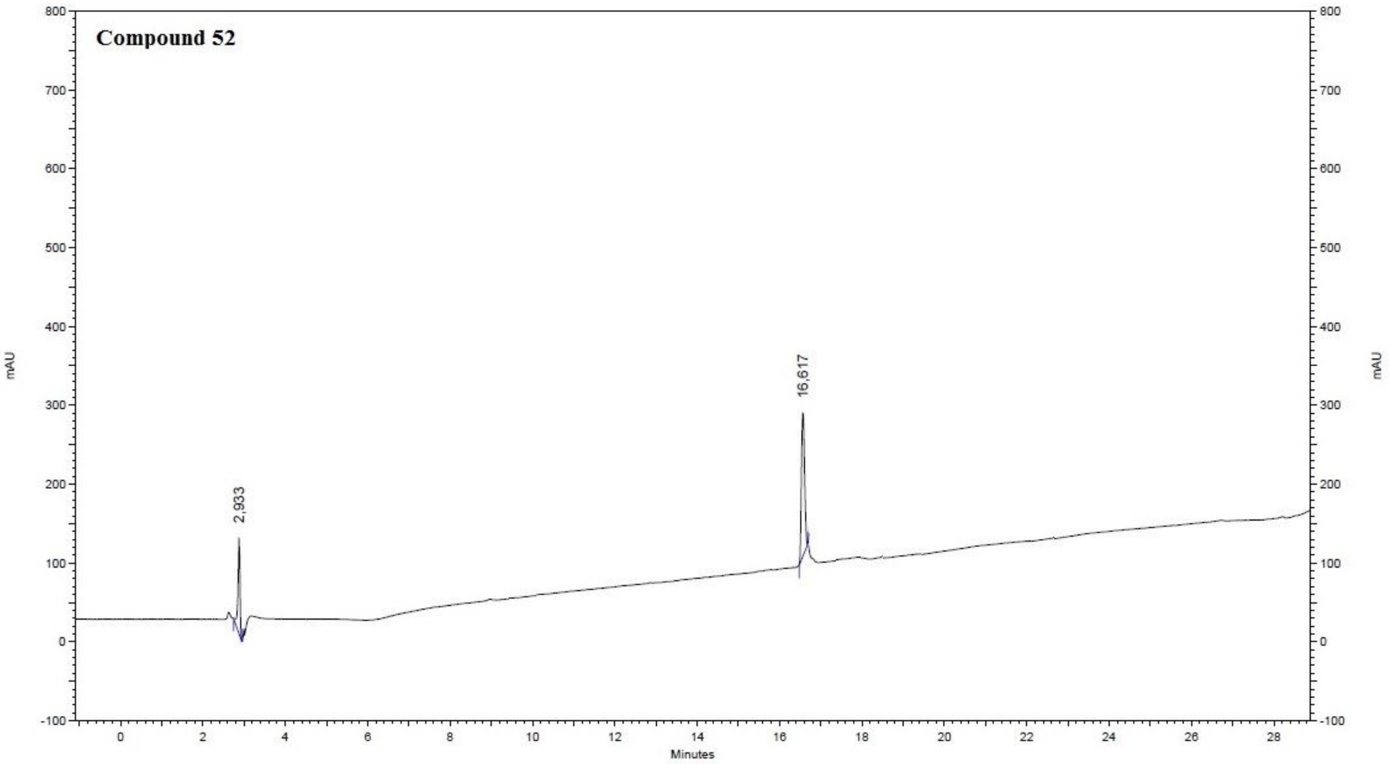

## Compound 52 (HRMS analysis)

| molecular formula | exact mass | | |
|---|---|---|---|
| $C_{105}H_{178}N_{28}O_{22}S$ | 2215,33912 | | |
| molecular ion | m/z (teo) | m/z (exp) | dm/z (ppm) |
| $[M+H]^{+}$ | 2216,34640 | - | - |
| $[M+2H]^{2+}$ | 1108,67684 | 1108,6757 | 1,0 |
| $[M+3H]^{3+}$ | 739,45365 | 739,4528 | 1,1 |
| $[M+4H]^{4+}$ | 554,84206 | 554,8434 | -2,4 |
| $[M+5H]^{5+}$ | 444,07510 | 444,0755 | -0,9 |

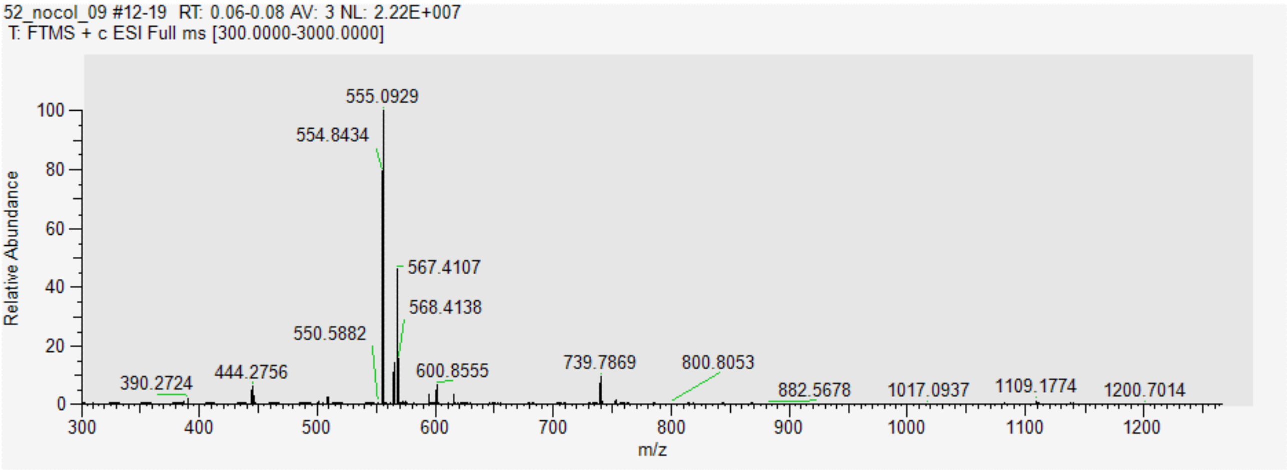


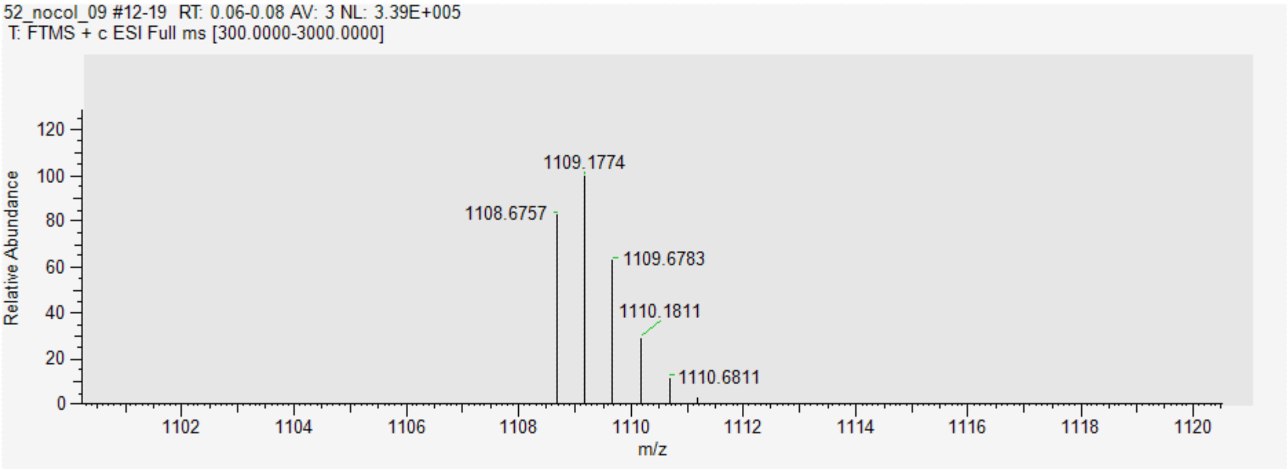

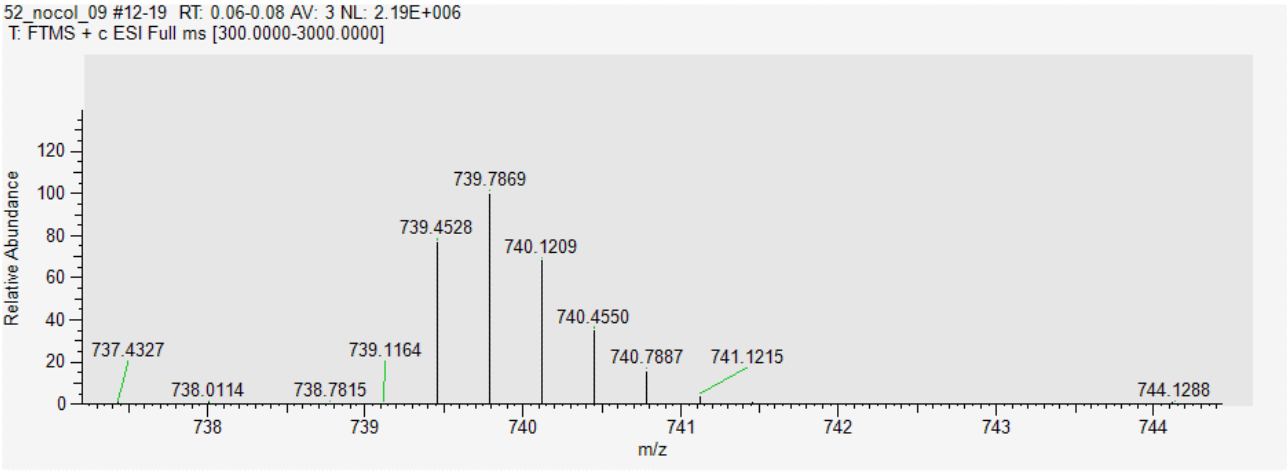
52_nocol_09 #12-19 RT: 0.06-0.08 AV: 3 NL: 2.19E+006
T: FTMS + c ESI Full ms [300.0000-3000.0000]
Relative Abundance
m/z
737.4327
738.0114
738.7815
739.1164
739.4528
739.7869
740.1209
740.4550
740.7887
741.1215
744.1288

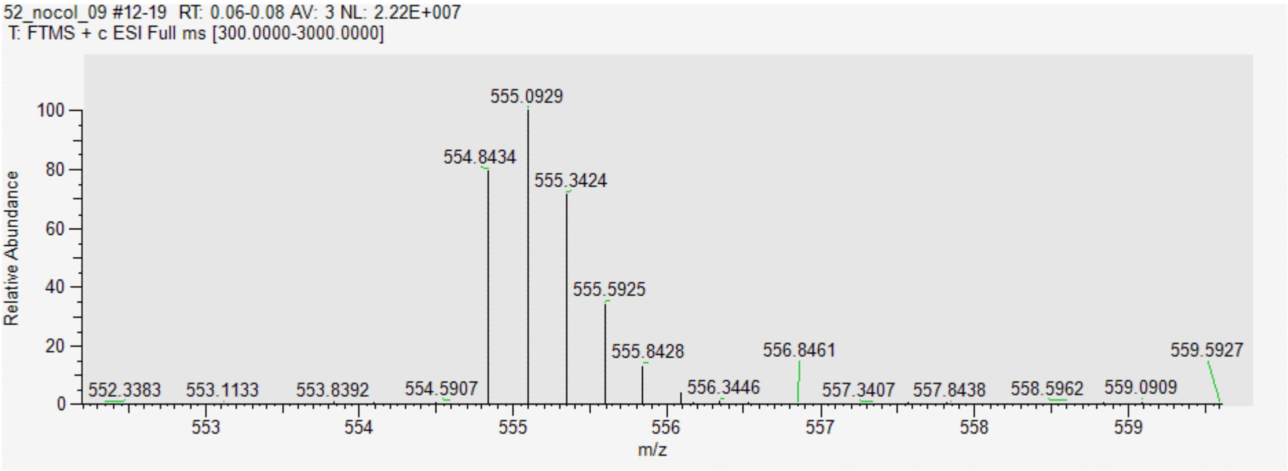
52_nocol_09 #12-19 RT: 0.06-0.08 AV: 3 NL: 2.22E+007
T: FTMS + c ESI Full ms [300.0000-3000.0000]
Relative Abundance
m/z
552.3383
553.1133
553.8392
554.5907
554.8434
555.0929
555.3424
555.5925
555.8428
556.3446
556.8461
557.3407
557.8438
558.5962
559.0909
559.5927

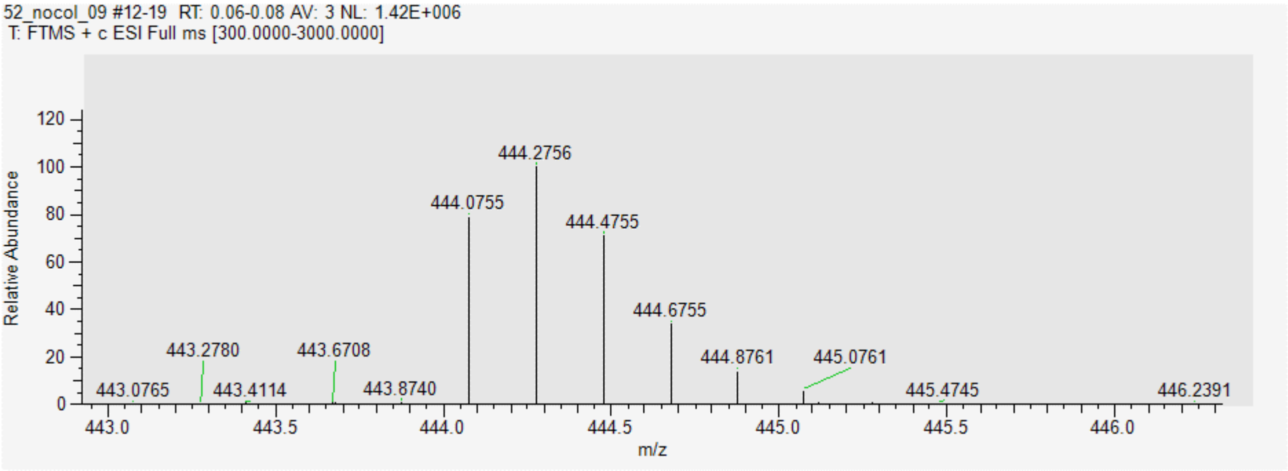
52_nocol_09 #12-19 RT: 0.06-0.08 AV: 3 NL: 1.42E+006
T: FTMS + c ESI Full ms [300.0000-3000.0000]
Relative Abundance
m/z
443.0765
443.2780
443.4114
443.6708
443.8740
444.0755
444.2756
444.4755
444.6755
444.8761
445.0761
445.4745
446.2391

**Compound 53**

MS (ESI): m/z calcd for $C_{84}H_{140}N_{24}O_{17}S$ $[M+3H]^{3+}$ 597.75 found 597.97. $t_R$ = 18.67

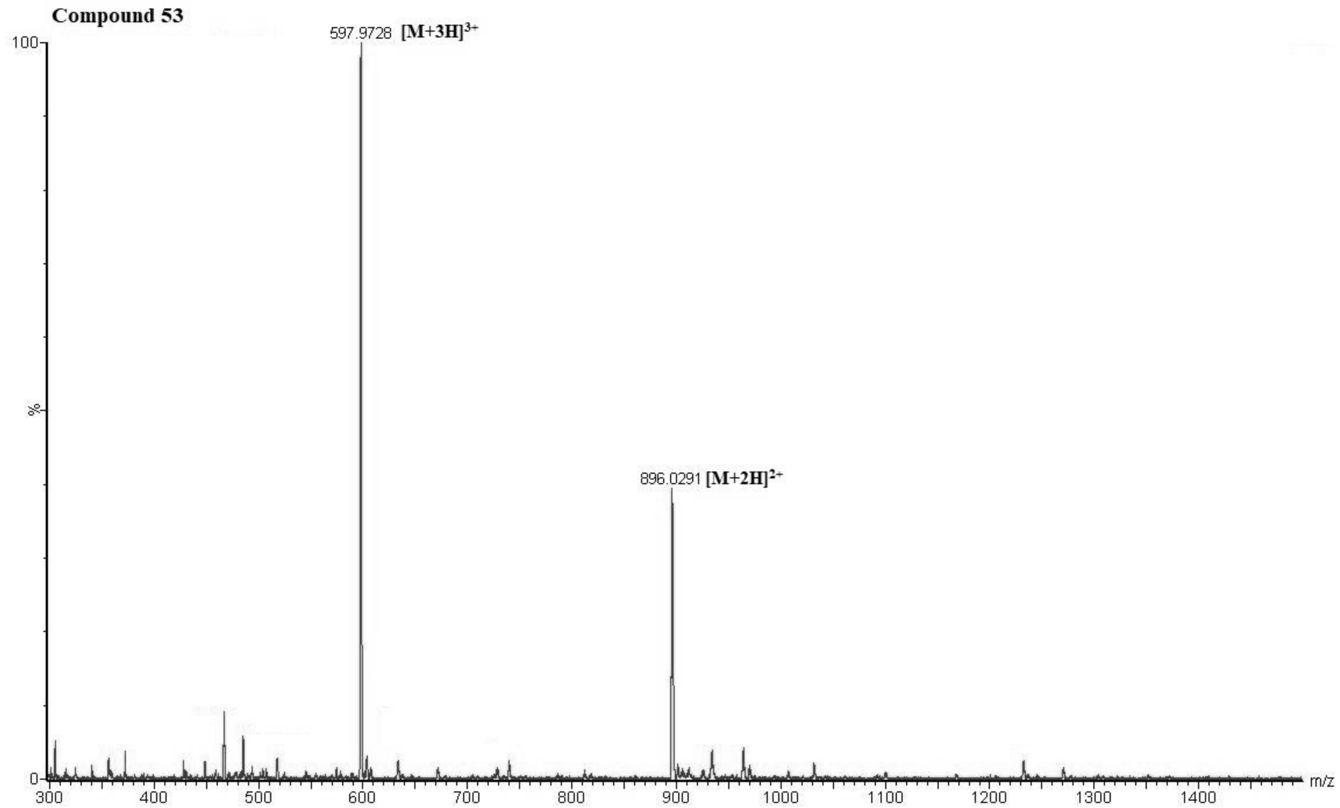


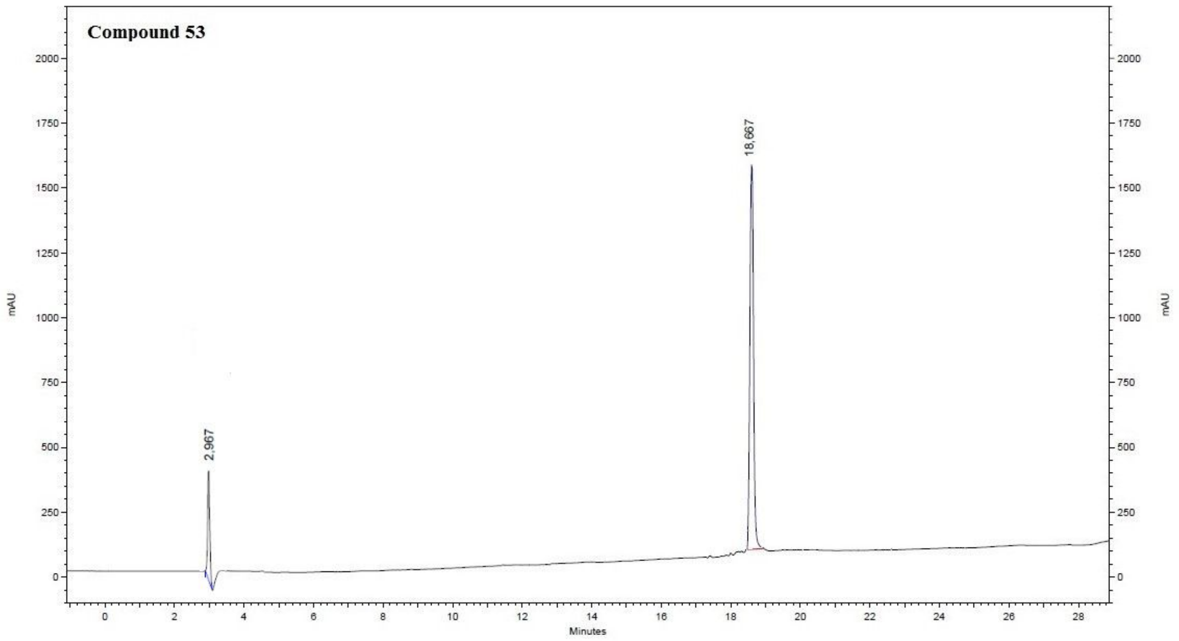

## Compound 53 (HRMS analysis)

| molecular formula | exact mass | | |
|---|---|---|---|
| $C_{84}H_{140}N_{24}O_{17}S$ | 1789,05490 | | |
| molecular ion | m/z (teo) | m/z (exp) | dm/z (ppm) |
| $[M+H]^{+}$ | 1790,06218 | - | - |
| $[M+2H]^{2+}$ | 895,53473 | 895,5324 | 2,6 |
| $[M+3H]^{3+}$ | 597,35891 | 597,3587 | 0,4 |
| $[M+4H]^{4+}$ | 448,27100 | 448,2707 | 0,7 |

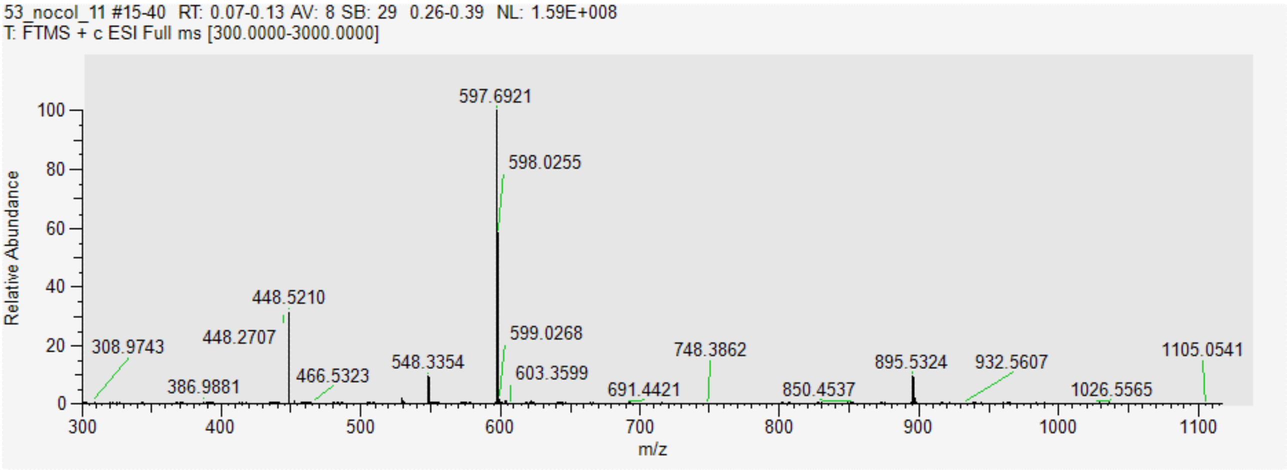


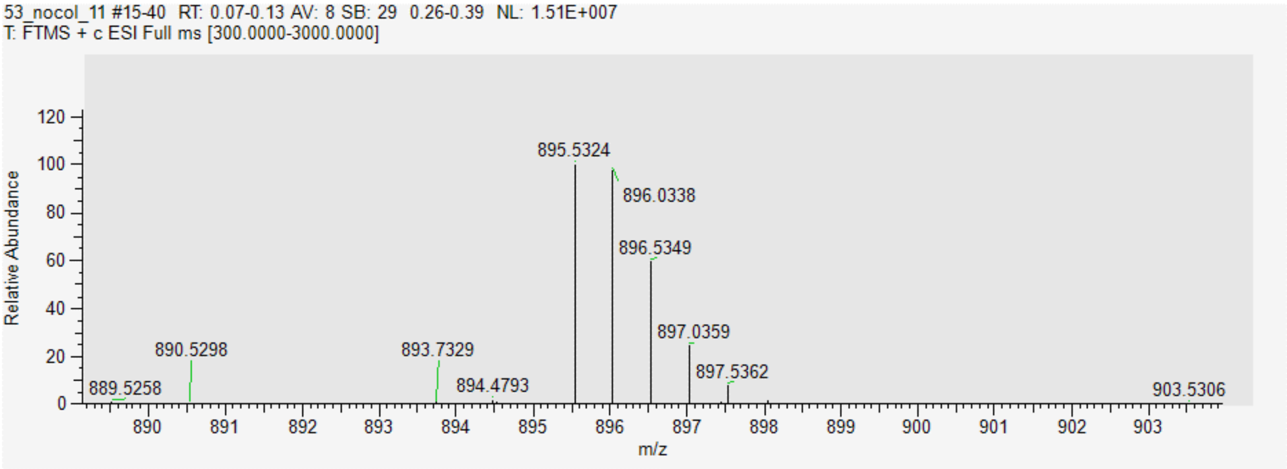

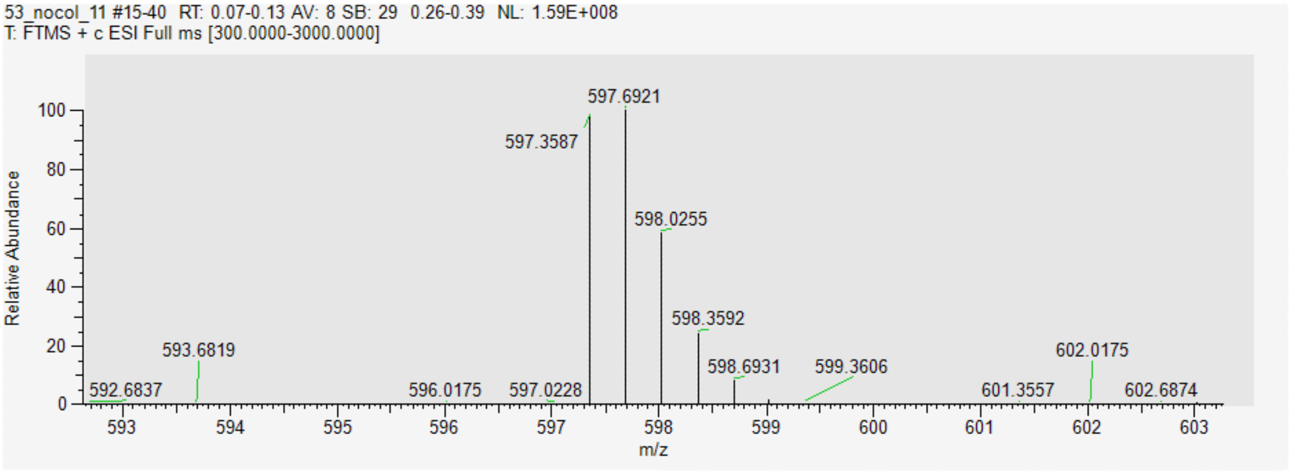
53_nocol_11 #15-40 RT: 0.07-0.13 AV: 8 SB: 29 0.26-0.39 NL: 1.59E+008
T: FTMS + c ESI Full ms [300.0000-3000.0000]
Relative Abundance
m/z
597.6921
597.3587
598.0255
598.3592
593.6819
598.6931
599.3606
602.0175
592.6837
596.0175
597.0228
601.3557
602.6874

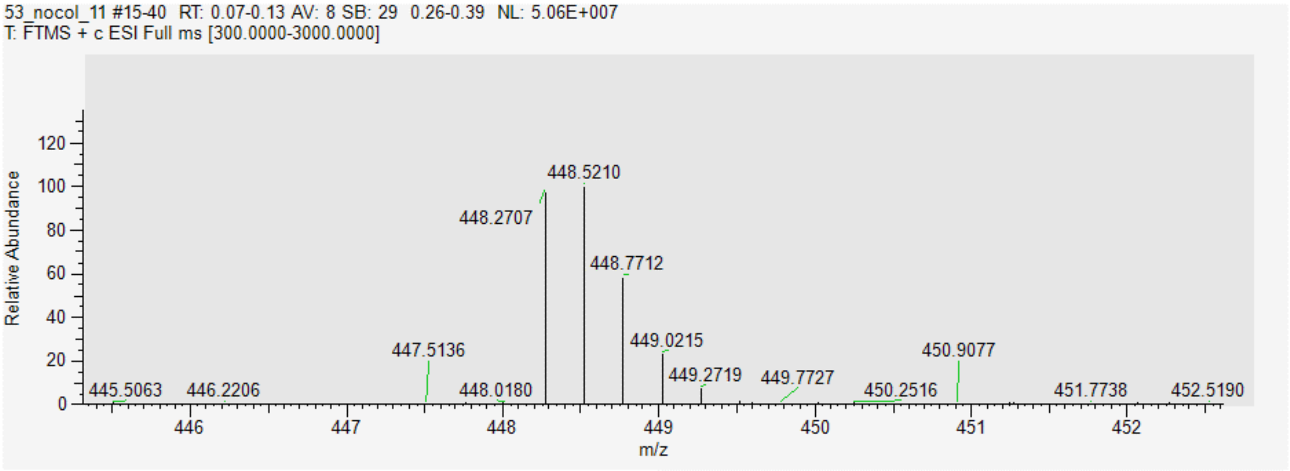
53_nocol_11 #15-40 RT: 0.07-0.13 AV: 8 SB: 29 0.26-0.39 NL: 5.06E+007
T: FTMS + c ESI Full ms [300.0000-3000.0000]
Relative Abundance
m/z
448.5210
448.2707
448.7712
449.0215
447.5136
450.9077
449.2719
449.7727
445.5063
446.2206
448.0180
450.2516
451.7738
452.5190

**Compound 54**

MS (ESI): m/z calcd for $C_{84}H_{140}N_{24}O_{18}S$ $[M+3H]^{3+}$ 603.08 found 603.33. $t_R$ = 16.12

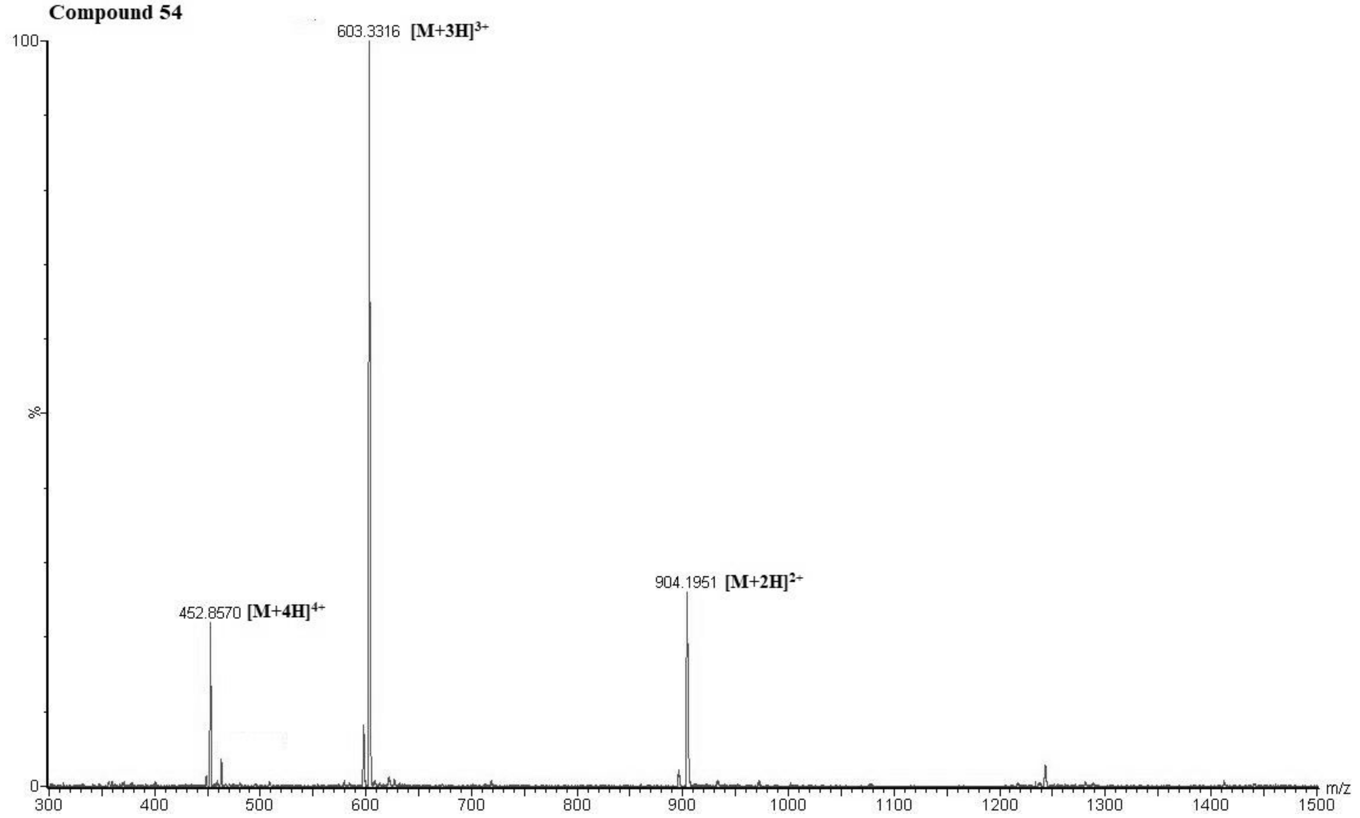


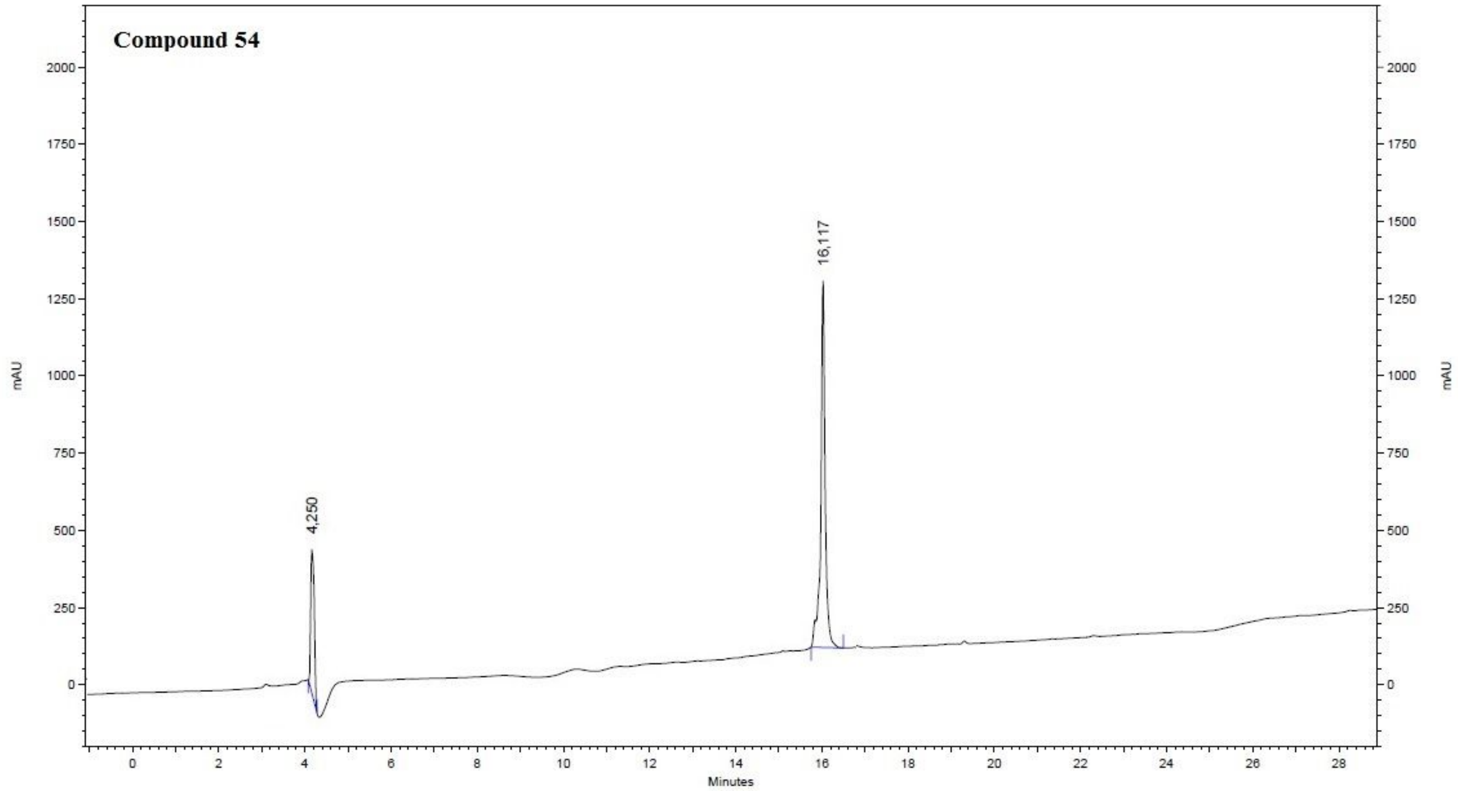

## Compound 54 (HRMS analysis)

| molecular formula | exact mass | | |
|---|---|---|---|
| $C_{84}H_{140}N_{24}O_{18}S$ | 1805,04981 | | |
| molecular ion | m/z (teo) | m/z (exp) | dm/z (ppm) |
| $[M+H]^{+}$ | 1806,05709 | - | - |
| $[M+2H]^{2+}$ | 903,53218 | 903,5306 | 1,8 |
| $[M+3H]^{3+}$ | 602,69055 | 602,6910 | -0,8 |
| $[M+4H]^{4+}$ | 452,26973 | 452,2700 | -0,6 |

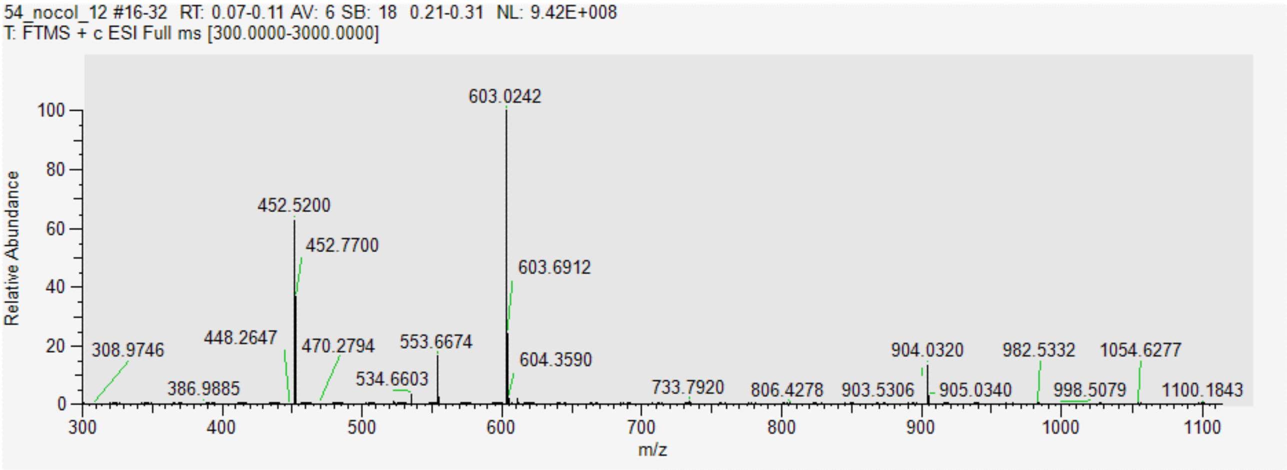


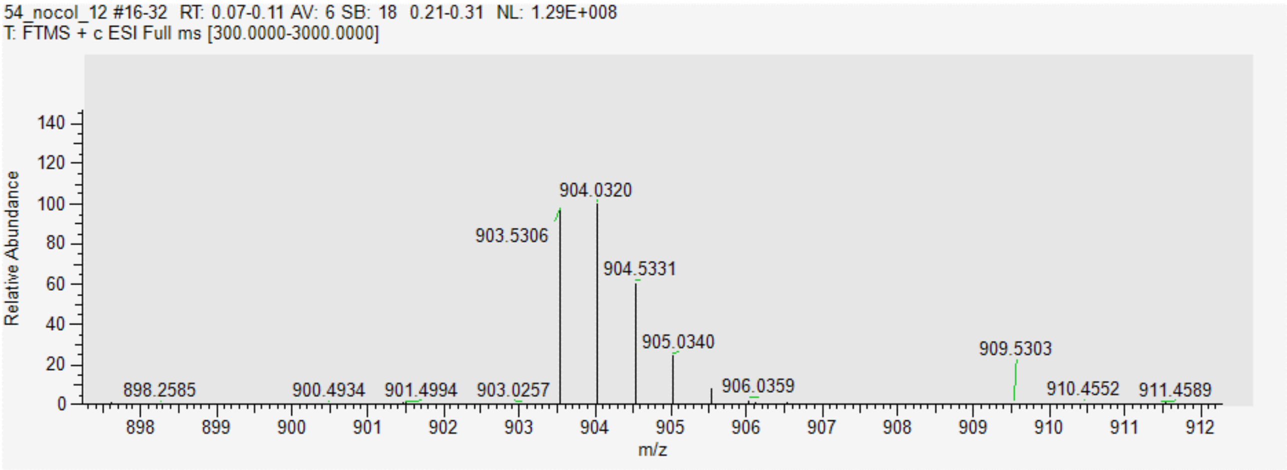


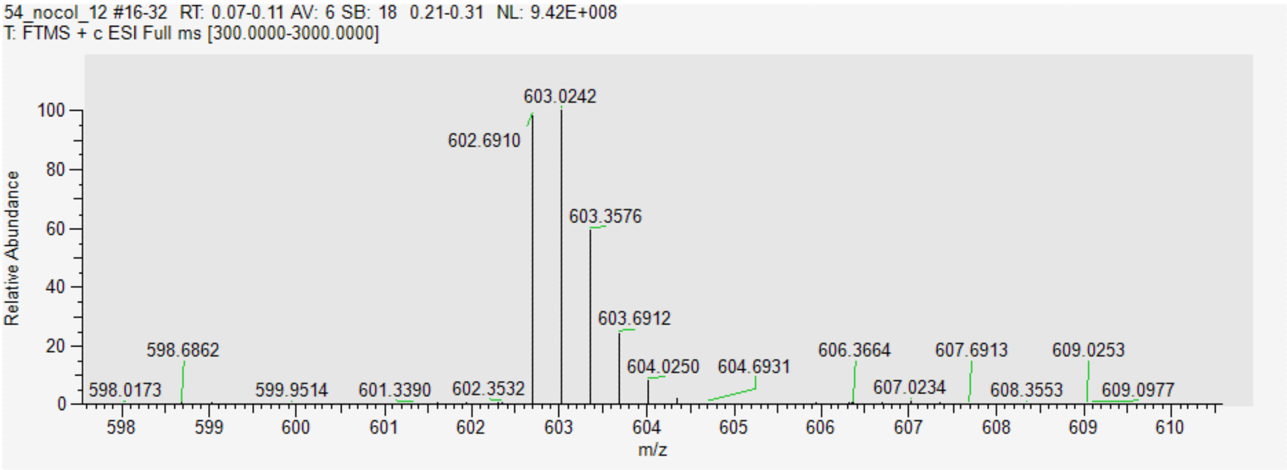

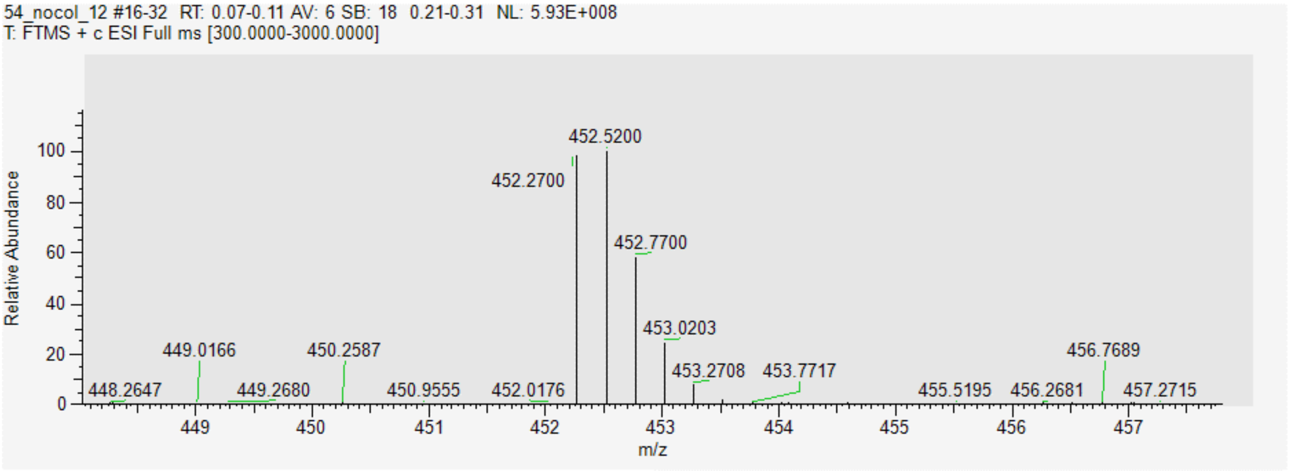
54_nocol_12 #16-32 RT: 0.07-0.11 AV: 6 SB: 18 0.21-0.31 NL: 5.93E+008
T: FTMS + c ESI Full ms [300.0000-3000.0000]
Relative Abundance
m/z
452.5200
452.2700
452.7700
453.0203
449.0166
450.2587
456.7689
453.2708
453.7717
448.2647
449.2680
450.9555
452.0176
455.5195
456.2681
457.2715

**Compound 55**

MS (ESI): m/z calcd for $C_{81}H_{133}N_{21}O_{18}S$ $[M+3H]^{3+}$ 574.71 found 574.77. $t_R$ = 20.07

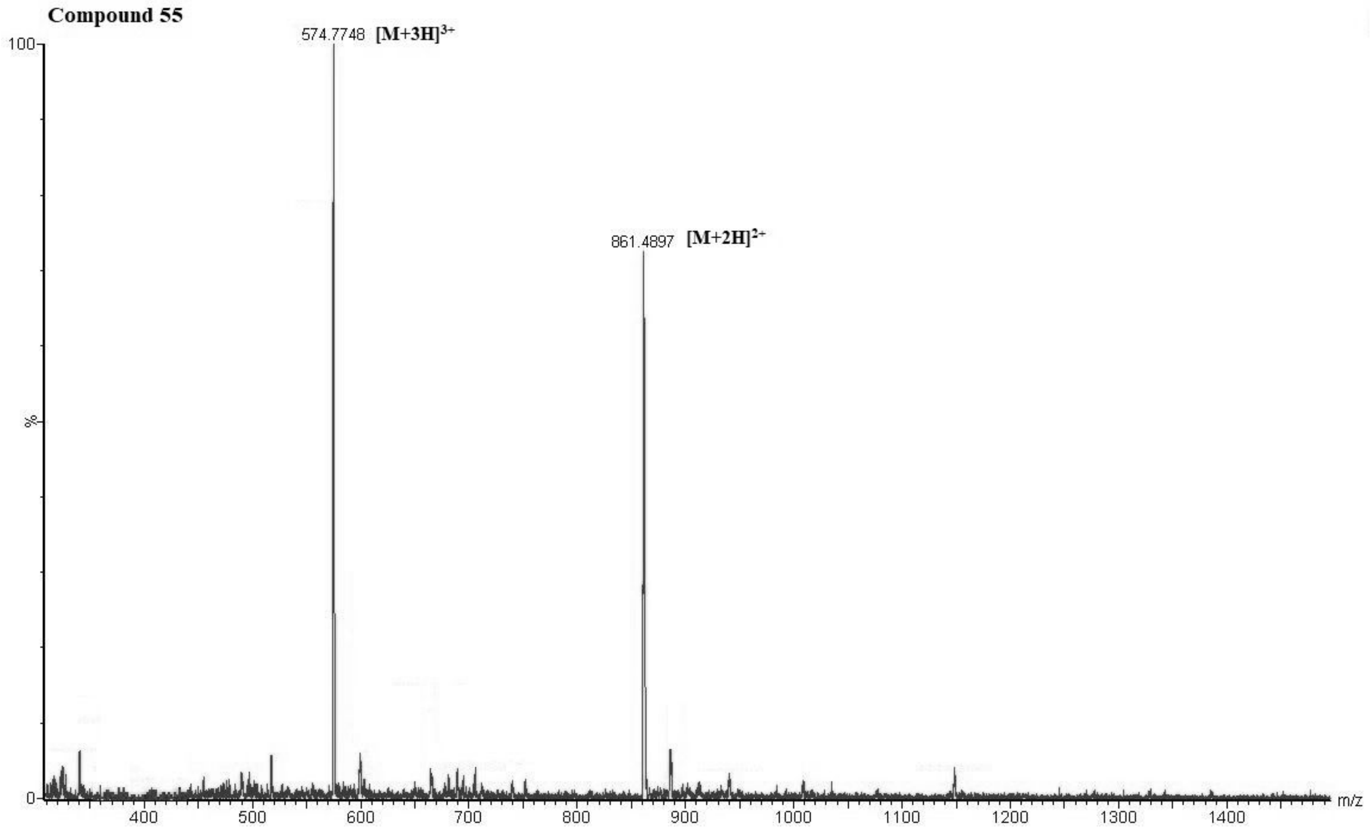


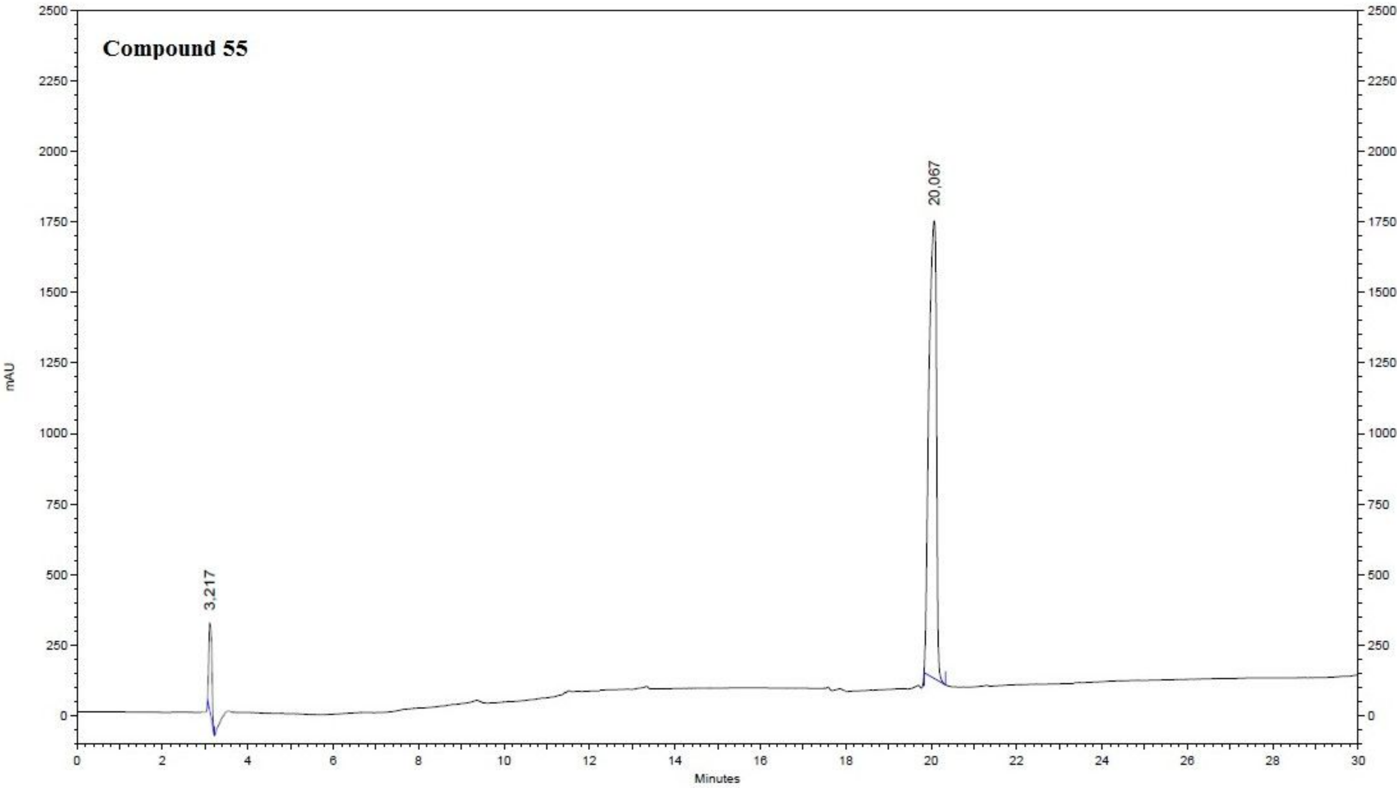

## Compound 55 (HRMS analysis)

| molecular formula | exact mass | | |
|---|---|---|---|
| $C_{81}H_{133}N_{21}O^{18}S$ | 1719,98582 | | |
| molecular ion | m/z (teo) | m/z (exp) | dm/z (ppm) |
| $[M+H]^{+}$ | 1720,99310 | - | - |
| $[M+2H]^{2+}$ | 861,00019 | 860,9992 | 1,1 |
| $[M+3H]^{3+}$ | 574,33588 | 574,3361 | -0,4 |
| $[M+4H]^{4+}$ | 431,00373 | 431,0038 | -0,2 |

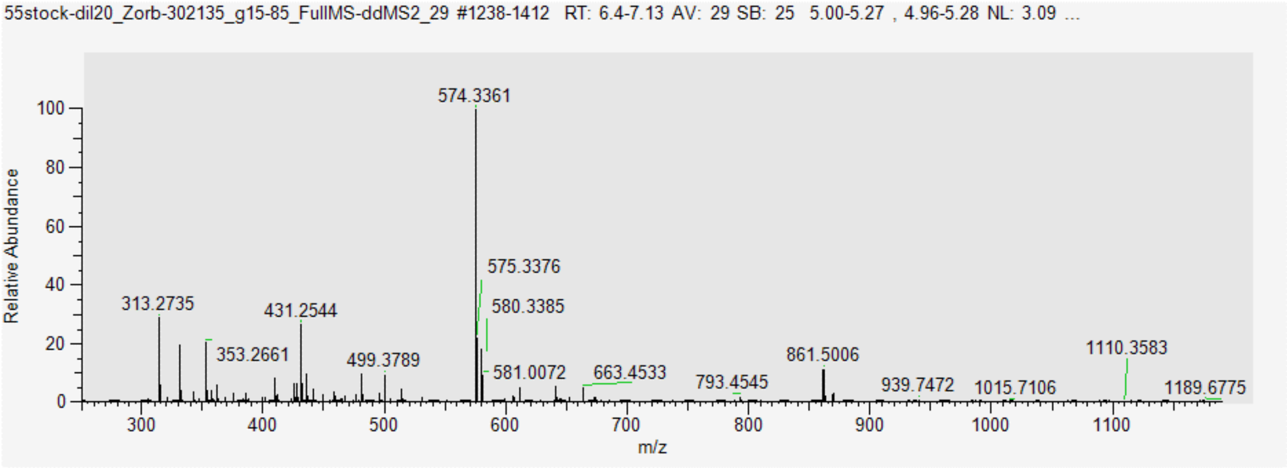


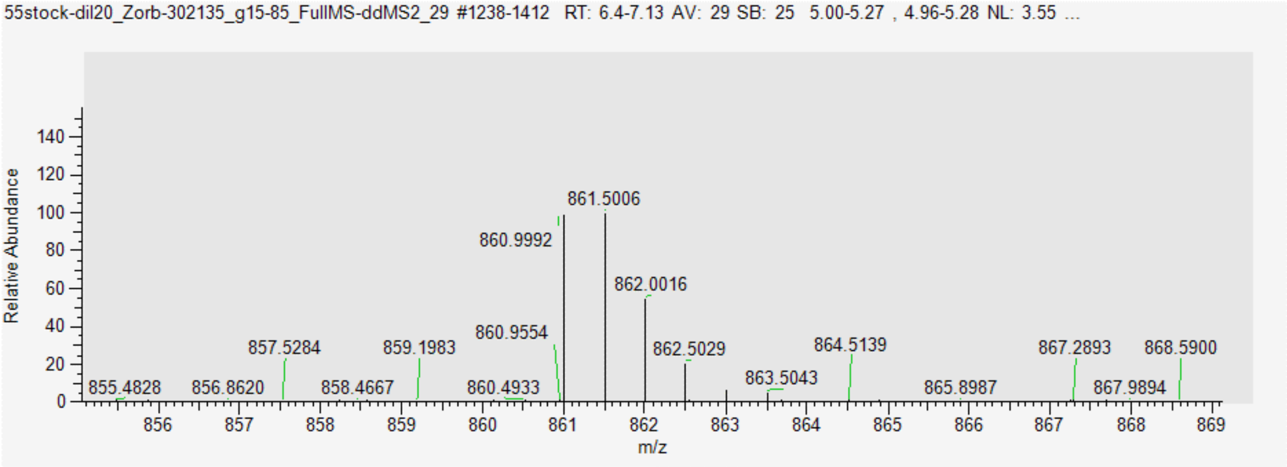

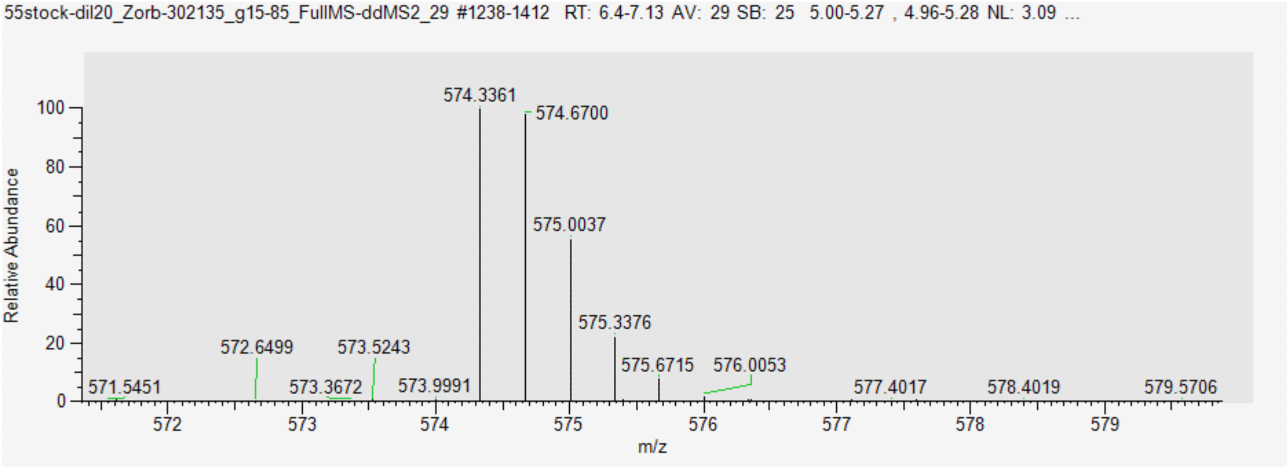
55stock-dil20_Zorb-302135_g15-85_FullMS-ddMS2_29 #1238-1412 RT: 6.4-7.13 AV: 29 SB: 25 5.00-5.27 , 4.96-5.28 NL: 3.09 ...
Relative Abundance
m/z
574.3361
574.6700
575.0037
575.3376
575.6715
576.0053
572.6499
573.5243
571.5451
573.3672
573.9991
577.4017
578.4019
579.5706

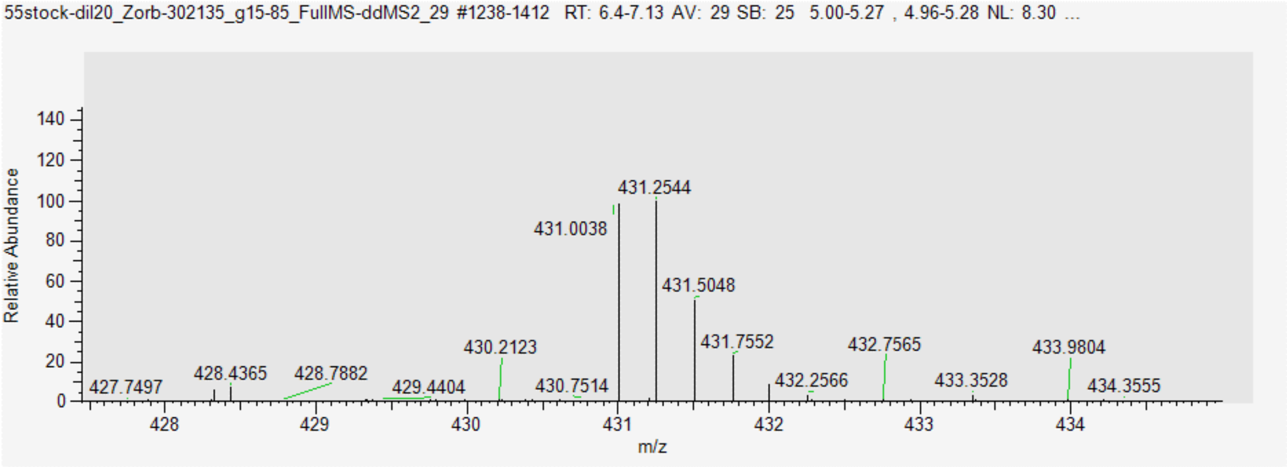
55stock-dil20_Zorb-302135_g15-85_FullMS-ddMS2_29 #1238-1412 RT: 6.4-7.13 AV: 29 SB: 25 5.00-5.27 , 4.96-5.28 NL: 8.30 ...
Relative Abundance
m/z
431.2544
431.0038
431.5048
431.7552
430.2123
432.7565
433.9804
427.7497
428.4365
428.7882
429.4404
430.7514
432.2566
433.3528
434.3555

**Compound 56**

MS (ESI): m/z calcd for $C_{81}H_{133}N_{23}O_{18}S$ $[M+3H]^{3+}$ 584.05 found 584.35. $t_R$ = 19.90

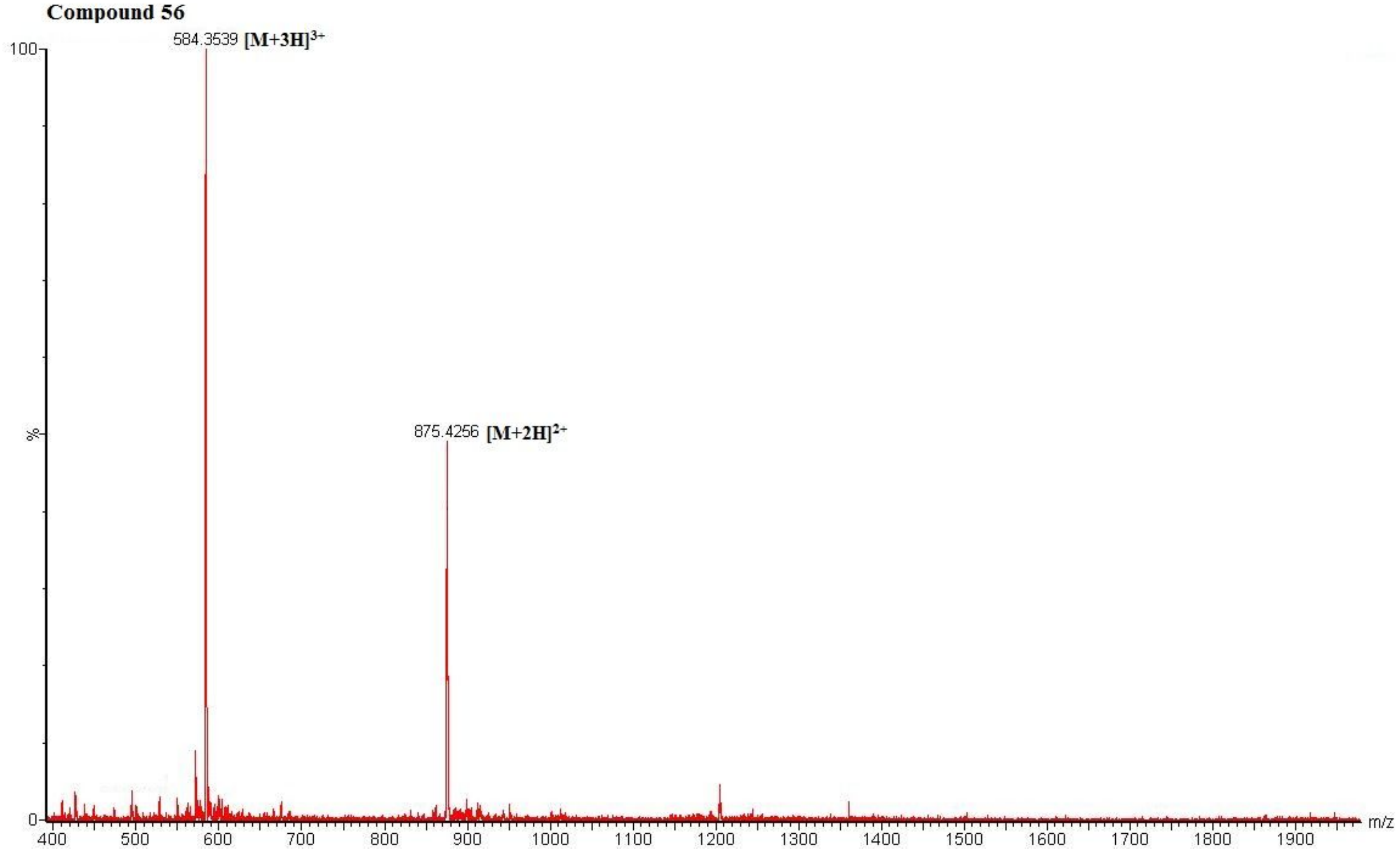


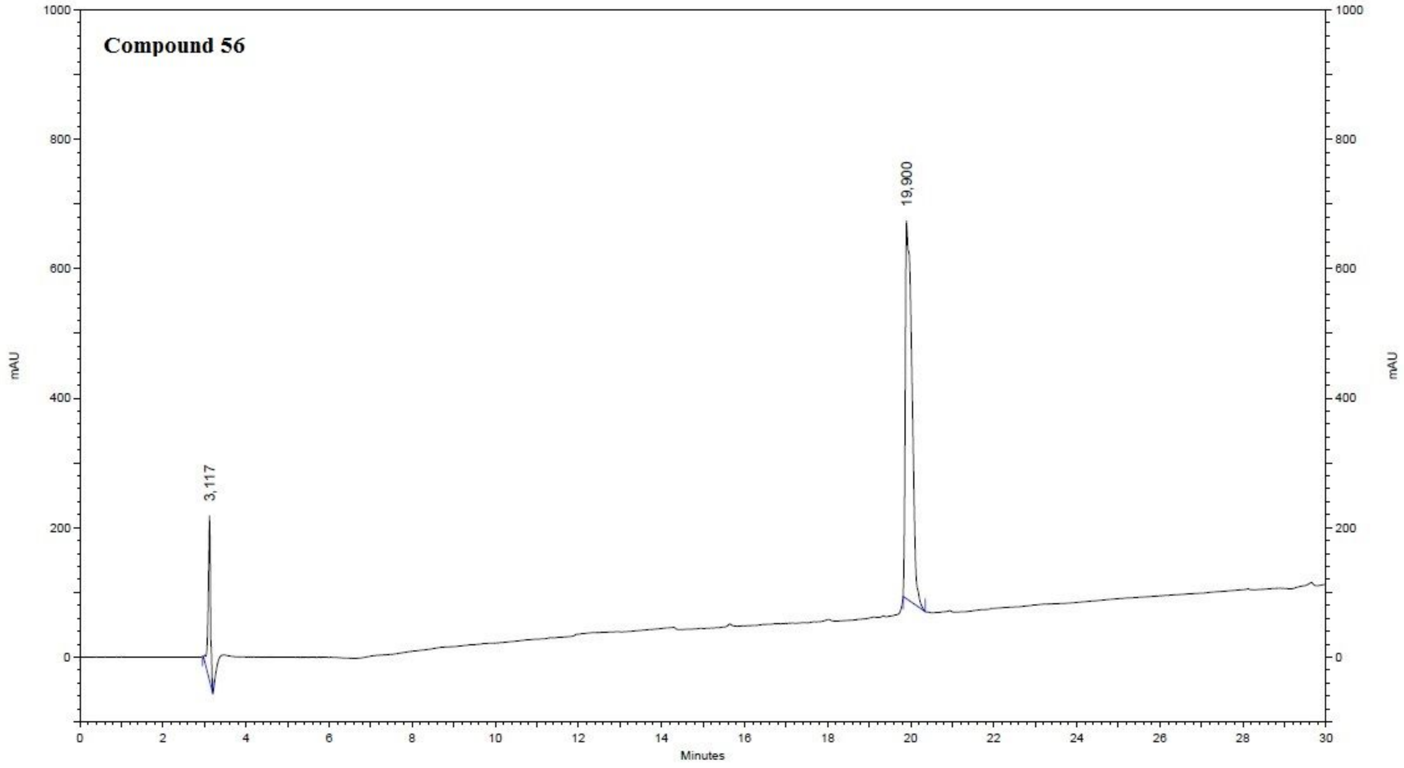

**Compound 57**

MS (ESI): m/z calcd for $C_{66}H_{95}N_{11}O_{14}S$ $[M+H]^+$ 1299.60 found 1299.31. $t_R$ = 23.08

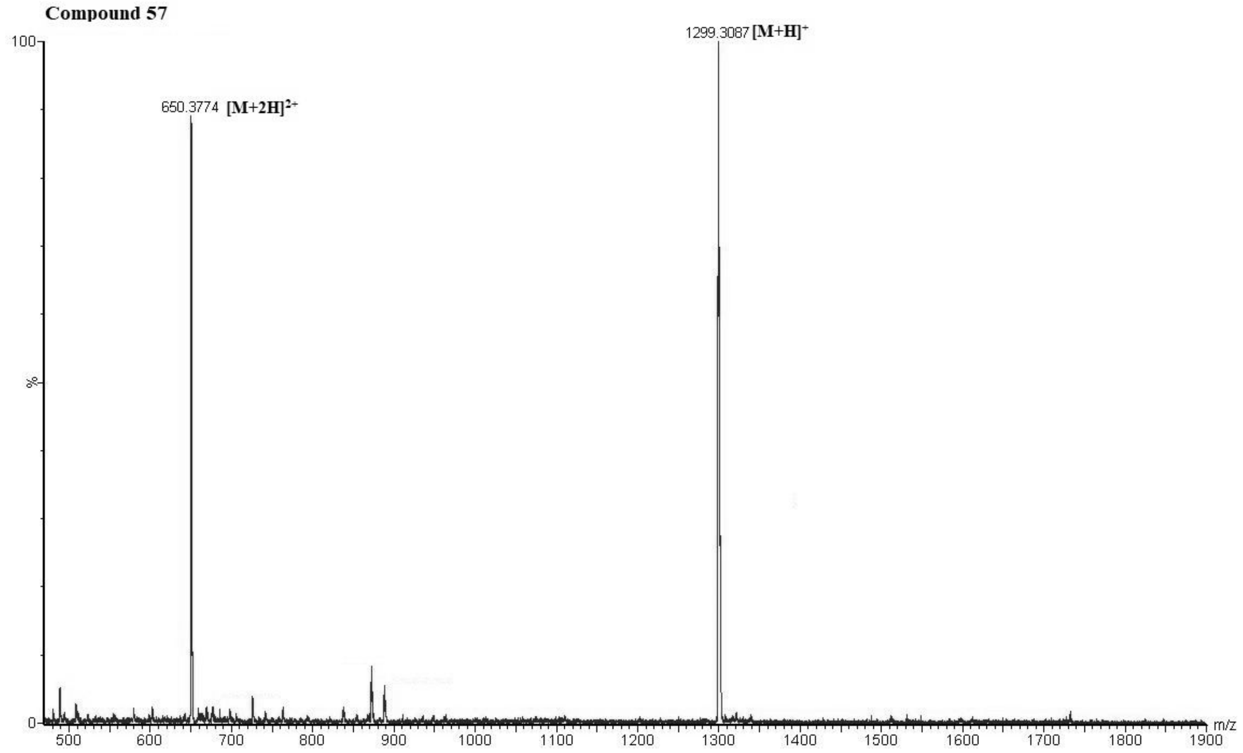


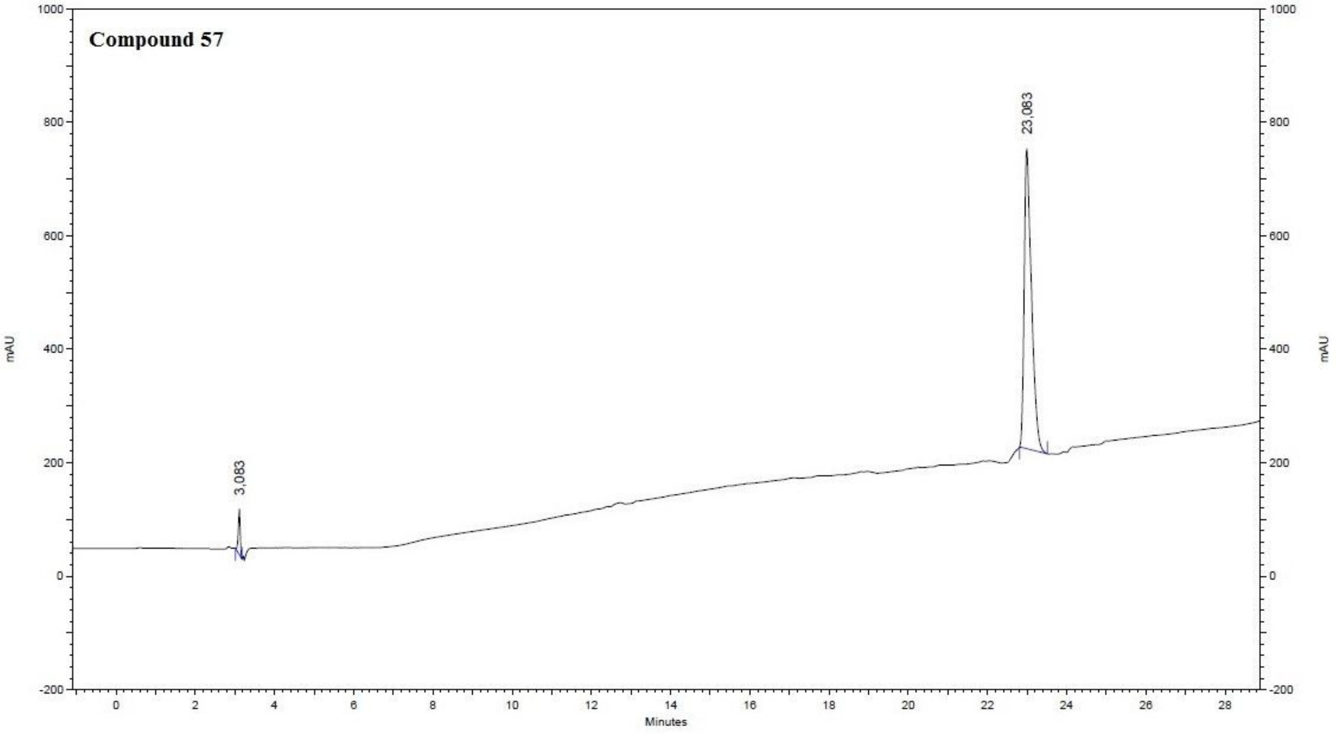

## Compound 57 (HRMS analysis)

| molecular formula | exact mass | | |
|---|---|---|---|
| $C_{66}H_{95}N_{11}O_{14}S$ | 1297,67807 | | |
| molecular ion | m/z (teo) | m/z (exp) | dm/z (ppm) |
| $[M+H]^+$ | 1298,68535 | 1298,687 | -1,0 |
| $[M+2H]^{2+}$ | 649,84631 | 649,8469 | -0,9 |

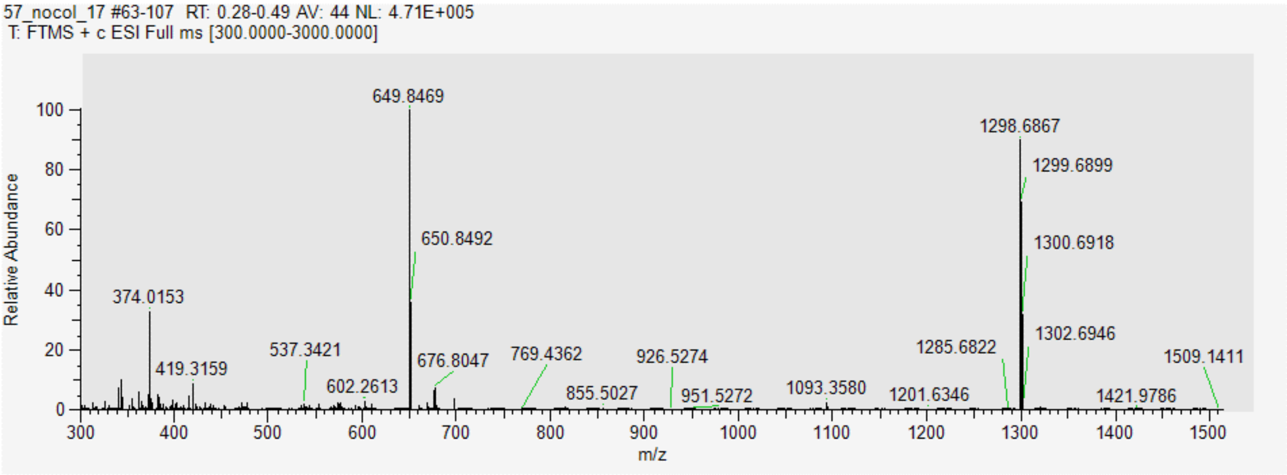


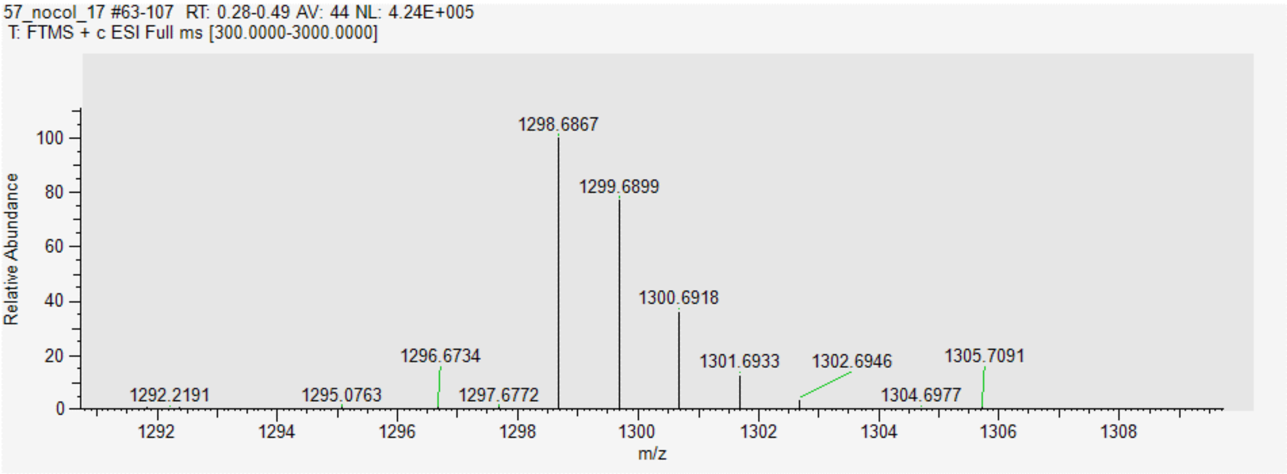


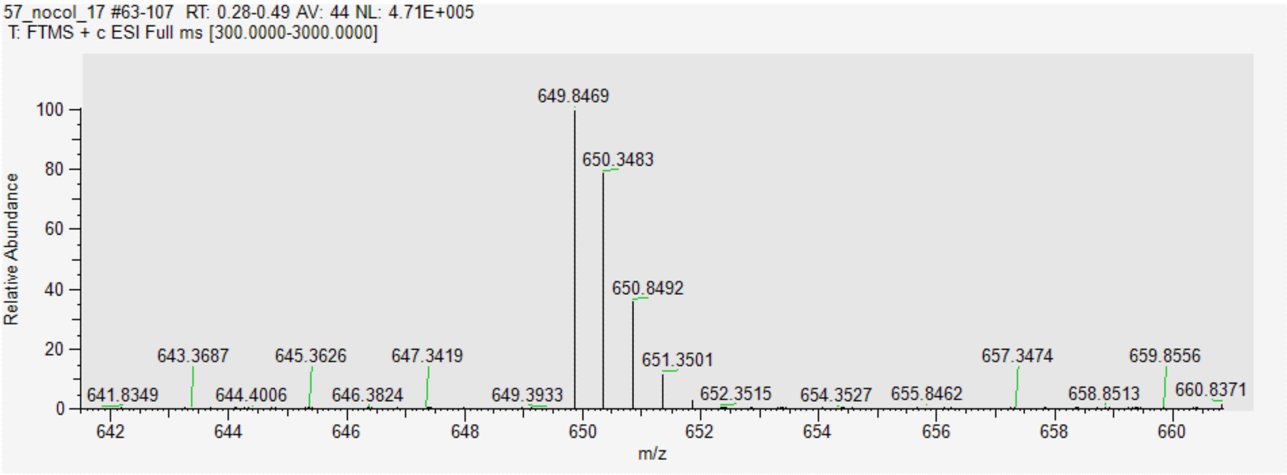

**Compound 58**

MS (ESI): m/z calcd for $C_{70}H_{107}N_{13}O_{14}S_3$ $[M+2H]^{2+}$ 726.44 found 726.46. $t_R$ = 22.75

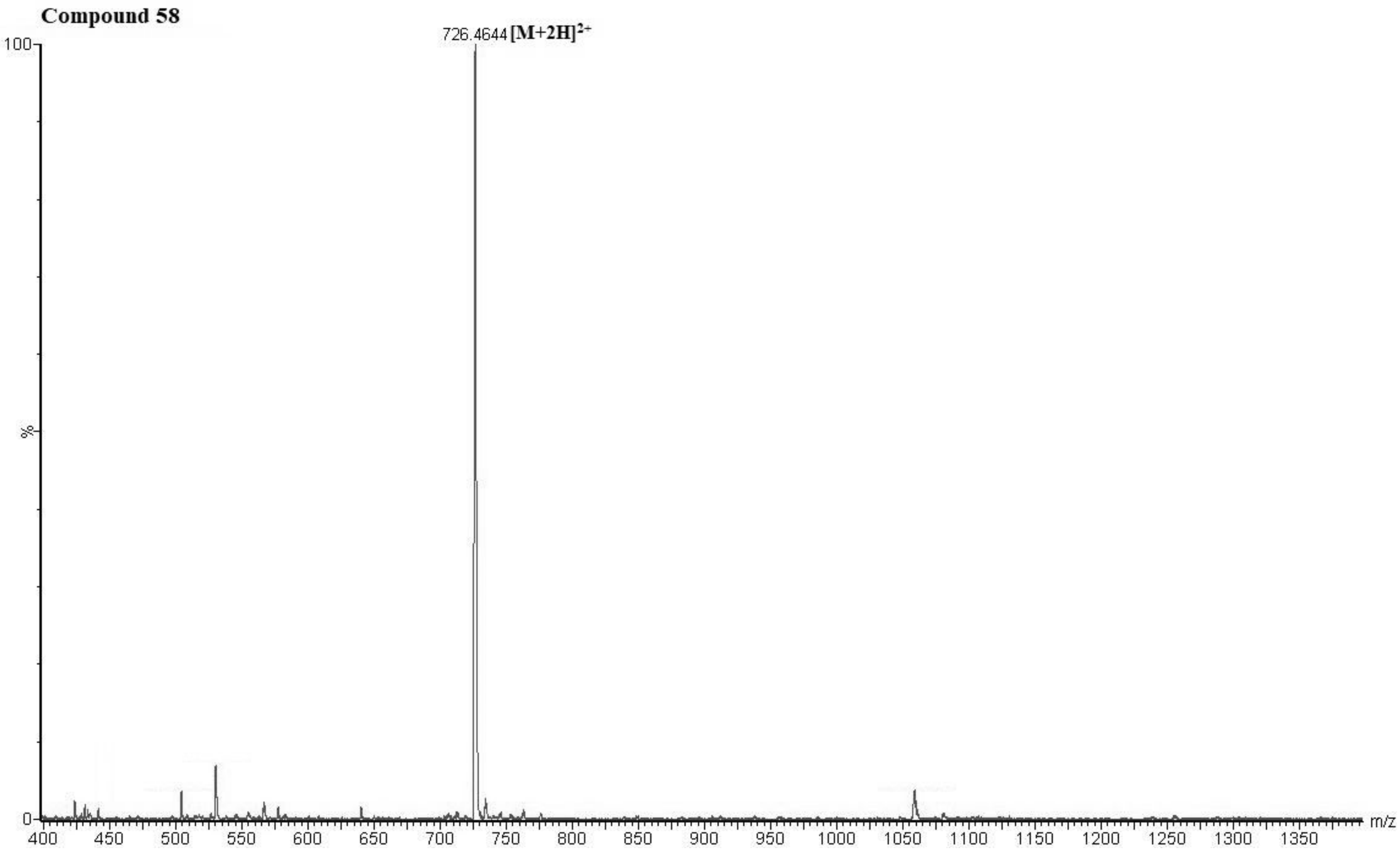


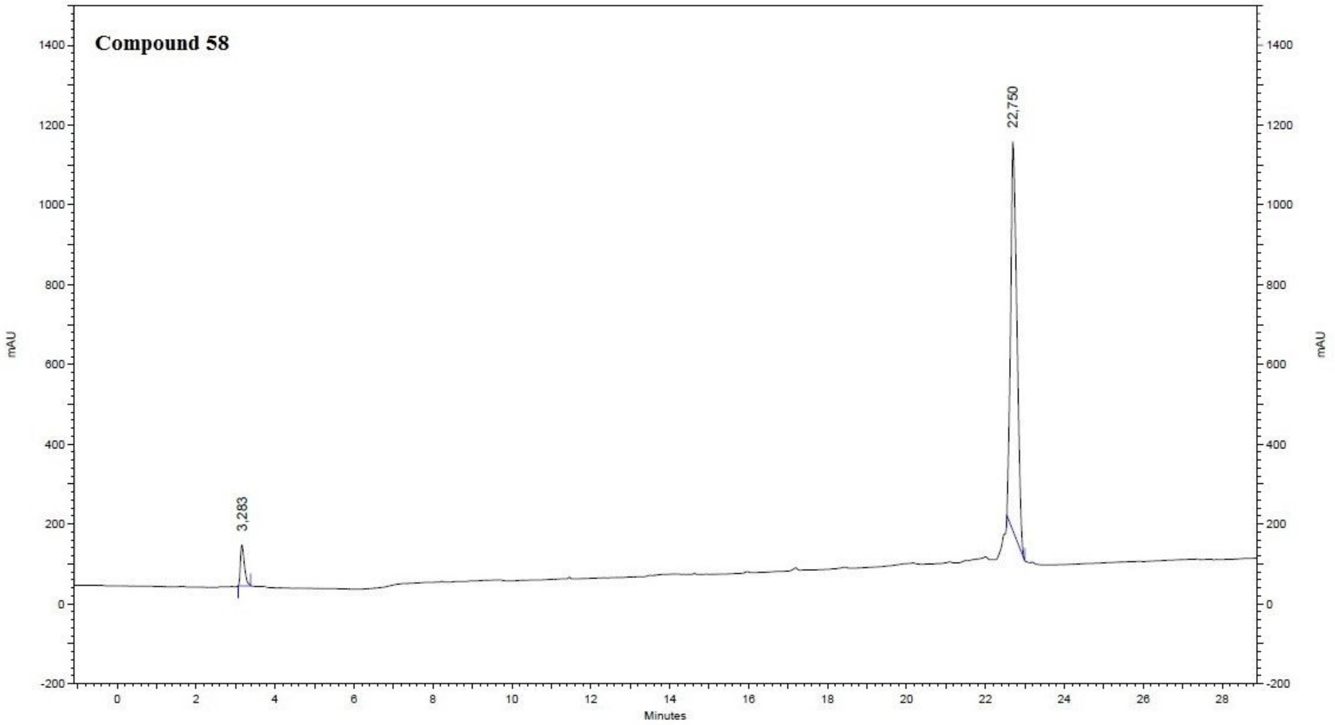

## Compound 58 (HRMS analysis)

| molecular formula | exact mass | | |
|---|---|---|---|
| $C_{70}H_{107}N_{13}O_{14}S_3$ | 1449,72226 | | |
| molecular ion | m/z (teo) | m/z (exp) | dm/z (ppm) |
| $[M+H]^+$ | 1450,72954 | 1450,7284 | 0,8 |
| $[M+2H]^{2+}$ | 725,86841 | 725,8690 | -0,8 |
| $[M+3H]^{3+}$ | 484,24803 | 484,2481 | -0,1 |

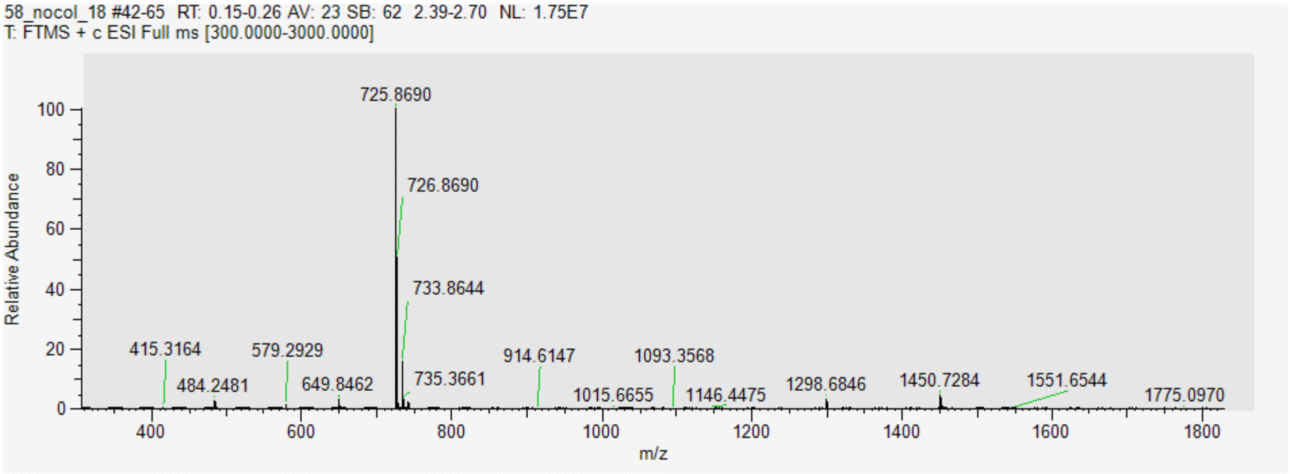


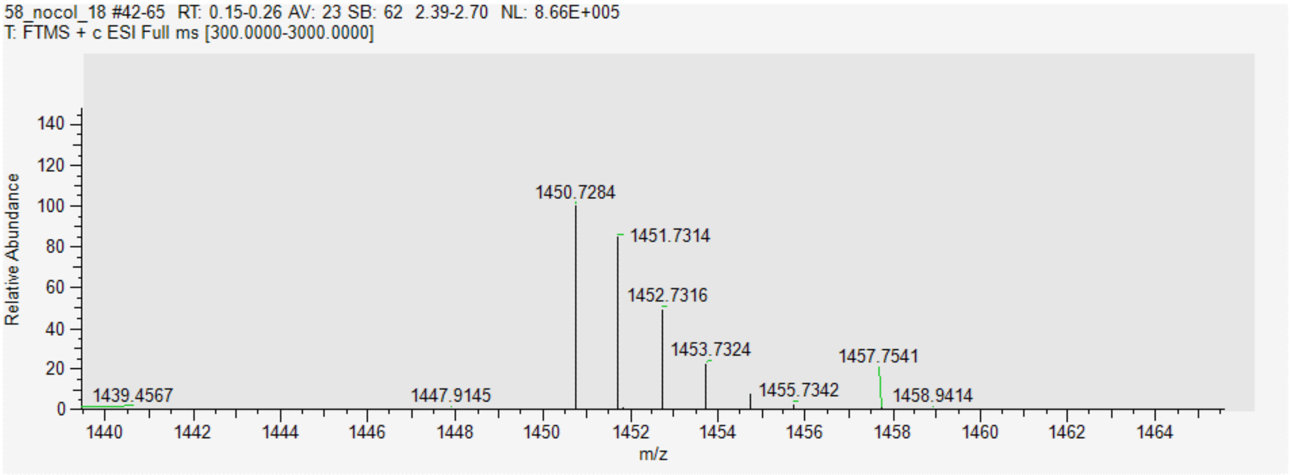

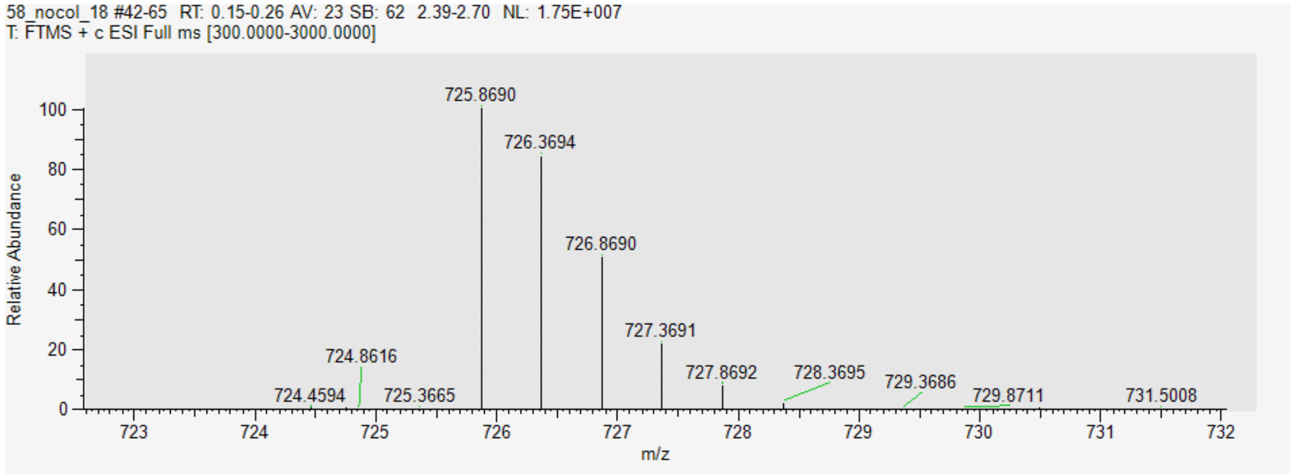

58_nocol_18 #42-65 RT: 0.15-0.26 AV: 23 SB: 62 2.39-2.70 NL: 1.75E+007
T: FTMS + c ESI Full ms [300.0000-3000.0000]
Relative Abundance
m/z
725.8690
726.3694
726.8690
727.3691
724.8616
727.8692
728.3695
729.3686
724.4594
725.3665
729.8711
731.5008


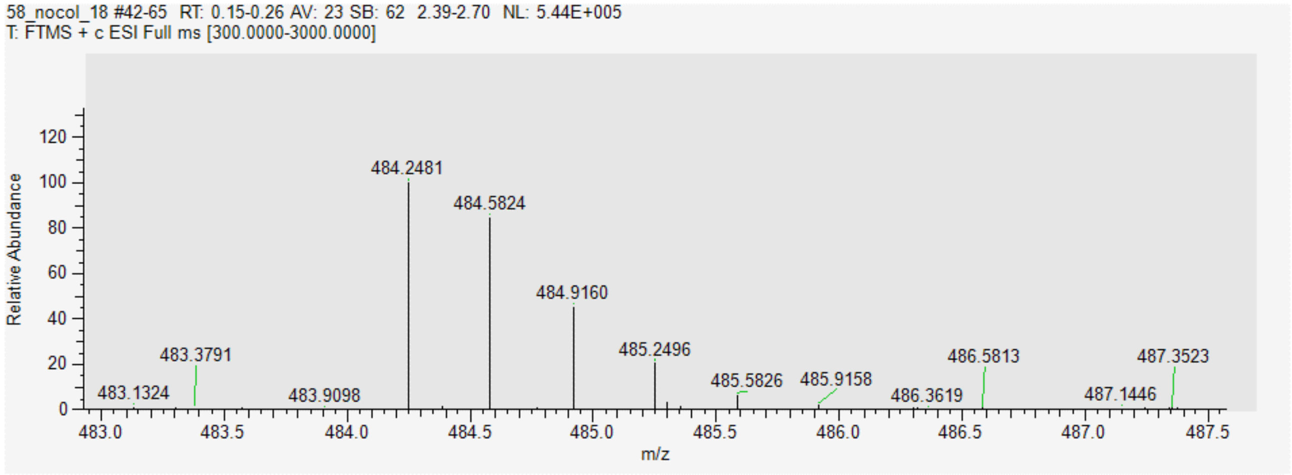

58_nocol_18 #42-65 RT: 0.15-0.26 AV: 23 SB: 62 2.39-2.70 NL: 5.44E+005
T: FTMS + c ESI Full ms [300.0000-3000.0000]
Relative Abundance
m/z
484.2481
484.5824
484.9160
483.3791
485.2496
486.5813
487.3523
485.5826
485.9158
483.1324
483.9098
486.3619
487.1446

**Compound 59**

MS (ESI): m/z calcd for $C_{54}H_{83}N_9O_{10}S$ $[M+H]^+$ 1051.36 found 1051.16. $t_R$ = 23.57

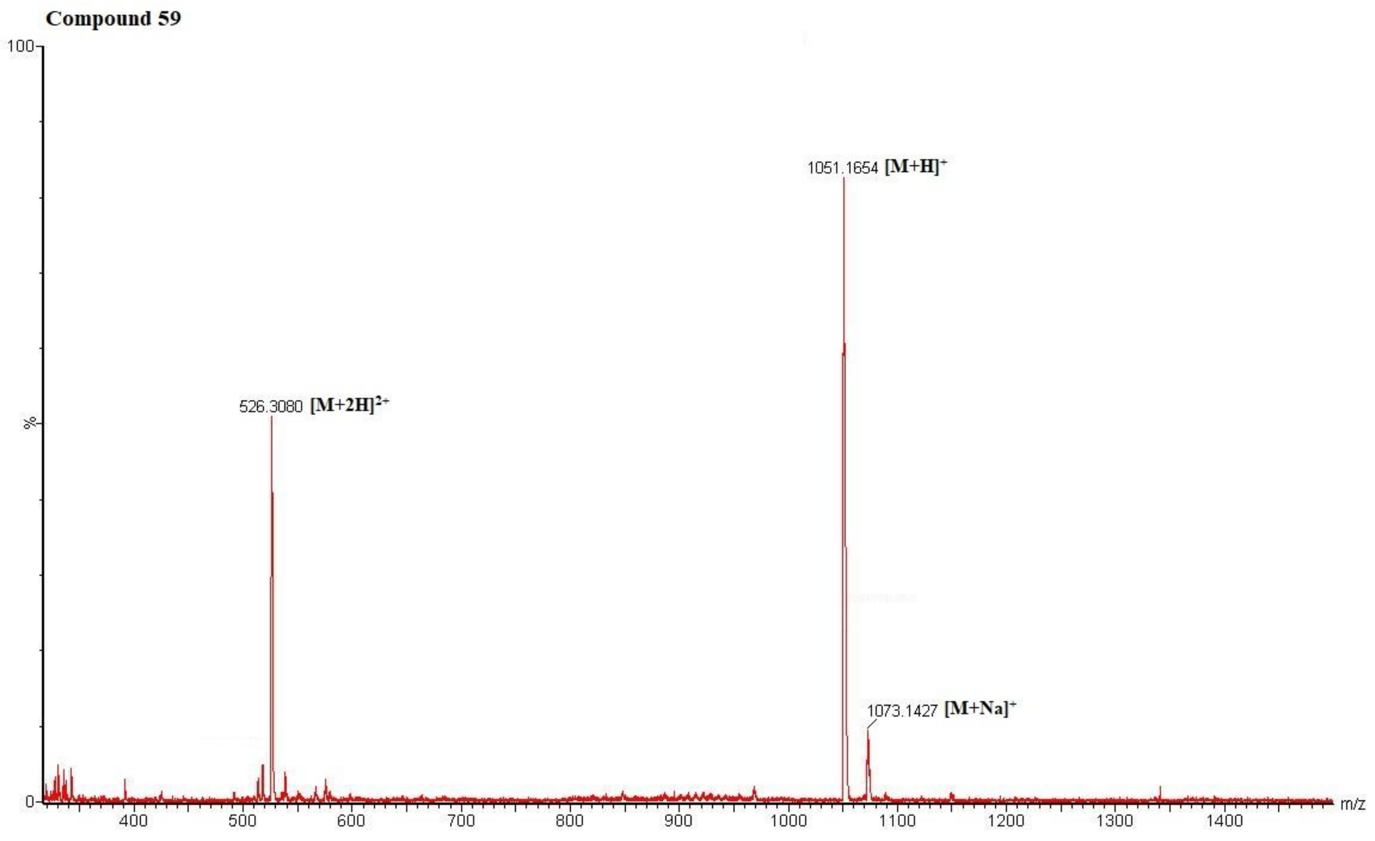


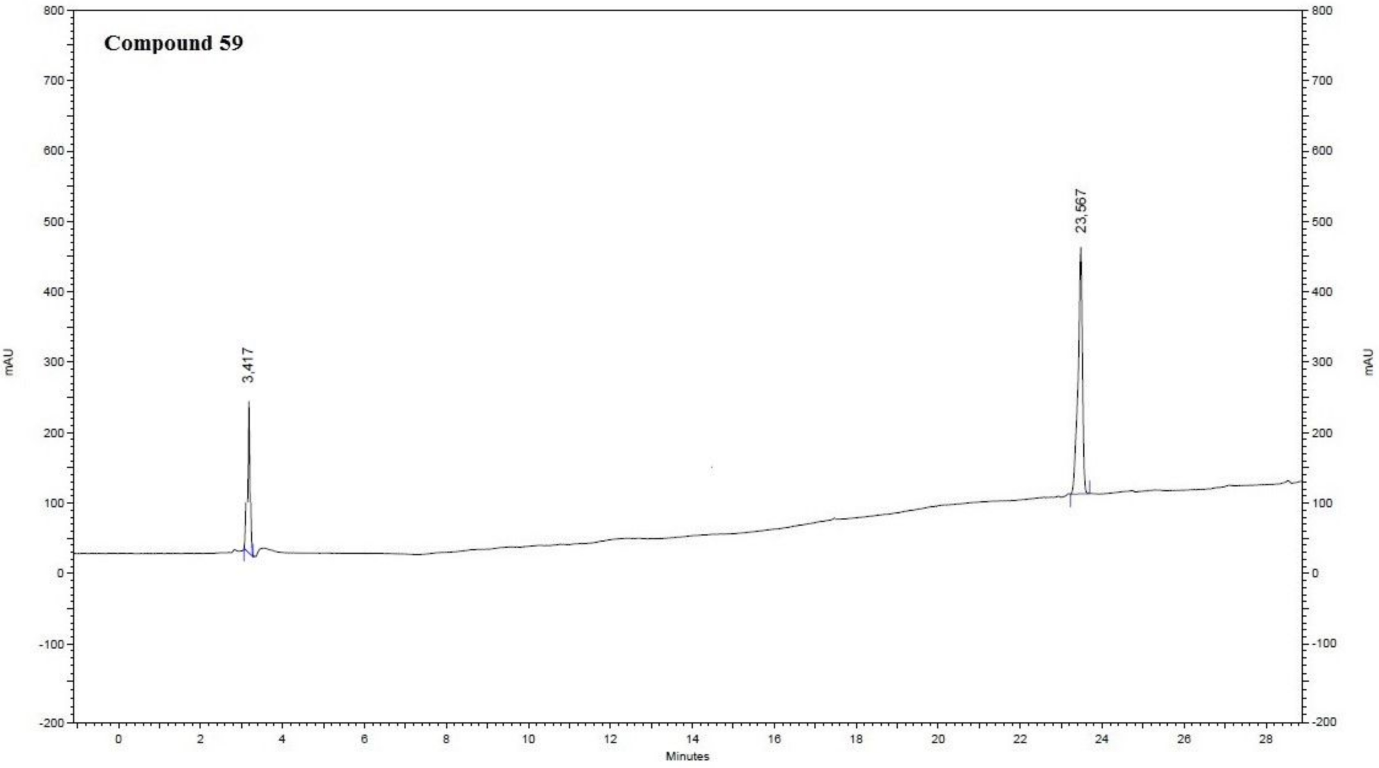

## Compound 59 (HRMS analysis)

| molecular formula | exact mass | | |
|---|---|---|---|
| $C_{54}H_{83}N_{9}O_{10}S$ | 1049,59836 | | |
| molecular ion | m/z (teo) | m/z (exp) | dm/z (ppm) |
| $[M+H]^{+}$ | 1050,60564 | 1050,6045 | 1,1 |
| $[M+2H]^{2+}$ | 525,80646 | 525,8042 | 4,3 |

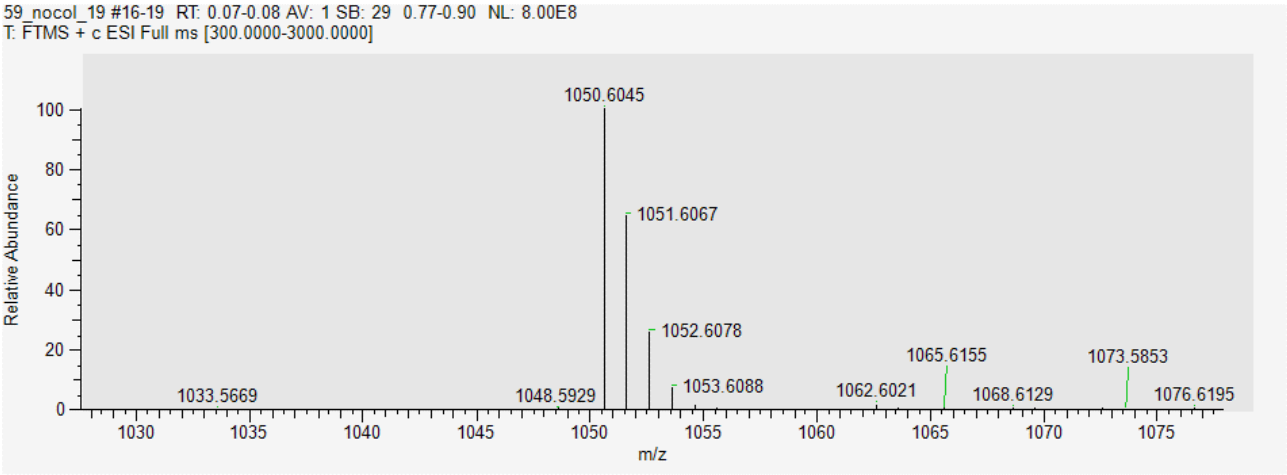


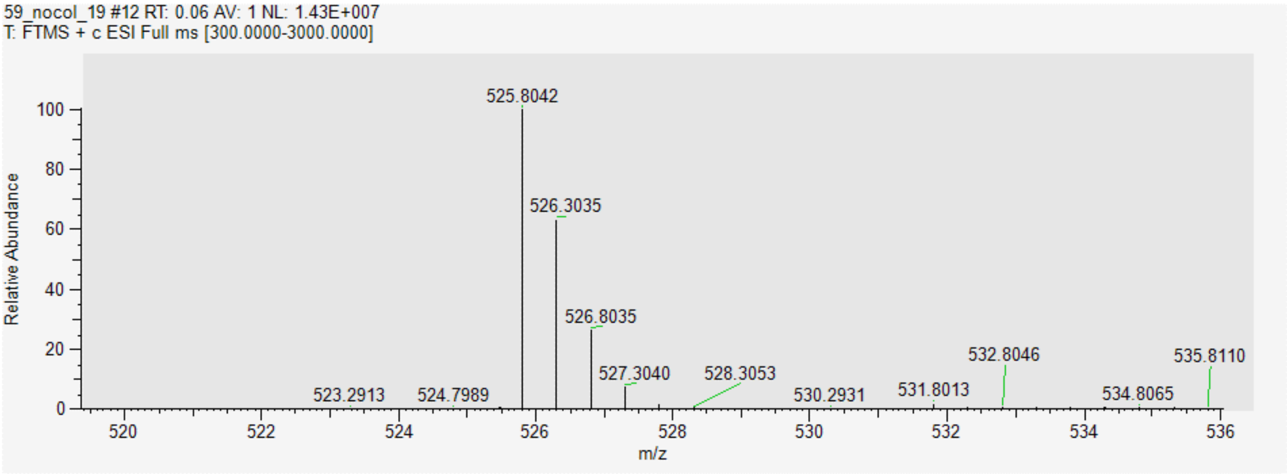

Supplement: Supplementary file 1 — jm9b02057_si_001.pdf [file jm9b02057_si_001.pdf]
